# Final analysis of the ZOE-LTFU trial to 11 years post-vaccination: efficacy of the adjuvanted recombinant zoster vaccine against herpes zoster and related complications

**DOI:** 10.1016/j.eclinm.2025.103241

**Published:** 2025-05-09

**Authors:** Ana Strezova, Javier Díez Domingo, Anthony L. Cunningham, Takashi Eto, Charles Andrews, Clovis Arns, Eun-Ju Choo, David Shu Cheong Hui, Giancarlo Icardi, Shelly A. McNeil, Airi Põder, Pavel Kosina, Lars Rombo, Tino F. Schwarz, Juan Carlos Tinoco, Chong-Jen Yu, Jing Wang, Jyoti Soni, Manyee Tsang, Rafael Leon, Agnes Mwakingwe-Omari, Zoster-049 Study Group, Abul Kashem Munir, Agnes Csuth, Agnes Himpel-Boenninghoff, Aino Forsten, Airi Poder, Ake Olsson, Alain Baty, Alain Boye, Alen Jambrecina, Alex Rodríguez Badia, Alexander Thompson, Andrea Gori, Anitta Ahonen, Anna Vilella Morato, Anthony Cunningham, Antje Dahmen, Axel Schaefer, Azhar Toma, Barry Lubin, Beate Moeckesch, Beatrice Gerlach, Benita Ukkonen, Benjamin Lasko, Benoit Daguzan, Bernhard Schmitt, Bo Liu, Brian Webster, Bruce Rankin, Calvin Powell, Carlos Brotons Cuixart, Carol Pretswell, Catherine Vaillancourt, Cecil Farrington, Charles Andrews, Chester Fisher, Chigomezgo Munthali, Chiu-Shong Liu, Chong-Jen Yu, Christian Duroy, Christian Schubert, Christiane Klein, Christine Cerna, Christine Grigat, Christophe Genies, Christopher Lucasti, Cláudia Murta de Oliveira, Claus Keller, Clovis Cunha, Concepcion Nunez Lopez, Covadonga Caso, Cristiano Zerbini, Dae Won Park, Damien Mcnally, Dan Curiac, David Francyk, David García Vidal, David Shu Cheong Hui, Denis Taminau, Domenico Montu, Dominique Saillard, Donald Quinn, Duane Wombolt, Edmund Kwok Yiu Sha, Elina Sirnela-Rif, Elisabeth Barberan, Eric St-Amour, Eriko Kinugasa, Ernie Riffer, Essam Abdulhakim, Eugene Athan, Eun-Ju Choo, Eva Ackefelt Frick, Felix Proepper, Ferdinandus de Looze, Francesco Schioppa, François Brault, Frederick Martin, Gabriele Illies, Georg Plassmann, George Freeman, George Raad, Gerald Shockey, Giancarlo Icardi, Giuseppe Fiore, Guglielmo Migliorino, Guy Tellier, Hanna Karhusaari, Hans-Joachim Koenig, Hee Jin Cheong, Hiroaki Ogata, Hirohiko Sueki, Holly Dushkin, Hsiao-Ting Chang, Huey-Shinn Cheng, Hyo Youl Kim, Ignacio Bardón Fernández-Pacheco, Ilkka Seppa, Irina Zahharova, Iris Gorfinkel, Isaac Marcadis, Isabelle Schenkenberger, Jacob Lee, Jan Dutz, Janice Patrick, Javier Diez-Domingo, Jean Beytout, Jean-Sebastien Gauthier, Jeannette Janzen, Jeffrey Zacher, Jérôme Nuel, Jessica Claveau, Jin-Soo Lee, Joachim Minnich, Joachim Sauter, Joakim Aronsson, Joan Rothenberg, Johan Sanmartin Berglund, John Earl, John Ervin, Jonathan Staub, Jonathan Wilson, José Luiz Neto, José Ramón Méndez Rivas, Jose-Fernando Barba-Gómez, Josef Grosskopf, Juan Carlos Tinoco, Juergen Berger-Roscher, Juergen Schmidt, Juergen Stockhausen, Juergen Wachter, Jukka Markkula, Junya Irimajiri, Jurij Eremenko, Kae Kobayashi, Karl Wilhelm, Karlis Pauksens, Katarina Berndtsson Blom, Ken Heaton, Kenjiro Nakamura, Kyong Ran Peck, Lars Rombo, Lauri Peltonen, Laurie Breger, Lluis Martínez Via, Loïc Boucher, Luciano Goldani, M Luisa Rodrguez de la Pinta, Mahadev Ramjee, Maija Rossi, Manuel Terns Riera, Marc Dionne, Margaret Rhee, Maria Giuseppina Desole, Maria Hemming-Harlo, Maria Maestre Naranjo, Marita Paassilta, Marjaana Sipila, Mark Turner, Marshall Freedman, Marta Aldea Novo, Martin Lundvall, Martin Van Cleeff, Mary Beth Manning, Matthew Finneran, Maximilian Kropp, Megumi Inoue, Meral Esen, Merce Perez Vera, Michael Adams, Michael Mueller, Michael Redmond, Miia Virta, Monika Hamann, Murdo Ferguson, Nell Wyatt, Nicolas Galerne, Nicole Toursarkissian, Niklas Bengtsson, Noah Vale, Olli Henriksson, Otso Arponen, Outi Laajalahti, Pascal Hanrion, Patrice Nault, Patrick Robert, Paul Hartley, Paul Ivan, Paula Gyllemark, Pauliina Paavola, Pavel Kosina, Pavel Naplava, Pekka Koskinen, Pembe Ozunlu, Peter Eizenberg, Peter Gal, Peter Levins, Petr Dite, Philippe Remaud, Piero Barbanti, Pierre André Ferrand, Pierre Lachance, Pierre-Alain Houle, Pyrene Martinez Piera, Ralf Freese, Rie Kuroki, Robert Lipetz, Robert Rosen, Roman Chlibek, Samir Purnell-Mullick, Satu Kokko, Scott Polster, Shari Rozen, Shelly McNeil, Shin Suzuki, Shinn-Jang Hwang, Silvia Narejos Perez, Spyridon Miyakis, Srikanth Malempati, Stephan Morscher, Stephanie Powell, Steve Mueller, Steven Geller, Suganthi Luci Magimaiseelan, Susan Datta, Susanna Koski, Susannah Eyre, Susanne Hoeltz-Roehrig, Suvi-Tuuli Simojoki, Sylvia Shoffner, Takashi Eto, Tamara Eckermann, Tark Kim, Terry Poling, Tetsuhiko Nagao, Thomas Horacek, Thomas Jung, Thomas Weinke, Tiina Haapaniemi, Tiina Karppa, Tiina Korhonen, Tino Schwarz, Tommaso Staniscia, Trevor Gooding, Uwe Kleinecke, Wayne Ghesquiere, Wilfred Yeo, William Ellison, Wilson Jacob, Xavier Farres Fabre, Yieng Huong, Young Goo Song, Yuji Naritomi, Agnes Mwakingwe-Omari, Alemnew F. Dagnew, Amy Tan, Ana Strezova, Anne Schuind, Andrew Hastie, Bruno Salaun, Céline Boutry, Emeline de Viron, Emmanuel Di Paolo, Hao Wang, Huizi Zhang, Joon Hyung Kim, Kamal Al Shawafi, Lidia Oostvogels, Mamadou Drame, Martine Douha, Mélanie Gilbert, Meng Shi, Mohamed Amakrane, Mohd Tariq, Nurhan Albayrak, Olivier Godeaux, Paola Pirrotta, Toufik Zahaf

**Affiliations:** a GSK, Wavre, Belgium; b FISABIO, Valencia, Spain; c Catholic University of Valencia, Spain; d Centre for Virus Research, Westmead Institute for Medical Research, Australia; e University of Sydney Institute for Infectious Diseases, Faculty of Medicine and Health, University of Sydney, Australia; f Hakata Clinic, Fukuoka, Japan; g IMA Clinical Research San Antonio, San Antonio, TX, USA; h Centro Medico Sao Francisco, Curitiba, Brazil; i Soonchunhyang University Bucheon Hospital, South Korea; j The Chinese University of Hong Kong, Hong Kong, China; k Hygiene Unit, IRCCS San Martino Policlinico Hospital, Genoa, Italy; l Department of Health Sciences (DISSAL), University of Genoa, Italy; m Canadian Center for Vaccinology, IWK Health, NS Health, Dalhousie University, Halifax, Nova Scotia, Canada; n Tartu University Hospital, Tartu, Estonia; o Dept. of Infectious Diseases, University Hospital and Faculty of Medicine, Hradec Kralove, Czech Republic; p Centre for Clinical Research Sormland, Uppsala University, Sweden; q Institute of Laboratory Medicine and Vaccination Centre, Klinikum Würzburg Mitte, Campus Juliusspital, Würzburg, Germany; r Hospital General de Durango, Durango, Mexico; s National Taiwan University College of Medicine and National Taiwan University Hospital, Taipei, Taiwan; t GSK, Rockville, MD, USA; u GSK, London, UK

**Keywords:** Recombinant zoster vaccine, Herpes zoster, Post-herpetic neuralgia, Long-term follow-up

## Abstract

**Background:**

Herpes zoster (HZ) vaccines should provide durable protection against HZ and HZ-related complications. We report the final analysis of a long-term follow-up (LTFU) study (ZOE-LTFU) including 11 years of follow-up after primary vaccination with recombinant zoster vaccine (RZV).

**Methods:**

ZOE-LTFU (NCT02723773) was an open-label, phase 3b study following participants of two phase 3 trials, ZOE-50 and ZOE-70. ZOE-LTFU started approximately 5 years post-vaccination in ZOE-50/70 and participants were followed for 6 years. The primary objective was to assess vaccine efficacy (VE) against HZ during ZOE-LTFU. Secondary objectives included VE against HZ from 1 month post-dose 2 in ZOE-50/70 until end of ZOE-LTFU, VE against post-herpetic neuralgia (PHN) and non-PHN complications, immunogenicity, and long-term safety. The VE calculation used a historical control constructed with ZOE-50/70 placebo data.

**Findings:**

VE was assessed in the modified total vaccinated cohort (n = 7273 [mean age 67·3 years at first vaccination]). During ZOE-LTFU, VE was 79·8% (95% confidence interval [CI]: 73·7, 84·6) and 73·2% (95% CI: 62·9, 80·9) against HZ in participants ≥50 and ≥70 years at first vaccination, respectively, and was 87·5% (95% CI: 64·8, 96·8) against PHN and 91·7% (95% CI: 43·7, 99·8) against other HZ-related complications in participants ≥50 years. From 1 month post-dose 2 in ZOE-50/70 to the end of ZOE-LTFU, VE against HZ was 87·7% (95% CI: 84·9, 90·1) in participants ≥50 years and sustained at 82·0% (95% CI: 63·0, 92·2) in the eleventh year post-vaccination. Humoural and cell-mediated immune responses plateaued at over 5-fold and ∼7-fold, respectively, above pre-vaccination levels in ZOE-50/70. No RZV-related serious adverse events occurred.

**Interpretation:**

Efficacy of RZV against HZ and associated complications remained high through 11 years post-vaccination, indicating sustained clinical benefit.

**Funding:**

The funder of the study was 10.13039/100004330GSK who was involved in study design, data collection, data analysis, data interpretation, writing of the report, and the decision to submit for publication.

**Research in context**

**Evidence before this study**

We searched PubMed (1 January 2004–30 September 2024) and the Cochrane library (1 January 2014–30 September 2024) using the terms (herpes zoster OR varicella-zoster virus) AND (vaccine OR vaccination) AND (protection OR efficacy OR immunogenicity) AND (adults OR elderly OR older adults). The live attenuated zoster vaccine (zoster vaccine live, ZVL) demonstrated vaccine efficacy (VE) of 51·3% against herpes zoster (HZ) up to 5 years after vaccination in adults vaccinated at ≥60 years, but efficacy waned substantially over time. Phase 3 trials of the recombinant zoster vaccine (RZV) demonstrated vaccine efficacy against HZ of 97·2% in adults vaccinated at ≥50 years and 91·3% in those vaccinated at ≥70 years over 3–4 years of follow-up.

**Added value of this study**

A long-term follow-up (LTFU) study of the Phase 3 ZOE-50 and ZOE-70 trials (ZOE-LTFU), in which more than 7000 participants were followed for up to 11 years after primary vaccination, demonstrated VE against HZ of 79·8% in adults vaccinated at ≥50 years of age and 73·2% in those vaccinated at ≥70 years of age. VE over the same period against post-herpetic neuralgia, the most common complication of HZ, was 87·5% in participants vaccinated at ≥50 years. During the period from 1 month after the second vaccine dose in ZOE-50/70 to the end of ZOE-LTFU, VE against HZ was 87·7% in participants vaccinated at ≥50 years and was sustained at 82·0% in the eleventh year post-vaccination.

**Implications of all the available evidence**

ZOE-LTFU showed that RZV offers durable protection against HZ and associated complications through approximately 11 years post-vaccination, with a clinically acceptable safety profile. The efficacy of RZV has led some countries to introduce preferential recommendations in favour of RZV, some that did not previously recommend HZ vaccination to include RZV in their routine immunisation programmes, and some to lower the recommended age of vaccination. Data from the ZOE-LTFU study, including high efficacy observed in all age groups and minimal waning of VE over time, might support further introductions and expansion of vaccination to all age groups ≥50 years.

## Introduction

Herpes zoster (HZ), also known as shingles, is caused by the varicella zoster virus (VZV).^1^^,^^2^ Primary infection with VZV causes varicella (chickenpox), usually in children. During primary infection, the virus enters sensory nerves and becomes latent; reactivation of the latent virus causes HZ, usually in older adults.^1^^,^^2^ The incidence of HZ increases with age.^3^

HZ often begins with a prodromal phase with pain, itching, and numbness, followed by a blistering rash that can leave residual scarring.^3^^,^^4^ HZ can result in complications including post-herpetic neuralgia (PHN), disseminated infections, secondary bacterial infection, ophthalmic and neurological complications, and vasculopathy.^3^ PHN, defined as pain persisting for >90 days after commencement of the HZ rash, is the most frequent complication of HZ and its incidence rises with older age.^5^ It is difficult to manage and can last for months to years, with a substantial impact on quality of life.^5^^,^^6^

A large proportion of the population is at risk for HZ and HZ-related complications, especially as populations are ageing in most parts of the world. Vaccines against HZ are approved from 50 years of age; thus, it is important to demonstrate that vaccinated individuals continue to be protected as they grow older, as the risk of HZ and associated complications rises with age. Long term follow-up of the first licenced HZ vaccine (live attenuated zoster vaccine [zoster vaccine live, ZVL, *Zostavax*, MSD]) indicated that vaccine efficacy (VE) waned substantially over time and was 51·3%, 39·6%, and 21·1% for follow-up periods of 0–4·9 years, 3·3–7·8 years, and 4·7–11·6 years.^7^ Real-world cohort studies have also demonstrated a decline in effectiveness of ZVL over time.^8^^,^^9^ More recently, an adjuvanted recombinant zoster vaccine (RZV; *Shingrix*, GSK) has been approved in more than 70 countries and is composed of a recombinant VZV glycoprotein E and the Adjuvant System (AS) 01_B_. It is indicated for the prevention of HZ in adults ≥50 years, as well as adults ≥18 years at increased risk of HZ.^10^

In this article, we focus on long-term protection in adults ≥50 years of age. The ZOE-50 and ZOE-70 pivotal phase 3 trials reported VE of 97·2% and 91·3% against HZ and 91·2% and 88·8% against PHN, in adults ≥50 and ≥70 years of age, respectively, over 3–4 years of follow-up.^11^^,^^12^ While ZOE-50 and ZOE-70 demonstrated that RZV was highly efficacious, longer term studies would be helpful to better understand the durability of protection with RZV. We therefore conducted a long-term follow-up (LTFU) extension study of ZOE-50 and ZOE-70 (ZOE-LTFU) in which participants who received RZV were followed for up to approximately 11 years after primary vaccination. Participants who received placebo in the ZOE-50/70 studies were offered vaccination with RZV as part of another study^13^ and were ineligible to participate in ZOE-LTFU. Because a concurrent placebo group was no longer available for the ZOE-LTFU study, a historical control constructed with ZOE-50/70 placebo data was used for the VE calculation for ZOE-LTFU. Two interim analyses of the ZOE-LTFU study have reported VE against HZ for the overall population of 84·0% over approximately 5·1–7·1 years post-vaccination and 81·6% over approximately 5·6–9·6 years post-vaccination.^14^^,^^15^ Here, we report the final analysis of the ZOE-LTFU study.

## Methods

### Study design

ZOE-LTFU (NCT02723773) was an open-label, phase 3b study that followed participants of the pivotal phase 3 trials of RZV (ZOE-50 in participants ≥50 years, NCT01165177 and ZOE-70 in participants ≥70 years, NCT01165229). The study used a historical control which was constructed using ZOE-50/70 placebo data. It was conducted in 18 countries or regions (Australia, Brazil, Canada, Czech Republic, Estonia, Finland, France, Germany, Hong Kong, Italy, Japan, Mexico, Republic of Korea, Spain, Sweden, Taiwan, the United Kingdom, and the United States of America).

The protocol was approved by necessary independent ethics committees or institutional review boards. The study was conducted in accordance with the Declaration of Helsinki and the principles of Good Clinical Practice. Participants provided written, informed consent at enrolment.

### Participants and vaccine

Participants were eligible for the ZOE-LTFU study if they had received at least one dose of RZV in ZOE-50 or ZOE-70. Similar to the ZOE-50/70 studies, participants with an immunosuppressive or immunodeficient condition or chronic administration of immunosuppressant drugs were excluded, as were those receiving immunoglobulins, blood products, or long-term antiviral agents. Participants who were immunodeficient or immunosuppressed due to disease or therapy were excluded at study entry; however, if they became immunocompromised or immunosuppressed during the ZOE-LTFU study, they were included in the analysis of efficacy. Participants with comorbid conditions common to the study population (e.g., hypertension, osteoarthritis and/or vertebral disorders, dyslipidaemia, diabetes, osteoporosis/osteopenia) were eligible to participate. Full eligibility criteria are shown in Appendix p5.

RZV comprises 50 μg of recombinant VZV glycoprotein E (gE) and the AS01_B_ Adjuvant System which is a liposome-based adjuvant comprising 50 μg 3-*O*-desacyl-4′-monophosphoryl lipid A and 50 μg QS-21, a saponin from the *Quillaja saponaria* Molina tree.^16^

### Study timelines

**Fig. 1:**
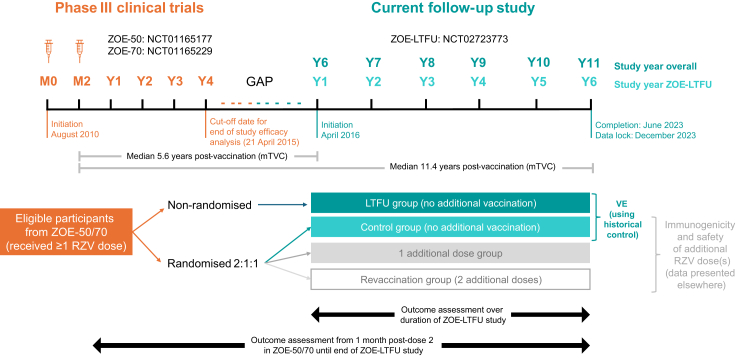
**Study design**. The gap period between the cut-off date for the end of study efficacy analysis in the ZOE-50/70 studies (21 April 2015) and the first visit in the ZOE-LTFU study varied between participants depending on the timing of their first visit in ZOE-LTFU. The median duration of the gap period was 1·4 years (range 0·9–2·2 years). LTFU, long term follow-up; mTVC, modified total vaccinated cohort; VE, vaccine efficacy.

The ZOE-50/70 studies encompassed a median follow-up of 4·4 years.^17^ There was a gap period between the cut-off date for the end of study efficacy analysis in the ZOE-50/70 studies (21 April 2015) and the first visit in the ZOE-LTFU study (Fig. 1). Since the gap between the end of the ZOE-50/70 studies and the start of the ZOE-LTFU study varied between participants, most participants had 11 years of follow-up, but some completed 12 years.

### Study groups

There were four study groups. Most participants were enrolled in the LTFU group and received no additional vaccination as planned in the protocol (Fig. 1). The other three groups were randomised to receive either one additional RZV dose, two additional doses, or no additional vaccination (control group). The randomised control group served two purposes. Firstly, it acted as a control for the randomised groups in an evaluation of the immunogenicity and safety of additional dose(s) of RZV; these data are reported elsewhere. Secondly, as the randomised control group did not receive additional vaccines in ZOE-LTFU, data from this group were combined with data from the LTFU group, which also received no additional vaccines. The analysis of VE was done in this combined group (LTFU and control), using historical control data as the comparator; this is the main focus of the present article. Long-term immunogenicity was analysed in a subset of the LTFU group. The present article also focuses on long-term safety in the LTFU and control groups.

### Outcomes

The primary objective was to assess VE against the first or only episode of HZ over the duration of the ZOE-LTFU study. The protocol included a list of 19 secondary outcome measures; we report data for 10 of these outcomes here and in Appendix p11–26. The outcomes for which data are not reported in this article can be found at clinicaltrials.gov (https://clinicaltrials.gov/study/NCT02723773?tab=results). Secondary objectives included assessment of VE against the first or only episode of HZ from 1 month post-dose 2 in ZOE-50/70 until the end of ZOE-LTFU and VE against the first or only episodes of PHN and non-PHN complications over the duration of ZOE-LTFU and from 1 month post-dose 2 in ZOE-50/70 until the end of ZOE-LTFU. VE was assessed overall and by age group at first vaccination; VE against HZ was also assessed annually. Other secondary objectives were persistence of humoural immunogenicity (HI) and cell-mediated immunogenicity (CMI) at yearly intervals over the duration of the ZOE-LTFU study and safety. A full list of secondary objectives is provided in Appendix p6–7.

#### Case definitions of HZ, PHN, and non-PHN complications

Cases of HZ and PHN were assessed as in the ZOE-50 and ZOE-70 studies.^11^^,^^12^ A suspected case of HZ was defined as new unilateral rash accompanied by pain (broadly defined to include allodynia, pruritus or other sensations) and no alternative diagnosis. Cases were confirmed by polymerase chain reaction from rash lesion samples or by ascertainment committee in the same way as in ZOE-50 and ZOE-70.^11^^,^^12^

HZ-associated pain was evaluated by the Zoster Brief Pain Inventory (ZBPI)^18^ questionnaire in participants experiencing HZ. PHN was defined as the presence of HZ-associated ‘worst’ pain rated as ≥3 on the ZBPI questionnaire, persisting or appearing more than 90 days after onset of the HZ rash. Non-PHN complications included HZ vasculitis, disseminated disease (≥6 lesions outside the primary dermatome), ophthalmic, neurological, or visceral disease, and stroke (full definitions are shown in Appendix p8).

#### Assessment of immunogenicity

HI was assessed by the concentration of anti-gE antibodies measured by enzyme-linked immunosorbent assay (cut-off 97 mIU/mL). CMI was assessed by the frequency of gE-specific CD4+ T cells expressing at least two of four activation markers (interferon-γ, tumour necrosis factor-α, interleukin-2, and CD40 ligand), measured by intracellular cytokine staining and flow cytometry after stimulation with a pool of overlapping peptides covering the entire gE sequence.

#### Assessment of long-term safety

This article reports long-term safety in participants who received no additional vaccination in ZOE-LTFU (LTFU and control). Occurrence of serious adverse events (SAEs) related to investigational vaccine, study participation (i.e., study-mandated procedures and invasive tests), or to a concurrent GSK medication or vaccine were analysed. In addition, adverse events (AEs) and SAEs leading to withdrawal and related to investigational vaccine, study participation, a concurrent GSK medication or vaccine, or HZ-associated complications were analysed, as well as other reasons for study withdrawal.

### Statistical analysis

Statistical analyses were performed using the Statistical Analysis Systems (SAS) Life Science Analytics Framework (SAS Institute Inc, Cary, NC, USA).

#### Analysis cohorts

The total vaccinated cohort (TVC) included all participants enrolled in the ZOE-LTFU study who received at least one RZV dose in ZOE-50/70, provided informed consent, and had data available. Long-term safety was assessed in the TVC (LTFU and control groups). The primary cohort for VE analysis was the modified TVC (mTVC) which comprised participants in the LTFU group and control group who received both RZV doses in ZOE-50/70 and had not developed HZ within 1 month after the second vaccination in ZOE-50/70.

The primary cohort for the persistence of immunogenicity analysis was the according to protocol (ATP) cohort adapted for each yearly timeframe in the HI and CMI subsets of the LTFU group. The ATP cohort was defined for each timepoint and comprised participants who received two RZV doses in ZOE-50/70, complied with protocol requirements in ZOE-50/70 and ZOE-LTFU, and had immunogenicity data available for the timepoint considered (further details in Appendix p9).

#### Sample size determination

The sample size was driven by the approximate number of participants expected to participate in the ZOE-LTFU study (i.e., approximately 6000 participants from ZOE-50/70). Because no concurrent placebo group was available in ZOE-LTFU, an approximate power computation was performed considering the incidence rate in the vaccine group as compared with the incidence rate of the historical control from the ZOE-50/70 studies. This sample size allowed a lower limit of the 95% confidence interval (CI) of the VE estimate of >30% to be achieved with a probability of ≥99%. The study was not designed for statistical testing and all statistical analyses were descriptive.

#### VE analysis

To construct the historical control, age stratum specific incidence rates (per age at vaccination at ZOE-50/70) were estimated with a Poisson regression model (adjusted for region), and the resulting incidence rates were multiplied by the actual follow-up time observed in the ZOE-LTFU study to obtain the predicted number of HZ cases for the historical control. The same sample size and follow-up time as the vaccinated group were assumed for the historical control to ensure that the two groups were comparable. The same approach was taken for historical control construction in both yearly and overall VE calculations.

A Poisson regression model was used to estimate VE and associated 95% CIs overall and by age strata (50–59, 60–69, 70–79, ≥70, and ≥80 years of age at vaccination), considering region as a fixed effect. The time at risk for each participant ended at the time of the first or only event of interest; or at the time of receiving any non-protocol HZ vaccination; or at the latest visit for which data were available for participants who did not experience an event (i.e., the earliest of the date of withdrawal, death, or study conclusion). For the annual VE estimates and the VE estimate from 1 month after the second vaccine dose to the end of the ZOE-LTFU study, calculations for Year 1–Year 4 used actual incidence rates from the ZOE-50/70 placebo arm, and calculations for Year 6 onwards used the estimated rates for the historical control. Any suspected HZ cases occurring during the gap period were recorded, but were not considered in the VE analysis of ZOE-LTFU, because HZ cases could not be confirmed per protocol requirements. Hence, it was not possible to estimate VE during the gap period. A sensitivity analysis including HZ cases identified during the gap period was also done.

Yearly follow-ups were computed from 1 month post-dose 2 in the ZOE-50/70 studies. Year 12 was pooled with Year 11 for the purpose of the VE calculation. Participants who developed HZ, PHN, or a non-PHN complication during ZOE-50/70 were not considered in the respective VE analysis calculated only over the duration of the ZOE-LTFU study; however, the analysis from 1 month post-dose 2 in ZOE-50/70 until the end of ZOE-LTFU did include events that had occurred during ZOE-50/70.

The incidence rate of the first or only episode of confirmed HZ by number of comorbidities at ZOE-LTFU baseline was also computed. Participants who developed HZ during ZOE-50/70 were not considered, as this calculation was done for the duration of the ZOE-LTFU study only.

#### Immunogenicity analysis

Since the interval between the end of the ZOE-50/70 studies and the start of the ZOE-LTFU study varied between participants, the timeframe of the first visit in ZOE-LTFU was computed as the time between the date of the second dose administration in ZOE-50/70 and the date of the first visit in ZOE-LTFU. For each subsequent yearly visit, 1 year was added to the timeframe of the first visit.

The HI response was reported in terms of seropositivity rate (percentage of participants with anti-gE antibody concentration ≥97 mIU/mL [cut-off value]), geometric mean concentration (GMC) of anti-gE antibody, vaccine response rate (VRR; percentage of participants with post-vaccination anti-gE antibody concentration ≥4-fold the cut-off value for initially seronegative participants and ≥4-fold the pre-vaccination value for initially seropositive participants), and mean geometric increase (MGI; ratio of the GMC post-vaccination/pre-vaccination). Pre-vaccination values were from ZOE-50/70. GMCs were calculated by taking the anti-log of the mean of the log concentration transformations. Concentrations below the assay cut-off level were given an arbitrary value of half the cut-off level.

The CMI response was reported in terms of the frequency of CD4+ T cells expressing ≥2 activation markers (CD4[2+]), calculated as the difference between the frequency of CD4[2+] T cells stimulated with gE peptides and those stimulated with culture medium alone. Descriptive statistics (mean, standard deviation) were reported.

### Role of the funding source

The funder of the study was GSK who was involved in study design, data collection, data analysis, data interpretation, writing of the report, and the decision to submit for publication.

## Results

The ZOE-50/70 studies took place between August 2010 and July 2015; ZOE-LTFU took place between April 2016 and June 2023 (Fig. 1). Data lock for ZOE-LTFU was December 2023.

### Participant disposition and demographics

**Fig. 2:**
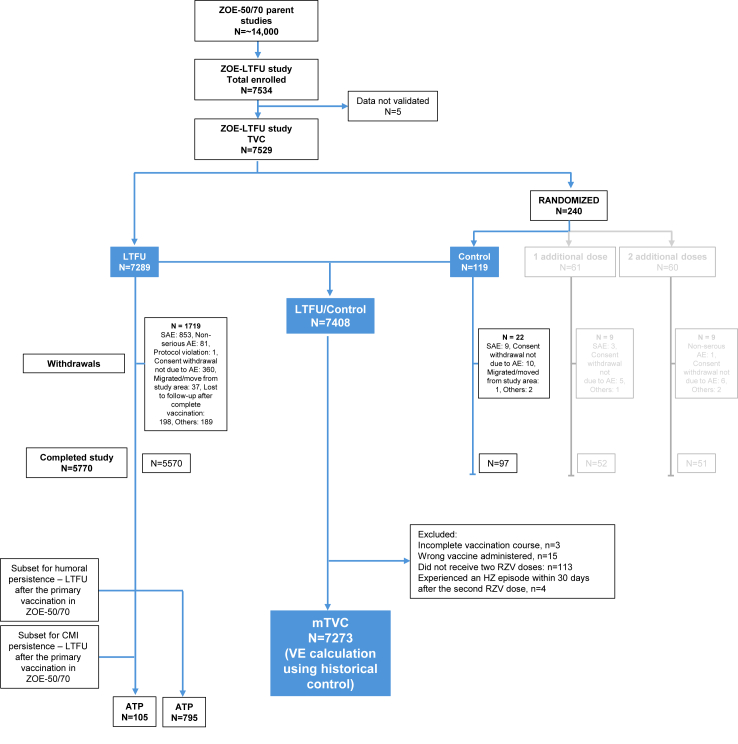
**Participant disposition**. Data on immunogenicity and safety of additional RZV dose(s) (greyed out boxes) will be presented elsewhere. AE, adverse event; ATP, according to protocol; CMI, cell-mediated immunogenicity; HZ, herpes zoster; LTFU, long term follow-up; mTVC, modified total vaccinated cohort; SAE, serious adverse event; TVC, total vaccinated cohort.

Over 14,000 participants who had received ≥1 dose of RZV in ZOE-50/70 were eligible for the ZOE-LTFU study. A total of 7529 participants were included in the TVC and 7273 in the mTVC (Fig. 2). A total of 5770 participants completed the study (Fig. 2).

In the mTVC, the mean age of participants was 67·3 years at first vaccination in ZOE-50/70 and 72·5 years at the start of ZOE-LTFU, 60·7% were female, 76·0% were of White Caucasian or European heritage, and 14·9% were of East Asian heritage (Table 1). A total of 84·5% of participants had ≥1 comorbidity (from a list of pre-defined selected medical conditions), 41·7% had ≥3 comorbidities, and 23·7% had ≥4 comorbidities (Table 1).

**Table 1:** Summary of demographic characteristics in the mTVC.

Characteristic	Participants (LTFU + control) N = 7273
Age at first vaccination in ZOE-50/70 studies, years, mean (SD)	67·3 (9·5)
Age at month 0 in ZOE-LTFU study, years, mean (SD)	72·5 (9·4)
Gender, n (%)	
Female	4417 (60·7)
Male	2856 (39·3)
Ethnicity, n (%)	
American Hispanic or Latino	529 (7·3)
Not American Hispanic or Latino	6744 (92·7)
Geographic ancestry, n (%)	
African heritage or African American	62 (0·9)
American Indian or Alaskan Native	6 (0·1)
Central or South Asian heritage	5 (0·1)
East Asian heritage	1083 (14·9)
Japanese heritage	261 (3·6)
South East Asian heritage	5 (0·1)
Native Hawaiian or other Pacific Islander	2 (0·0)
White (Arabic or North African heritage)	33 (0·5)
White (Caucasian or European heritage)	5530 (76·0)
Other	286 (3·9)
Region	
Australasia	1380 (19·0)
Europe	4680 (64·3)
Latin America	505 (6·9)
North America	708 (9·7)
Selected medical conditions, n (%)	
Hypertension	4211 (57·9)
Osteoarthritis or vertebral disorders	3450 (47·4)
Dyslipidaemia	2431 (33·4)
Diabetes	1299 (17·9)
Osteoporosis/osteopenia	811 (11·2)
Hypothyroidism	744 (10·2)
Coronary heart disease	724 (10·0)
Prostatic diseases	667 (9·2)
Sleep disorder	654 (9·0)
Gastroesophageal reflux disease	600 (8·2)
Depression	451 (6·2)
Asthma	366 (5·0)
Respiratory disorders	331 (4·6)
Cataract	307 (4·2)
Renal disorders	242 (3·3)
Number of comorbidities from above list of selected medical conditions, n (%)	
0	1126 (15·5)
1	1511 (20·8)
2	1605 (22·1)
3	1305 (17·9)
≥3	3031 (41·7)
≥4	1726 (23·7)

LTFU, long-term follow-up; mTVC, modified total vaccinated cohort; SD, standard deviation.

### Long-term vaccine efficacy against HZ (mTVC)

During the ZOE-LTFU study (period ranging from a median of 5·6 years to 11·4 years after the primary vaccination), 69 participants experienced a confirmed HZ episode. Overall VE against HZ during this period in participants ≥50 years of age at vaccination (primary endpoint) was 79·8% (95% CI: 73·7, 84·6) (Table 2). VE against HZ was higher among younger versus older participants (86·7%, 87·1%, and 73·2% in participants 50–59, 60–69, and ≥70 years of age at vaccination, respectively) (Table 2). VE was similar in women and men, with minor differences across regions (Table 2).

**Table 2:** Vaccine efficacy against HZ during the ZOE-LTFU study overall and by age strata, sex, region, and number of comorbidities (mTVC).

	RZV^a^				Historical control^b^				VE, % (95% CI)
	N	n	Sum of follow-up, years	Incidence per 1000 person-years	N	n	Sum of follow-up, years	Incidence per 1000 person-years	
Age at first vaccination									
Overall (≥50 years)	7258	69	39,109·3	1·8	7258	341	39,109·3	8·7	79·8 (73·7, 84·6)
50–59 years	2043	12	11,739·3	1·0	2043	90	11,739·3	7·7	86·7 (75·6, 93·4)
60–69 years	1242	9	6957·4	1·3	1242	70	6957·4	10·1	87·1 (74·2, 94·4)
70–79 years	3349	41	17,648·0	2·3	3349	146	17,648·0	8·3	71·9 (60·1, 80·6)
≥70 years	3973	48	20,412·6	2·4	3973	179	20,412·6	8·8	73·2 (62·9, 80·9)
≥80 years	624	7	2764·6	2·5	624	29	2764·6	10·5	75·9 (43·7, 91·1)
Sex									
Female	4404	39	24,023·2	1·6	4404	216	24,023·2	9·0	81·9 (74·5, 87·5)
Male	2854	30	15,086·1	2·0	2854	126	15,086·1	8·4	76·2 (64·3, 84·6)
Region									
Australasia	1377	17	7573·9	2·2	1377	101	7573·9	13·3	83·2 (71·7, 90·6)
Europe	4671	33	25,261·9	1·3	4671	176	25,261·9	7·0	81·3 (72·7, 87·5)
Latin America	503	9	2575·9	3·5	503	27	2575·9	10·5	66·7 (27·0, 86·2)
North America	707	10	3697·6	2·7	707	37	3697·6	10·0	73·0 (44·6, 88·0)
Number of comorbidities^c^									
0	1125	8	6319·5	1·3	NC	NC	NC	NC	NC
1	1511	10	8315·0	1·2	NC	NC	NC	NC	NC
2	1602	16	8672·0	1·9	NC	NC	NC	NC	NC
3	1300	19	6940·1	2·7	NC	NC	NC	NC	NC
≥3	3020	35	15,802·8	2·2	NC	NC	NC	NC	NC
≥4	1720	16	8862·7	1·8	NC	NC	NC	NC	NC

The incidence rate of HZ and VE against HZ during the ZOE-LTFU study were calculated in 7258 participants: 15 participants were not considered in the analyses because they had experienced HZ during ZOE-50/70.

Age strata are based on age at first vaccination in ZOE-50/70.

CI, confidence interval; HZ, herpes zoster; LTFU, long term follow-up; mTVC, modified total vaccinated cohort; N, number of participants in each group; n, number of participants with event; NC, not calculated; RZV, recombinant zoster vaccine; VE, vaccine efficacy.

a Data from participants in the LTFU and control groups of the ZOE-LTFU study.

b Data from the placebo group of ZOE-50/70 were used to form the historical control. The number of participants (N) and follow-up period in the historical control were assumed to be the same as the vaccinated group. The n for historical controls represents the projected number of confirmed HZ episodes based on the estimated incidence rates using placebo group data of ZOE-50/70.

c From the list of selected medical conditions shown in Table 1. Calculation of the incidence rate was a post-hoc analysis.

A post-hoc analysis showed that HZ incidence remained low in participants with comorbid conditions during this period (range 1·2 per 1000 in participants with one comorbidity to 2·7 per 1000 in those with three comorbidities) and similar to the overall study population (Table 2).

**Fig. 3:**
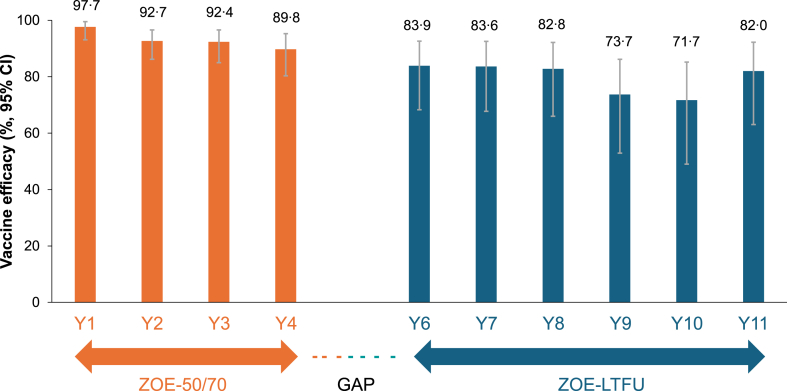
**Vaccine efficacy against HZ by year in ZOE-50/70 to the end of the ZOE-LTFU study (mTVC)**. Assessed in the LTFU and control groups. HZ, herpes zoster; LTFU, long-term follow-up; mTVC, modified total vaccinated cohort.

Over the entire post-vaccination period from 1 month post dose 2 in ZOE-50/70 to the end of ZOE-LTFU, VE against HZ was 87·7% (95% CI: 84·9, 90·1) overall (Table 3) and 82·0% (95% CI: 63·0, 92·2) in the eleventh year post-vaccination (Fig. 3; Appendix p11). The sensitivity analysis including HZ cases identified during the gap period showed similar results (Appendix p15).

**Table 3:** Vaccine efficacy against HZ from 1 month after the second vaccine dose in ZOE-50/70 to the end of the ZOE-LTFU study overall and by age strata, sex, and region (mTVC).

	RZV^a^				Placebo (ZOE-50/70) or historical control^b^				VE, % (95% CI)
	N	n	Sum of follow-up, years	Incidence per 1000 person-years	N	n	Sum of follow-up, years	Incidence per 1000 person-years	
Age at first vaccination									
Overall (≥50 years)	13,881	101	92,232·2	1·1	14,035	818	91,736·5	8·9	87·7 (84·9, 90·1)
50–59 years	3491	16	25,519·3	0·6	3523	193	25,453·3	7·6	91·7 (86·3, 95·4)
60–69 years	2140	12	15,574·7	0·8	2166	160	15,455·9	10·4	92·6 (86·7, 96·2)
70–79 years	6468	60	42,058·9	1·4	6554	362	41,910·8	8·6	83·5 (78·3, 87·7)
≥70 years	8250	73	51,138·1	1·4	8346	463	50,827·3	9·1	84·3 (79·9, 87·9)
≥80 years	1782	13	9079·2	1·4	1792	97	8916·5	10·9	86·8 (76·4, 93·2)
Sex									
Female	8044	58	55,000·1	1·1	8178	504	54,907·1	9·2	88·5 (84·9, 91·4)
Male	5837	43	37,232·1	1·2	5857	315	36,829·4	8·6	86·5 (81·4, 90·4)
Region									
Australasia	2766	23	18,480·9	1·2	2814	244	18,317·0	13·3	90·7 (85·7, 94·2)
Europe	7352	47	53,775·3	0·9	7432	373	53,579·5	7·0	87·5 (83·0, 90·9)
Latin America	1194	13	6800·2	1·9	1217	72	6848·1	10·5	81·8 (66·9, 90·8)
North America	2569	18	13,175·8	1·4	2572	129	12,991·9	9·9	86·2 (77·4, 92·1)

Age strata are based on age at first vaccination in the parent studies.

CI, confidence interval; HZ, herpes zoster; LTFU, long term follow-up; mTVC, modified total vaccinated cohort; N, number of participants in each group; n, number of participants with event; RZV, recombinant zoster vaccine; VE, vaccine efficacy.

a Data from participants in the RZV group of ZOE-50/70 were used for Year 1–Year 4; data from participants in the LTFU and control groups of the ZOE-LTFU study were used for Year 6 onwards.

b Data from participants in the placebo group of ZOE-50/70 were used for Year 1–Year 4; a historical control formed with data from participants in the placebo group of ZOE-50/70 was used for Year 6 onwards.

### Long-term vaccine efficacy against PHN and non-PHN complications (mTVC)

Four cases of PHN in participants ≥60 years at vaccination and one case of a HZ-related complication other than PHN (disseminated HZ) in those 70–79 years of age at vaccination were reported during ZOE-LTFU (Appendix p16). There were no cases of HZ complications in participants 50–59 years of age at vaccination. Overall VE estimates during ZOE-LTFU against PHN and HZ-related complications other than PHN were 87·5% (95% CI: 64·8, 96·8) and 91·7% (95% CI: 43·7, 99·8), respectively (Table 4). Over the entire post-vaccination period from 1 month post dose 2 in ZOE-50/70 to the end of ZOE-LTFU, VE was 89·7% (95% CI: 78·7, 95·7) against PHN and 92·8% (95% CI: 71·6, 99·2) against HZ-related complications other than PHN (Appendix p17–18).

**Table 4:** Vaccine efficacy against PHN and HZ-related complications other than PHN during the ZOE-LTFU study overall and by age strata (mTVC).

	RZV^a^				Historical control (placebo group in ZOE-50/70)^b^				VE, % (95% CI)
	N	n	Sum of follow-up, years	Incidence per 1000 person-years	N	n	Sum of follow-up, years	Incidence per 1000 person-years	
**PHN**									
Overall (≥50 years)	7271	4	39,347·3	0·1	7271	32	39,347·3	0·8	87·5 (64·8, 96·8)
50–59 years	2046	0	11,795·6	0·0	2046	7	11,795·6	0·6	100·0 (46·6, 100·0)
60–69 years	1243	1	6986·1	0·1	1243	2	6986·1	0·3	50·0 (−860·5, 99·2)
70–79 years	3357	2	17,773·1	0·1	3357	20	17,773·1	1·1	90·0 (58·8, 98·9)
≥70 years	3982	3	20,565·7	0·1	3982	23	20,565·7	1·1	87·0 (56·8, 97·5)
≥80 years	625	1	2792·6	0·4	625	2	2792·6	0·7	50·0 (−860·5, 99·2)
**HZ-related complications other than PHN**									
Overall (≥50 years)	7273	1	39,359·9	0·0	7273	12	39,359·9	0·3	91·7 (43·7, 99·8)
50–59 years	2046	0	11,795·6	0·0	2046	0	11,795·6	0·0	Undefined^c^
60–69 years	1243	0	6986·3	0·0	1243	2	6986·3	0·3	100·0 (−247·2, 100·0)
70–79 years	3359	1	17,780·8	0·1	3359	6	17,780·8	0·3	83·3 (−37·4, 99·6)
≥70 years	3984	1	20,578·1	0·0	3984	9	20,578·1	0·4	88·9 (19·8, 99·8)
≥80 years	625	0	2797·3	0·0	625	2	2797·3	0·7	100·0 (−247·2, 100·0)

VE against PHN was calculated in 7271 participants: 2 participants were not considered in the VE analysis because they had experienced PHN during ZOE-50/70.

Age strata are based on age at first vaccination in ZOE-50/70.

CI, confidence interval; HZ, herpes zoster; LTFU, long term follow-up; mTVC, modified total vaccinated cohort; N, number of participants in each group; n, number of participants with event; PHN, post-herpetic neuralgia; RZV, recombinant zoster vaccine; VE, vaccine efficacy.

a Data from participants in the LTFU and control groups of the ZOE-LTFU study.

b Data from the placebo group of ZOE-50/70 were used to form the historical control. The number of participants (N) and follow-up period in the historical control were assumed to be the same as the vaccinated group. The n for historical controls represents the projected number of confirmed PHN episodes or non-PHN complications based on the estimated incidence rates using placebo group data of ZOE-50/70.

c VE is mathematically undefined when zero cases were observed in the historical control group.

### Pain experienced during HZ episodes (mTVC)

Of the 69 participants who experienced a confirmed HZ episode, 61 completed a ZBPI evaluation, of whom 58 (95·1%) reported pain (Worst Pain Score ≥1), 52 (85·2%) reported clinically significant pain (Worst Pain Score ≥3), and 38 (62·3%) reported severe pain (Worst Pain Score ≥7). The mean Worst Pain Score on the ZBPI was 6·7 (standard deviation [SD] 3·0). Clinically significant pain resolved after a median of 19 days.

Most participants (85·2%) received pain medication for the HZ episode. The median duration of pain medication receipt was 34·5 days (interquartile range [IQR]: 14·0, 73·0), with a trend for longer duration of use with increasing age: 24·0 days (IQR: 19·0, 42·0) in participants 50–59 years of age; 43·0 days (IQR: 18·0, 64·5) for 60–69 years; 38·0 days (IQR: 9·0, 118·0) for 70–79 years; and 82·5 days (IQR: 34·5, 358·5) for ≥80 years.

### Persistence of the immune response (adapted ATP cohort)

**Fig. 4:**
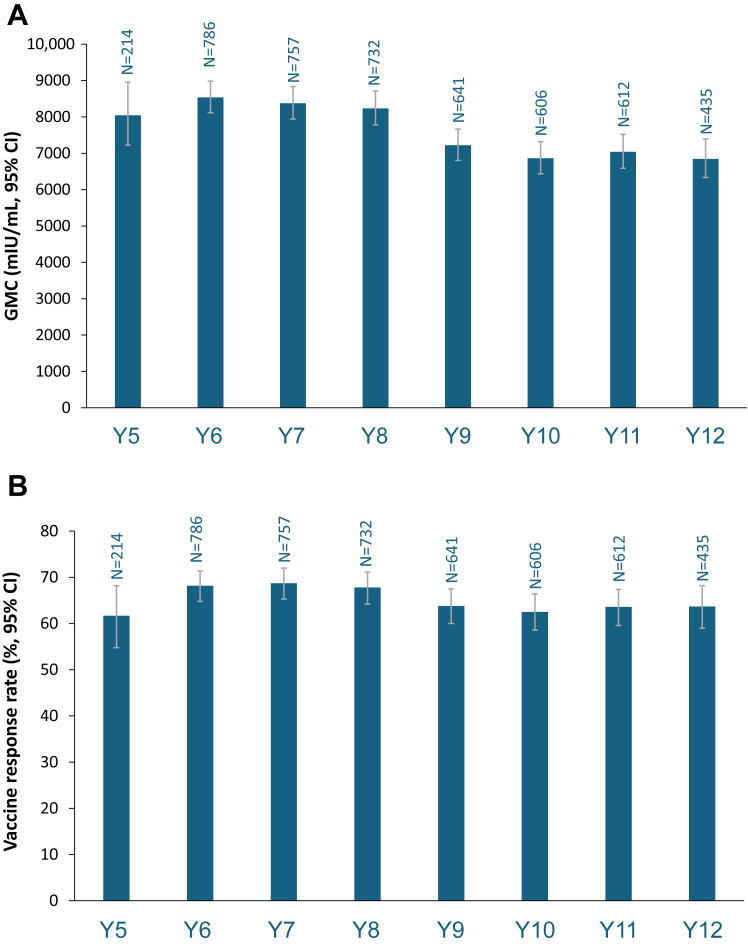

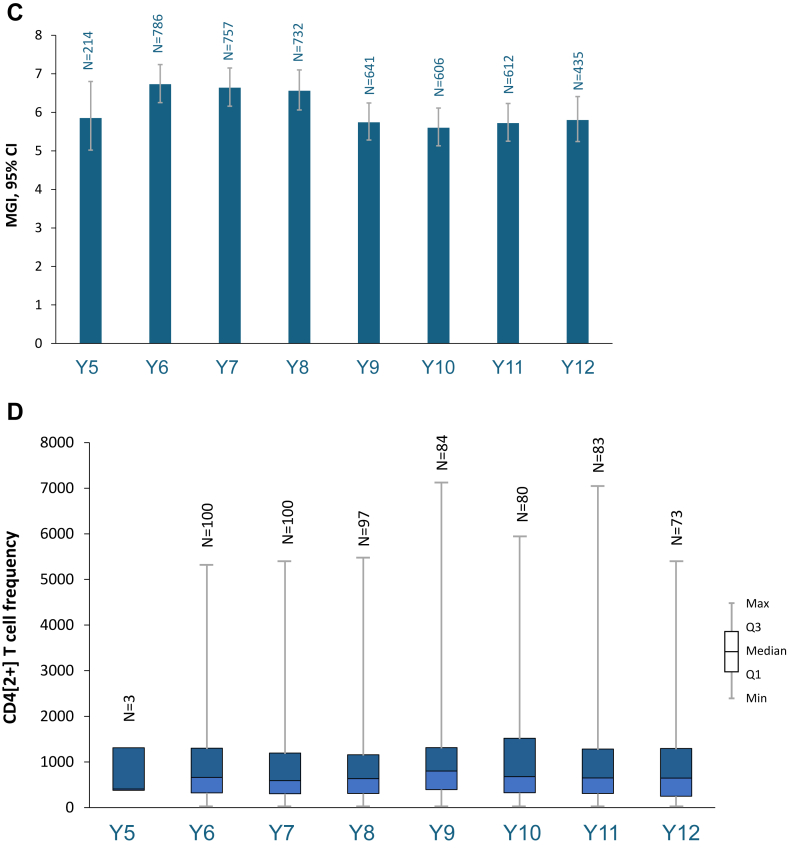
**Immunogenicity assessments (adapted ATP cohort)**. A. Geometric mean concentration of anti-gE antibody. B. Vaccine response rates for anti-gE antibody. C. Mean geometric increase of anti-gE antibody over pre-vaccination levels in the ZOE-50/70 studies. D. Frequency of gE-specific CD4[2+] T cells. Assessed in the LTFU group. VRR was the percentage of participants with post-vaccination anti-gE antibody concentration ≥4-fold of the cut-off value for initially seronegative participants and ≥4-fold of the pre-vaccination value for initially seropositive participants. MGI was the ratio of the GMC post-vaccination/pre-vaccination. ATP, according to protocol; CI, confidence interval; GMC, geometric mean concentration; max; maximum; MGI, mean geometric increase; min, minimum; N, number of participants evaluated; Q1, first quartile; Q3, third quartile.

During ZOE-LTFU, GMCs of anti-gE Ab were stable from Year 5 to Year 8, with a modest decrease at Year 9, and remained overall stable through Year 12 (Fig. 4, Appendix p19–20). GMCs of anti-gE antibody were 8536·6 mIU/mL at Year 6 and 6844·3 mIU/mL at Year 12. VRRs were stable throughout the study and MGI (ratio of antibody concentration post-vaccination versus pre-vaccination) was sustained at 5·9-fold at Year 5–5·8-fold at Year 12 (Fig. 4). Median CD4[2+] T cell frequency was 407 at Year 5 and 644 at Year 12 (Fig. 4).

Analysis of antibody persistence by age strata showed similar results among the 50–59, 60–69, and ≥60 years age strata compared with the overall populations. GMCs, VRR, and MGI of anti-gE antibodies were lower in the ≥70 years stratum than other age strata (Appendix p19–24). For the analysis by age strata performed for CMI persistence, the number of participants were too small for any meaningful interpretation (Appendix p25–26).

### Safety (TVC)

In the LTFU and control groups, there were no deaths or SAEs, or AEs or SAEs leading to withdrawal that were considered related to the investigational vaccine, study participation or to a concurrent GSK medication or vaccine during the entire ZOE-LTFU study. The most frequent withdrawals from the study in these groups were due to unrelated SAEs in 853 and 9 participants, respectively, consent withdrawal not due to an AE (360 and 10 participants, respectively), and loss to follow-up (198 participants in LTFU group only).

## Discussion

The ageing population continues to increase in most parts of the world.^19^ HZ primarily occurs in adults 50 years of age and older and the risk of complications rises with age.^3^^,^^20^ Although the incidence of HZ rises with age, some studies have shown that the highest number of cases occurs in people 50–59 years of age.^21^^,^^22^ Therefore, it is important to evaluate the duration of RZV efficacy to ensure that people are adequately protected as they age. These long-term data can also guide HCPs in decision-making regarding vaccination for HZ prevention.

The ZOE-LTFU study, an extension of the two pivotal phase 3 trials (ZOE-50/70), was designed to evaluate the long-term efficacy of RZV and followed participants from a median of 5·6 years to 11·4 years after primary vaccination in the parent studies. The population of ZOE-LTFU was generally representative of the ZOE-50/70 population. In the study population of ZOE-LTFU, the mean age at first vaccination was 67·3 years and 68·5 years in the ZOE-50/70 population^23^ and the proportion of women was 61% in the ZOE-LTFU population and 58% in the ZOE-50/70 population^23^ (mTVC cohorts). The proportion of participants with ≥3 comorbidities at study enrolment was 42% in ZOE-LTFU and 37% in ZOE-50/70 (mTVC)^23^; the higher proportion in ZOE-LTFU was to be expected, as the participants had aged between enrolment into ZOE-50/70 and enrolment into ZOE-LTFU.

Our study showed that RZV provides a long duration of protection. Overall VE against HZ in adults ≥50 years of age during ZOE-LTFU (the primary endpoint) was 79·8%. Over the entire post-vaccination period in ZOE-50/70 and ZOE-LTFU, overall VE against HZ was 87·7%. Yearly estimates of VE were sustained at an overall high level during long-term follow-up: VE was stable between 83⋅9% and 82·8% in Years 6–8, decreased at 73·7% during Year 9 and 71·7% during Year 10, and remained high at 82·0% during Year 11. The reason for the observed decline in VE during Years 9 and 10 is not clear but might have been related to the COVID-19 pandemic. Some participants of ZOE-LTFU are enrolled in an ongoing extension study (NCT05371080) which might provide further insights.

ZOE-LTFU demonstrated that RZV offered durable protection across a broad population. Efficacy was consistent in men and women. VE against HZ was higher in age strata vaccinated at a younger age (86·7% in participants 50–59 years and 87·1% in participants 60–69 years) than in older age groups (73·2% in participants ≥70 years). As expected among people ≥50 years of age, most had comorbidities (84·5% had ≥1 comorbidity and 41·7% had ≥3 comorbidities). The most common comorbidities were hypertension, osteoarthritis and/or vertebral disorders, and dyslipidaemia. Although participants who were immunodeficient or immunosuppressed due to disease or therapy were excluded from study entry, those who became immunocompromised or immunosuppressed during the ZOE-LTFU study were included in the analysis of efficacy. Individuals with comorbidities can be at greater risk of infectious diseases and it is important that they are included in vaccine trials. Reassuringly, HZ incidence in ZOE-LTFU remained low and similar to the overall study population, indicating that RZV continues to offer protection against HZ in individuals with multiple comorbidities. A previous analysis of ZOE-50/70 has also shown that VE is maintained in individuals with selected medical conditions, regardless of the number of conditions present.^23^

HZ complications have a substantial impact on quality of life^24^ and the economic burden of disease.^25^ The incidence of HZ-related complications rises with age,^3^^,^^20^ and protecting against such complications is an important role of vaccination. Efficacy of RZV against PHN remained high during ZOE-LTFU at 87·5% compared with 91·2% in ZOE-50/70.^11^ Efficacy of RZV against complications other than PHN was 91·7% in ZOE-LTFU. All complications occurred in individuals >75 years of age at the onset of the HZ episode.

In ZOE-50/70, anti-gE antibody GMCs were 1321 mIU/mL and 17,297 mIU/mL at pre-vaccination and Year 1, respectively.^14^^,^^26^ At 3 years post-vaccination, GMCs remained 8-fold above pre-vaccination levels.^26^ In ZOE-LTFU, the humoural immune response reached a plateau at over 5-fold above pre-vaccination levels. Median CD4[2+] T cell frequencies in ZOE-50/70 were 90 and 800 at pre-vaccination and Year 1, respectively, and remained at approximately 8-fold above pre-vaccination levels at 3 years.^14^^,^^26^ Median CD4[2+] T cell frequencies remained broadly stable during ZOE-LTFU at approximately 7-fold pre-vaccination levels. The cell-mediated immune response is thought to be a better indicator of immunogenicity for HZ; however, it was measured in a smaller number of participants than the humoural response in ZOE-LTFU. An extension of a phase 2 study of RZV in adults vaccinated at ≥60 years of age showed sustained immune responses up to 10 years after vaccination, with humoural and cell-mediated responses approximately 6-fold and 3·5-fold, respectively, above pre-vaccination levels.^27^ Overall, ZOE-LTFU provides actual long-term immunogenicity data consistent with previous studies and supporting the hypothesis of persistent high efficacy.

There were no deaths or SAEs, or AEs or SAEs leading to withdrawal that were considered related to the investigational vaccine, study participation or to a concurrent GSK medication or vaccine during the entire ZOE-LTFU study. The most common reasons for study withdrawal were unrelated SAEs, consent withdrawal not related to an AE, and loss to follow-up. The study adds to the long-term safety profile of RZV which has been established across multiple clinical trials and during over 5 years of post-licensure use.

The efficacy of RZV in the ZOE-50/70 studies and the potential public health benefits have contributed to changes in the national immunisation programmes of several countries. Some countries have introduced preferential recommendations in favour of RZV, some that did not previously recommend HZ vaccination have included RZV in their routine immunisation programmes, and some have lowered the recommended age of vaccination.^28^, ^29^, ^30^, ^31^, ^32^ Data from the ZOE-LTFU study, including high efficacy observed in all age groups and minimal waning of VE over time, might support further introductions and expansion of vaccination to all age groups ≥50 years.

Our study had some limitations. Only approximately one half of potentially eligible participants from ZOE-50/70 took part in the ZOE-LTFU study, although the participants in ZOE-LTFU were generally representative of those in the parent studies. Due to the high VE and favourable safety profile of RZV in ZOE-50/70, participants from the placebo group were offered the vaccine in a cross-vaccination study. Therefore, there was no placebo group in the ZOE-LTFU study, meaning that a historical control had to be used for assessment of VE. However, this is an acceptable methodology in these circumstances. A potential weakness of the historical control is that it did not capture any possible impact of the COVID-19 pandemic; furthermore, the incidence of HZ is known to fluctuate over time.^33^ However, VE estimates could be considered conservative, as the HZ incidence rate in the historical controls does not account for population ageing or severity of comorbidities. A further weakness is that no data were collected during the gap period between the end of ZOE-50/70 and the start of ZOE-LTFU; however, a sensitivity analysis including data from this period showed similar results to the main analysis. The major strength of our study lies in its long duration and broad population representative of the age groups recommended for vaccination.

This large trial of more than 7000 participants demonstrated that RZV offers durable protection against HZ and associated complications through 11 years post-vaccination. Minimal waning of VE against HZ was observed and VE against HZ complications including PHN was sustained at similar levels to the original studies. VE was high and maintained throughout the study with a minimal impact of age at first vaccination. Both humoural and cell-mediated immune responses remained above pre-vaccination levels throughout the study and the safety profile remained clinically acceptable. In conclusion, the vaccine offers long-term protection across a broad population, including those vaccinated at ≥70 years of age and those with multiple comorbidities.

## Contributors

Conceptualisation: Ana Strezova, Anthony L Cunningham, Chong-Jen Yu. Data curation: Jing Wang, Jyoti Soni. Formal analysis: Jing Wang, Jyoti Soni. Investigation: Javier Díez Domingo, Anthony L Cunningham, Takashi Eto, Lars Rombo, Charles Andrews, Clovis Arns, Eun-Ju Choo, David Shu Cheong Hui, Giancarlo Icardi, Shelly A McNeil, Airi Põder, Pavel Kosina, Tino F Schwarz, Juan Carlos Tinoco, Chong-Jen Yu. Methodology: Ana Strezova, Jing Wang, Jyoti Soni, Manyee Tsang, Rafael Leon, Agnes Mwakingwe-Omari. Project administration: All authors. Resources: All authors. Supervision: Ana Strezova, Jing Wang, Agnes Mwakingwe-Omari. Validation: Ana Strezova, Jing Wang, Jyoti Soni, Manyee Tsang, Rafael Leon, Agnes Mwakingwe-Omari, Javier Diez-Domingo, Juan Carlos Tinoco, Chong-Jen Yu. Visualisation: Ana Strezova, Jing Wang, Agnes Mwakingwe-Omari. Writing of the original draft: Ana Strezova, Agnes Mwakingwe-Omari. Review and editing of draft: All authors.

Ana Strezova and Jing Wang verified the underlying data in the study. All authors approved the final version and had final responsibility for the decision to submit for publication. All authors had full access to all the data.

## Data sharing statement

Please refer to GSK weblink to access GSK's data sharing policies and as applicable seek anonymised subject level data via the link https://www.gsk-studyregister.com/en/.

## Declaration of interests

Ana Strezova is employed by GSK and holds financial equities in GSK. Javier Díez Domingo reports payments to his institution from GSK, Sanofi, MSD, Moderna, and AZ sponsored studies. Javier Díez Domingo also reports payments from GSK, Sanofi Pasteur, Pfizer, and Moderna, as well as support for attending meetings and/or travel from GSK and Pfizer. Anthony L Cunningham reports payments to his institution from GSK, Moderna, Seqirus, Abbvie, and Healthed. Anthony L Cunningham also reports support for attending meetings and/or travel from GSK and Moderna. Takashi Eto declares no financial and non-financial relationships and activities and no conflicts of interest. Charles Andrews reports consulting fees from Boehringer Ingelheim, Merck, and Bayer, as well as participation on boards for Moderna. Clovis Arns reports honoraria for lectures and presentations, and payments for attending meetings and/or travel from GSK. Eun-Ju Choo declares no financial and non-financial relationships and activities and no conflicts of interest. David Shu Cheong Hui declares no financial and non-financial relationships and activities and no conflicts of interest. Giancarlo Icardi declares no financial and non-financial relationships and activities and no conflicts of interest. Shelly A McNeil reports payments to her institution from GSK and consulting fees or participation on advisory boards and a DSMB from GSK. Airi Põder declares no financial and non-financial relationships and activities and no conflicts of interest. Pavel Kosina declares no financial and non-financial relationships and activities and no conflicts of interest. Lars Rombo declares no financial and non-financial relationships and activities and no conflicts of interest. Tino F Schwarz reports payments from AstraZeneca, Bavarian Nordic, Biogen, Biontech, CRM, CSL Seqirus, CSL Vifor, GSK, Janssen-Cilag, Merck-Serono, Moderna, Novavax, MSD, Pfizer, Roche, Sanofi-Aventis, Synlab, Takeda. Tino F Schwarz also reports participantions on boards for Bavarian Nordic, Biontech, CSL Seqirus, GSK, Moderna, Novavax, Takeda. Juan Carlos Tinoco reports payments for lectures and speakers bureaus from GSK. Chong-Jen Yu declares no financial and non-financial relationships and activities and no conflicts of interest. Jing Wang is employed by GSK. Jyoti Soni is employed by GSK and holds financial equities in GSK. Manyee Tsang is employed by GSK and holds financial equities in GSK. Rafael Leon is employed by GSK and holds financial equities in GSK. Agnes Mwakingwe-Omari is employed by GSK and holds financial equities in GSK. The authors declare no other financial and non-financial relationships and activities.

## Appendix ASupplementary data

Protocol Amendment 5
