# Supplementary material for: Final analysis of the ZOE-LTFU trial to 11 years post-vaccination: efficacy of the adjuvanted recombinant zoster vaccine against herpes zoster and related complications
Source: eClinicalMedicine. 2025 May 9;83:103241. doi: 10.1016/j.eclinm.2025.103241 (PMC12235393; doi:10.1016/j.eclinm.2025.103241)
Supplement: Protocol Amendment 5 [file mmc1.pdf]

Protocol Amendment 5

Study ID: 201190

Official Title of Study: A phase IIIb, open-label, multi-country, multi-centre, long-term follow-up study (ZOE-LTFU) of studies 110390 and 113077 (ZOSTER-006/022) to assess the prophylactic efficacy, safety, and immunogenicity persistence of GSK Biologicalsâ€™ Herpes Zoster subunit (HZ/su) vaccine and assessment of 1 or 2 additional doses on a 0 or 0, 2-month schedule in two subgroups of older adults.

NCT number: NCT02723773

Date of Document: 11 May 2020

Clinical Study Protocol
Sponsor:
GlaxoSmithKline Biologicals
Rue de l'Institut 89, 1330 Rixensart, Belgium

**Primary Study vaccine (and number)**	GlaxoSmithKline (GSK) Biologicals' lyophilised formulation of the Herpes Zoster subunit (HZ/su) vaccine (GSK1437173A)
**eTrack study number and Abbreviated Title**	201190 (ZOSTER-049 EXT:006-022)
**Investigational New Drug (IND) number**	BB-IND 13857
**EudraCT number**	2015-001778-17
**Date of protocol**	Final Version 1: 11 December 2015
**Date of protocol amendment/adminis-trative change**	Amendment 1 Final: 19 February 2016<br>Amendment 2 Final: 19 January 2017<br>Administrative Change 1 Final: 05 May 2017<br>Amendment 3 Final: 16 March 2018<br>Amendment 4 Final: 23 October 2018<br>Administrative Change 2 Final: 11 February 2019<br>Amendment 5 Final: 11 May 2020
**Title**	Efficacy, safety and immunogenicity of GSK Biologicals' HZ/su vaccine GSK1437173A in a phase IIIb, open-label, long-term follow-up study (ZOE-LTFU) of studies 110390/113077 (ZOSTER-006/022) and assessment of additional doses in older adults
**Detailed Title**	A phase IIIb, open-label, multi-country, multi-centre, long-term follow-up study (ZOE-LTFU) of studies 110390 and 113077 (ZOSTER-006/022) to assess the prophylactic efficacy, safety, and immunogenicity persistence of GSK Biologicals' Herpes Zoster subunit (HZ/su) vaccine and assessment of 1 or 2 additional doses on a 0 or 0, 2-month schedule in two subgroups of older adults.
**Co-ordinating author (Amended 11 May 2020)**	PPD, PPD ***and*** PPD, ***Scientific Writer***
**Contributing authors (Amended 11 May 2020)**	• PPD, PPD ***and*** PPD Clinical Research and Development Leads<br>• PPD, PPD, PPD, Study Delivery Leads

**eTrack study number and Abbreviated Title**	201190 (ZOSTER-049 EXT:006-022)
**Investigational New Drug (IND) number**	BB-IND 13857
**EudraCT number**	2015-001778-17
**Detailed Title**	A phase IIIb, open-label, multi-country, multi-centre, long-term follow-up study (ZOE-LTFU) of studies 110390 and 113077 (ZOSTER-006/022) to assess the prophylactic efficacy, safety, and immunogenicity persistence of GSK Biologicals' Herpes Zoster subunit (HZ/su) vaccine and assessment of 1 or 2 additional doses on a 0 or 0, 2-month schedule in two subgroups of older adults.

Contributing authors (continued) (Amended 11 May 2020)

- PPD and PPD, Clinical Laboratory Sciences Clinical readout Team Leader
- PPD and PPD, Clinical Laboratory Sciences Study Manager
- PPD, Vaccine Supply Coordinator
- PPD, PPD and PPD PPD, Clinical Safety representatives
- PPD, Clinical Safety Physician
- PPD ***and*** PPD, Oversight Data Managers
- PPD, PPD and PPD PPD (USA), Global Regulatory Affairs
- PPD***, Local delivery Lead***
- PPD, Global Patent
- PPD and PPD, Lead Statisticians
- PPD***, Project Statistician***
- PPD, Project Statistician
- PPD, Clinical and Epidemiology Project Lead for Zoster, GlaxoSmithKline Biologicals, US RDC

GSK Biologicals' Protocol DS v14.1.1

©2015 GSK group of companies or its licensor

Protocol Amendment 5 Sponsor Signatory Approval

**eTrack study number and Abbreviated Title**	201190 (ZOSTER-049 EXT:006-022)
**IND number**	BB-IND 13857
**EudraCT number**	2015-001778-17
**Date of protocol amendment**	Amendment 5 Final: 11 May 2020
**Detailed Title**	A phase IIIb, open-label, multi-country, multi-centre, long-term follow-up study (ZOE-LTFU) of studies 110390 and 113077 (ZOSTER-006/022) to assess the prophylactic efficacy, safety, and immunogenicity persistence of GSK Biologicals' Herpes Zoster subunit (HZ/su) vaccine and assessment of 1 or 2 additional doses on a 0 or 0, 2-month schedule in two subgroups of older adults.
**Sponsor signatory**	Anne Schuind, Clinical and Epidemiology Project Lead for Zoster, GlaxoSmithKline Biologicals, US RDC

Signature ____________________________________

Date ____________________________________

Protocol Amendment 5 Rationale

**Amendment number**	5

Rationale/background for changes:

This protocol amendment 5 outlines measures that may be applicable during special circumstances (e.g., during COVID-19 pandemic). The purpose of the amendment is to introduce measures that may allow protection of subject's welfare and safety, as well as maintaining the integrity of the study.

This amendment is considered substantial based on the criteria defined in Article 10(a) of Directive 2001/20/EC of the European Parliament and the Council of the European Union because it significantly impacts the safety of subjects or/nor the scientific value of the study.

As much as possible all study specified visits and procedures should be completed according to the protocol, taking into account clinical judgment and local public health guidance to protect the safety of staff and subjects.

Section 6.6 outlines the measures which include allowing flexibility in schedule and procedures to optimize site staff safety, patient safety and to preserve study integrity.

Other changes include,

- The classification of HZ cases by HZAC has been clarified with the addition of "not able to decide" to be classified as "not HZ" to Section 4.5.2.4.2 for confirmation of suspected HZ by the HZAC.
- The timelines concerning data entry into the eCRF for France in Section 13.1 have been updated to align with the timelines presented in the data management plan.

Please refer to Appendix C for a list of all the changes.

Protocol Amendment 5 Investigator Agreement

I agree:

- To conduct the study in compliance with this protocol, any future protocol amendments or protocol administrative changes, with the terms of the clinical trial agreement and with any other study conduct procedures and/or study conduct documents provided by GlaxoSmithKline (GSK) Biologicals.
- To assume responsibility for the proper conduct of the study at this site.
- That I am aware of, and will comply with, 'Good Clinical Practice' (GCP) and all applicable regulatory requirements.
- To ensure that all persons assisting me with the study are adequately informed about the GSK Biologicals' investigational vaccines and other study-related duties and functions as described in the protocol.
- To acquire the reference ranges for laboratory tests performed locally and, if required by local regulations, obtain the laboratory's current certification or Quality Assurance procedure manual.
- To ensure that no clinical samples (including serum samples) are retained onsite or elsewhere without the approval of GSK Biologicals and the express written informed consent of the subject and/or the subject's legally acceptable representative.
- To perform no other biological assays on the clinical samples except those described in the protocol or its amendment(s).
- To co-operate with a representative of GSK Biologicals in the monitoring process of the study and in resolution of queries about the data.
- That I have been informed that certain regulatory authorities require the sponsor to obtain and supply, as necessary, details about the investigator's ownership interest in the sponsor or the investigational vaccines, and more generally about his/her financial ties with the sponsor. GSK Biologicals will use and disclose the information solely for the purpose of complying with regulatory requirements.

Hence, I:

- Agree to supply GSK Biologicals with any necessary information regarding ownership interest and financial ties (including those of my spouse and dependent children).
- Agree to promptly update this information if any relevant changes occur during the course of the study and for one year following completion of the study.
- Agree that GSK Biologicals may disclose any information it has about such ownership interests and financial ties to regulatory authorities.
- Agree to provide GSK Biologicals with an updated Curriculum Vitae and other documents required by regulatory agencies for this study.

**eTrack study number and Abbreviated Title**	201190 (ZOSTER-049 EXT:006-022)
**IND number**	BB-IND 13857
**EudraCT number**	2015-001778-17
**Date of protocol amendment**	Amendment 5 Final: 11 May 2020
**Detailed Title**	A phase IIIb, open-label, multi-country, multi-centre, long-term follow-up study (ZOE-LTFU) of studies 110390 and 113077 (ZOSTER-006/022) to assess the prophylactic efficacy, safety, and immunogenicity persistence of GSK Biologicals' Herpes Zoster subunit (HZ/su) vaccine and assessment of 1 or 2 additional doses on a 0 or 0, 2-month schedule in two subgroups of older adults.

Investigator name ____________________

Signature ____________________

Date ____________________

PPD
PPD **name, function and title** ____________________

Signature ____________________

Date ____________________

**eTrack study number and Abbreviated Title**	201190 (ZOSTER-049 EXT:006-022)
**IND number**	BB-IND 13857
**EudraCT number**	2015-001778-17
**Date of protocol amendment**	Amendment 5 Final: 11 May 2020
**Detailed Title**	A phase IIIb, open-label, multi-country, multi-centre, long-term follow-up study (ZOE-LTFU) of studies 110390 and 113077 (ZOSTER-006/022) to assess the prophylactic efficacy, safety, and immunogenicity persistence of GSK Biologicals' Herpes Zoster subunit (HZ/su) vaccine and assessment of 1 or 2 additional doses on a 0 or 0, 2-month schedule in two subgroups of older adults.
**GSK Japan representative name, function and title**	
**Signature**	______________________________
**Date**	______________________________

Sponsor Information

1. Sponsor

GlaxoSmithKline Biologicals
Rue de l'Institut 89, 1330 Rixensart, Belgium

2. Sponsor Medical Expert for the Study

Refer to the local study contact information document.

3. Sponsor Study Monitor

Refer to the local study contact information document.

4. Sponsor Study Contact for Reporting of a Serious Adverse Event

GSK Biologicals Central Back-up Study Contact for Reporting SAEs: refer to protocol Section 9.4.2.

SYNOPSIS

**Detailed Title**	A phase IIIb, open-label, multi-country, multi-centre, long-term follow-up study (ZOE-LTFU) of studies 110390 and 113077 (ZOSTER-006/022) to assess the prophylactic efficacy, safety, and immunogenicity persistence of GSK Biologicals' Herpes Zoster subunit (HZ/su) vaccine and assessment of 1 or 2 additional doses on a 0 or 0, 2-month schedule in two subgroups of older adults.
**Indication**	Prevention of Herpes Zoster (HZ) and related complications in adults aged 50 years and older and in immunocompromised (IC) adults aged 18 years and older.
**Rationale for the study and study design**	• Rationale for the study<br>GlaxoSmithKline (GSK) Biologicals' study vaccine for the prevention of HZ, is a recombinant subunit (su) vaccine consisting of Varicella Zoster Virus (VZV) glycoprotein E (gE) as antigen and an adjuvant system (AS01), has been and is being evaluated in several studies in older adults and immunocompromised adults. In these studies, it was shown to elicit strong cellular and humoral immune responses. Furthermore, the safety and reactogenicity profile of the study vaccine was clinically acceptable. Based on phase II data from the antigen dose-ranging study, ZOSTER-003, and the adjuvant dose comparison study, ZOSTER-010, a gE antigen dose of 50 µg and the adjuvant system $AS01_B$ were selected for the final vaccine formulation. Henceforth, the final vaccine formulation will be referred to as HZ/su. The results of the previous studies in older adults demonstrated strong vaccine-induced immune responses following HZ/su administration at 0 and 2 months, supporting the selection of a 2-dose vaccine schedule.<br>Two large pivotal phase III trials ZOE-50 and ZOE-70 that enrolled subjects ≥ 50 and ≥ 70 years of age (YOA) respectively, evaluated the vaccine efficacy (VE), immunogenicity and safety of GSK Biologicals' HZ/su vaccine. These trials, hereafter referred to as ZOSTER-006 and ZOSTER-022, respectively and collectively referred to as the ZOSTER-006/022 studies, enrolled more than 30,000 subjects who either received the HZ/su vaccine or placebo on a 0, 2-month schedule.<br>The final HZ/su VE results from the ZOSTER-006 phase III trial demonstrated the HZ/su vaccine to be highly efficacious in the prevention of HZ overall and in all age strata and available safety data until the data lock point raised no safety concerns in this population of subjects ≥ 50 YOA. The results for the ZOSTER-

022 study demonstrated that the HZ/su vaccine reduced the risks of HZ and PHN among adults ≥ 70 YOA.

Since the mean follow-up period for VE at the time of the final analysis in the ZOSTER-006/022 studies was about four years, further follow-up for long term efficacy assessment is needed, in order to establish that the HZ/su vaccine provides not only strong, but also persistent protection.

This study will also assess the immunogenicity responses of 1 or 2 additional doses in two subgroups of older adults who previously received two doses of the HZ/su vaccine in the ZOSTER-006/022 studies. If over time, VE of HZ/su wanes, additional vaccination with HZ/su could be needed to prevent HZ and its complications. Knowing if 1 or 2 additional doses of HZ/su vaccine can stimulate the immune response again, could support the concept of providing additional doses with the expectation that this may translate into preserving VE.

- Rationale for the study design

The primary objective of this study is to assess the efficacy of the HZ/su vaccine in the prevention of HZ. The data from this study will also be analysed combined with ZOSTER-006/022 data (analysis over the full duration of the follow-up in the primary ZOSTER-006/022 studies and the ZOSTER-049 study). Persistence of immunogenicity and long-term safety of the HZ/su vaccine administered in ZOSTER-006/022 will also be evaluated. Safety, reactogenicity, and immunogenicity one month after 1 or 2 additional doses of the HZ/su vaccine in subjects who previously received 2 doses of the vaccine in the primary ZOSTER-006/022 studies will also be assessed to further support evaluation of additional vaccination if it is determined VE wanes over time.

Due to the high VE observed in the ZOSTER-006/022 studies, it was considered that subjects having received placebo during both studies should be offered cross-vaccination with HZ/su vaccine as soon as possible. Since they will be enrolled in a separate cross-vaccination study, no placebo recipients will be available for this study and historic controls will be used for assessment of VE.

All subjects that were previously vaccinated with at least one dose of the HZ/su vaccine in the ZOSTER-006/022 primary studies, and confirmed their interest to enrol in the study at participating centres, will be considered for entry in study ZOSTER-049, after they have completed all study-related activities in the ZOSTER-006 or ZOSTER-022 studies and when their treatment assignment can be released without compromising the integrity of the ZOSTER-006/022 data. Since the interval

between the end of the ZOSTER-006/022 studies and the start of study ZOSTER-049 will vary per subject and is dependent on receipt of approval or implementing the study in the different participating countries/centres, the study will analyze the annual LTFU data from Year 5 up to Year 10 and annually beyond Year 10 after the primary vaccination in the ZOSTER-006/022 studies (in case samples from these timepoints are collected).

The study will be comprised of four study groups as follows:

LTFU Group: Subjects from this group [N ≤ 14,000] will be followed for VE and safety. In addition, immunogenicity will be followed in subjects that were part of the immunogenicity subset (HI) in the ZOSTER-006/022 studies and Cell-Mediated Immunity (CMI) subset from the ZOSTER-006 study;

1-Additional Dose Group: Subjects from this group [N = 60] will receive 1 additional dose of the HZ/su vaccine, at the time of enrolment, to assess the immunogenicity, reactogenicity and safety of 1 additional dose;

Revaccination Group: Subjects from this group [N = 60] will be revaccinated with 2 additional doses of the HZ/su vaccine, on a 0, 2-month schedule from the time of enrolment, to assess the immunogenicity, reactogenicity and safety of 2 additional doses;

Control Group: Subjects from this group [N = 120] will not be vaccinated but will serve as a control to assess immunogenicity and safety compared to the two vaccinated groups 1-Additional Dose and Revaccination. This Control group will also be used to evaluate the VE, safety and immunogenicity.

Subjects (N, to be determined) who developed HZ during the ZOSTER-006/022 studies (confirmed HZ) and/or during the interval between the end of the ZOSTER-006/022 studies and beginning of the ZOSTER-049 study, and/or during the ZOSTER-049 study (suspected HZ) will be part of the **HZ subset**.

Subjects from the 1-Additional Dose and Revaccination groups who will develop HZ cases during the ZOSTER-049 study will follow the sampling schedule as per their original group and will not be part of the HZ subset.

Note: Subjects from the immunogenicity subset of studies ZOSTER-006/022 are not allocated to the 1-Additional Dose, Revaccination and Control groups. Subjects in these three groups will be enrolled amongst subjects who received 2 doses of the HZ/su vaccine and were part of the ATP cohort for analysis of efficacy in the previous studies ZOSTER-006/022. In order not to confound immune responses, subjects who developed HZ prior to enrolment in the ZOSTER-049 study (confirmed HZ during the

ZOSTER-006/022 studies or suspected HZ during the interval between the end of the ZOSTER-006/022 studies and beginning of the ZOSTER-049 study), will not be enrolled in the 1-Additional Dose, Revaccination and Control groups.

Objectives

Primary

- To assess the VE in the prevention of HZ over the total duration of the ZOSTER-049 study as measured by the reduction in HZ risk in subjects ≥ 50 YOA overall at the time of first vaccination in the ZOSTER-006/022 studies.

Secondary

- To assess the VE in the prevention of HZ over the total duration of the ZOSTER-049 study as measured by the reduction in HZ risk in subjects within each of the age ranges* at the time of first vaccination in the ZOSTER-006/022 studies;
- To assess the VE in the prevention of HZ from one month post dose 2 in the ZOSTER-006/022 studies until the end of the ZOSTER-049 study as measured by the reduction in HZ risk in subjects ≥ 50 YOA overall and within each of the specified age ranges* at the time of first vaccination in the ZOSTER-006/022 studies;
- To assess the VE in the prevention of HZ over each year of follow-up from one month post dose 2 in the ZOSTER-006/022 studies as measured by the reduction in HZ risk in subjects ≥ 50 YOA overall and within each of the specified age ranges* at the time of first vaccination in the ZOSTER-006/022 studies;
- To assess the VE over the total duration of the ZOSTER-049 study in prevention of PHN in subjects ≥ 50 YOA overall and within each of the specified age ranges* at the time of first vaccination in the ZOSTER-006/022 studies;
- To assess the VE in the prevention of PHN from one month post dose 2 in the ZOSTER-006/022 studies until the end of the ZOSTER-049 study in subjects ≥ 50 YOA overall and within each of the specified age ranges* at the time of first vaccination in the ZOSTER-006/022 studies;
- To assess the VE over the total duration of the ZOSTER-049 study in prevention of HZ related complications (other than PHN) in subjects ≥ 50 YOA overall and within each of the specified age ranges* at the time of first vaccination in the ZOSTER-006/022 studies;
- To assess the VE in the prevention of HZ related complications (other than PHN) from one month post dose 2

in the ZOSTER-006/022 studies until the end of the ZOSTER-049 study in subjects ≥ 50 YOA overall and within each of the specified age ranges* at the time of first vaccination in the ZOSTER-006/022 studies;

- To assess persistence of humoral immune responses at Year 5, 6, 7, 8, 9 and 10 and beyond after the primary vaccination in the ZOSTER-006/022 studies in the HI subset in subjects ≥ 50 YOA overall and within each of the specified age ranges* at the time of first vaccination, in the ZOSTER-006/022 studies;
- To assess persistence of vaccine induced cell-mediated immune responses at Year 5, 6, 7, 8, 9 and 10 and beyond after the primary vaccination in the ZOSTER-006/022 studies in the CMI subset in subjects ≥ 50 YOA overall and within each of the specified age ranges* at the time of first vaccination, in the ZOSTER-006 study;
- To assess humoral immune responses at Year 5, 6, 7, 8, 9 and 10 and beyond after the primary vaccination in the ZOSTER-006/022 studies in subjects ≥ 50 YOA at the time of first vaccination, in the ZOSTER-006/022 studies, who had a confirmed HZ episode previously for the timepoint considered;
- To assess vaccine induced cell-mediated immune responses at Year 5, 6, 7, 8, 9 and 10 and beyond after the primary vaccination in the ZOSTER-006/022 studies **in the CMI subset** in subjects ≥ 50 YOA at the time of first vaccination, in the ZOSTER-006 study, who had a confirmed HZ episode previously for the timepoint considered;
- To assess humoral immune responses one month after the first additional HZ/su vaccine dose (1-Additional Dose and Revaccination groups) and at the same timepoint in the Control group;
- To assess vaccine induced cell-mediated immune responses one month after the first additional HZ/su vaccine dose (1-Additional Dose and Revaccination groups) and at the same timepoint in the Control group;
- To assess humoral immune responses one month after the second additional HZ/su vaccine doses (Revaccination group and at the same timepoint in the Control group);
- To assess vaccine induced cell-mediated immune responses one month after the second additional HZ/su vaccine doses (Revaccination group and at the same timepoint in the Control group);

- To assess persistence of humoral immune responses at Year 1, 2, 3, 4, 5 and 6 timepoints of this study in subjects from the 1-Additional Dose, Revaccination and Control groups;
- To assess persistence of vaccine induced cell-mediated immune responses at Year 1, 2, 3, 4, 5 and 6 timepoints of this study in subjects from the 1-Additional Dose, Revaccination and Control groups;
- To assess vaccine safety and reactogenicity in the 1-Additional Dose and Revaccination groups
- To assess vaccine safety in the LTFU and Control groups.

* Specified age range: 50-59 YOA, 60-69 YOA, ≥ 60 YOA and ≥ 70 YOA at time of primary vaccination in ZOSTER-006/022.

Tertiary

CCI [redacted]

Study design

- Experimental design: Phase IIIb, open-label, multi-centre, multi-country study with 4 groups.
- Duration of the study: Each subject will be followed for approximately 6 years.
- Epoch 001: LTFU of studies ZOSTER-006/022 starting at Visit Month 0 and ending at Visit Year 6 (Month 72) and assessment of 1 or 2 additional doses in two groups of subjects.
- Study groups:

Synopsis Table 1 Study groups and epochs foreseen in the study

Study groups	Number of subjects			Epochs
	From ZOSTER-006 primary study	From ZOSTER-022 primary study	Total	Epoch 001
ZOSTER-049 Total	≤ 7698	≤ 6750	≤ 14448 *	
LTFU			≤ 14208 **	x
1-Additional Dose			60	x
Revaccination			60	x
Control			120	x

* Maximum number of subjects expected for ZOSTER-049 based on the number of subjects in the ZOSTER-006/022 studies.

** All subjects (N ≤ 14,448) entering the study will have a HI blood sample (approximately 5 mL) at Visit Month 0. For subjects in the LTFU group, who do not belong to any subset, these samples will be stored and tested for HI only if the subject develops HZ during the ZOSTER-049 study or if there are other reasons requiring the HI testing of these samples.

Synopsis Table 2 Study groups and treatment foreseen in the study

Treatment name	Product name	Study groups			
		LTFU	1-Additional Dose	Revaccination	Control
HZ/su	VZV gE<br>$AS01_B$	None	x	x	None

- Control: historical control.
- Vaccination schedule: Two groups of subjects (1-Additional Dose and Revaccination) will receive 1 or 2 additional doses of HZ/su vaccine at Month 0 or Months 0 and 2, respectively.
- Treatment allocation: Randomised to treatment schedule accounting for age at primary vaccination in studies ZOSTER-006/022 (50-59, 60-69 and ≥ 70 YOA) and countries for the three groups 1-Additional Dose, Revaccination and Control.
- Blinding: Open-label.

Synopsis Table 3 Blinding of study epochs

Study Epochs	Blinding
Epoch 001	open-label

	• Sampling schedule:<br>**All subjects** (N ≤ 14,448) entering the study will have a HI blood sample (approximately 5 mL) at Visit Month 0.<br>For subjects in the LTFU group, who do not belong to any subset, these samples will be stored and tested for HI only if the subject develops HZ during the ZOSTER-049 study or if there are other reasons requiring the HI testing of these samples.<br>**LTFU Group (HI subset):** Subjects (N ≤ 1,729) who were in the immunogenicity subset during studies ZOSTER-006/022 and continue participation in this study. Blood samples (approximately 5 mL) will be collected and tested from Visit Month 0 to Visit Year 6, on a yearly basis, to assess HI responses.<br>**LTFU Group (CMI subset):** Subjects (N ≤ 234) who were in the CMI subset during study ZOSTER-006 and continue participation in this study. Blood samples (approximately 20 mL) will be collected and tested from Visit Month 0 to Visit Year 6 on a yearly basis to assess CMI responses.<br>**HZ subset:** Subjects (N to be determined) who developed confirmed HZ during ZOSTER-006 or ZOSTER-022, or who develop HZ during the interval between the end of the ZOSTER-006/022 and the beginning of ZOSTER-049, or who develop suspected HZ during ZOSTER-049 will be part of the HZ subset. Blood sampling for subjects in the HZ subset is described below.<br>Subjects who develop HZ at any time after enrolment in ZOSTER-006 or ZOSTER-022 and who are already part of the HI subset in ZOSTER-006 or ZOSTER-022 will provide blood samples to assess HI responses (approximately 5 mL) from Visit Month 0 to Visit Year 6 irrespective of when the HZ episode occurs. If these subjects are part of the ZOSTER-006 CMI subset, then they will also continue to provide blood to assess CMI responses (approximately 20 mL).<br>Subjects who develop HZ at any time after enrolment in ZOSTER-006 or ZOSTER-022 and who were NOT part of the HI or CMI subsets in these studies, will provide blood samples to assess HI responses (approximately 5 mL) beginning at the annual visit in the ZOSTER-049 study subsequent to the occurrence of the HZ episode. The blood sample taken upon enrolment at Visit Month 0 will be tested and included in the analyses for these subjects.

	**1-Additional Dose Group:** Subjects (N = 60) to be administered HZ/su vaccine on a 1-dose schedule at Visit Month 0. Blood samples (approximately 5 and 20 mL) will be collected at Visit Month 0, Visit Month 1, and from Visit Year 1 to Visit Year 6 on a yearly basis to assess HI and CMI responses.<br>**Revaccination Group:** Subjects (N = 60) to be administered HZ/su vaccine on a 2-dose schedule at Visit Month 0 and Visit Month 2. Blood samples (approximately 5 and 20 mL) will be collected at Visit Month 0, Visit Month 1, Visit Month 3 and from Visit Year 1 to Visit Year 6 on a yearly basis to assess HI and CMI responses.<br>**Control Group:** Subjects (N = 120) in the non-vaccinated control group. Blood samples (approximately 5 and 20 mL) will be collected at Visit Month 0, Visit Month 1, Visit Month 3 and from Visit Year 1 to Visit Year 6 on a yearly basis to assess HI and CMI responses.<br>**Specimens of HZ lesions** will be collected from subjects clinically diagnosed as having a suspected case of HZ.<br>***Refer to Section 6.6 for study procedures to be considered during special circumstances (Amended 11 May 2020)***.<br>• Type of study: extension of other protocol(s), i.e., 110390 (ZOSTER-006) and 113077 (ZOSTER-022).<br>• Data collection: Electronic Case Report Form (eCRF).
**Case definition**	• **Suspected HZ**<br>A suspected case of HZ is defined as new unilateral rash accompanied by pain (broadly defined to include allodynia, pruritus or other sensations) and no alternative diagnosis.<br>Subjects clinically diagnosed as having a suspected case of HZ by the investigator will be referred to as a case of 'suspected HZ', and followed up. If a case is not clinically diagnosed as suspected HZ, the investigator should not progress further with evaluation of the case.<br>The HZ onset date is the earlier of the following two events: 1) the HZ rash start date; or 2) the date on which pain at the site of a subsequent HZ rash is first noted.<br>• **Confirmed HZ**<br>A suspected case of HZ can be confirmed in two ways:<br>– By Polymerase Chain Reaction (PCR);<br>– By the HZ Ascertainment Committee (HZAC).

	• **Postherpetic neuralgia (PHN)**<br>PHN is defined by the presence of HZ-associated severe 'worst' pain persisting or appearing more than 90 days after onset of the HZ rash. Severe 'worst' pain is defined as HZ-associated pain rated as 3 or greater on the "worst pain" question on the Zoster Brief Pain Inventory (ZBPI) questionnaire.<br>• **HZ complications**<br>Any HZ complications will be recorded by the investigator in HZ-specific eCRF screens, and on the AE/SAE reporting screens as appropriate. The reporting period for HZ complications will be from Month 0 to study end. If a recorded complication is associated with a case of suspected HZ, and that case is finally not considered to be a confirmed case, the associated complication will not be considered as a complication of HZ.
**Number of subjects**	Expected number of subjects for enrolment will be up to 14,448 potentially eligible subjects.
**Endpoints**	**Primary**<br>• Confirmed HZ cases (LTFU and Control groups);<br>– Confirmed HZ cases during the ZOSTER-049 study.
	**Secondary**<br>• Confirmed HZ cases;<br>– Confirmed HZ cases since one month post dose 2 in the previous ZOSTER-006/022 studies;<br>• PHN cases;<br>– PHN cases during the ZOSTER-049 study and since one month post dose 2 in the previous ZOSTER-006/022 studies;<br>• HZ related complications (other than PHN);<br>– HZ related complications (other than PHN) during the ZOSTER-049 study and since one month post dose 2 in the previous ZOSTER-006/022 studies;<br>• Antigen-gE humoral immunogenicity at Months 0, 12, 24, 36, 48, 60 and 72 (LTFU HI subset, 1-Additional Dose, Revaccination and Control groups), at Month 1 (1-Additional Dose, Revaccination and Control groups), and at Month 3 (Revaccination and Control groups);<br>– Anti-gE Ab concentrations as determined by ELISA;

	• CMI in terms of frequencies of antigen-specific CD4+ T cells at Months 0, 12, 24, 36, 48, 60 and 72 (LTFU CMI subset, 1-Additional Dose, Revaccination and Control groups), at Month 1 (1-Additional Dose, Revaccination and Control groups), and at Month 3 (Revaccination and Control groups);<br>– Frequencies of CD4+ T cells with antigen-specific Interferon gamma (IFN-γ) and/or Interleukin-2 (IL-2) and/or Tumour Necrosis Factor alpha (TNF-α) and/or CD40 Ligand (CD40L) secretion/expression to gE as determined by ICS;<br>• Solicited local and general symptoms in subjects administered with 1 or 2 additional doses of HZ/su vaccine (1-Additional Dose and Revaccination groups);<br>– Occurrence, intensity and duration of each solicited local symptom within 7 days (Days 0 – 6) after each vaccination;<br>– Occurrence, intensity, duration and relationship to vaccination of each solicited general symptom within 7 days (Days 0 – 6) after each vaccination;<br>• Unsolicited AEs in subjects administered with 1 or 2 additional doses of HZ/su vaccine (1-Additional Dose and Revaccination groups);<br>– Occurrence, intensity and relationship to vaccination of unsolicited AEs during 30 days (Days 0 – 29) after each vaccination, according to the Medical Dictionary for Regulatory Activities (MedDRA) classification;<br>• Serious AEs;<br>– Occurrence and relationship to vaccination of all SAEs;<br>– from Visit Month 0 until 12 months: for Control and 1-Additional Dose groups;<br>– from Visit Month 0 until 12 months after last HZ/su vaccination: for the Revaccination group;<br>– Occurrence of SAEs related to investigational vaccine, related to study participation or to GSK concomitant medication/vaccine during the entire study period in all subjects;

	• Occurrence of AEs of specific interest: Potential immune-mediated diseases (pIMDs) (1-Additional Dose, Revaccination and Control groups);<br>– Occurrence and relationship to vaccination of all pIMDs;<br>– from Visit Month 0 until 12 months: for Control and 1-Additional Dose groups;<br>– from Visit Month 0 until 12 months after last HZ/su vaccination: for the Revaccination group.<br>**Tertiary**<br>CCI [redacted]

TABLE OF CONTENTS

PAGE

SPONSOR INFORMATION 8

SYNOPSIS 9

LIST OF ABBREVIATIONS 30

GLOSSARY OF TERMS 34

TRADEMARKS 38

1. INTRODUCTION 39
   1.1. Background 39
   1.2. Rationale for the study and study design 41
      1.2.1. Rationale for the study 41
      1.2.2. Rationale for the study design 41
   1.3. Benefit : Risk Assessment 43
      1.3.1. Risk Assessment 43
      1.3.2. Benefit Assessment 44
      1.3.3. Overall Benefit: Risk Conclusion 44

2. OBJECTIVES 44
   2.1. Primary objective 44
   2.2. Secondary objectives 44
   2.3. Tertiary Objectives 46

3. STUDY DESIGN OVERVIEW 47

4. CASE DEFINITION 52
   4.1. Suspected HZ 52
   4.2. Confirmed HZ 52
   4.3. Postherpetic neuralgia (PHN) 52
   4.4. HZ complications 53
   4.5. Evaluation and confirmation of suspected HZ cases 54
      4.5.1. Definitions 54
      4.5.2. Evaluation of suspected case of HZ 54
         4.5.2.1. For clinically diagnosed suspected HZ cases, the following will take place at Visit HZ-1: 55
         4.5.2.2. The following will take place at each visit or contact that occurs for each episode: 56
         4.5.2.3. Evaluation of severity of HZ-associated pain using the Zoster Brief Pain Inventory 57
         4.5.2.4. Confirmation of a suspected case of HZ 57
            4.5.2.4.1. Confirmation of suspected HZ by PCR 57
            4.5.2.4.2. Confirmation of suspected HZ by the HZAC 57
      4.5.3. Follow up of suspected HZ cases and HZ-associated pain 58

5. STUDY COHORT 59

5.1. Number of subjects/centres 59
5.2. Inclusion criteria for enrolment 60
5.3. Exclusion criteria for enrolment 61

6. CONDUCT OF THE STUDY 63
6.1. Regulatory and ethical considerations, including the informed consent process 63
6.1.1. Subject identification and randomisation of treatment 64
6.1.1.1. Randomisation of supplies 64
6.1.1.2. Randomisation of treatment 64
6.1.1.2.1. Treatment allocation to the subject for the 1-Additional Dose, Revaccination and Control groups 64
6.1.1.2.2. Treatment number allocation for subsequent doses 65
6.1.2. Allocation of subjects to assay subsets 65
6.2. Method of blinding 66
6.3. General study aspects 66
6.3.1. Data collection 66
6.3.1.1. Monthly contacts 66
6.3.1.2. Diary cards and questionnaires 66
6.4. Outline of study procedures 67
6.5. Detailed description of study procedures 76
6.5.1. Informed consent 76
6.5.2. Check inclusion and exclusion criteria 76
6.5.3. Medical history 76
6.5.4. Physical examination 77
6.5.5. Pregnancy test 77
6.5.6. Check contraindications, warnings and precautions to vaccination 77
6.5.7. Training on self-reporting by subjects 77
6.5.7.1. Reminder for monthly follow-up contacts/yearly follow-up visits 78
6.5.8. Sampling 78
6.5.8.1. Blood sampling for immune response assessments 78
6.5.8.2. Clinical specimens of HZ lesions for PCR analysis 79
6.5.9. Check and record concomitant medication/vaccination and IMCs 79
6.5.10. Recording of AEs and SAEs 80
6.5.11. Study conclusion 80
6.6. *Study procedures during special circumstances (Amended 11 May 2020)* 81
6.7. Biological sample handling and analysis 83
6.7.1. Use of specified study materials 84
6.7.2. Biological samples 84
6.7.3. Laboratory assays 85
6.7.4. Biological samples evaluation 86
6.7.4.1. Immunological read-outs 86
6.7.4.2. Test for laboratory diagnosis of HZ 87
6.7.5. Immunological correlates of protection 87

7. STUDY VACCINE AND ADMINISTRATION....87
7.1. Description of study vaccine....87
7.2. Storage and handling of study vaccine....88
7.3. Dosage and administration of study vaccine....88
7.4. Replacement of unusable vaccine doses....89
7.5. Contraindications to subsequent vaccination....89
7.6. Concomitant medications/products and concomitant vaccinations....90
7.6.1. Recording of concomitant medications/products and concomitant vaccinations....90
7.6.2. Concomitant medications/products/vaccines that may lead to the elimination of a subject from ATP analyses....91
7.7. Intercurrent medical conditions (IMCs) that may lead to elimination of a subject from ATP analyses....92

8. HEALTH ECONOMICS....92

9. SAFETY....92
9.1. Safety definitions....92
9.1.1. Definition of an adverse event....92
9.1.2. Definition of a serious adverse event....93
9.1.3. Solicited adverse events....94
9.1.3.1. Solicited local (injection-site) adverse events....94
9.1.3.2. Solicited general adverse events....94
9.1.4. Clinical laboratory parameters and other abnormal assessments qualifying as adverse events or serious adverse events....95
9.1.5. Medically attended visits....95
9.1.6. Adverse events of specific interest....95
9.1.6.1. Potential immune-mediated diseases....95
9.2. Events or outcomes not qualifying as adverse events or serious adverse events....97
9.2.1. Pregnancy....97
9.3. Detecting and recording adverse events, serious adverse events and pregnancies....98
9.3.1. Time period for detecting and recording adverse events, serious adverse events and pregnancies....98
9.3.2. Post-Study adverse events and serious adverse events....102
9.3.3. Evaluation of adverse events and serious adverse events....102
9.3.3.1. Active questioning to detect adverse events and serious adverse events....102
9.3.3.2. Assessment of adverse events....103
9.3.3.2.1. Assessment of intensity....103
9.3.3.2.2. Assessment of causality....104
9.3.3.3. Assessment of outcomes....105
9.4. Reporting of serious adverse events, pregnancies and other events....106
9.4.1. Prompt reporting of serious adverse events, pregnancies and other events to GSK Biologicals....106
9.4.2. Contact information for reporting serious adverse events, pregnancies and pIMDs....106
9.4.3. Completion and transmission of SAE reports to GSK Biologicals....107

9.4.3.1. Back-up system in case the electronic reporting system does not work 107
9.4.4. Completion and transmission of pregnancy reports to GSK Biologicals 107
9.4.5. Reporting of pIMDs to GSK Biologicals 108
9.4.6. Updating of SAE, pregnancy, and pIMD information after removal of write access to the subject's eCRF 108
9.4.7. Regulatory reporting requirements for serious adverse events 108
9.5. Follow-up of adverse events, serious adverse events, and pregnancies 109
9.5.1. Follow-up of adverse events and serious adverse events 109
9.5.1.1. Follow-up during the study 109
9.5.1.2. Follow-up after the subject is discharged from the study 109
9.5.2. Follow-up of pregnancies 110
9.6. Treatment of adverse events 110
9.7. Subject card 110

10. SUBJECT COMPLETION AND WITHDRAWAL 110
10.1. Subject completion 110
10.2. Subject withdrawal 110
10.2.1. Subject withdrawal from the study 110
10.2.2. Subject withdrawal from investigational vaccine 111

11. STATISTICAL METHODS 112
11.1. Primary endpoint 112
11.2. Secondary endpoints 112
11.3. Tertiary endpoints 113
11.4. Determination of sample size 113
11.4.1. VE assessment 114
11.5. Cohorts for Analyses 115
11.5.1. Total vaccinated cohort 115
11.5.2. Modified Total Vaccinated cohort (LTFU and Control group) 115
11.5.3. According To Protocol cohort for analysis of efficacy (LTFU and Control groups) 116
11.5.4. According To Protocol cohort for analysis of immunogenicity (1-Additional Dose, Revaccination and Control groups) 116
11.5.5. According To Protocol cohort for analysis of persistence of immunogenicity (LTFU group) 117
11.5.6. According To Protocol cohort for analysis of persistence of immunogenicity (1-Additional Dose, Revaccination and Control groups) 117
11.6. Derived and transformed data 118
11.6.1. Handling of missing data 118
11.6.2. Humoral immune response 118
11.6.3. CMI response 119
11.7. Statistical analyses 120
11.7.1. Analysis of demographics (for each of the four groups of the study) 120

11.7.2. Analysis of efficacy (LTFU and Control groups) 120
11.7.3. Analysis of immunogenicity 121
11.7.3.1. Assessment for 1-Additional Dose, Revaccination and Control groups 122
11.7.3.1.1. Humoral immune response 122
11.7.3.1.2. Cell-mediated immune response 123
11.7.3.2. Analysis on subjects with HZ cases 123
11.7.3.3. Analysis of persistence (immune subset of LTFU group) 123
11.7.3.3.1. Humoral immune response 124
11.7.3.3.2. Cell-mediated immune response 124
11.7.4. Analysis of safety 125
11.8. Interpretation of analyses 126
11.9. Conduct of analyses 126
11.9.1. Sequence of analyses 126
11.9.2. Statistical considerations for interim analyses 127

12. ADMINISTRATIVE MATTERS 127
12.1. electronic Case Report Form instructions 127
12.2. Study Monitoring by GSK Biologicals 127
12.3. Record retention 128
12.4. Quality assurance 129
12.5. Posting of information on publicly available clinical trial registers and publication policy 129
12.6. Provision of study results to investigators 129

13. COUNTRY SPECIFIC REQUIREMENTS 130
13.1. Requirements for France 130
13.2. Requirements for Germany 134
13.3. Requirements for Japan 135

14. REFERENCES 138

LIST OF TABLES

		PAGE
Table 1	Study groups and epochs foreseen in the study	50
Table 2	Study groups and treatment foreseen in the study	50
Table 3	Blinding of study epochs	50
Table 4	Subsets	59
Table 5	List of study procedures (for the LTFU group)	68
Table 6	List of study procedures (for the 1-Additional Dose, Revaccination and Control groups)	69
Table 7	Study procedures to be performed during the follow-up period for each suspected HZ case	72
Table 8	Intervals between study visits for the LTFU group	74
Table 9	Intervals between study visits for the 1-Additional Dose and Control groups	74
Table 10	Intervals between study visits for the Revaccination group	75
Table 11	Intervals between visits/contacts for subjects in case of suspected HZ	75
Table 12	*Intervals for blood sampling for the LTFU group during special circumstances (Amended 11 May 2020)*	81
Table 13	*Intervals for blood sampling for the 1-Additional Dose and Control groups during special circumstances (Amended 11 May 2020)*	82
Table 14	*Intervals for blood sampling for the Revaccination group during special circumstances (Amended 11 May 2020)*	82
Table 15	Biological samples	84
Table 16	Humoral Immunity (Antibody determination)	85
Table 17	Cell-Mediated Immunity (CMI)	85
Table 18	Molecular Biology (PCR tests)	85
Table 19	Immunological read-outs	86
Table 20	Study vaccine	87
Table 21	Dosage and administration	89

Table 22 Solicited local adverse events 94

Table 23 Solicited general adverse events 94

Table 24 List of potential immune-mediated diseases 96

Table 25 Reporting periods for collecting safety information (for the LTFU group) 99

Table 26 Reporting periods for collecting safety information (for the 1-Additional Dose, Revaccination and Control groups) 100

Table 27 Intensity scales for solicited symptoms in adults 103

Table 28 Timeframes for submitting serious adverse events, pregnancy and other event reports to GSK Biologicals 106

Table 29 Probability to get a Lower Limit (LL) of 95% CI (95%LL) of the VE above 0% every year considering a VE of 80% 114

Table 30 Probability to get a Lower Limit (LL) of 95% CI (95%LL) of the VE above 0% every year considering a VE of 65% 115

Table 31 GSK Biologicals' laboratories 144

Table 32 Outsourced laboratories 144

LIST OF FIGURES

PAGE

Figure 1 Study design overview 47

Figure 2 Detailed overview of the 1-Additional Dose, Revaccination and Control groups (Year 1 only) 48

Figure 3 Algorithm for HZ case definition by PCR 143

LIST OF APPENDICES

PAGE

APPENDIX A LABORATORY ASSAYS 140

APPENDIX B CLINICAL LABORATORIES 144

APPENDIX C AMENDMENTS AND ADMINISTRATIVE CHANGES TO THE PROTOCOL 145

LIST OF ABBREVIATIONS

**Ab:**	Antibody
**AE:**	Adverse event
**ANCOVA:**	Analysis of Covariance
**$AS01_B$:**	MPL, QS21, liposome based Adjuvant System (50 µg MPL and 50 µg QS21)
**ATP:**	According-To-Protocol
**CD40 L:**	CD40 Ligand
**CDC:**	Centres for Disease Control (and Prevention)
**CEVAC:**	Centre for Vaccination
**CI:**	Confidence Interval
**CMI:**	Cell-Mediated Immunogenicity/Immunity
COVID-19:	***Coronavirus Disease 2019 (Amended 11 May 2020)***
**CT:**	Computerised Tomography
**CVA:**	Cerebrovascular Accident
**DNA:**	Deoxyribonucleic Acid
**eCRF:**	electronic Case Report Form
**EDD:**	Estimated Date of Delivery
**EGA:**	Estimated Gestational Age
**ELISA:**	Enzyme Linked Immunosorbent Assay
CCI [redacted]	
**EMA:**	European Medicines Agency
**(e)TDF:**	Electronic Temperature excursion Decision Form
**FDA:**	Food and Drug Administration, United States
**FSFV:**	First Subject First Visit
**GCP:**	Good Clinical Practice

**gE:**	VZV glycoprotein E
**GMC:**	Geometric Mean Concentration
**GSK:**	GlaxoSmithKline
**HI:**	Humoral Immunogenicity
**HZ/su:**	Herpes Zoster subunit vaccine
**HZ:**	Herpes Zoster
**HZAC:**	Herpes Zoster Ascertainment Committee
**IB:**	Investigator Brochure
**IC:**	Immunocompromised
**ICF:**	Informed Consent Form
**ICH:**	International Conference on Harmonisation
**ICS:**	Intracellular Cytokine Staining
**IEC:**	Independent Ethics Committee
**IFN-γ:**	Interferon Gamma
**IgG:**	Immunoglobulin class G
**IL-2:**	Interleukin-2
**IM:**	Intramuscular
**IMC:**	Intercurrent Medical Condition
**IMP:**	Investigational Medicinal Product
**IND:**	Investigational New Drug
**IRB:**	Institutional Review Board
**LL:**	Lower Limit
**LLL:**	Lower Limit of Linearity
**LMP:**	Last Menstrual Period
**LOD:**	Limit of Detection

**LSLV/C:**	Last Subject Last Visit/Contact
**LSLV:**	Last Subject Last Visit
**LTFU:**	Long-Term Follow-up
**MACDP:**	Metropolitan Atlanta Congenital Defects Program
**MAR:**	Missing At Random
**MATEX:**	MATerial EXcellence
**MCAR:**	Missing Completely At Random
**MedDRA:**	Medical Dictionary for Regulatory Activities
**MGI:**	Mean Geometric Increase
**mIU:**	Milli-International Unit
**mL:**	Millilitre
**MRI:**	Magnetic Resonance Imaging
**mTVc:**	Modified Total Vaccinated cohort
**ORF:**	Open Reading Frame
**PBMC:**	Peripheral Blood Mononuclear Cells
**PCR:**	Polymerase Chain Reaction
**PHN:**	Postherpetic Neuralgia
**pIMD:**	Potential Immune-Mediated Disease
**Post-Vacc:**	Post-Vaccination
**Pre-Vacc:**	Pre-Vaccination
**PT:**	Preferred Term
**SAE:**	Serious Adverse Event
**SAP:**	Statistical Analysis Plan
**SAS:**	Statistical Analysis System
**SBIR:**	Randomisation System on Internet

**SD:**	Standard Deviation
**SDV:**	Source Document Verification
**SmPC:**	Summary of Product Characteristics
**SMS:**	Short Message Service
**SPM:**	Study Procedures Manual
**su:**	Subunit
**TNF-α:**	Tumour Necrosis Factor Alpha
**TVc:**	Total Vaccinated cohort
**UL:**	Upper Limit
**VE:**	Vaccine Efficacy
**VRR:**	Vaccine Response Rate
**VZV:**	Varicella-Zoster Virus
**YOA:**	Years of age
**ZBPI:**	Zoster Brief Pain Inventory
**µg:**	Microgram

GLOSSARY OF TERMS

Adequate contraception: Adequate contraception is defined as a contraceptive method with failure rate of less than 1% per year when used consistently and correctly and when applicable, in accordance with the product label for example:

- abstinence from penile-vaginal intercourse, when this is their preferred and usual lifestyle,
- oral contraceptives, either combined or progestogen alone,
- injectable progestogen,
- implants of etenogestrel or levonorgestrel,
- estrogenic vaginal ring,
- percutaneous contraceptive patches,
- intrauterine device or intrauterine system,
- male partner sterilisation prior to the female subject's entry into the study, and this male is the sole partner for that subject,

  The information on the male sterility can come from the site personnel's review of the subject's medical records; or interview with the subject on her medical history.
- male condom combined with a vaginal spermicide (foam, gel, film, cream or suppository),
- male condom combined with a female diaphragm, either with or without a vaginal spermicide (foam, gel, film, cream, or suppository).

Adequate contraception does not apply to subjects of child bearing potential with same sex partners, when this is their preferred and usual lifestyle.

Adverse event: Any untoward medical occurrence in a patient or clinical investigation subject, temporally associated with the use of a medicinal product, whether or not considered related to the medicinal product.

An adverse event (AE) can therefore be any unfavourable and unintended sign (including an abnormal laboratory finding), symptom, or disease (new or exacerbated) temporally associated with the use of a medicinal product. For marketed medicinal products, this also includes failure to produce expected benefits (i.e., lack of efficacy), abuse or misuse.

**Blinding:**	A procedure in which one or more parties to the trial are kept unaware of the treatment assignment in order to reduce the risk of biased study outcomes. The level of blinding is maintained throughout the conduct of the trial, and only when the data are cleaned to an acceptable level of quality will appropriate personnel be unblinded or when required in case of a serious adverse event (SAE). In an open-label study, no blind is used. Both the investigator and the subject know the identity of the treatment assigned.
**Caregiver**	Someone who lives in the close surroundings of a subject having a continuous caring role or may be someone having substantial periods of contact with a subject and is engaged in his/her daily health care (e.g., a relative of the subject, a nurse who helps with daily activities in case of residence in a nursing home). In a context of a clinical study, a caregiver could include an individual appointed to oversee and support the subject's compliance with protocol specified procedures.
**Eligible:**	Qualified for enrolment into the study based upon strict adherence to inclusion/exclusion criteria.
**Epoch:**	An epoch is a self-contained set of consecutive timepoints or a single timepoint from a single protocol. Self-contained means that data collected for all subjects at all timepoints within that epoch allows to draw a complete conclusion to define or precise the targeted label of the product. Typical examples of epochs are primary vaccinations, boosters, yearly immunogenicity follow-ups, and surveillance periods for efficacy or safety.
**eTrack:**	GSK’s tracking tool for clinical trials.
**Evaluable:**	Meeting all eligibility criteria, complying with the procedures defined in the protocol, and, therefore, included in the according-to-protocol (ATP) analysis.
**Intercurrent Medical Condition:**	It is defined as a condition with onset during the study period that has the capability of confounding the immune response to the study vaccine or its interpretation. Examples of other IMCs include a confirmed or suspected immunosuppressive or immunodeficient condition resulting from disease (e.g., HIV infection, malignancy). Occurrence of HZ during this study will be considered an intercurrent medical condition (IMC).

**Immunological correlate of protection:**	The defined immune response above which there is a high likelihood of protection in the absence of any host factors that might increase susceptibility to the infectious agent.
**Investigational vaccine/product:**<br>**(Synonym of Investigational Medicinal Product)**	A pharmaceutical form of an active ingredient or placebo being tested or used as a reference in a clinical trial, including a product with a marketing authorisation when used in a way different from the approved form, or when used for an unapproved indication, or when used to gain further information about an approved use.
**Menarche:**	Menarche is the onset of menses for the first time in a young female and is preceded by several changes associated with puberty including breast development and pubic hair growth. Menarche usually occurs within 1-2 years of breast development, thelarche. However, a young female can become pregnant before her first menses. Thus, a conservative definition of non-childbearing potential in a pre-menarcheal female is a young female who has not yet entered puberty as evidenced by lack of breast development (palpable glandular breast tissue).
**Menopause:**	Menopause is the age associated with complete cessation of menstrual cycles, menses, and implies the loss of reproductive potential by ovarian failure. A practical definition accepts menopause after 1 year without menses with an appropriate clinical profile at the appropriate age e.g., > 45 years.
**Potential Immune-Mediated Disease:**	Potential immune-mediated diseases (pIMDs) are a subset of AEs that include autoimmune diseases and other inflammatory and/or neurologic disorders of interest which may or may not have an autoimmune aetiology.
**Primary completion date:**	The date that the final subject was examined or received an intervention for the purpose of final collection of data for the primary outcome, whether the clinical trial concluded according to the pre-specified protocol or was terminated.
**Protocol amendment:**	The International Conference on Harmonisation (ICH) defines a protocol amendment as: 'A written description of a change(s) to or formal clarification of a protocol.' GSK Biologicals further details this to include a change to an approved protocol that affects the safety of subjects, scope of the investigation, study design, or scientific integrity of the study.

**Protocol administrative change:**	A protocol administrative change addresses changes to only logistical or administrative aspects of the study.
**Randomisation:**	Process of random attribution of treatment to subjects in order to reduce bias of selection.
**Self-contained study:**	Study with objectives not linked to the data of another study.
**Site Monitor:**	An individual assigned by the sponsor who is responsible for assuring proper conduct of clinical studies at one or more investigational sites.
**Solicited adverse event:**	AEs to be recorded as endpoints in the clinical study. The presence/occurrence/intensity of these events is actively solicited from the subject or an observer during a specified post-vaccination follow-up period.
**Subject:**	Term used throughout the protocol to denote an individual who has been contacted in order to participate or participates in the clinical study, either as a recipient of the vaccine(s)/product(s) or as a control.
**Subject number:**	A unique number identifying a subject, assigned to each subject consenting to participate in the study.
**Treatment:**	Term used throughout the clinical study to denote a set of investigational product(s) or marketed product(s) or placebo intended to be administered to a subject, identified by a unique number, according to the study randomisation or treatment allocation.
**Treatment number:**	A number identifying a treatment to a subject, according to the study randomisation or treatment allocation.
**Unsolicited adverse event:**	Any AE reported in addition to those solicited during the clinical study. Also any ‘solicited’ symptom with onset outside the specified period of follow-up for solicited symptoms will be reported as an unsolicited adverse event.

TRADEMARKS

The following trademarks are used in the present protocol.

Note: In the body of the protocol (including the synopsis), the names of the vaccines/products and/or medications will be written without the superscript symbol ™ or ® and in *italics*.

Trademarks not owned by the GlaxoSmithKline group of companies	Generic description
Varivax (Merck & Co., Inc.)	Varicella vaccine consisting of live attenuated varicella-zoster virus (Oka strain)
Zostavax (Merck & Co., Inc.)	Herpes zoster vaccine consisting of high-titre live attenuated Varicella-zoster virus (Oka strain)

Trademarks of the GSK group of companies		Generic description
Shingrix		Herpes zoster vaccine non-live recombinant, $AS01_B$ adjuvanted

1. INTRODUCTION

1.1. Background

Varicella-zoster Virus (VZV) causes two distinct diseases. Varicella (chickenpox) occurs shortly after primary VZV infection and is characterised by systemic illness and a widely disseminated rash. Herpes zoster (HZ), commonly called shingles, occurs when VZV reactivates from latency and typically manifests as a localised, dermatomal rash.

The typical HZ rash usually lasts 2 to 4 weeks and is typically accompanied by pain that is often described as burning, shooting, or stabbing. In some patients, even touching the affected area lightly may cause pain, a phenomenon known as allodynia. This HZ-associated pain may be severe, and pruritus, which can also be severe, may be as common as pain.

The most common complication of HZ is postherpetic neuralgia (PHN). PHN is defined as pain that persists after the resolution of the HZ rash. Affected patients typically report constant burning, throbbing, intermittent sharp or electric shock-like pain, or allodynia. Older age is a clear risk factor for PHN. Other risk factors may include a severe HZ rash and a painful HZ prodrome. PHN tends to improve over a period of months. About 70-80% of cases resolve within 1 year, however, in some persons PHN persists for many years [Dworkin, 2007].

Other complications of HZ include ophthalmologic, neurological, cutaneous and visceral disease, which can result in severe disability. The most common ocular complications of HZ are keratitis and uveitis; other ophthalmologic complications include ptosis, episcleritis/scleritis, retinitis, secondary glaucoma and cataract [Schmader, 2008; Carter, 2008]. Neurologic complications associated with HZ include myelitis, motor neuropathy, ischaemic infarction of the brain and spinal cord, aneurysm, and subarachnoid and cerebral haemorrhage [Gilden, 2009; Schmader, 2008].

Age is the most common risk factor for developing HZ. The incidence of HZ is relatively constant at 2-3 cases per 1000 persons per year until age 40, and then increases progressively with age: At 50-59 years of age (YOA) the incidence is about 5 cases per 1000 persons per year, and it increases to 10 cases per 1000 persons per year in people ≥ 60 YOA [CDC, 2008; Oxman, 2005]. While most HZ incidence data come from the United States (US) and Europe, available data indicate similar incidences of HZ in other parts of the world including Japan, Korea, Australia and Latin America [Araújo, 2007; Garcia, 2008; Kang, 2008; Toyama, 2009].

Half of all HZ cases occur in patients over the age of 60, and individuals who reach 85 years old have a 50% chance of having HZ during their lifetime [Oxman, 2005]. The risk for PHN is also highest in older people with HZ, occurring in 18-50% of those aged 70 years and older [Oxman, 2005; Scott, 2006]. Patients with impaired cell-mediated immunity (CMI) due to disease, drug treatment, medical interventions or advanced age are at increased risk for the development of HZ [Cohen, 2007]. Since the loss of VZV-specific T cell responses as a result of aging or immunosuppression leads to heightened susceptibility to HZ, vaccination is considered as a means to reduce the risk of HZ in older adults and immunocompromised persons [Oxman, 2005; Sperber, 1992].

The potential of vaccination to protect against HZ was evaluated in a large efficacy study in which *Zostavax* (a live attenuated HZ vaccine that is a high titre preparation of the varicella vaccine, *Varivax* [both manufactured by Merck & Co**.,** Inc.]) partially protected immunocompetent older adults against HZ [Oxman, 2005]. In the overall population (≥ 60 YOA), *Zostavax* reduced the incidence of HZ by 51.3% (p-value < 0.001), although its efficacy decreased with the age of the vaccinee. In particular, vaccine efficacy (VE) diminished to 37.6% among persons in older age groups (≥ 70 YOA) [Oxman, 2005]. Based on the data from this study, *Zostavax* was licensed in the US and other countries. In the US, *Zostavax* is indicated for prevention of HZ in individuals ≥ 50 YOA and older [Zostavax Prescribing information, 2011]. In Australia, *Zostavax* is indicated for the prevention of HZ, PHN and for reduction of acute and chronic HZ-associated pain in individuals ≥ 60 YOA, and for the prevention of HZ in individuals 50-59 YOA [TGA, 2015]. In Europe, *Zostavax* is indicated for prevention of HZ and PHN in individuals ≥ 50 YOA [EMEA, 2011]. *Zostavax* is contraindicated in persons with immunodeficiency due to malignancy, human immunodeficiency virus (HIV) infection or immunosuppressive medical therapy.

In a short term persistence sub-study of the large efficacy trial (Shingles Prevention Study) it was reported that *Zostavax* continued to reduce the incidence of HZ and PHN in subjects evaluated from 3.3 to 7.8 years after vaccination [Schmader, 2012]. However, the recent long-term persistence study of *Zostavax* showed that the VE for three outcomes (1. HZ burden of illness [a severity-by-duration measure of HZ pain and discomfort]; 2. incidence of PHN; and 3. incidence of HZ) declined from year 7 through year 11 post vaccination [Morrison, 2015].

GlaxoSmithKline (GSK) Biologicals' study vaccine (Shingrix) for the prevention of HZ, is a recombinant subunit (su) vaccine consisting of Varicella Zoster Virus (VZV) glycoprotein E (gE) as antigen and an adjuvant system (AS01), has been and is being evaluated in several studies in older adults and immunocompromised adults. In these studies it was shown to elicit strong cellular and humoral immune responses. Furthermore, the safety and reactogenicity profile of the study vaccine was clinically acceptable. Based on phase II data from the antigen dose-ranging study, ZOSTER-003, and the adjuvant dose comparison study, ZOSTER-010, a gE antigen dose of 50 μg and the adjuvant system $AS01_B$ were selected for the final vaccine formulation. Henceforth, the final vaccine formulation will be referred to as HZ/su. The results of the previous studies in older adults demonstrated strong vaccine-induced immune responses following HZ/su administration at 0 and 2 months, supporting the selection of a 2-dose vaccine schedule.

Two large pivotal phase III trials ZOE-50 and ZOE-70 that enrolled subjects ≥ 50 and ≥ 70 years of age (YOA) respectively, evaluated the vaccine efficacy (VE), immunogenicity and safety of GSK Biologicals' HZ/su vaccine. These trials, hereafter referred to as ZOSTER-006 and ZOSTER-022, respectively and collectively referred to as the ZOSTER-006/022 studies, enrolled more than 30,000 subjects who either received the HZ/su vaccine or placebo on a 0, 2-month schedule.

The final HZ/su VE results from the ZOSTER-006 phase III trial demonstrated the HZ/su vaccine to be highly efficacious in the prevention of HZ overall and in all age strata and available safety data until the data lock point raised no safety concerns in this population of subjects ≥ 50 YOA [Lal, 2015]. The results for the ZOSTER-022 study demonstrated that the HZ/su vaccine reduced the risks of HZ and PHN among adults ≥ 70 YOA [Cunningham, 2016].

An indication in adults ≥ 50 YOA was filed for registration. HZ/su (trade name Shingrix) was first approved in Canada and the United States in October 2017.

Please refer to the current Investigator Brochure (IB) for information regarding the pre-clinical and clinical studies of HZ/su vaccine.

1.2. Rationale for the study and study design

1.2.1. Rationale for the study

Since the mean follow-up period for VE at the time of the final analysis in the ZOSTER-006/022 studies was about four years, further follow-up for long term efficacy assessment is needed in order to establish that the HZ/su vaccine provides not only strong, but also persistent protection.

This study will also assess the immunogenicity responses of 1 or 2 additional doses in two subgroups of older adults who previously received two doses of the HZ/su vaccine in the ZOSTER-006/022 studies. If over time, VE of HZ/su wanes, additional vaccination with HZ/su could be needed to prevent HZ and its complications. Knowing if 1 or 2 additional doses of HZ/su vaccine can stimulate the immune response again, could support the concept of providing additional doses with the expectation that this may translate into preserving VE.

1.2.2. Rationale for the study design

The primary objective of this study is to assess the efficacy of the HZ/su vaccine in the prevention of HZ. The data from this study will also be analysed combined with ZOSTER-006/022 data (analysis over the full duration of the follow-up in the primary ZOSTER-006/022 studies and the ZOSTER-049 study). Persistence of immunogenicity and long-term safety of the HZ/su vaccine administered in ZOSTER-006/022 will also be evaluated. Safety, reactogenicity, and immunogenicity one month after 1 or 2 additional doses of the HZ/su vaccine in subjects who previously received 2 doses of the vaccine in the primary ZOSTER-006/022 studies will also be assessed to further support evaluation of additional vaccination if it is determined VE wanes over time.

Due to the high VE observed in the ZOSTER-006/022 studies, it was considered that subjects having received placebo during both of the studies should be offered cross-vaccination with HZ/su vaccine as soon as possible. Since they will be enrolled in a separate cross-vaccination study, no placebo recipients will be available for this study and historic controls will be used for assessment of VE.

All subjects that were previously vaccinated with at least one dose of the HZ/su vaccine in the ZOSTER-006/022 primary studies, and confirmed their interest to enrol in the study at participating centres, will be considered for entry in study ZOSTER-049, after they have completed all study-related activities in the ZOSTER-006 or ZOSTER-022 studies and when their treatment assignment can be released without compromising the integrity of the ZOSTER-006/022 data. Since the interval between the end of the ZOSTER-006/022 studies and the start of study ZOSTER-049 will vary per subject and is dependent on receipt of approval or implementing the study in the different participating countries/centres, the study will analyze the annual LTFU data from Year 5 up to Year 10 and annually beyond Year 10 after the primary vaccination in the ZOSTER-006/022 studies (in case samples from these timepoints are collected).

The study will be comprised of four study groups as follows:

LTFU Group: Subjects from this group [N ≤ 14,000] will be followed for VE and safety. In addition, immunogenicity will be followed in subjects that were part of the immunogenicity subset (HI) in the ZOSTER-006/022 studies and Cell-Mediated Immunity (CMI) subset from the ZOSTER-006 study;

1-Additional Dose Group: Subjects from this group [N = 60] will receive 1 additional dose of the HZ/su vaccine, at the time of enrolment, to assess the immunogenicity, reactogenicity and safety of 1 additional dose;

Revaccination Group: Subjects from this group [N = 60] will be revaccinated with 2 additional doses of the HZ/su vaccine, on a 0, 2-month schedule from the time of enrolment, to assess the immunogenicity, reactogenicity and safety of 2 additional doses;

Control Group: Subjects from this group [N = 120] will not be vaccinated but will serve as a control to assess immunogenicity and safety compared to the two vaccinated groups 1-Additional Dose and Revaccination. This Control group will also be used to evaluate the long-term VE, safety and immunogenicity.

Subjects (N, to be determined) who developed HZ during the ZOSTER-006/022 studies (confirmed HZ) and/or during the interval between the end of the ZOSTER-006/022 studies and beginning of the ZOSTER-049 study, and/or during the ZOSTER-049 study (suspected HZ) will be part of the **HZ subset.**

Subjects from the 1-Additional Dose and Revaccination groups who will develop HZ cases during the ZOSTER-049 study will follow the sampling schedule as per their original group and will not be part of the HZ subset.

Note: Subjects from the immunogenicity subset of studies ZOSTER-006/022 are not allocated to the 1-Additional Dose, Revaccination and Control groups. Subjects in these three groups will be enrolled amongst subjects who received 2 doses of the HZ/su vaccine and were part of the ATP cohort for analysis of efficacy in the previous studies ZOSTER-006/022. In order not to confound immune responses, subjects who developed HZ prior to enrolment in the ZOSTER-049 study (confirmed HZ during the ZOSTER-006/022 studies or suspected HZ during the interval between the end of the ZOSTER-006/022 studies and beginning of the ZOSTER-049 study), will not be enrolled in the 1-Additional Dose, Revaccination and Control groups (Refer to Table 4 and Section 6.1.2).

1.3. Benefit : Risk Assessment

Please refer to the current IB for the summary of potential risks and benefits of HZ/su vaccine.

The following section outlines the risk assessment and mitigation strategy for this study protocol:

1.3.1. Risk Assessment

Important Potential/Identified Risk	Data/Rationale for Risk	Mitigation Strategy
**Investigational HZ/su vaccine**		
Theoretical risk of acquiring a vaccine induced autoimmune disease after vaccination (1-Additional Dose and Revaccination groups only).	No confirmed signals related to this potential risk have been identified during the clinical program. Available clinical data do not highlight any concern.	Close monitoring of pIMDs as per study protocol.<br>The potential risk of events of possible autoimmune aetiology to occur is mentioned in the ICF. In addition, the ICF advises subjects to contact the study doctor or the study staff immediately, should they get any symptoms that they feel maybe serious.
Hypersensitivity reactions (including anaphylaxis)	No confirmed signals related to this potential risk have been identified during the clinical program. Available clinical data do not highlight any concern.	Administration of the study vaccination is to be preceded by a review of the subjects' medical history (especially with regard to previous vaccination and possible occurrence of undesirable events) and a clinical examination.<br>As with all injectable vaccines, appropriate medical treatment and supervision should always be readily available in case of an anaphylactic event following the administration of the vaccine.
**Study Procedures**		
Risk from blood sampling.	Blood sampling associated risk of discomfort, syncope, dizziness, infection at the site after or during venipuncture.	Blood samples will be obtained by a trained professional and medical assistance will be available.<br>The potential risk of feeling faint, or experiencing mild local pain, bruising, irritation or redness at the site where blood was taken, is mentioned in the ICF. The amount of blood to be taken for sampling will not be harmful to the subject's health.
Risk from lesion sampling.	Swab/ needle sampling of lesions/crusts associated risk of secondary infection, and discomfort related to the procedure.	Lesion samples will be obtained by a trained professional and anti-bacterial ointment may be applied to minimise the potential for secondary infection.<br>The potential risk of some temporary discomfort during the sampling procedure and the precautionary use of an anti-bacterial ointment to reduce the risk of infection are mentioned in the ICF.

1.3.2. Benefit Assessment

Benefits include:

- Potential benefit of receiving the study vaccine that may provide a clinical benefit in case the VE of HZ/su vaccine wanes over time (for the vaccinated groups 1-Additional Dose and Revaccination).
- Medical evaluations/assessments associated with study procedures [e.g., physical examination].

1.3.3. Overall Benefit: Risk Conclusion

Taking into account the measures to minimise risk to subjects participating in this study, the potential or recognised risks identified in association with the investigational HZ/su vaccine and study procedures are offset by the potential benefits (prolonged prevention of HZ and related complications in case of waning VE for subjects in the 1-Additional Dose and Revaccination groups, and additional medical follow-up and assessments) that may be afforded to the subject(s).

2. OBJECTIVES

The statistical analyses to be performed for the study objectives described in Sections 2.1 and 2.2 will be descriptive. No success criteria have been defined.

2.1. Primary objective

- To assess the VE in the prevention of HZ over the total duration of the ZOSTER-049 study as measured by the reduction in HZ risk in subjects ≥ 50 YOA overall at the time of first vaccination in the ZOSTER-006/022 studies.

Refer to Section 11.1 for the definition of the primary endpoint.

2.2. Secondary objectives

- To assess the VE in the prevention of HZ over the total duration of the ZOSTER-049 study as measured by the reduction in HZ risk in subjects within each of the age ranges* at the time of first vaccination in the ZOSTER-006/022 studies;
- To assess the VE in the prevention of HZ from one month post dose 2 in the ZOSTER-006/022 studies until the end of the ZOSTER-049 study as measured by the reduction in HZ risk in subjects ≥ 50 YOA overall and within each of the specified age ranges* at the time of first vaccination in the ZOSTER-006/022 studies;
- To assess the VE in the prevention of HZ over each year of follow-up from one month post dose 2 in the ZOSTER-006/022 studies as measured by the reduction in HZ risk in subjects ≥ 50 YOA overall and within each of the specified age ranges* at the time of first vaccination in the ZOSTER-006/022 studies;

- To assess the VE over the total duration of the ZOSTER-049 study in prevention of PHN in subjects ≥ 50 YOA overall and within each of the specified age ranges* at the time of first vaccination in the ZOSTER-006/022 studies;
- To assess the VE in the prevention of PHN from one month post dose 2 in the ZOSTER-006/022 studies until the end of the ZOSTER-049 study in subjects ≥ 50 YOA overall and within each of the specified age ranges* at the time of first vaccination in the ZOSTER-006/022 studies;
- To assess the VE over the total duration of the ZOSTER-049 study in prevention of HZ related complications (other than PHN) in subjects ≥ 50 YOA overall and within each of the specified age ranges* at the time of first vaccination in the ZOSTER-006/022 studies;
- To assess the VE in the prevention of HZ related complications (other than PHN) from one month post dose 2 in the ZOSTER-006/022 studies until the end of the ZOSTER-049 study in subjects ≥ 50 YOA overall and within each of the specified age ranges* at the time of first vaccination in the ZOSTER-006/022 studies;
- To assess persistence of humoral immune responses at Year 5, 6, 7, 8, 9 and 10 and beyond after the primary vaccination in the ZOSTER-006/022 studies in the HI subset in subjects ≥ 50 YOA overall and within each of the specified age ranges* at the time of first vaccination, in the ZOSTER-006/022 studies;
- To assess persistence of vaccine induced cell-mediated immune responses at Year 5, 6, 7, 8, 9 and 10 and beyond after the primary vaccination in the ZOSTER-006/022 studies **in the CMI subset** in subjects ≥ 50 YOA overall and within each of the specified age ranges* at the time of first vaccination, in the ZOSTER-006 study;
- To assess humoral immune responses at Year 5, 6, 7, 8, 9 and 10 and beyond after the primary vaccination in the ZOSTER-006/022 studies in subjects ≥ 50 YOA at the time of first vaccination, in the ZOSTER-006/022 studies, who had a confirmed HZ episode previously for the timepoint considered;
- To assess vaccine induced cell-mediated immune responses at Year 5, 6, 7, 8, 9 and 10 and beyond after the primary vaccination in the ZOSTER-006/022 studies **in the CMI subset** in subjects ≥ 50 YOA at the time of first vaccination, in the ZOSTER-006 study, who had a confirmed HZ episode previously for the timepoint considered;
- To assess humoral immune responses one month after the first additional HZ/su vaccine dose (1-Additional Dose and Revaccination groups) and at the same timepoint in the Control group;
- To assess vaccine induced cell-mediated immune responses one month after the first additional HZ/su vaccine dose (1-Additional Dose and Revaccination groups) and at the same timepoint in the Control group;
- To assess humoral immune responses one month after the second additional HZ/su vaccine doses (Revaccination group and at the same timepoint in the Control group);
- To assess vaccine induced cell-mediated immune responses one month after the second additional HZ/su vaccine doses (Revaccination group and at the same timepoint in the Control group);

- To assess persistence of humoral immune responses at Year 1, 2, 3, 4, 5 and 6 timepoints of this study in subjects from the 1-Additional Dose, Revaccination and Control groups;
- To assess persistence of vaccine induced cell-mediated immune responses at Year 1, 2, 3, 4, 5 and 6 timepoints of this study in subjects from the 1-Additional Dose, Revaccination and Control groups;
- To assess vaccine safety and reactogenicity in the 1-Additional Dose and Revaccination groups;
- To assess vaccine safety in the LTFU and Control groups.

* Specified age range: 50-59 YOA, 60-69 YOA, ≥ 60 YOA and ≥ 70 YOA at time of primary vaccination in ZOSTER-006/022.

Refer to Section 11.2 for the definition of the secondary endpoints.

2.3. Tertiary Objectives

CCI

[redacted]

3. STUDY DESIGN OVERVIEW

Figure 1 Study design overview

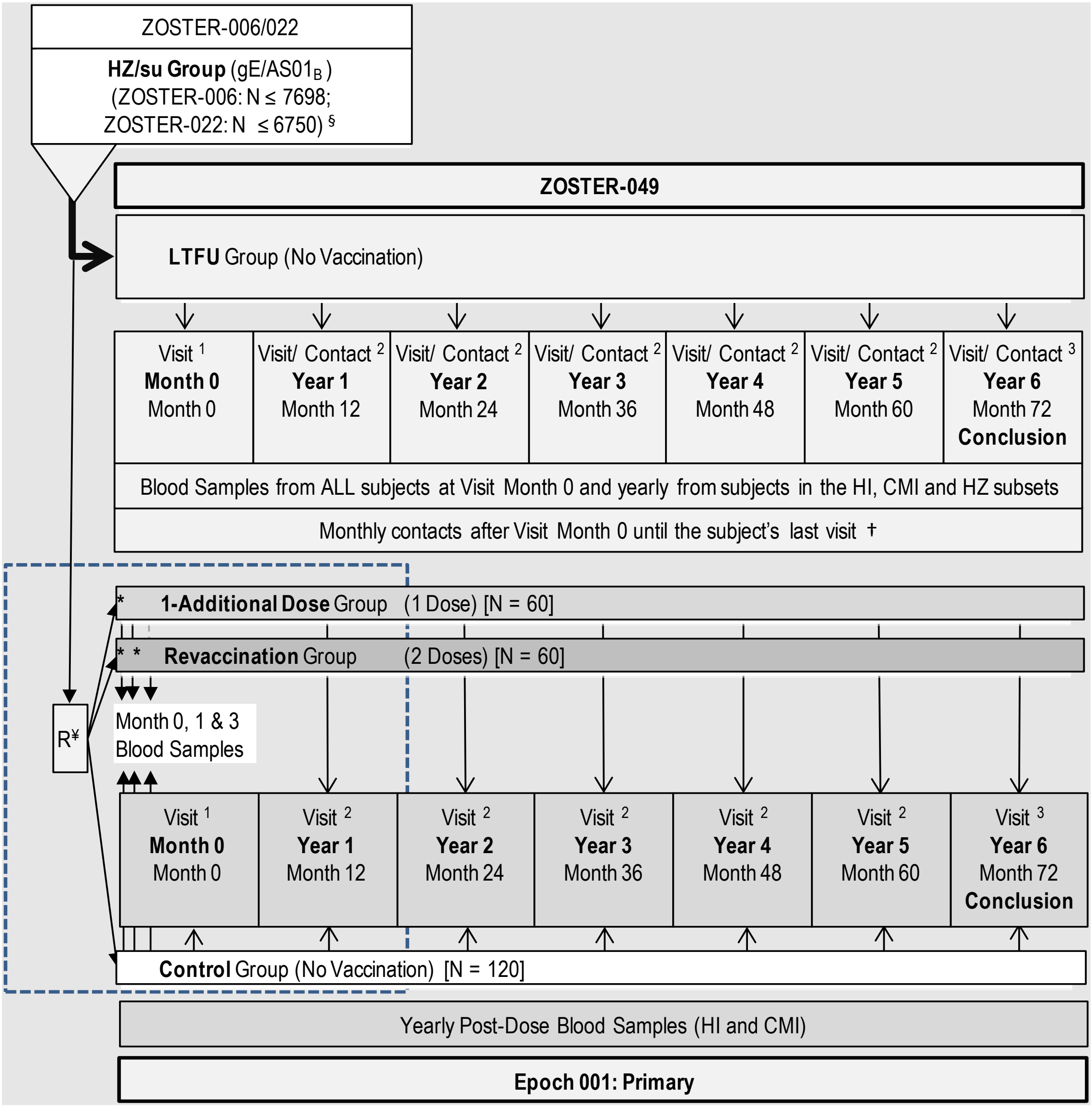

§ N = Maximum number of subjects expected for ZOSTER-049 based on the number of subjects in the ZOSTER-006/022 studies.

Note: The interval between the end of ZOSTER-006/022 studies and start of study ZOSTER-049 is depicted by the gray inverted triangle. There is to be a database freeze and unblinding of subjects at the end of the ZOSTER-006/022 studies and before ZOSTER-049 begins. The interval between the end of the ZOSTER-006/022 studies and start of study the ZOSTER-049 will vary per subject and is dependent on receipt of approval or implementing the study in the different participating countries/centres.

[1] A new reference timepoint Visit Month 0 is set for the first visit in ZOSTER-049.

[2] From Visit Month 0 onwards, yearly visits/contacts occur at Months 12, 24, 36, 48, 60 and 72 (Refer to Section 6.4).

[3] Study conclusion takes place at the last yearly visit/ contact (Refer to Section 6.5.11).

† Monthly phone contacts will be done to assess HZ cases and safety follow up for all study groups and will NOT occur when subjects have a scheduled visit (see Table 5 and Table 6).

¥ R= Randomisation 1:1:2.

See Figure 2 for study activities up to Year 1 for the 1-Additional Dose, Revaccination and Control groups.

Note: In case of suspected HZ, there are additional visits and phone contacts for follow-up of the HZ episode (see Table 7).

Figure 2 Detailed overview of the 1-Additional Dose, Revaccination and Control groups (Year 1 only)

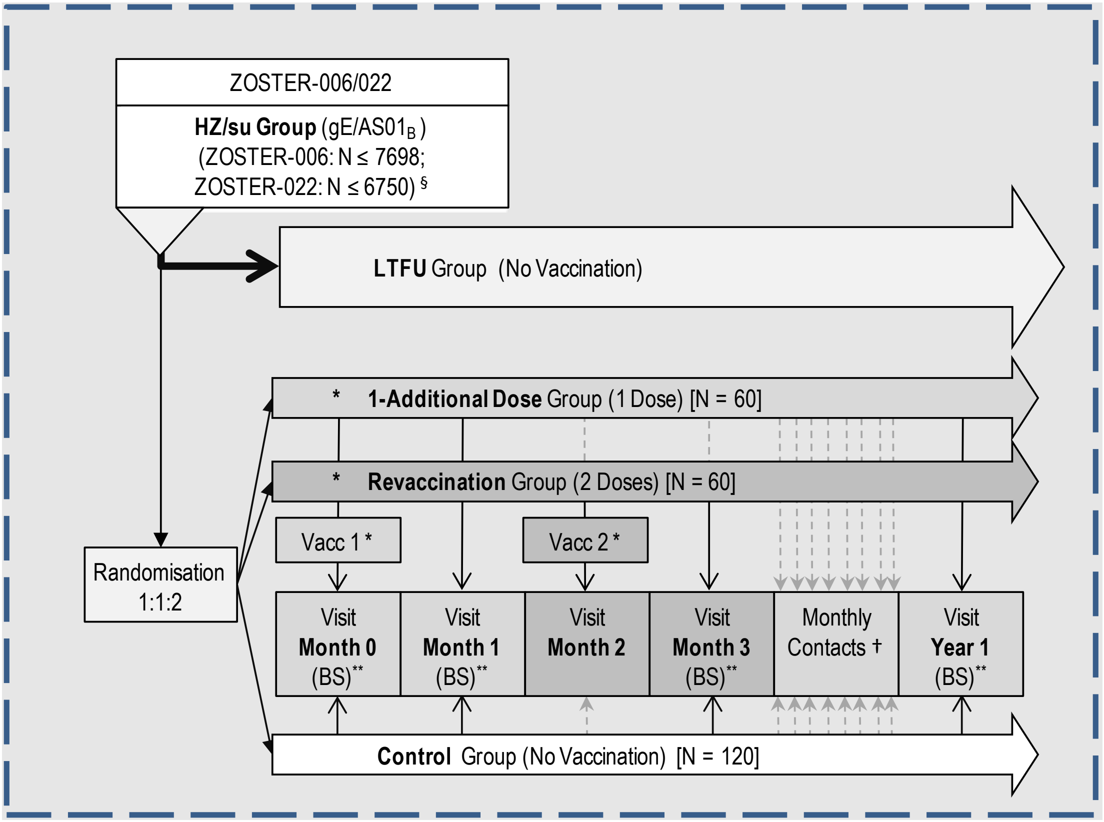

§ N = Maximum number of subjects expected for ZOSTER-049 based on the number of subjects in the ZOSTER-006/022 studies.

* The two groups (1-Additional Dose and Revaccination, only) will have a HZ/su vaccination (Vacc 1) at Visit Month 0 and the Revaccination group will have a second dose (Vacc 2) at Visit Month 2 (see Table 6 and Table 21).

** Blood sampling (BS) as in the Sampling schedule in Section 3, Table 4, Table 15 and Table 19.

Refer to Figure 1 for study activities beyond Year 1 and to Table 9 and Table 10 for the intervals between study visits for the 1-Additional Dose and Control groups and for the Revaccination group, respectively.

† Monthly phone contacts will be done to assess HZ cases and safety follow up for all study groups and will NOT occur when subjects have a scheduled visit (see Table 5 and Table 6).

Protocol waivers or exemptions are not allowed with the exception of immediate safety concerns. Therefore, adherence to the study design requirements, including those specified in the outline of study procedures (Section 6.4), are essential and required for study conduct. ***Refer to Section 6.6 for study procedures to be considered during special circumstances (Amended 11 May 2020)***.

- Experimental design: Phase IIIb, open-label, multi-centre, multi-country study with 4 groups.
- Duration of the study: Each subject will be followed for approximately 6 years.
- Epoch 001: LTFU of studies ZOSTER-006/022 starting at Visit Month 0 and ending at Visit Year 6 (Month 72) and assessment of 1 or 2 additional doses in two groups of subjects.

- Study groups:

The study will be comprised of four study groups as follows:

LTFU Group: Subjects from this group [N ≤ 14,000] will be followed for VE and safety. In addition, immunogenicity will be followed in subjects that were part of the immunogenicity subset (HI) in the ZOSTER-006/022 studies and Cell-Mediated Immunity (CMI) subset from the ZOSTER-006 study;

1-Additional Dose Group: Subjects from this group [N = 60] will receive 1 additional dose of the HZ/su vaccine, at the time of enrolment, to assess the immunogenicity, reactogenicity and safety of 1 additional dose;

Revaccination Group: Subjects from this group [N = 60] will be revaccinated with 2 additional doses of the HZ/su vaccine, on a 0, 2-month schedule from the time of enrolment, to assess the immunogenicity, reactogenicity and safety of 2 additional doses;

Control Group: Subjects from this group [N = 120] will not be vaccinated but will serve as a control to assess immunogenicity and safety compared to the two vaccinated groups 1-Additional Dose and Revaccination. This Control group will also be used to evaluate the VE, safety and immunogenicity.

Subjects (N, to be determined) who developed HZ during the ZOSTER-006/022 studies (confirmed HZ) and/or during the interval between the end of the ZOSTER-006/022 studies and beginning of the ZOSTER-049 study, and/or during the ZOSTER-049 study (suspected HZ) will be part of the **HZ subset**.

Subjects from the 1-Additional Dose and Revaccination groups who will develop HZ cases during the ZOSTER-049 study will follow the sampling schedule as per their original group and will not be part of the HZ subset.

Note: Subjects from the immunogenicity subset of studies ZOSTER-006/022 are not allocated to the 1-Additional Dose, Revaccination and Control groups. Subjects in these three groups will be enrolled amongst subjects who received 2 doses of the HZ/su vaccine and were part of the ATP cohort for analysis of efficacy in the previous studies ZOSTER-006/022. In order not to confound immune responses, subjects who developed HZ prior to enrolment in the ZOSTER-049 study (confirmed HZ during the ZOSTER-006/022 studies or suspected HZ during the interval between the end of the ZOSTER-006/022 studies and beginning of the ZOSTER-049 study), will not be enrolled in the 1-Additional Dose, Revaccination and Control groups (Refer to Table 4 and Section 6.1.2).

Table 1 Study groups and epochs foreseen in the study

Study groups	Number of subjects			Epochs
	From ZOSTER-006 primary study	From ZOSTER-022 primary study	Total	Epoch 001
ZOSTER-049 Total	≤ 7698	≤ 6750	≤ 14448 *	
LTFU			≤ 14208 **	x
1-Additional Dose			60	x
Revaccination			60	x
Control			120	x

* Maximum number of subjects expected for ZOSTER-049 based on the number of subjects in the ZOSTER-006/022 studies.

** All subjects (N ≤ 14,448) entering the study will have a HI blood sample (approximately 5 mL) at Visit Month 0. For subjects in the LTFU group, who do not belong to any subset, these samples will be stored and tested for HI only if the subject develops HZ during the ZOSTER-049 study or if there are other reasons requiring the HI testing of these samples (see Section 6.5.8.1).

Table 2 Study groups and treatment foreseen in the study

Treatment name	Product name	Study groups			
		LTFU	1-Additional Dose	Revaccination	Control
HZ/su	VZV gE	None	x	x	None
	$AS01_B$				

- Control: historical control.
- Vaccination schedule: Two groups of subjects (1-Additional Dose and Revaccination) will receive 1 or 2 additional doses of HZ/su vaccine at Month 0 or Months 0 and 2, respectively.
- Treatment allocation: Randomised to treatment schedule accounting for age at primary vaccination in studies ZOSTER-006/022 (50-59, 60-69 and ≥ 70 YOA) and countries for the three groups 1-Additional Dose, Revaccination and Control.
- Blinding: Open-label.

Table 3 Blinding of study epochs

Study Epochs	Blinding
Epoch 001	open-label

- Sampling schedule:

  All subjects (N ≤ 14,448) entering the study will have a HI blood sample (approximately 5 mL) at Visit Month 0.

  For subjects in the LTFU group, who do not belong to any subset, these samples will be stored and tested for HI only if the subject develops HZ during the ZOSTER-049 study or if there are other reasons requiring the HI testing of these samples.

  LTFU Group (HI subset): Subjects (N ≤ 1,729) who were in the immunogenicity subset during studies ZOSTER-006/022 and continue participation in this study. Blood samples (approximately 5 mL) will be collected and tested from Visit Month 0 to Visit Year 6, on a yearly basis, to assess HI responses.

LTFU Group (CMI subset): Subjects (N ≤ 234) who were in the CMI subset during study ZOSTER-006 and continue participation in this study. Blood samples (approximately 20 mL) will be collected and tested from Visit Month 0 to Visit Year 6 on a yearly basis to assess CMI responses.

HZ subset: Subjects (N to be determined) who developed confirmed HZ during ZOSTER-006 or ZOSTER-022, or who develop HZ during the interval between the end of the ZOSTER-006/022 and the beginning of ZOSTER-049, or who develop suspected HZ during ZOSTER-049 will be part of the HZ subset. Blood sampling for subjects in the HZ subset is described below.

Subjects who develop HZ at any time after enrolment in ZOSTER-006 or ZOSTER-022 and who are already part of the HI subset in ZOSTER-006 or ZOSTER-022 will provide blood samples to assess HI responses (approximately 5 mL) from Visit Month 0 to Visit Year 6 irrespective of when the HZ episode occurs. If these subjects are part of the ZOSTER-006 CMI subset, then they will also continue to provide blood to assess CMI responses (approximately 20 mL).

Subjects who develop HZ at any time after enrolment in ZOSTER-006 or ZOSTER-022 and who were NOT part of the HI or CMI subsets in these studies, will provide blood samples to assess HI responses (approximately 5 mL) beginning at the annual visit in the ZOSTER-049 study subsequent to the occurrence of the HZ episode. The blood sample taken upon enrolment at Visit Month 0 will be tested and included in the analyses for these subjects.

1-Additional Dose Group: Subjects (N = 60) to be administered HZ/su vaccine on a 1-dose schedule at Visit Month 0. Blood samples (approximately 5 and 20 mL) will be collected at Visit Month 0, Visit Month 1, and from Visit Year 1 to Visit Year 6 on a yearly basis to assess HI and CMI responses.

Revaccination Group: Subjects (N = 60) to be administered HZ/su vaccine on a 2-dose schedule at Visit Month 0 and Visit Month 2. Blood samples (approximately 5 and 20 mL) will be collected at Visit Month 0, Visit Month 1, Visit Month 3 and from Visit Year 1 to Visit Year 6 on a yearly basis to assess HI and CMI responses.

Control Group: Subjects (N = 120) in the non-vaccinated control group. Blood samples (approximately 5 and 20 mL) will be collected at Visit Month 0, Visit Month 1, Visit Month 3 and from Visit Year 1 to Visit Year 6 on a yearly basis to assess HI and CMI responses.

Specimens of HZ lesions will be collected from subjects clinically diagnosed as having a suspected case of HZ.

- Type of study: extension of other protocols, i.e., 110390 (ZOSTER-006) and 113077 (ZOSTER-022).
- Data collection: Electronic Case Report Form (eCRF).

4. CASE DEFINITION

4.1. Suspected HZ

A suspected case of HZ is defined as new unilateral rash accompanied by pain (broadly defined to include allodynia, pruritus or other sensations) and no alternative diagnosis.

Subjects clinically diagnosed as having a suspected case of HZ by the investigator will be referred to as a case of 'suspected HZ', and followed up. If a case is not clinically diagnosed as suspected HZ, the investigator should not progress further with evaluation of the case. Also refer to Section 4.5.

The HZ onset date is the earlier of the following two events: 1) the HZ rash start date; or 2) the date on which pain at the site of a subsequent HZ rash is first noted.

The end date of a HZ episode is defined as the first time at which a subject had no rash (papules, vesicles, ulcers or crusts) present. This end date will be recorded in the eCRF.

The occurrence of HZ will be recorded in HZ-specific eCRF screens. The reporting period for cases of HZ will be from Month 0 to study end. Refer to Section 4.5 for more details on the evaluation and confirmation of suspected HZ cases.

Subjects who developed confirmed HZ during ZOSTER-006 or ZOSTER-022, or who develop HZ during the interval between the end of the ZOSTER-006/022 and the beginning of ZOSTER-049, or who develop suspected HZ during ZOSTER-049 will be part of the HZ subset (Table 4 and Section 6.5.8.1).

4.2. Confirmed HZ

A suspected case of HZ can be confirmed in two ways:

- By Polymerase Chain Reaction (PCR):

  Rash lesion samples will be collected from subjects clinically diagnosed as having a suspected case of HZ. The samples will be transferred to GSK Biologicals or a validated laboratory designated by GSK Biologicals using standardised and validated procedures for laboratory diagnosis of HZ by PCR (see Section 4.5.2.4.1).

- By the HZ Ascertainment Committee (HZAC):

  All suspected HZ cases will be referred to the HZAC. The HZAC will classify all referred cases as either "HZ" or "not HZ" (see Section 4.5.2.4.2).

4.3. Postherpetic neuralgia (PHN)

PHN is defined by the presence of HZ-associated severe 'worst' pain persisting or appearing more than 90 days after onset of the HZ rash. Severe 'worst' pain is defined as HZ-associated pain rated as 3 or greater on the "worst pain" question on the Zoster Brief Pain Inventory (ZBPI) questionnaire (see Sections 6.3.1.2 and 4.5.2.3).

Cessation of pain to assess duration of HZ-associated pain: A 28-day pain-free period is used to confirm cessation of HZ-associated pain.

Acute pain is defined as pain measured during the 4-week period following the onset of confirmed HZ.

4.4. HZ complications

Any HZ complications listed below will be recorded by the investigator in HZ-specific eCRF screens, and on the AE/SAE reporting screens. The reporting period for HZ complications will be from Month 0 to study end. Any HZ complications, according to the definitions below, will be recorded by the investigator. If a recorded complication is associated with a case of suspected HZ, and that case is finally not considered to be a confirmed case, the associated complication will not be considered as a complication of HZ.

HZ vasculitis	Vasculopathy or vasculitis (based on clinical, laboratory or radiologic findings) that is temporally associated with an episode of HZ and, in the opinion of the investigator, was caused directly by the VZV infection arising from the HZ episode.
Disseminated disease	Defined as ≥ 6 HZ lesions outside the primary dermatome as per the investigator's judgment.
Ophthalmic disease	Defined as HZ affecting any eye structure as per investigator's judgment.
Neurologic disease	Defined as cranial or peripheral nerve palsies, myelitis, meningoencephalitis, stroke, etc. that is temporally associated with an episode of HZ and, in the opinion of the investigator, was caused directly by VZV infection arising from the HZ episode.
Visceral disease	Defined as an abnormality of one or more internal organs (e.g., hepatitis, pneumonitis, gastroenteritis, etc.) that is temporally associated with an episode of HZ and, in the opinion of the investigator, was caused directly by VZV infection arising from the HZ episode.
Stroke A	diagnosis of stroke requires that criteria 1, 2 and 3 are fulfilled or criteria 1 and 4 and in the opinion of the investigator is temporally associated with an episode of HZ<br>Criterion 1: Rapid onset of localising neurological deficit and/or change in level of consciousness;<br>Criterion 2: Localising neurological deficit or change in level of consciousness that lasts greater than 24 hours;<br>Criterion 3: No other cerebral process, peripheral lesion, or other disorder is the cause of the localising neurological deficit or change in level of consciousness;

Criterion 4: Computerised Tomography (CT) scan or Magnetic Resonance Imaging (MRI) scan evidence of an acute thrombotic or hemorrhagic lesion.

4.5. Evaluation and confirmation of suspected HZ cases

4.5.1. Definitions

Refer to Sections 4.1, 4.2, 4.3 and 4.4 for definitions of suspected HZ, confirmed HZ PHN and HZ complications, respectively.

For all subjects in case of a suspected or confirmed case of HZ:

HZ-specific diary card: To be completed by subjects (or subject's caregiver) who develop symptoms suggestive of HZ beginning immediately upon development of these symptoms and prior to visiting the study site for evaluation of the suspected HZ.

Zoster Brief Pain Inventory (ZBPI) questionnaire: To be completed by subjects with suspected HZ (or subject's caregiver) on Day HZ-0 (Visit HZ-1) and daily from Day HZ-1 (day after the Visit HZ-1) up to Day HZ-28, and weekly from Day HZ-29 onwards until a 4-week pain-free period is documented. For all subjects with ongoing HZ-associated pain at the time of last subject last visit/contact (LSLV/C), ZBPI data will be collected until a 4-week pain-free period is documented OR until at least Day HZ-91 (Refer to Section 4.5.2 for more details).

Please refer to Table 5, Table 6 and Table 7 for information on when diary cards and questionnaires are dispensed to the subjects.

4.5.2. Evaluation of suspected case of HZ

All HZ cases that occur during the study period up to the LSLV/C will be followed and evaluated as was done for the previous ZOSTER-006/022 studies. Any symptom/sign suggestive of HZ must be evaluated. At Visit Month 0, all subjects (and subject's caregiver if applicable) will be re-educated with regard to the signs and symptoms of HZ. The subjects are also given a HZ-specific diary card that they would complete with the date that rash and/or pain began. Subjects will be instructed to contact their study site immediately, and start completing the HZ-specific diary card (with the help of the subject caregiver if applicable) if he/she develops any symptoms suggestive of HZ. The subjects will be asked to visit the study site (within 48 hours if possible) for evaluation of the "suspected case of HZ". The subject will be asked to bring the completed HZ-specific diary card when he/she visits the study site for evaluation of the suspected HZ. The investigator will perform a clinical examination when the subject visits the study site for the first evaluation of the suspected case of HZ [Visit HZ-1 at Day HZ-0]. If not considered a suspected HZ diagnosis, the investigator should not progress further with evaluation of this event as a HZ case for the purpose of this study. However, if meeting the definition of an AE/SAE (Section 9.1), the case should be handled as applicable (Section 9.3).

The schedule of visits/contacts that will take place for follow-up of suspected HZ cases is presented in Table 7.

4.5.2.1. For clinically diagnosed suspected HZ cases, the following will take place at Visit HZ-1:

- The investigator or his delegate will verify the completed HZ-specific diary card returned by the subject and the subject's caregiver if applicable. The information from the diary card will be transcribed into the eCRF. The investigator or his delegate will record relevant information regarding the HZ episode in the eCRF (such as date of onset of pain and rash, date of clinical diagnosis of HZ, location and nature of HZ lesions, HZ-related complications if any);
- The study staff/investigator will ask the subject (with the help of subject's caregiver if necessary) to complete a ZBPI questionnaire at Visit HZ-1 to rate HZ-associated pain within the last 24 hours. If the time between the HZ onset and clinical evaluation at Visit HZ-1 is greater than 24 hours, the subject will be asked to complete a second ZBPI also for the elapsed time between the HZ onset and 24 hours before Visit HZ-1;
- The rash will be documented by digital photography. Please refer to the SPM for specific instructions;
- The study staff/investigator will record concomitant medication/vaccination, including concomitant medication for HZ treatment or any HZ-related complications (Section 7.6), and record intercurrent medical conditions (Section 7.7). If antiviral therapy is needed, it is recommended to use valacyclovir, acyclovir or famciclovir. Concomitant medication the subject has already received and/or will receive for HZ treatment will be recorded in the eCRF. The study staff/investigator will check if the subject received any medical attention [hospitalization, emergency room visit, or a visit to or from medical personnel (medical doctor)] for HZ or any HZ-related complication.
- Rash lesion samples (three replicate samples on the same day) will be collected from subjects clinically diagnosed as having a suspected case of HZ (Section 4.5.3);
- The subject will be given a supply of ZBPI questionnaires. The ZBPI questionnaires will be used to collect information on the severity and duration of HZ-associated pain. The study staff/investigator will provide instructions to the subjects for completing the ZBPI questionnaires and explain the importance of completing and returning the questionnaires to the site in order to provide more information on HZ.
- The subject/subject's caregiver will be asked to complete the ZBPI questionnaires on Day HZ-0 (Visit HZ-1) and daily from Day HZ-1 (day after the Visit HZ-1) up to Day 28 (ZBPI must be completed to Day HZ-28 at minimum) and weekly from Day HZ-29 onwards until:
  - 28 days after HZ-associated pain ceases. The subject/ subject's caregiver should continue to complete the ZBPI questionnaires weekly until a 28-day (or 4-week) pain free period is documented (a 'No' answer to the ZBPI question: 'Have you had any pain caused by your shingles in the last 24 hours' (item 1) at each assessment during that entire period);

  - For all subjects with ongoing HZ-associated pain at the time of LSLV/C, completion of ZBPI questionnaires will continue until a 4-week pain-free period is documented OR until at least Day HZ-91.

- After Visit HZ-1 until Visit HZ-7, visits/contacts will take place for follow-up of the HZ episode according to the schedule presented in Table 7. Follow-up of HZ-associated pain and complications will continue irrespective of whether the rash has ended in some cases. When a case initially clinically diagnosed as suspected HZ is subsequently no longer considered by the investigator as suspected HZ, this will be noted in the eCRF. However, study procedures to be performed during the follow-up period for a suspected HZ case (see Table 7) should be continued.

If HZ-associated pain ceases (defined as a 28-day [or 4-week] pain free period), the study staff/investigator will inform the subject/ subject's caregiver to stop completing the ZBPI questionnaires and will provide instructions for the subject to return the completed questionnaires to the study site.

If a 4-week pain-free period is achieved and the HZ rash resolves, subsequent follow-up visits or contacts related to this case of HZ will be cancelled meaning that collection of subsequent HZ episode-related information will be stopped and no further information on that ***particular*** suspected HZ episode will be encoded in the clinical database. However, if pain reappears in the same area after a 4-week pain-free period and is not accompanied by a new HZ rash, it will be assigned to the previous HZ-episode. Visits/contacts will restart with Day HZ-0 defined as the first visit of the assigned episode, prior to the pain free period.

Follow-up of HZ-associated pain persisting beyond Visit HZ-7 (Day HZ-91) or other complications will be done at monthly contacts between the subject and the investigator and/or investigator's delegate.

HZ related complications if considered as SAE will be followed as appropriate.

4.5.2.2. The following will take place at each visit or contact that occurs for each episode:

- The study staff/investigator will: 1) record relevant information regarding the suspected HZ case (such as the location and nature of HZ lesions, the end date of the rash, HZ-related complications, if any); 2) record concomitant medications/vaccinations, including concomitant medication the subject has already received and/or will receive for HZ treatment or treatment of any HZ-related complications (Section 7.6); 3) record intercurrent medical conditions (Section 7.7); and 4) check if the subject received any medical attention [hospitalization, emergency room visit, or a visit to or from medical personnel (medical doctor)] for HZ or any HZ-related complication.
- Additional photographs of HZ lesions may be taken after the first visit for the HZ episode to help note the progression of the rash.
- If the investigator determines that adequate rash samples are not present at the first Visit (i.e., <3 lesions present or only papules present), the investigator has the option of collecting three additional samples prior to or at the second Visit HZ-2 (see Section 4.5.3).

- The study staff/investigator will remind subjects/subject's caregiver to complete the ZBPI questionnaires, and return the completed ZBPI questionnaires to the study site according to the instructions given by the study staff/investigator. Once the completed ZBPI questionnaires are available, the investigator will transcribe the information into the subject's eCRF. A new supply of ZBPI questionnaires will be provided to the subjects as necessary.

4.5.2.3. Evaluation of severity of HZ-associated pain using the Zoster Brief Pain Inventory

The ZBPI is an assessment tool in the form of a questionnaire completed by the subject/ subject's caregiver that is specifically designed to assess HZ-associated pain and discomfort during an HZ episode.

In each case of suspected HZ, the subjects will be asked to assess their HZ-associated pain by completing the ZBPI questionnaire either themselves or assisted, by an aide (such as a family member or caregiver who is not involved in the study) (Section 6.3.1.2, see also further details in Section 4.5.2).

Information on HZ-associated pain is derived from the ZBPI question: "Please rate your pain by circling the one number that best describes your pain at its worst in the last 24 hours" (item 3), so called "worst pain" in this protocol.

4.5.2.4. Confirmation of a suspected case of HZ

A suspected case of HZ can be confirmed in two ways:

4.5.2.4.1. Confirmation of suspected HZ by PCR

Rash lesion samples will be collected from subjects clinically diagnosed as having a suspected case of HZ. The samples will be transferred to GSK Biologicals or a validated laboratory designated by GSK Biologicals using standardised and validated procedures for laboratory diagnosis of HZ by PCR. Refer to APPENDIX A for details of PCR assay to be performed on HZ lesion samples and for details of the PCR testing algorithm to classify suspected cases of HZ.

4.5.2.4.2. Confirmation of suspected HZ by the HZAC

All suspected HZ cases will be referred to the HZAC. The HZAC will classify all referred cases as either "HZ" or "not HZ" ***or "not able to decide".*** However, the HZAC classification will serve as the final case definition only when the case cannot be confirmed or excluded by PCR, e.g., when all samples from a given subject are inadequate (as when both VZV and β-actin PCR results are negative), or when no samples are available for a given subject. Therefore, definitive PCR results, when available, will determine the final HZ case assignment. In such cases, the HZAC classification will not contribute to HZ case determination decision ***(Amended 11 May 2020)***.

The HZAC will consist of three to five physicians with HZ expertise. HZAC members, participating as investigator in this study, will not evaluate cases from their own study site. HZAC members will be blinded to group assignments. For every case, each reviewing HZAC member will be asked to make a clinical determination of whether the case is HZ based on review of the available clinical information (e.g., summary of the rash and pain evaluations, digital photographs of the subject's rash, and clinical progress notes). A suspected case of HZ will be considered as "HZ" if the HZAC members concur unanimously; otherwise, it will be classified as "not HZ". ***A case of "not able to decide" will be classified as "not HZ".*** As described above, the HZAC case assignment will only be considered as the final case assignment if definitive PCR results are not available. Further details will be provided in the HZAC charter ***(Amended 11 May 2020)***.

4.5.3. Follow up of suspected HZ cases and HZ-associated pain

Data will be collected on all suspected HZ cases that occur from Visit Month 0 until the study conclusion Visit. For each suspected case of HZ that the investigator concludes is clinically consistent with HZ, data on HZ-associated pain (using ZBPI questionnaires completed by the subject) will be collected daily until Day- HZ-28, and weekly from Day HZ-29 until the subject has no HZ-associated pain for 4 consecutive weeks.

For all subjects with ongoing HZ-associated pain at the time of LSLV/C, ZBPI data will be collected until a 4-week pain-free period is documented OR until Day HZ-91. If pain reappears in the same area after a 4-week pain-free period and is not accompanied by a new HZ rash, it will be assigned to the previous HZ-episode. The completion of the ZBPI questionnaire will resume based upon the weekly schedule established at the start of the assigned episode. Visits/contacts will also restart according to the schedule in Table 7 with Day HZ-0 defined as the first visit of the assigned episode, prior to the pain free period. Follow-up is described in Section 4.5.2.

At the first HZ evaluation visit (Visit HZ-1 at Day HZ-0 – the visit at which the suspected case of HZ is first evaluated by the investigator), rash lesion samples will be collected from the subject if the investigator considers the symptoms/signs to be consistent with HZ. Three replicate rash lesion samples (see Table 15) should be collected on the same day. If during clinical evaluation at Visit HZ-1, the investigator determines that adequate rash lesion samples cannot be collected (i.e., less than three lesions present, or if only papules are present), the investigator has the option of collecting three additional samples preferably within 7 days, or at the Visit HZ-2 if there is rash progression (i.e., appearance of new/additional lesions if originally less than three lesions present, or appearance of vesicles if originally only papules present). When the subject returns for repeat sample collection, if possible, three samples from separate lesions should be collected. Refer to the SPM for further details on sample collection.

At Visit HZ-1, the rash will be documented by digital photography. Additional photographs of HZ lesions may be taken after Visit HZ-1 to help note the progression of the rash.

5. STUDY COHORT

5.1. Number of subjects/centres

Subjects who received at least 1 dose of HZ/su vaccine in study ZOSTER-006 or study ZOSTER-022, and confirmed their interest to enrol in the study at participating centres, will be considered for entry in study ZOSTER-049. Refer to Sections 5.2 and 5.3 for inclusion/exclusion criteria, respectively.

The subsets foreseen for HI analysis and for CMI analyses as well as the 1-Additional Dose, Revaccination and Control groups are described in Table 4. Refer to Section 11.4 for details regarding the estimation of sample size for the LTFU study group.

Table 4 Subsets

Subset name	Description	Estimated maximum number of subjects
All subjects	All subjects entering the study will have a HI blood sample (approximately 5 mL) at Visit Month 0. For subjects in the LTFU group, who do not belong to any subset, these samples will be stored and tested for HI only if the subject develops HZ during the ZOSTER-049 study or if there are other reasons requiring the HI testing of these samples.	≤ 14448
LTFU Group HI subset	Subjects in the LTFU group who were in the immunogenicity subset during studies ZOSTER-006/022 and continue participation in this study. Blood samples (approximately 5 mL) will be collected and tested from Visit Month 0 to Visit Year 6, on a yearly basis, to assess HI responses.	≤ 1740
LTFU Group CMI subset	Subjects in the LTFU group who were in the CMI subset during study ZOSTER-006 and continue participation in this study. Blood samples (approximately 20 mL) will be collected and tested from Visit Month 0 to Visit Year 6 on a yearly basis to assess CMI responses.	≤ 234
HZ subset	Subjects who developed confirmed HZ during ZOSTER-006 or ZOSTER-022, or who develop HZ during the interval between the end of the ZOSTER-006/022 and the beginning of ZOSTER-049, or who develop suspected HZ during ZOSTER-049 will be part of the HZ subset.<br>Subjects who develop HZ at any time after enrolment in ZOSTER-006 or ZOSTER-022 and who are already part of the HI subset in ZOSTER-006 or ZOSTER-022 will provide blood samples to assess HI responses (approximately 5 mL) from Visit Month 0 to Visit Year 6 irrespective of when the HZ episode occurs. If these subjects are part of the ZOSTER-006 CMI subset, then they will also continue to provide blood to assess CMI responses (approximately 20 mL).<br>Subjects who develop HZ at any time after enrolment in ZOSTER-006 or ZOSTER-022 and who were NOT part of the HI or CMI subsets in these studies, will provide blood samples to assess HI responses (approximately 5 mL) beginning at the annual visit in the ZOSTER-049 study subsequent to the occurrence of the HZ episode. The blood sample taken upon enrolment at Visit Month 0 will be tested and included in the analyses for these subjects.	TBD

Subset name	Description	Estimated maximum number of subjects
1-Additional Dose Group	Subjects to be administered HZ/su vaccine on a 1-dose schedule at Visit Month 0. Blood samples (approximately 5 and 20 mL) will be collected at Visit Month 0, Visit Month 1, and from Visit Year 1 to Visit Year 6 on a yearly basis to assess HI and CMI responses.	60
Revaccination Group	Subjects to be administered HZ/su vaccine on a 2-dose schedule at Visit Month 0 and Visit Month 2. Blood samples (approximately 5 and 20 mL) will be collected at Visit Month 0, Visit Month 1, Visit Month 3 and from Visit Year 1 to Visit Year 6 on a yearly basis to assess HI and CMI responses.	60
Control Group	Subjects in the non-vaccinated control group. Blood samples (approximately 5 and 20 mL) will be collected at Visit Month 0, Visit Month 1, Visit Month 3 and from Visit Year 1 to Visit Year 6 on a yearly basis to assess HI and CMI responses.	120

HI = Humoral Immunogenicity; mL = Millilitre; LTFU = Long-Term Follow-Up; CMI = Cell-Mediated Immunity; HZ= Herpes Zoster; TBD = To Be Determined.

Overview of the recruitment plan:

Potentially eligible subjects from centres who participated and received HZ/su vaccine in ZOSTER-006/022 studies will be contacted and considered for entry in the ZOSTER-049 study. The rationale for not enrolling any of these subjects will be recorded in the site's screening log. This study is planned to be conducted in multiple countries worldwide at centres which participated in the ZOSTER-006/022 studies.

5.2. Inclusion criteria for enrolment

Deviations from inclusion criteria are not allowed because they can potentially jeopardise the scientific integrity of the study, regulatory acceptability or subject safety. Therefore, adherence to the criteria as specified in the protocol is essential.

All subjects must satisfy ALL the following criteria at study entry:

- Subjects who, in the opinion of the investigator, can and will comply with the requirements of the protocol (e.g., completion of the diary cards, return for follow-up visits, ability to have scheduled contacts to allow evaluation during the study). Or subjects with a caregiver who, in the opinion of the investigator, can and will comply with the requirements of the protocol (e.g., completion of the diary cards, availability for follow-up contacts);
- Written informed consent obtained from the subject prior to performance of any study specific procedure;
- Subject who participated in ZOSTER-006 or ZOSTER-022 studies and received at least one dose of HZ/su vaccine.[1]

[1] HZ/su vaccine is referred to as $gE/AS01_B$ vaccine in ZOSTER-006/022 protocols.

Note: Subjects from the immunogenicity subset of studies ZOSTER-006/022 are not allocated to the 1-Additional Dose, Revaccination and Control groups. Subjects in these three groups will be enrolled amongst subjects who received 2 doses of the HZ/su vaccine and were part of the ATP cohort for analysis of efficacy in the previous studies ZOSTER-006/022. In order not to confound immune responses, subjects who developed HZ prior to enrolment in the ZOSTER-049 study (confirmed HZ during the ZOSTER-006/022 studies or suspected HZ during the interval between the end of the ZOSTER-006/022 studies and beginning of the ZOSTER-049 study), will not be enrolled in the 1-Additional Dose, Revaccination and Control groups (Refer to Table 4 and Section 6.1.2).

Additional inclusion criteria for the 1-Additional Dose, Revaccination and Control groups, ONLY:

- Female subjects of non-childbearing potential may be enrolled.
  - Non-childbearing potential is defined as pre-menarche, current tubal ligation, hysterectomy, ovariectomy or post-menopause.

Please refer to the glossary of terms for the definition of menarche and menopause.

- Female subjects of childbearing potential may be enrolled if the subject:
  - has practiced adequate contraception for 30 days prior to vaccination, and
  - has a negative pregnancy test on the day of vaccination and
  - has agreed to continue adequate contraception during the entire treatment period and for 2 months after completion of the vaccination series.

Please refer to the glossary of terms for the definition of adequate contraception.

5.3. Exclusion criteria for enrolment

Deviations from exclusion criteria are not allowed because they can potentially jeopardise the scientific integrity of the study, regulatory acceptability or subject safety. Therefore, adherence to the criteria as specified in the protocol is essential.

The following criteria should be checked at the time of study entry. If ANY exclusion criterion applies, the subject must not be included in the study:

- Use of any investigational or non-registered product (pharmaceutical product or device) at the time of enrolment or planned use during the study period;
- Previous vaccination against VZV or HZ and/or planned administration during the study of a VZV or HZ vaccine (including an investigational or non-registered vaccine other than the HZ/su vaccine administered in studies ZOSTER-006/022);
- Chronic administration (defined as > 14 consecutive days in total) of immunosuppressants or other immune-modifying drugs during the period starting six months prior to Visit Month 0 of study ZOSTER-049 or expected administration at any time during the study period. For corticosteroids, this will mean prednisone ≥ 20 mg/day or equivalent. A prednisone dose of < 20 mg/day is allowed. Inhaled, topical and intra-articular corticosteroids are allowed;

- Administration of long-acting immune-modifying drugs (e.g., infliximab, rituximab) within 6 months prior to Visit Month 0 of study ZOSTER-049 or expected administration at any time during the study period;
- Any confirmed or suspected immunosuppressive or immunodeficient condition resulting from disease (e.g., malignancy, human immunodeficiency virus [HIV] infection) or immunosuppressive/cytotoxic therapy (e.g., medications used during cancer chemotherapy, organ transplantation or to treat autoimmune disorders);
- Administration of immunoglobulins and/or any blood products within 3 months prior to Visit Month 0 of study ZOSTER-049 or planned administration during the study period;
- Prolonged use (> 14 consecutive days) of oral and/or parenteral antiviral agents that are active against VZV (acyclovir, valacyclovir, famciclovir, etc.) and planned to be used during the study period for an indication other than to treat suspected or confirmed HZ or an HZ-related complication (topical use of these antiviral agents is allowed).
- Important underlying illness that in the opinion of the investigator would be expected to interfere significantly during the study;

Additional exclusion criteria for the 1-Additional Dose, Revaccination and Control groups, ONLY:

- Subjects who experienced an SAE from first vaccination in the previous ZOSTER-006/022 studies to enrolment in study ZOSTER-049 that was considered related to study vaccine by either the investigator or the sponsor;
- Subjects with a new onset of a pIMD or exacerbation of a pIMD from first vaccination in the previous ZOSTER-006/022 studies to enrolment in study ZOSTER-049;
- Use of any investigational or non-registered product (pharmaceutical product or device) within 30 days preceding the first dose of study vaccine or planned use during the study period;
- Administration or planned administration of any other immunizations within 30 days before the first study vaccination or scheduled within 30 days after study vaccination. However, licensed non-replicating vaccines (i.e., inactivated and subunit vaccines, including inactivated and subunit influenza vaccines for seasonal or pandemic flu, with or without adjuvant) may be administered up to 8 days prior to each dose and/or at least 14 days after any dose of study vaccine;
- History of allergic disease or reactions likely to be exacerbated by any component of the vaccine. Additionally, consider allergic reactions to other material or equipment related to study participation (such as materials that may possibly contain latex - gloves, syringes, etc.). Please note, the vaccine and vials in this study do not contain latex;
- Pregnant or lactating female;
- Female planning to become pregnant or planning to discontinue contraceptive precautions (if of childbearing potential);
- Previous episode/history of HZ.

6. CONDUCT OF THE STUDY

6.1. Regulatory and ethical considerations, including the informed consent process

The study will be conducted in accordance with all applicable regulatory requirements.

The study will be conducted in accordance with the ICH Guideline for Good Clinical Practice (GCP), all applicable subject privacy requirements and the guiding principles of the Declaration of Helsinki.

The study has been designed and will be conducted in accordance with the ICH Harmonised Tripartite Guideline for clinical investigation of medicinal products in the paediatric population (ICH E11) and all other applicable ethical guidelines.

GSK will obtain favourable opinion/approval to conduct the study from the appropriate regulatory agency, in accordance with applicable regulatory requirements, prior to a site initiating the study in that country.

Conduct of the study includes, but is not limited to, the following:

- Institutional Review Board (IRB)/Independent Ethics Committee (IEC) review and favourable opinion/approval of study protocol and any subsequent amendments.
- Subject informed consent and subject informed assent, as appropriate.
- Investigator reporting requirements as stated in the protocol.

GSK will provide full details of the above procedures to the investigator, either verbally, in writing, or both.

Freely given and written or witnessed/ thumb printed informed consent must be obtained from each subject, as appropriate, prior to participation in the study.

GSK Biologicals will prepare a model Informed Consent Form (ICF) which will embody the ICH GCP and GSK Biologicals required elements. While it is strongly recommended that this model ICF is to be followed as closely as possible, the informed consent requirements given in this document are not intended to pre-empt any local regulations which require additional information to be disclosed for informed consent to be legally effective. Clinical judgement, local regulations and requirements should guide the final structure and content of the local version of the ICF.

The investigator has the final responsibility for the final presentation of the ICF, respecting the mandatory requirements of local regulations. The ICF generated by the investigator with the assistance of the sponsor’s representative must be acceptable to GSK Biologicals and be approved (along with the protocol, and any other necessary documentation) by the IRB/IEC.

6.1.1. Subject identification and randomisation of treatment

The original subject identification numbers used in the primary studies ZOSTER-006/022 (containing up to 5 digits) will be used in the ZOSTER-049 study and they will be preceded by an additional sixth digit: a number 6 for the subjects initially enrolled in ZOSTER-006 and a number 2 for the subjects initially enrolled in ZOSTER-022. E.g., if two subjects, one from ZOSTER-006 and the other from ZOSTER-022 both with subject identification number 111 are to be enrolled in ZOSTER-049, they will be attributed the following identification numbers:

- 600111 for the subject initially enrolled in ZOSTER-006;
- 200111 for the subject initially enrolled in ZOSTER-022.

Subject numbers will be pre-loaded into a centralised randomisation system on the internet (SBIR) that will be used for subject number assignment. Refer to the SPM for further details on the assignment of subject identification numbers.

6.1.1.1. Randomisation of supplies

For the 1-Additional Dose, Revaccination and Control groups:

The randomisation within blocks will be performed at GSK Biologicals, using MATerial EXcellence (MATEX), a program developed for use in Statistical Analysis System (SAS®) (Cary, NC, USA) by GSK Biologicals. Entire blocks of supplies will be shipped to the study centres /warehouse(s).

For countries/centres that will enrol subjects in the 1-Additional Dose, Revaccination and Control groups an over-randomisation of supplies will be prepared in order to take advantage of greater rates of recruitment in one or more centres and thus reduce the overall recruitment period in these groups.

6.1.1.2. Randomisation of treatment

6.1.1.2.1. Treatment allocation to the subject for the 1-Additional Dose, Revaccination and Control groups

The target will be to enrol approximately 240 eligible subjects who will be randomly assigned to the 3 study groups in a (1:1:2) ratio (approximately 60 subjects in each group 1-Additional Dose and Revaccination and 120 subjects in group Control). Allocation of a subject to a study group at the investigator site will be performed using SBIR.

The treatment numbers will be allocated by dose. The randomisation algorithm will use a minimisation procedure accounting for age at primary vaccination (50-59, 60-69 and ≥ 70 YOA) at the time of first vaccination in the ZOSTER-006/022 studies and country. All factors will have equal weight in the minimisation algorithm.

After obtaining the signed and dated ICF from the subject and having checked the eligibility of the subject, the study staff in charge of the vaccine administration will access SBIR. The randomisation system will determine the study group and will provide the treatment number to be used for the first dose. Note that the treatment number issued for the Control group will not be used for administration.

The number of each administered treatment must be recorded in the eCRF on the Vaccine Administration screen.

When SBIR is not available, please refer to the SBIR user guide or the SPM for specific instructions.

6.1.1.2.2. Treatment number allocation for subsequent doses

For each dose subsequent to the first dose, the study staff in charge of the vaccine administration will access SBIR, provide the subject identification number, and the system will provide a treatment number.

The number of each administered treatment must be recorded in the eCRF on the Vaccine Administration screen.

6.1.2. Allocation of subjects to assay subsets

Subjects who were part of the immunogenicity subset in the ZOSTER-006/022 studies will be allocated to the HI subset of the LTFU group in the ZOSTER-049 study. From this HI subset, subjects will be allocated to the CMI subset of the LTFU group if they were part of the CMI subset in the ZOSTER-006 study.

Subjects who developed HZ episode(s) during the ZOSTER-006/022 studies (confirmed HZ) and/or during the interval between the end of the ZOSTER-006/022 studies and beginning of ZOSTER-049 study and/or during the ZOSTER-049 study (suspected HZ) will be included in the HZ subset.

Subjects who were part of the immunogenicity subset in the ZOSTER-006/022 studies will be excluded from allocation to the three groups 1-Additional Dose, Revaccination and Control. Subjects can be allocated to the three groups 1-Additional Dose, Revaccination and Control if they:

- received 2 doses of HZ/su vaccine in the ZOSTER-006/022 studies;
- were NOT part of the immunogenicity and CMI subsets in the ZOSTER-006/022 studies;
- were part of the ATP cohort for analyses of efficacy in the ZOSTER-006/022 studies;
- did NOT develop HZ (confirmed or suspected HZ) prior to enrolment in this ZOSTER-049 study.
- and comply with inclusion and exclusion criteria (see Sections 5.2 and 5.3, respectively) for the 1-Additional Dose, Revaccination and Control groups.

As there is a requirement to collect CMI samples for subjects enrolled in the 1-Additional Dose, Revaccination and Control groups, these subjects will be enrolled in selected countries and sites that have access to a facility for CMI sample processing.

Refer to Table 4 for a description of subsets and to Table 19 for a schedule of immunological sampling and read-outs.

6.2. Method of blinding

This is an open-label study with two treatment groups (1-Additional Dose and Revaccination).

The laboratory in charge of the laboratory testing will be blinded to the treatment, and codes will be used to link the subject and study (without any link to the treatment attributed to the subject) to each sample.

6.3. General study aspects

Supplementary study conduct information not mandated to be present in this protocol is provided in the accompanying SPM. The SPM provides the investigator and the site personnel with administrative and detailed technical information that does not impact the safety of the subjects.

6.3.1. Data collection

6.3.1.1. Monthly contacts

After Visit Month 0, monthly contacts between the subjects/ subject's caregiver and the investigator and/or his delegate will take place to collect information on any event of interest that may have occurred [see Section 6.5.7 for details]. The contacts will not take place at months that coincide with the subject's scheduled study visits. Also, subjects with suspected HZ will be contacted periodically as outlined in Table 7. The contacts will take place using the most convenient method suited for the sites (e.g., telephone calls by site staff or designee, or Short Message Service (SMS) text messages through a call centre, or visit by the study staff to the subject's home). A guidance document outlining the information that needs to be collected at each contact will be provided to each country, and will serve as a guidance to develop the local script (Refer to the SPM). The logistic details on the set-up of the contacts will be documented by each site/country. At each contact, the subject/ subject's caregiver will respond to a standard set of questions in a language that is understandable to them. The investigator and/or his delegate will transcribe the relevant information on any event of interest in the appropriate of the subject's eCRF, in English. In case of ongoing HZ, subjects / subjects' caregiver will also be reminded to complete Zoster Brief Pain Inventory (ZBPI) questionnaires as described in Section 4.5.2.3.

6.3.1.2. Diary cards and questionnaires

The diary cards and/or questionnaires to be completed by the subject/ subject's caregiver will be distributed and explained by the investigator or his/her delegate. Any supplied diary cards or questionnaires should be preferably completed by the subject themselves. In case of difficulty in self-completion of the diary cards or questionnaires, an aide (such as a family member or caregiver who is not involved in the study) may provide assistance with reading the questions (verbatim) and/or transcribing the subject's responses on the questionnaires and/or diary cards.

For subjects in the two groups (1-Additional Dose and Revaccination, only), 7-day and 30-day diary cards will be dispensed on the day of vaccination to be completed by the subjects/ subjects' caregiver. The 7-day diary cards will be completed for solicited AEs (from Day 0 to Day 6 after each vaccination) and the 30-day diary cards will be completed by for unsolicited AEs (from Day 0 to Day 29 after each vaccination) and any concomitant medication and vaccination taken from Day 0 to Day 29 after each vaccination (see Table 6 and Table 26).

When the completed diary cards and/or questionnaires are returned to the study staff, the study staff will ask the subject (at the time of return or at subsequent contact) if he/she received any assistance in completing diary cards or questionnaires. If the subject had assistance completing the diary card and/or questionnaires (e.g., by a caregiver), it should be noted in the eCRF. In case questionnaires are completed at the study site, study staff can assist in reading the questions (verbatim).

6.4. Outline of study procedures

Table 5 presents the list study of procedures for the LTFU group.

Table 6 presents the list study of procedures for the three groups 1-Additional Dose, Revaccination and Control.

Table 7 presents follow-up procedures to be performed for each suspected HZ case.

Table 5 List of study procedures (for the LTFU group)

Epoch	LONG-TERM FOLLOW-UP			
Type of contact	Visit MONTH 0	Monthly contacts [1]	Visit/ Contact YEAR 1, 2, 3, 4, 5 [2]	Study conclusion Visit/ Contact [3] Year 6 [2]
Timepoints	Day 0/ Month 0		Month 12, 24, 36, 48, 60	Month 72
Sampling timepoints	Post-Primary		Post-Primary	Post-Primary
Informed consent	●			
Check inclusion criteria	●			
Check exclusion criteria	●			
Medical history including HZ history (see Section 6.5.3)	●			
Physical examination (see Section 6.5.4)	O			
Training on self-reporting by subjects [4]	O		O	
Blood sampling (approximately 5 mL) for all LTFU non-subset subjects (Table 4 and Section 6.5.8.1)	●			
Blood sampling (approximately 5 mL) for HI Ab determination (Table 4 and Section 6.5.8.1)	●		●	●
Blood sampling (approximately 20 mL) for CMI responses (Table 4 and Section 6.5.8.1)	●		●	●
Blood sampling (approximately 5 mL) for Ab determination for the HZ subset (Table 4 and Section 6.5.8.1	●		●	●
Dispensing of HZ-specific diary cards to all subjects	O			
Recording of IMCs (Section 7.7)	●	●	●	●
Reporting of SAEs related to investigational vaccine (Table 25)	●	●	●	●
Recording of SAEs related to study participation or to concurrent GSK medication/vaccine (Table 25)	●	●	●	●
Follow-up of HZ (Section 4)	●	●	●	●
Recording of concomitant medication/vaccination by study staff/investigator (Section 7.6.2)	●	●	●	●
E-signature at year 2 and year 4 (for the year 2 and year 4 analyses)			● [5]	
Study conclusion (e signature)				●

● is used to indicate a study procedure that requires documentation in the individual eCRF.
O is used to indicate a study procedure that does not require documentation in the individual eCRF.
Post-Primary = Post-primary vaccination with HZ/su in studies ZOSTER-006/022; Ab = Antibody; HI = Humoral Immunogenicity; CMI = Cell-Mediated Immunity; mL = Millilitre; HZ = Herpes Zoster; IMC = Intercurrent Medical Conditions; SAE = Serious Adverse Event; GSK = GlaxoSmithKline.

[1] After Visit Month 0, monthly phone contacts between the subjects and the investigator and/or his delegate will be scheduled to collect information on safety, on the occurrence of HZ, and to follow-up ongoing HZ cases (see Section 6.3.1.1). Monthly contacts will occur after Visit Month 0 until the subject's last visit, except at months that coincide with the subject's scheduled visits.

[2] The study procedures applicable for the Visit Year 1 to Visit Year 6 are identical (except Visit Year 6 is the Study Conclusion).

[3] Refer to Section 6.5.11.

[4] Subjects/ subject's caregiver will be instructed to contact their study site immediately if he/she develops any symptoms suggestive of HZ and/or, if he/she manifests any symptoms he/she perceives as serious.

[5] E-sign off at year 2 and year 4 for the year 2 and year 4 analyses.

Table 6 List of study procedures (for the 1-Additional Dose, Revaccination and Control groups)

Epoch	LONG-TERM FOLLOW-UP						
Type of contact	Visit MONTH 0	Visit Month 1	Visit Month 2†	Visit Month 3‡	Monthly contacts [a]	Visit YEAR 1, 2, 3, 4, 5 [b]	Study conclusion Visit [c] Year 6 [b]
Timepoints	Day 0/ Month 0	Month 1	Month 2 †	Month 3 ‡		Month 12, 24, 36, 48, 60	Month 72
Sampling timepoints	Pre-Vacc	Post-Vacc 1		Post-Vacc 2		Post-Vacc 2	Post-Vacc 2
Informed consent	●						
Check inclusion criteria	●						
Check exclusion criteria	●						
Medical history including HZ history (see Section 6.5.3)	●						
Physical examination (see Section 6.5.4)	O						
Pregnancy test if applicable [d] (see Section 6.5.5)	●		●				
Check contraindications (see Section 6.5.6)	O		O				
Training on self-reporting by subjects [e]	O	O	O	O		O	
Randomisation	O						
Pre-vaccination body temperature [f]	●		●				
Blood sampling (approximately 5 mL) for HI Ab determination (Table 4 and Section 6.5.8.1)	●	●		●		●	●
Blood sampling (approximately 20 mL) for CMI responses (Table 4 and Section 6.5.8.1)	●	●		●		●	●
Recording of treatment number	●		●				
Vaccination [f]	●		●				
Dispensing of HZ-specific diary cards to all subjects	O						
Reporting of medically attended visits until 6 months after Visit Month 0 or last HZ/su vaccination (see Section 9.1.5 and Table 26) [g]	●	●	●	●	●		
Reporting of pIMDs according to guidelines in Section 9.1.6.1 (see Table 26) [h]	●	●	●	●	●	●	
Reporting of pIMD related to investigational vaccine	●	●	●	●	●	●	●
Reporting of pregnancy [f] (see Table 26)	●	●	●	●	●	●	●
Recording of IMCs (see Section 7.7)	●	●	●	●	●	●	●
Reporting of all SAEs (Table 26) [h]	●	●	●	●	●	●	

Epoch	LONG-TERM FOLLOW-UP						
Type of contact	Visit MONTH 0	Visit Month 1	Visit Month 2†	Visit Month 3‡	Monthly contacts [a]	Visit YEAR 1, 2, 3, 4, 5 [b]	Study conclusion Visit [c] Year 6 [b]
Timepoints	Day 0/ Month 0	Month 1	Month 2 †	Month 3 ‡		Month 12, 24, 36, 48, 60	Month 72
Sampling timepoints	Pre-Vacc	Post-Vacc 1		Post-Vacc 2		Post-Vacc 2	Post-Vacc 2
Reporting of SAEs related to investigational vaccine (Table 26)	●	●	●	●	●	●	●
Recording of SAEs related to study participation or to concurrent GSK medication/vaccine (Table 26)	●	●	●	●	●	●	●
Follow-up of HZ (see Section 4 and Table 26)	●	●	●	●	●	●	●
Recording of concomitant medication/vaccination by study staff/investigator (Section 7.6.2)	●	●	●	●	●	●	●
Dispensing of 7-day diary cards for solicited AEs to the 7-day diary card and 30-day diary cards for unsolicited AEs and concomitant medication/vaccination to all vaccinated subjects [f]	O		O				
Daily post-vaccination recording by subjects of solicited symptoms (Days 0 - 6 after each vaccination) on the 7-day diary card by all vaccinated subjects [f] (see Table 26)	O		O				
Daily post-vaccination recording of unsolicited symptoms (Days 0 - 29 after each vaccination), and concomitant medication/ vaccination (Days 0 - 29 after each vaccination) on the 30-day diary card by all vaccinated subjects [f] (see Table 26)	O		O				
Returning by subjects of 7-day diary cards for solicited symptoms and 30-day diary cards for unsolicited AEs and concomitant medication and vaccination [f]		O		O			
Transcription of 7-day diary cards for solicited symptoms and 30-day diary cards for unsolicited AEs and concomitant medication and vaccination by study staff/investigator [f]		●		●			
E-signature at Month 3 (for the Month 3 analysis)				●			
E-signature at year 2 and year 4 (for the year 2 and year 4 analyses for the Control group, only)						● [i]	
Study conclusion (e signature)							●

● is used to indicate a study procedure that requires documentation in the individual eCRF.
O is used to indicate a study procedure that does not require documentation in the individual eCRF.

Pre-Vacc = Pre Vaccination; Post Vacc 1 = Post Vaccination 1; Post Vacc 2 = Post Vaccination 2; Ab = Antibody; HI = Humoral Immunogenicity; CMI = Cell-Mediated Immunity; mL = Millilitre; pIMDs = potential Immune-Mediated Diseases; IMC = Intercurrent Medical Conditions; SAE = Serious Adverse Event; GSK = GlaxoSmithKline; HZ = Herpes Zoster.

† Visit Month 2 for Vacc 2 is only for the Revaccination group.

‡ Visit Month 3 for blood sample is only for the Revaccination and Control groups.

a After Visit Month 3 (After Visit Month 1 for the 1-Additional Dose group), monthly phone contacts between the subjects and the investigator and/or his delegate will be scheduled for the subject to respond to a standard set of questions, in a language that is understandable to the subject, to collect information on safety, on the occurrence of HZ, and to follow-up ongoing HZ cases (see Section 6.3.1.1). The Control group will also have a phone contact at Visit Month 2. Monthly contacts will occur after Visit Month 3 until the subject's last visit, except at months that coincide with the subject's scheduled visits.

[b] The study procedures applicable for the Visit Year 1 to Visit Year 6 are identical (except Visit Year 6 is the Study Conclusion).

[c] Refer to Section 6.5.11.

[d] Only applicable for females of childbearing potential. A urine pregnancy test is sufficient. A serum pregnancy test instead of a urine pregnancy test should only be considered if required by country, local or ethics committee regulations (see Section 6.5.5).

[e] Subjects/ subject's caregiver will be instructed to contact their study site immediately if he/she develops any symptoms suggestive of HZ and/or, if he/she manifests any symptoms he/she perceives as serious.

[f] A study procedure only for the two groups vaccinated with 1 or 2 additional doses of HZ/su vaccine (1-Additional Dose and Revaccination).

[g] The reporting of medically attended visits will be from Visit Month 0 until 6 months (for the Control and 1-Additional Dose groups) and from Visit Month 0 until 6 months after last HZ/su vaccination (for the Revaccination group) (see Section 9.1.5 and Table 26).

[h] The reporting of all SAEs and pIMDs will be from Visit Month 0 until 12 months (for Control and 1-Additional Dose groups) and from Visit Month 0 until 12 months after last HZ/su vaccination (for the Revaccination group) (see Table 26).

[i] E-sign off at year 2 and year 4 for the year 2 and year 4 analyses (for the Control group, only).

Table 7 Study procedures to be performed during the follow-up period for each suspected HZ case

	VISITS/CONTACTS IN CASE OF HZ						
**Type of contact**	**Visit HZ-1**	**Visit HZ-2**	**Contact HZ-3**	**Contact HZ-4**	**Visit HZ-5**	**Contact HZ-6**	**Visit HZ-7**
**Timepoints**	**Day HZ-0**	**Day HZ-7**	**Day HZ-14**	**Day HZ-21**	**Day HZ-28**	**Day HZ-56**	**Day HZ-91†**
Perform clinical examination	O						
Return HZ-specific diary cards to study staff/investigator	O						
Transcription of the HZ-specific diary card by study staff/investigator	●						
Take digital photographs of HZ rash $^{\alpha}$	●						
Recording of the HZ onset date by study staff/investigator	●						
Collect HZ lesion samples (3 replicate samples) for confirmation by PCR of a case of clinically diagnosed suspected HZ as specified in Section 4.5.3 *	●						
Record relevant information regarding HZ in eCRF by study staff/investigator	●	●	●	●	●	●	●
Record concomitant medication/vaccination according to guidelines in Section 7.6	●	●	●	●	●	●	●
Record IMCs according to guidelines in Section 7.7	●	●	●	●	●	●	●
Record any medical attention received for HZ or any HZ-related complication	●	●	●	●	●	●	●
Dispense ZBPI questionnaires to subjects ‡	O						
Completion of ZBPI questionnaires by the subjects until pain ceases or LSLV/C (ZBPI pain data will be collected until at least Day HZ-91) †	O	O	O	O	O	O	O
Return completed ZBPI questionnaires to study staff/investigator according to instructions provided by the investigator/study staff to subjects		O	O	O	O	O	O
Transcription of ZBPI questionnaires by study staff/investigator	●	●	●	●	●	●	●

● is used to indicate a study procedure that requires documentation in the individual eCRF.
O is used to indicate a study procedure that does not require documentation in the individual eCRF.
HZ = Herpes Zoster; PCR = Polymerase Chain Reaction; eCRF = electronic Case Report Form; IMC = Intercurrent Medical Condition; ZBPI = Zoster Brief Pain Inventory; LSLV/C = Last Subject Last Visit/Contact.

Note: If HZ-associated pain ceases (defined as a 28-day [or 4-week] pain free period) and the HZ rash resolves, subsequent HZ follow-up visits or contacts will be cancelled. If pain reappears in the same area after a 4-week pain-free period and is not accompanied by a new HZ rash, it will be assigned to the previous HZ-episode. Visits/contacts will restart with Day HZ-0 defined as the first visit of the assigned episode, prior to the pain free period.

$^{\alpha}$ Additional photographs of HZ lesions may be taken after Visit HZ-1 to help note the progression of the rash.

* If during clinical evaluation at Visit HZ-1, the investigator determines that adequate rash samples are not present at Visit HZ-1 (i.e., <3 lesions present or only papules present), the investigator has the option of collecting three additional samples prior to or at Visit HZ-2 (preferably within 7 days). When the subject returns to repeat sample collection, if possible, 3 samples from separate lesions should be collected.

‡ The study staff/investigator will dispense additional questionnaires and provide instructions for the subject to return the completed questionnaires to the study site. The subjects will be given a new supply of questionnaires as necessary.

† Subjects with suspected HZ will be asked to complete the ZBPI questionnaire at Day HZ-0 (Visit HZ-1) to rate HZ-associated pain within the last 24 hours (If the time between the HZ onset and clinical evaluation at Visit HZ-1 is greater than 24 hours, the subject will be asked to complete a second ZBPI also for the elapsed time between the HZ onset and 24 hours before Visit HZ-1); daily from Day HZ- 1 to Day HZ-28, and weekly from Day HZ-29 onwards until a 4-week pain-free period is documented. If pain reappears in the same area after a 4-week pain-free period and is not accompanied by a new HZ rash, it will be assigned to the previous HZ-episode. The completion of ZBPI questionnaires will resume based upon the weekly schedule established at the start of the assigned episode. For all subjects with ongoing HZ-associated pain at the time of the end of study, ZBPI data will be collected until a 4-week pain-free period is documented OR until at least Day HZ-91 (See Section 4.5.2.3).

Time intervals between the yearly study visits/contacts related to study procedures performed on subjects are presented in Table 8 for the LTFU group, in Table 9 for the 1-Additional Dose and Control groups and in Table 10 for the Revaccination group. In addition; between Visit Month 0 and the last yearly visit, monthly contacts between the subjects and the investigator and/or his delegate will be scheduled. These monthly contacts will not take place if coinciding with the yearly study visits. Intervals of 25 – 45 days are recommended between the monthly contacts.

Table 8 Intervals between study visits for the LTFU group

Interval between Visits/ Contacts	Optimal length of interval (in months) [1]	Allowed interval (range in days) [2]
Visit Month 0 → Visit/ Contact Year 1	12 months	335 – 395
Visit Month 0 → Visit/ Contact Year 2	24 months	700 – 760
Visit Month 0 → Visit/ Contact Year 3	36 months	1065 – 1125
Visit Month 0 → Visit/ Contact Year 4	48 months	1430 – 1490
Visit Month 0 → Visit/ Contact Year 5	60 months	1795 – 1855
Visit Month 0 → Visit/ Contact Year 6 [3]	72 months	2160 – 2220

[1] Whenever possible the investigator should arrange study visits/contacts within this interval.
[2] Subjects will not be eliminated from the ATP cohort for analysis if they make the study visit outside this interval.
[3] For subjects in HZ follow-up at the time of their last yearly visit, their study conclusion will coincide with their last Day HZ-91 visit (see Table 11).

Table 9 Intervals between study visits for the 1-Additional Dose and Control groups

Interval between Visits	Optimal length of interval (in months) [1]	Allowed interval (range in days)
Visit Month 0 → Visit Month 1	1 month	28 – 48 2
Visit Month 0 → Visit Month 3 *	3 months	75 – 105 [3]
Visit Month 0 → Visit Year 1	12 months	335 – 395 [3]
Visit Month 0 → Visit Year 2	24 months	700 – 760 [3]
Visit Month 0 → Visit Year 3	36 months	1065 – 1125 [3]
Visit Month 0 → Visit Year 4	48 months	1430 – 1490 [3]
Visit Month 0 → Visit Year 5	60 months	1795 – 1855 [3]
Visit Month 0 → Visit Year 6 4	72 months	2160 – 2220 3

* Interval ONLY for the Control group.
[1] Whenever possible the investigator should arrange study visits within this interval.
[2] Subjects may not be eligible for inclusion in the ATP cohort for analysis of immunogenicity if they make the study visit outside this interval.
[3] Subjects may not be eliminated from the ATP cohort for analysis if they make the study visit outside this interval.
[4] For subjects in HZ follow-up at the time of their last yearly visit, their Study conclusion will coincide with their last Day HZ-91 visit (see Table 11).

Table 10 Intervals between study visits for the Revaccination group

Interval between Visits	Optimal length of interval (in months) [1]	Allowed interval (range in days)
Visit Month 0 → Visit Month 1	1 month	28 – 48 [3]
Visit Month 0 → Visit Month 2	2 months	49 – 83 [2]
Visit Month 2 → Visit Month 3	1 month	28 – 48 [2]
Visit Month 2 → Visit Year 1	12 months	335 – 395 [3]
Visit Month 2 → Visit Year 2	24 months	700 – 760 [3]
Visit Month 2 → Visit Year 3	36 months	1065 – 1125 [3]
Visit Month 2 → Visit Year 4	48 months	1430 – 1490 [3]
Visit Month 2 → Visit Year 5	60 months	1795 – 1855 [3]
Visit Month 2 → Visit Year 6 [4]	72 months	2160 – 2220 [3]

[1] Whenever possible the investigator should arrange study visits within this interval.
[2] Subjects may not be eligible for inclusion in the ATP cohort for analysis of immunogenicity if they make the study visit outside this interval.
[3] Subjects may not be eliminated from the ATP cohort for analysis if they make the study visit outside this interval.
[4] For subjects in HZ follow-up at the time of their last yearly visit, their Study conclusion will coincide with their last Day HZ-91 visit (see Table 11).

Time intervals between study visits/contacts to be performed for follow-up of HZ are presented in Table 11.

Table 11 Intervals between visits/contacts for subjects in case of suspected HZ

Interval between Visits/ Contacts	Length of interval	Optimal Timing of contact (range of days)
Visit HZ-1 (Day HZ-0) → Visit HZ-2 (Day HZ-7)	7 days	Day HZ-7 (+/- 3 days)*
Visit HZ-2 (Day HZ-7) → Contact HZ-3 (Day HZ-14)	7 days	Day HZ-14 (+/- 3 days)*
Contact HZ-3 (Day HZ-14) → Contact HZ-4 (Day HZ-21)	7 days	Day HZ-21 (+/- 3 days)*
Contact HZ-4 (Day HZ-21) → Visit HZ-5 (Day HZ-28)	7 days	Day HZ-28 (+/- 3 days)*
Visit HZ-5 (Day HZ-28) → Contact HZ-6 (Day HZ-56)	28 days	Day HZ-56 (+/- 7 days)*
Visit HZ-1 (Day HZ-0) → Visit HZ-7 (Day HZ-91)	91 days	Day HZ-91 (+ 7 days)

Note: The date of the previous visit/contact is used as reference date to define the interval between the subsequent study visits/contacts.
Note: If HZ-associated pain ceases (i.e., after a 4-week pain-free period is documented) and the HZ rash resolves, subsequent follow-up HZ visits or contacts will be cancelled (see Section 4.5.2). Follow-up of HZ-associated pain persisting beyond Visit HZ-7 (Day HZ-91) or other complications will be done at monthly contacts between the subject and the investigator and/or investigator's delegate.
* If contacted early in the window, then remaining days in the interval will need to be captured with the next contact.

Refer to Section 6.6 for study procedures to be considered during special circumstances (Amended 11 May 2020).

6.5. Detailed description of study procedures

6.5.1. Informed consent

The signed/witnessed/thumb printed informed consent of the subject must be obtained before study participation. Refer to Section 6.1 for the requirements on how to obtain informed consent and assent, as appropriate.

When a subject needs the assistance of a caregiver in completing study procedures, this will be confirmed during informed consent by the subject. In addition, the caregiver will also provide his/her agreement to be involved in the study and express willingness to act in a support role during the conduct of study specific procedures. The agreement of the caregiver to participate to the study will be included in the subject's ICF in a separate dedicated paragraph/annex. The role of the caregiver will be fully explained in the ICF or annex where the subject and the caregiver will confirm their involvement in the study.

The caregiver can stop participation in the study for any reason at any time, and he/she should be replaced by another caregiver. The former caregiver must not be involved in the consent process and the appointment of a new caregiver. The new caregiver will confirm his/her participation following the same process: by having both subject and him/her-self (re-)signing the annex part of the ICF.

For subjects with suspected HZ at Visit HZ-1 (i.e., either ongoing cases or subjects developing a new case), an informed consent addendum or update may be required to inform these subjects of the change in the follow-up period for pain assessment questionnaire.

6.5.2. Check inclusion and exclusion criteria

Check all inclusion and exclusion criteria as described in Sections 5.2 and 5.3 before enrolment.

6.5.3. Medical history

Obtain the subject's clinically relevant medical history by interview and/or review of the subject's medical records and record any pre-existing conditions or signs and/or symptoms present in a subject prior to the first study visit in the eCRF.

Clinically relevant prior medical history is defined as: chronic disease or medical conditions requiring continued or chronic treatment (e.g., diabetes, psoriasis), any previous malignant cancer, acute disease resolved with sequelae (e.g., hemiplegia due to cerebrovascular accident [CVA]), any pIMDs (see Table 24).

Excluded from recording are: acute infections that have resolved (e.g., lobar pneumonia, influenza), medical events that have resolved (e.g., hip fracture with replacement, cataract treated with surgery). A predefined list of categories and diseases will be available in the eCRF.

In addition, history of prior HZ episode must be recorded.

Any available information for the diagnosis of HZ episode (clinical diagnosis, PCR, laboratory methods, etc.) during the interval between the end of the ZOSTER-006/022 studies and beginning of the ZOSTER-049 study is to be collected.

6.5.4. Physical examination

A history-directed physical examination according to local practice should be performed to ensure the subject is in good physical condition.

6.5.5. Pregnancy test

Female subjects of childbearing potential are to have a urine or serum pregnancy test before randomisation (in the 1-Additional Dose, Revaccination and Control groups) and prior to study vaccine second dose administration (in the Revaccination group, only). The study vaccines may only be administered if the pregnancy test is negative. Note: The urine pregnancy test must be performed even if the subject is menstruating at the time of the study visit.

A urine pregnancy test is sufficient. A serum pregnancy test instead of a urine pregnancy test should only be considered if required by country, local or ethics committee regulations.

6.5.6. Check contraindications, warnings and precautions to vaccination

Contraindications to vaccination must be checked before randomisation (in the 1-Additional Dose, Revaccination and Control groups, only) and prior to study vaccine second dose administration (in the Revaccination group, only). Refer to Section 7.5 for more details.

6.5.7. Training on self-reporting by subjects

Subjects/ subject's caregiver will be instructed at Visit Month 0 (and will be reminded at yearly visit or phone contact) to contact their study site immediately:

- should the subject manifest any signs or symptoms he/she perceive as serious;
- should the subject become pregnant (for women of childbearing potential in the groups 1-Additional Dose and Revaccination, only).
- should the subject develop any symptoms suggestive of HZ. The subject should be reminded to start completion of the HZ-specific diary card immediately upon development of these symptoms prior to visiting the study site for evaluation of the suspected HZ.

6.5.7.1. Reminder for monthly follow-up contacts/yearly follow-up visits

The subject/ subject's caregiver will be reminded that, after Visit Month 0, monthly contacts between the subjects and the investigator and/or his delegate will take place (except at months that coincide with the subject's scheduled visits [see Section 6.3.1.1]) in order to collect all relevant information on occurrence or follow up of a suspected episode of HZ (Section 4.5), SAEs (Section 9.3), IMCs (Section 7.7) or the use of concomitant medications and/or vaccinations (Section 7.6)], and that information will be recorded in the appropriate section of the subject's eCRF.

6.5.8. Sampling

Refer to the Module on Biospecimen Management in the SPM for detailed instructions for the collection, handling and processing of the samples.

6.5.8.1. Blood sampling for immune response assessments

As specified in (Table 4, Table 5, Table 15 and Table 19), blood samples will be taken during study visits from subsets of subjects as follows:

- **All subjects** (N ≤ 14,448) entering the study will have a HI blood sample (approximately 5 mL) at Visit Month 0.

  For subjects in the LTFU group, who do not belong to any subset, these samples will be stored and tested for HI only if the subject develops HZ during the ZOSTER-049 study or if there are other reasons requiring the HI testing of these samples.

- **LTFU Group (HI subset):** Subjects (N ≤ 1,729) who were in the immunogenicity subset during studies ZOSTER-006/022 and continue participation in this study. Blood samples (approximately 5 mL) will be collected and tested from Visit Month 0 to Visit Year 6, on a yearly basis, to assess HI responses.

- **LTFU Group (CMI subset):** Subjects (N ≤ 234) who were in the CMI subset during study ZOSTER-006 and continue participation in this study. Blood samples (approximately 20 mL) will be collected and tested from Visit Month 0 to Visit Year 6 on a yearly basis to assess CMI responses.

- **HZ subset:** Subjects (N to be determined) who developed confirmed HZ during ZOSTER-006 or ZOSTER-022, or who develop HZ during the interval between the end of the ZOSTER-006/022 and the beginning of ZOSTER-049, or who develop suspected HZ during ZOSTER-049 will be part of the HZ subset. Blood sampling for subjects in the HZ subset is described below.

  Subjects who develop HZ at any time after enrolment in ZOSTER-006 or ZOSTER-022 and who are already part of the HI subset in ZOSTER-006 or ZOSTER-022 will provide blood samples to assess HI responses (approximately 5 mL) from Visit Month 0 to Visit Year 6 irrespective of when the HZ episode occurs. If these subjects are part of the ZOSTER-006 CMI subset, then they will also continue to provide blood to assess CMI responses (approximately 20 mL).

Subjects who develop HZ at any time after enrolment in ZOSTER-006 or ZOSTER-022 and who were NOT part of the HI or CMI subsets in these studies, will provide blood samples to assess HI responses (approximately 5 mL) beginning at the annual visit in the ZOSTER-049 study subsequent to the occurrence of the HZ episode. The blood sample taken upon enrolment at Visit Month 0 will be tested and included in the analyses for these subjects.

- **1-Additional Dose Group:** Subjects (N = 60) to be administered HZ/su vaccine on a 1-dose schedule at Visit Month 0. Blood samples (approximately 5 and 20 mL) will be collected at Visit Month 0, Visit Month 1, and from Visit Year 1 to Visit Year 6 on a yearly basis to assess HI and CMI responses.
- **Revaccination Group:** Subjects (N = 60) to be administered HZ/su vaccine on a 2-dose schedule at Visit Month 0 and Visit Month 2. Blood samples (approximately 5 and 20 mL) will be collected at Visit Month 0, Visit Month 1, Visit Month 3 and from Visit Year 1 to Visit Year 6 on a yearly basis to assess HI and CMI responses.
- **Control Group:** Subjects (N = 120) in the non-vaccinated control group. Blood samples (approximately 5 and 20 mL) will be collected at Visit Month 0, Visit Month 1, Visit Month 3 and from Visit Year 1 to Visit Year 6 on a yearly basis to assess HI and CMI responses.

After centrifugation, serum samples for HI assessment should be kept at –20°C/ –4°F or –70/80°C until shipment.

The blood samples for CMI assessment should be stored at the investigator's site at room temperature and they must not be centrifuged. Samples will be shipped at room temperature (20 to 25°C/68 to 77°F) to the designated laboratory for cell separation to be performed within 24 hours.

Refer to the Module on Biospecimen Management in the SPM for general handling of blood samples.

6.5.8.2. Clinical specimens of HZ lesions for PCR analysis

Clinical specimens of HZ lesions will be collected from subjects clinically diagnosed as having a suspected case of HZ (see Sections 4.5.2.4.1, 4.5.3 and 6.7.4.2). Samples for HZ cases assessment should be kept at –20°C/ –4°F or –70/80°C until shipment.

Refer to the SPM for more details on sample storage conditions.

6.5.9. Check and record concomitant medication/vaccination and IMCs

Concomitant medication/vaccination must be checked and recorded in the eCRF as described in Section 7.6.

Intercurrent medical conditions must be checked and recorded in the eCRF as described in Section 7.7.

6.5.10. Recording of AEs and SAEs

- Refer to Section 9.3 for procedures for the investigator to record AEs, SAEs, pregnancies and pIMDs. Refer to Section 9.4 for guidelines and how to report SAE, pregnancy and pIMD reports to GSK Biologicals.
- The subject/ subject's caregiver will be instructed to contact the investigator immediately should the subjects manifest any signs or symptoms they perceive as serious.
- For the two groups (1-Additional Dose and Revaccination, only), at each vaccination visit, diary cards will be provided to the subject/ subject's caregiver (see Section 6.3.1.2). The subjects/ subject's caregiver will be trained on how to complete the diary cards. The subject/ subject's caregiver will record any solicited AEs (from Day 0 to Day 6 after each vaccination) on the 7-day diary cards and will record any unsolicited AEs (from Day 0 to Day 29 after each vaccination) and any concomitant medication and vaccination taken from Day 0 to Day 29 after each vaccination on the 30-day diary cards (see Table 6 and Table 26).
- The subject/ subject's caregiver will be instructed to return the completed diary cards to the investigator at the next study visit. The study staff/investigator will collect and verify completed diary cards during discussion with the subject/ subject's caregiver on Visit Month 1 (1-Additional Dose and Revaccination groups) and Visit Month 3 (Revaccination group, only).
- Any unreturned diary cards will be sought from the subject/ subject's caregiver through telephone call(s) or any other convenient procedure. The investigator will transcribe the collected information into the eCRF in English.

6.5.11. Study conclusion

Study conclusion takes place at the last yearly visit/ contact (i.e., the study visit/ contact following completion of approximately 6 years follow-up from Visit Month 0) for subjects not in HZ follow-up at the time of the last yearly visit. The study conclusion visit/ contact for subjects in HZ follow-up at the time of the last yearly visit will occur after until a 4-week pain-free period is documented OR after 90 days of HZ follow-up has been completed and will be combined with the visit/ contact at Day HZ-91. The Day HZ-91 visit/ contact will preferably be a visit, but a contact is an option.

At the study conclusion visit/contact, the investigator will:

- review data collected to ensure accuracy and completeness,
- complete the Study Conclusion screen in the eCRF.

6.6. *Study procedures during special circumstances (Amended 11 May 2020)*

During special circumstances (e.g., COVID-19 pandemic), the specific guidance from local public health and other competent authorities regarding the protection of individuals' welfare must be applied. For the duration of such special circumstances, the following measures may be implemented for enrolled subjects:

The impact of special circumstances to the study conduct will be documented in the clinical study report.

- ***Yearly site visits/contact may be made by other means of virtual contact or home visit, if appropriate.***
- ***Biological samples may be collected at a different location* other than the study site or at subject's home. Biological samples should not be collected if they cannot be processed in a timely manner or appropriately stored until the intended use.***

****** It is the investigator's responsibility to identify an alternate location. The investigator should ensure that this alternate location meets ICH GCP requirements, such as adequate facilities to perform study procedures, appropriate training of the staff and documented delegation of responsibilities in this location. This alternate location should be covered by proper insurance for the conduct of study on subjects by investigator and staff at a site other than the designated study site. Refer to EMA Guidance on the Management of Clinical Trials during the COVID-19 (Coronavirus) pandemic (version 3, 28 April 2020) or other relevant authority guidance for more details.***

- ***If despite best efforts it is not possible to collect the biological samples within the interval predefined in the protocol (see Table 8 to Table 10), then the allowed interval may be extended by 30 days as outlined from Table 12 to Table 14.***
  - ***The visit can be replaced by a phone contact or other means of virtual contact and will be performed in the interval predefined in the protocol. The blood sampling can be performed according to the interval allowed during special circumstances as shown from Table 12 to Table 14.***

Table 12 ***Intervals for blood sampling for the LTFU group during special circumstances (Amended 11 May 2020)***

Interval for blood sampling	Optimal length of interval (in months)	Allowed interval during normal circumstances (range in days)	*Allowed interval during special circumstances (range in days)*
Visit Month 0 → Blood sampling Year 1	12 months	335 – 395	335 ***– 425***
Visit Month 0 → Blood sampling Year 2	24 months	700 – 760	700 ***– 790***
Visit Month 0 → Blood sampling Year 3	36 months	1065 – 1125	1065 ***– 1155***
Visit Month 0 → Blood sampling Year 4	48 months	1430 – 1490	1430 ***– 1520***
Visit Month 0 → Blood sampling Year 5	60 months	1795 – 1855	1795 ***– 1885***
Visit Month 0 → Blood sampling Year 6	72 months	2160 – 2220	2160 ***– 2250***

Note: Investigator should prioritize performing the blood sampling as close to the optimal window as possible.

Table 13 Intervals for blood sampling for the 1-Additional Dose and Control groups during special circumstances (Amended 11 May 2020)

Interval for blood sampling	Optimal length of interval (in months)	Allowed interval during normal circumstances (range in days)	*Allowed interval during special circumstances (range in days)*
Visit Month 0 → Blood sampling Year 1	12 months	335 – 395	335 **– *425***
Visit Month 0 → Blood sampling Year 2	24 months	700 – 760	700 **– *790***
Visit Month 0 → Blood sampling Year 3	36 months	1065 – 1125	1065 **– *1155***
Visit Month 0 → Blood sampling Year 4	48 months	1430 – 1490	1430 **– *1520***
Visit Month 0 → Blood sampling Year 5	60 months	1795 – 1855	1795 **– *1885***
Visit Month 0 → Blood sampling Year 6	72 months	2160 – 2220	2160 **– *2250***

Note: Investigator should prioritize performing the blood sampling as close to the optimal window as possible.

Table 14 Intervals for blood sampling for the Revaccination group during special circumstances (Amended 11 May 2020)

Interval for blood sampling	Optimal length of interval (in months)	Allowed interval during normal circumstances (range in days)	*Allowed interval during special circumstances (range in days)*
Visit Month 2 → Blood sampling Year 1	12 months	335 – 395	335 **– *425***
Visit Month 2 → Blood sampling Year 2	24 months	700 – 760	700 **– *790***
Visit Month 2 → Blood sampling Year 3	36 months	1065 – 1125	1065 **– *1155***
Visit Month 2 → Blood sampling Year 4	48 months	1430 – 1490	1430 **– *1520***
Visit Month 2 → Blood sampling Year 5	60 months	1795 – 1855	1795 **– *1885***
Visit Month 2 → Blood sampling Year 6	72 months	2160 – 2220	2160 **– *2250***

Note: Investigator should prioritize performing the blood sampling as close to the optimal window as possible.

- ***Visits for suspected HZ may take place in a different location* other than the study site or at subject's home. If this is not feasible, then the medical evaluation of suspected HZ may take place virtually with documentation of all the signs and symptoms as outlined in Table 7 and Table 11.***
  - ***If the subject is not able to immediately contact the study staff/investigator to evaluate/ clinically diagnose the suspected HZ case, the subject should be encouraged to document all the signs and symptoms, HZ related treatments and record the progression of the rash and share it with the investigator when possible.***
  - ***Digital photographs can be taken by the study staff/investigator at a different location* other than the study site or at subject's home. If this is not feasible, subjects might be asked to take photographs of their suspected HZ lesions themselves. The photographs will be transferred to the investigator via email or other modes of virtual contact when possible.***
  - ***If feasible, the subject may be asked to collect rash lesion sample at home.***

- ***HZ-specific diary cards may be transmitted from and to the site by electronic means and/or conventional mail.***
- ***ZBPI questionnaire may be transmitted from and to the site by electronic means and/or conventional mail. If this is not feasible, the study staff/investigator can conduct ZBPI questionnaire by telephone contact. Study staff/investigator will read the questions (verbatim) and transcribe the subject's responses on the questionnaires.***

Impact on the modified TVC for efficacy and ATP for efficacy and immunogenicity (humoral and CMI) will be determined on a case by case basis.

6.7. Biological sample handling and analysis

Please refer to the SPM for details on biospecimen management (handling, storage and shipment).

Samples will not be labelled with information that directly identifies the subject but will be coded with the identification number for the subject (subject number).

- Collected samples will be used for protocol mandated research and purposes related to the improvement, development and quality assurance of the laboratory tests described in this protocol. This may include the management of the quality of these tests, the maintenance or improvement of these tests, the development of new test methods, as well as making sure that new tests are comparable to previous methods and work reliably.
- It is also possible that future findings may make it desirable to use the samples acquired in this study for future research, not described in this protocol. Therefore, all subjects in countries where this is allowed, will be asked to give a specific consent to allow GSK or a contracted partner to use the samples for future research. Future research will be subject to the laws and regulations in the respective countries and will only be performed once an independent Ethics Committee or Review Board has approved this research.

Information on further investigations and their rationale can be obtained from GSK Biologicals.

Any sample testing will be done in line with the consent of the individual subject.

Refer also to the a Investigator Agreement, where it is noted that the investigator cannot perform any other biological assays except those described in the protocol or its amendment(s).

Collected samples will be stored for a maximum of 20 years (counting from when the last subject performed the last study visit), unless local rules, regulations or guidelines require different timeframes or different procedures, which will then be in line with the subject consent. These extra requirements need to be communicated formally to and discussed and agreed with GSK Biologicals.

6.7.1. Use of specified study materials

When materials are provided by GSK Biologicals, it is MANDATORY that all clinical samples (including serum samples) be collected and stored exclusively using those materials in the appropriate manner. The use of other materials could result in the exclusion of the subject from the ATP analysis (See Section 11.5 for the definition of cohorts to be analysed). The investigator must ensure that his/her personnel and the laboratory(ies) under his/her supervision comply with this requirement. However, when GSK Biologicals does not provide material for collecting and storing clinical samples, appropriate materials from the investigator's site must be used. Refer to the Module on Clinical Trial Supplies in the SPM.

6.7.2. Biological samples

Table 15 Biological samples

Sample type	Quantity (approximate volume)	Unit	Timepoint(s) [1]	Subset Name [2]
Blood (Humoral immunology)	5	mL	Visit Month 0	All Subjects
Blood (Humoral immunology)	5	mL	Visit Month 0, Visit Years 1, 2, 3, 4, 5 and 6	LTFU Group HI subset
Blood (Humoral immunology)	5	mL	Visit Month 0, Visit Years 1, 2, 3, 4, 5 and 6	HZ subset
Blood (Humoral immunology)	5	mL	Visit Month 0, Visit Month 1, Visit Month 3 [3] and Visit Years 1, 2, 3, 4, 5 and 6	1-Additional Dose Group, Revaccination Group; Control Group
Blood (Cell-mediated immunology)	20	mL	Visit Month 0, and Visit Years 1, 2, 3, 4, 5 and 6	LTFU Group CMI subset
Blood (Cell-mediated immunology)	20	mL	Visit Month 0, Visit Month 1, Visit Month 3 [3] and Visit Years 1, 2, 3, 4, 5 and 6	1-Additional Dose Group Revaccination Group Control Group
Specimens of HZ lesions	3 replicate samples, taken on the same day, of the highest priority lesion type available (1) vesicle fluid; 2) crust; 3) crust swab; 4) papule swab) [4]	NA	Scheduled in case of suspected HZ for diagnosis	Subjects clinically diagnosed as having a suspected case of HZ

mL = Millilitre, LTFU = Long-Term Follow-Up; HI = Humoral Immunogenicity; HZ = Herpes Zoster; CMI = Cell-Mediated Immunity; NA = Not Applicable.

1 Refer to Section 6.5.8.1 for the blood sampling timepoints.

2 Refer to Table 4 and Table 19 for description of the subsets and Section 6.1.2 for the allocation of subjects to subsets.

3 Only the Revaccination and Control groups.

4 If during clinical evaluation at Visit HZ-1, the investigator determines that adequate rash samples are not present at Visit HZ-1 (i.e., <3 lesions present or only papules present), the investigator has the option of collecting three

additional samples prior to or at Visit HZ-2 (preferably within 7 days). See Table 7 and the SPM for further details on sample collection.

6.7.3. Laboratory assays

Please refer to Appendix A for a detailed description of the assays performed in the study. Please refer to Appendix B for the address of the clinical laboratories used for sample analysis.

Laboratory assays, which will be used in this study, are summarised in Table 16 (Humoral Immunity), Table 17 (Cell-Mediated Immunity) and Table 18 (Molecular Biology), respectively.

Table 16 Humoral Immunity (Antibody determination)

System	Component	Method	Kit / Manufacturer	Unit	Cut-off	Laboratory
Serum	Varicella Zoster Virus.Glycoprotein E Ab.IgG	ELISA	NA	mIU/ml	97	GSK Biologicals*

Ab = antibody; IgG = Immunoglobulin class G; ELISA = Enzyme-linked Immunosorbent Assay; NA = Not applicable; mIU = milli-international unit.
* see Table 31 in Appendix B.

Table 17 Cell-Mediated Immunity (CMI)

System	Component	Challenge	Method	Unit	Cut-off	Laboratory
PBMC	CD4.polypositives CD40L+IL-2+TNFα+IFNγ*	gE	ICS	Events	N/A	CEVAC +
PBMC	[redacted]					GSK Biologicals*

PBMC = Peripheral Blood Mononuclear Cells; IL-2 = Interleukin-2; TNF = Tumor Necrosis Factor; IFN = Interferon; gE = Glycoprotein E; ICS = Intracellular cytokine staining; [redacted] N/A = Not Applicable.
* CD4.polypositives CD40L+IL-2+TNF-α + IFN-γ = CD4+ T-cells expressing at least 2 activation markers (from among IFN-γ, IL-2, TNF-α and CD40L).
+ CEVAC refers to the Centre for Vaccinology, (see Table 32 in Appendix B).
* see Table 31 in Appendix B.

Table 18 Molecular Biology (PCR tests)

System	Component	Method	Unit	Laboratory
HZ lesion sample	Varicella Zoster Virus DNA	PCR	No unit	GSK Biologicals*
HZ lesion sample	Actin Gene DNA	PCR	No unit	GSK Biologicals*

HZ = Herpes Zoster; DNA = Deoxyribonucleic acid; PCR = Polymerase Chain Reaction.
* see Table 31 in Appendix B.

The GSK Biologicals' clinical laboratories have established a Quality System supported by procedures. The activities of GSK Biologicals' clinical laboratories are audited regularly for quality assessment by an internal (sponsor-dependent) but laboratory-independent Quality Department.

6.7.4. Biological samples evaluation

6.7.4.1. Immunological read-outs

The plan for immunogenicity testing on samples obtained is shown in Table 19.

Table 19 Immunological read-outs

Blood sampling timepoint [1]			Subset Name [2]	Approximate No. of Subjects	Component
Visit No.[3]	Month	Timing			
Visit Month 0	0	Post-Primary	All Subjects	≤ 14448 [4]	Ab gE ELISA
			LTFU Group CMI subset	≤ 234	ICS gE
		Pre-Vacc	1-Additional Dose, Revaccination and Control Groups	240	gE ICS & CCI [redacted]
Visit Month 1	1	Post-Vacc 1	1-Additional Dose, Revaccination and Control Groups	240	gE ICS & CCI [redacted]
			1-Additional Dose, Revaccination and Control Groups	240	Ab gE ELISA
Visit Month 3	3	Post–Vacc 2	Revaccination and Control Groups	180	gE ICS & CCI [redacted]
			Revaccination and Control Groups	180	Ab gE ELISA
Visit Year 1, Visit Year 2, Visit Year 3, Visit Year 4, Visit Year 5, Visit Year 6	12, 24, 36, 48, 60, 72	Post-Primary	LTFU Group CMI subset	≤ 234	gE ICS g
			LTFU Group HI subset	≤ 1729 [5]	Ab gE ELISA
			HZ subset	TBD	Ab gE ELISA
		Post-Vacc 1/2	1-Additional Dose, Revaccination and Control Groups	240	gE ICS & CCI [redacted]
			1-Additional Dose, Revaccination and Control Groups	240	Ab gE ELISA

Post-Primary = Post-primary vaccination during ZOSTER-006/022; Pre-Vacc = Pre Vaccination; Post-Vacc 1 = Post Vaccination 1 (post dose 1); Post-Vacc 2 = Post-Vaccination 2 (post dose 2); LTFU = Long-Term Follow-Up; CMI = Cell-Mediated Immunity; HI = Humoral Immunogenicity; HZ = Herpes Zoster; TBD = To Be Determined; ICS = Intracellular cytokine staining; gE = Glycoprotein E; Ab = Antibody; ELISA = Enzyme-linked Immunosorbent Assay; CCI [redacted]

[1] Refer to Section 6.5.8.1 for the blood sampling timepoints.

[2] Refer to Table 4 and Table 15 for description of the subsets.

[3] Refer to Table 8, Table 9 and Table 10 for the intervals between study visits for the LTFU group, the 1-Additional Dose and Control groups and for the Revaccination group, respectively.

[4] All subjects (N ≤ 14,448) entering the study will have a HI blood sample (approximately 5 mL) at Visit Month 0. For subjects in the LTFU group, who do not belong to any subset, these samples will be stored and tested for HI only if the subject develops HZ during the ZOSTER-049 study or if there are other reasons requiring the HI testing of these samples (see Section 6.5.8.1).

[5] Subjects to be included in the LTFU Group HI subset are from the immunogenicity subset in studies ZOSTER-006/022 [N = 1269 (ZOSTER-006) + 460 (ZOSTER-022) = 1729 subjects at a maximum].

[6] CCI [redacted] experiments will be performed on available PBMC following gE ICS analysis.

Additional blood samples given at the pre-vaccination (Month 0) and 1 month post-dose 2 (Month 3) timepoints in the ZOSTER-006/022 primary studies will be tested for the analysis of the humoral immune response from subjects in the 1-Additional Dose, Revaccination and Control groups and from subjects belonging to the HZ subset, if they were not tested previously.

Additional testing (e.g., VZV specific CMI measurements on available blood samples) may be performed if deemed appropriate by GSK Biologicals should any findings in the present study, or in other studies, indicate that further investigation of the immunogenicity of the vaccine is warranted.

6.7.4.2. Test for laboratory diagnosis of HZ

In case of a suspected HZ case diagnosis in any of the subjects, clinical specimens from HZ lesions will be collected to confirm the diagnosis of HZ by PCR (Table 18). Please refer to Appendix A for or a detailed description of the PCR.

6.7.5. Immunological correlates of protection

No generally accepted immunological correlate of protection against HZ has been demonstrated so far for the gE antigen used in the HZ/su study vaccine.

7. STUDY VACCINE AND ADMINISTRATION

7.1. Description of study vaccine

The study vaccine/product to be used has been developed and manufactured by GSK Biologicals.

The Quality Control Standards and Requirements for the study vaccine are described in separate Quality Assurance documents (e.g., release protocols, certificate of analysis) and the required approvals have been obtained.

The vaccine is labelled and packed according to applicable regulatory requirements.

Table 20 Study vaccine

Treatment name	Vaccine name	Formulation	Presentation	Volume to be administered*	Group	Number of doses
HZ/su	VZV gE	gE=50µg	Lyophilized pellet in a monodose vial	0.5 mL	1-Additional Dose	1
	$AS01_B$	MPL=50µg; QS21=50µg; Liposomes	Liquid in a monodose vial		Revaccination	2

*Refer to the SPM for the volume after reconstitution.
HZ/su = Herpes Zoster subunit vaccine; VZV = Varicella Zoster Virus, gE = recombinant purified Glycoprotein E; µg = Microgram; mL = Millilitre; AS01B = Adjuvant System AS01B; MPL = 3-O-desacyl-4’-monophosphoryl lipid A; QS21 = Quillaja saponaria Molina, fraction 21 (purified saponin extract from the South American tree).

7.2. Storage and handling of study vaccine

The study vaccine must be stored at the respective label storage temperature conditions in a safe and locked place. Access to the storage space should be limited to authorised study personnel. The storage conditions will be assessed during pre-study activities under the responsibility of the sponsor study contact. The storage temperature should be continuously monitored with calibrated (if not validated) temperature monitoring device(s) and recorded. Refer to the Module on Clinical Trial Supplies in the SPM for more details on storage of the study vaccine.

Temperature excursions must be reported in degree Celsius.

Any temperature excursion outside the range of 0.0 to +8.0°C (for +2 to +8°C/+36 to +46°F label storage condition) impacting investigational medicinal products (IMPs) must be reported in the appropriate (electronic) temperature excursion decision form ([e]TDF). The impacted IMPs must not be used and must be stored in quarantine at label temperature conditions until usage approval has been obtained from the sponsor.

In case of temperature excursion below +2.0°C down to 0.0°C impacting IMP(s) there is no need to report in (e)TDF, but adequate actions must be taken to restore the +2 to +8°C/+36 to +46°F label storage temperature conditions. The impacted IMP(s) may still be administered, but the site should avoid re-occurrence of such temperature excursion. Refer to the Module on Clinical Trial Supplies in the SPM for more details on actions to take.

Refer to the Module on Clinical Trial Supplies in the SPM for details and instructions on the temperature excursion reporting and usage decision process, packaging and accountability of the study vaccine.

7.3. Dosage and administration of study vaccine

After removal of the vaccine components from the temperature monitored refrigerator, the vaccine should be reconstituted and administered within 6 hours, and should be kept at room temperature (between 2°C/36°F and 30°C/86°F).

Vaccine will be administered as indicated in Table 21.

The reconstituted vaccine (0.5 mL) should be administered by IM injection into the deltoid muscle of the non-dominant arm using a standard aseptic technique.

Table 21 Dosage and administration

Type of contact (and timepoint)	Study group	Volume to be administered	Treatment name	Route [1]	Site	Side [2]
Visit Month 0 (Month 0)	1-Additional Dose	0.5 mL	HZ/su	IM	Deltoid	Non-dominant
	Revaccination					
Visit Month 2 (Month 2)	Revaccination					

[1] Intramuscular (IM)
[2] In rare situations when there is no other alternative, the injection may be given in the dominant arm.

7.4. Replacement of unusable vaccine doses

In addition to the vaccine doses provided for the planned number of subjects (including over-randomisation when applicable), at least 5 % additional vaccine doses will be supplied to replace those that are unusable.

Additional doses of the study vaccine will be supplied if necessary.

7.5. Contraindications to subsequent vaccination

The following events constitute absolute contraindications to further administration of HZ/su. If any of these events occur during the study, the subject must not receive additional doses of vaccine but may continue other study procedures at the discretion of the investigator (see Section 9.5).

- Anaphylaxis following the administration of vaccine Dose 1.
- Pregnancy (see Section 9.2.1).
- An SAE judged to be vaccine-related by the investigator.
- An episode of HZ between Visit Month 0, (Vaccination 1) and Visit Month 2 (Vaccination 2).
- Any confirmed or suspected immunosuppressive or immunodeficient condition, resulting from disease (e.g., malignancy, HIV infection) or immunosuppressive/cytotoxic therapy (e.g., medications used during cancer chemotherapy, organ transplantation or to treat autoimmune disorders). However subjects who have received less than 15 consecutive days of immunosuppressants or other immune modifying drugs should not be contraindicated from receiving subsequent vaccinations. Also, for corticosteroids, prednisone < 20 mg/day, or equivalent, is allowed. Inhaled, topical and intra-articular corticosteroids are allowed.
- Other events that constitute contraindications to administration of HZ/su vaccine.
  - Occurrence of a new pIMD or the exacerbation of an existing pIMD that, in the opinion of the investigator, exposes the subject to unacceptable risk from subsequent vaccination. In such cases, the investigator should use his/her clinical judgement prior to administering the next dose of the vaccine. Refer to Section 9.1.6.1 for the definition of pIMDs.

The following events constitute contraindications to administration of HZ/su at that point in time; if any of these events occur at the time scheduled for vaccination, the subject may be vaccinated at a later date, within the time window specified in the protocol (see Section 6.4), or the subject may be withdrawn at the discretion of the investigator (see Section 9.5).

- Acute disease and/or fever at the time of vaccination.
  - Fever is defined as temperature ≥ 37.5°C/99.5°F for oral, axillary or tympanic route, or ≥ 38.0°C/100.4°F for rectal route. The preferred route for recording temperature in this study will be oral.
  - Subjects with a minor illness (such as mild diarrhoea, mild upper respiratory infection) without fever can be administered all vaccines.
- Any condition that in the judgment of the investigator would make intramuscular injection unsafe.

7.6. Concomitant medications/products and concomitant vaccinations

At each study visit/ contact, the investigator should question the subject/ subject's caregiver about any medications/products taken and vaccinations received by the subject.

7.6.1. Recording of concomitant medications/products and concomitant vaccinations

The following concomitant medication(s)/product(s)/vaccine(s) must be recorded in the eCRF.

- All concomitant medications/products, except vitamins and dietary supplements, administered during the period starting 30 days (Days 0-29) following each dose/the dose of study vaccine. This also applies to concomitant medication administered prophylactically in anticipation of reaction to the vaccination and any medication intended to treat an AE (For the 1-Additional Dose and Revaccination groups ONLY).

  E.g., an anti-pyretic is considered to be prophylactic when it is given in the absence of fever and any other symptom, to prevent fever from occurring [fever is defined as temperature ≥ 37.5°C/99.5°F for oral, axillary or tympanic route, or ≥ 38.0°C/100.4°F for rectal route]**.**
- Any concomitant medications/products/vaccines listed in Section 7.6.2, respecting the detailed time window.
- Any concomitant medications relevant to a SAE/pIMD to be reported as per protocol or administered during the study period for the treatment of a SAE/pIMD. In addition, concomitant medications relevant to SAEs and pIMD need to be recorded on the Expedited Adverse Event report.
- Any concomitant medication/product administered for the treatment of HZ or any HZ-related complications (including pain) during the study period must be recorded in the eCRF and coded as 'Treatment for HZ'.

7.6.2. Concomitant medications/products/vaccines that may lead to the elimination of a subject from ATP analyses

The use of the following concomitant medications/products/vaccines will not require withdrawal of the subject from the study but may determine a subject's evaluability in the ATP analysis. See Section 11.5 for cohorts to be analysed.

- Use of any investigational or non-registered product (pharmaceutical product or device) during the study period;
- Administration of long-acting immune-modifying drugs (e.g., infliximab, rituximab) at any time during the study period;
- Chronic administration (defined as > 14 consecutive days in total) of immunosuppressants or other immune-modifying drugs at any time during the study period. For corticosteroids, this will mean prednisone ≥ 20 mg/day or equivalent. A prednisone dose of < 20 mg/day is allowed. Inhaled, topical and intra-articular corticosteroids are allowed;
- Administration of immunoglobulins and/or any blood products during the study period;
- Administration of cytotoxic chemotherapy at any time during the study period
- Prolonged use (> 14 consecutive days) of oral and/or parenteral antiviral agents that are active against VZV (acyclovir, valacyclovir, famciclovir, etc.) during the study period for an indication other than to treat suspected or confirmed HZ or an HZ-related complication (topical use of these antiviral agents is allowed);
- Receipt of a vaccine against VZV or HZ (including an investigational or non-registered vaccine other than the HZ/su vaccine administered in this study to groups 1-Additional Dose and Revaccination) during the entire study period;
- Administration of a vaccine not foreseen by the study protocol within 30 days prior to dose 2 of vaccine and/or within 30 days after any dose. However, licensed nonreplicating vaccines (i.e., inactivated and subunit vaccines, including inactivated and subunit influenza vaccines, with or without adjuvant for seasonal or pandemic flu) may be administered up to 8 days prior to dose 2 and/or at least 14 days after any dose of study vaccine (for the 1-Additional Dose and Revaccination groups, only);

  In case an emergency mass vaccination for an unforeseen public health threat (e.g.: a pandemic) is organised by the public health authorities, outside the routine immunisation program, the time period described above can be reduced if necessary for that vaccine provided it is licensed and used according to its Summary of Product Characteristics (SmPC) or Prescribing Information and according to the local governmental recommendations and provided a written approval of the Sponsor is obtained.

7.7. Intercurrent medical conditions (IMCs) that may lead to elimination of a subject from ATP analyses

At each study visit subsequent to study enrolment until the study end, it must be verified if the subject has experienced or is experiencing any IMC. If it is the case, the condition(s) must be recorded in the eCRF (see Table 25).

An IMC is defined as a condition that has the capability of confounding the immune response to the study vaccine or its interpretation. Subjects may be eliminated from certain ATP cohorts for immunogenicity if, during the study, they incur an IMC.

Examples of IMCs include cases of HZ or a confirmed or suspected immunosuppressive or immunodeficient condition resulting from disease (e.g., malignancy, HIV infection).

8. HEALTH ECONOMICS

Not applicable.

9. SAFETY

The investigator or site staff is/are responsible for the detection, documentation and reporting of events meeting the criteria and definition of an adverse event (AE) or serious adverse event (SAE) as provided in this protocol.

Each subject/ subject's caregiver will be instructed to contact the investigator immediately should they/the subject manifest any signs or symptoms they perceive as serious.

9.1. Safety definitions

9.1.1. Definition of an adverse event

An AE is any untoward medical occurrence in a clinical investigation subject, temporally associated with the use of a medicinal product, whether or not considered related to the medicinal product.

An AE can therefore be any unfavourable and unintended sign (including an abnormal laboratory finding), symptom, or disease (new or exacerbated) temporally associated with the use of a medicinal product. For marketed medicinal products, this also includes failure to produce expected benefits (i.e., lack of efficacy), abuse or misuse.

Examples of an AE include:

- Exacerbation of a chronic or intermittent pre-existing condition including either an increase in frequency and/or intensity of the condition.
- New conditions detected or diagnosed after investigational vaccine administration even though they may have been present prior to the start of the study.

- Signs, symptoms, or the clinical sequelae of a suspected interaction.
- Signs, symptoms, or the clinical sequelae of a suspected overdose of either investigational vaccine or a concurrent medication (overdose per se should not be reported as an AE/SAE).
- Signs, symptoms temporally associated with vaccine(s)/product(s) administration.
- Pre- or post-treatment events that occur as a result of protocol-mandated procedures (i.e., invasive procedures, modification of subject's previous therapeutic regimen).

Examples of an AE DO NOT include:

- Medical or surgical procedures (e.g., endoscopy, appendectomy); the condition that leads to the procedure is an AE/SAE.
- Situations where an untoward medical occurrence did not occur (e.g., social and/or convenience admission to a hospital, admission for routine examination).
- Anticipated day-to-day fluctuations of pre-existing disease(s) or condition(s) present or detected at the start of the study that do not worsen.
- Pre-existing clinically relevant conditions or signs and/or symptoms present in a subject prior to the study entry. These events will be recorded in the medical history section of the eCRF.

9.1.2. Definition of a serious adverse event

A SAE is any untoward medical occurrence that:

a. Results in death,

b. Is life-threatening,

   Note: The term 'life-threatening' in the definition of 'serious' refers to an event in which the subject was at risk of death at the time of the event. It does not refer to an event, which hypothetically might have caused death, had it been more severe.

c. Requires hospitalisation or prolongation of existing hospitalisation,

   Note: In general, hospitalisation signifies that the subject has been admitted at the hospital or emergency ward for observation and/or treatment that would not have been appropriate in the physician's office or in an out-patient setting. Complications that occur during hospitalisation are also considered AEs. If a complication prolongs hospitalisation or fulfils any other serious criteria, the event will also be considered serious. When in doubt as to whether 'hospitalisation' occurred or was necessary, the AE should be considered serious.

   Hospitalisation for elective treatment of a pre-existing condition (known or diagnosed prior to informed consent signature) that did not worsen from baseline is NOT considered an AE.

d. Results in disability/incapacity, OR

Note: The term disability means a substantial disruption of a person's ability to conduct normal life functions. This definition is not intended to include experiences of relatively minor medical significance such as uncomplicated headache, nausea, vomiting, diarrhoea, influenza like illness, and accidental trauma (e.g., sprained ankle) which may interfere or prevent everyday life functions but do not constitute a substantial disruption.

e. Is a congenital anomaly/birth defect in the offspring of a study subject.

Medical or scientific judgement should be exercised in deciding whether reporting is appropriate in other situations, such as important medical events that may not be immediately life-threatening or result in death or hospitalisation but may jeopardise the subject or may require medical or surgical intervention to prevent one of the other outcomes listed in the above definition. These should also be considered serious. Examples of such events are invasive or malignant cancers, intensive treatment in an emergency room or at home for allergic bronchospasm, blood dyscrasias or convulsions that do not result in hospitalisation.

9.1.3. Solicited adverse events

9.1.3.1. Solicited local (injection-site) adverse events

The following local (injection-site) AEs will be solicited (Table 22):

Table 22 Solicited local adverse events

Pain at injection site
Redness at injection site
Swelling at injection site

9.1.3.2. Solicited general adverse events

The following general AEs will be solicited (Table 23):

Table 23 Solicited general adverse events

Fatigue
Fever
Gastrointestinal symptoms †
Headache
Myalgia
Shivering

†Gastrointestinal symptoms include nausea, vomiting, diarrhea and/or abdominal pain.

Note: Temperature (oral route preferred) will be recorded in the evening. Should additional temperature measurements be performed at other times of day, the highest temperature will be recorded in the eCRF.

9.1.4. Clinical laboratory parameters and other abnormal assessments qualifying as adverse events or serious adverse events

In absence of diagnosis, abnormal laboratory findings (e.g., clinical chemistry, haematology, urinalysis) or other abnormal assessments (e.g., imaging studies) that are judged by the investigator to be clinically significant will be recorded as AE or SAE if they meet the definition of an AE or SAE (refer to Sections 9.1.1 and 9.1.2). Clinically significant abnormal laboratory findings or other abnormal assessments that are present at baseline and significantly worsen following the start of the study will also be reported as AEs or SAEs.

The investigator will exercise his or her medical and scientific judgement in deciding whether an abnormal laboratory finding or other abnormal assessment is clinically significant.

9.1.5. Medically attended visits

The subjects in the 1-Additional Dose, Revaccination and Control groups only will be asked if they received medical attention defined as hospitalization, an emergency room visit or a visit to or from medical personnel (medical doctor) for any reason, other than routine health care visits. This information on medically attended visits will be collected from Visit Month 0 until 6 months (for the Control and 1-Additional Dose groups) and from Visit Month 0 until 6 months after last HZ/su vaccination (for the Revaccination group) and will be recorded in the eCRF (see Table 6 and Table 26).

9.1.6. Adverse events of specific interest

9.1.6.1. Potential immune-mediated diseases

pIMDs are a subset of AEs that include autoimmune diseases and other inflammatory and/or neurologic disorders of interest which may or may not have an autoimmune aetiology. AEs that need to be recorded and reported as pIMDs include those listed in Table 24.

However, the investigator will exercise his/her medical and scientific judgement in deciding whether other diseases have an autoimmune origin (i.e., pathophysiology involving systemic or organ-specific pathogenic autoantibodies) and should also be recorded as a pIMD.

Table 24 List of potential immune-mediated diseases

Neuroinflammatory disorders	Musculoskeletal disorders	Skin disorders
• Cranial nerve disorders, including paralyses/paresis (e.g., Bell's palsy)<br>• Optic neuritis<br>• Multiple sclerosis<br>• Transverse myelitis<br>• Guillain-Barré syndrome, including Miller Fisher syndrome and other variants<br>• Acute disseminated encephalomyelitis, including site specific variants: e.g., non-infectious encephalitis, encephalomyelitis, myelitis, myeloradiculoneuritis<br>• Myasthenia gravis, including Lambert-Eaton myasthenic syndrome<br>• Immune-mediated peripheral neuropathies and plexopathies, (including chronic inflammatory demyelinating polyneuropathy, multifocal motor neuropathy and polyneuropathies associated with monoclonal gammopathy).<br>• Narcolepsy	• Systemic lupus erythematosus and associated conditions<br>• Systemic scleroderma (Systemic sclerosis), including diffuse systemic form and CREST syndrome<br>Idiopathic inflammatory myopathies, including dermatomyositis<br>• Polymyositis<br>• Antisynthetase syndrome<br>• Rheumatoid arthritis, and associated conditions including juvenile chronic arthritis and Still's disease<br>• Polymyalgia rheumatica<br>• Spondyloarthritis, including ankylosing spondylitis, reactive arthritis (Reiter's Syndrome) and undifferentiated spondyloarthritis<br>• Psoriatic arthropathy<br>• Relapsing polychondritis<br>• Mixed connective tissue disorder	• Psoriasis<br>• Vitiligo<br>• Erythema nodosum<br>• Autoimmune bullous skin diseases (including pemphigus, pemphigoid and dermatitis herpetiformis)<br>• Alopecia areata<br>• Lichen planus<br>• Sweet's syndrome<br>• Localised Scleroderma (Morphoea)
**Vasculitides**	**Blood disorders**	**Others**
• Large vessels vasculitis including: giant cell arteritis such as Takayasu's arteritis and temporal arteritis.<br>• Medium sized and/or small vessels vasculitis including: polyarteritis nodosa, Kawasaki's disease, microscopic polyangiitis, Wegener's granulomatosis, Churg–Strauss syndrome (allergic granulomatous angiitis), Buerger's disease (thromboangiitis obliterans), necrotizing vasculitis and anti-neutrophil cytoplasmic antibody (ANCA) positive vasculitis (type unspecified), Henoch-Schonlein purpura, Behcet's syndrome, leukocytoclastic vasculitis.	• Autoimmune hemolytic anemia<br>• Autoimmune thrombocytopenia<br>• Antiphospholipid syndrome<br>• Pernicious anemia<br>• Autoimmune aplastic anaemia<br>• Autoimmune neutropenia<br>• Autoimmune pancytopenia	• Autoimmune glomerulonephritis (including IgA nephropathy, glomerulonephritis rapidly progressive, membranous glomerulonephritis, membranoproliferative glomerulonephritis, and mesangioproliferative glomerulonephritis)<br>• Ocular autoimmune diseases (including autoimmune uveitis and autoimmune retinopathy)<br>• Autoimmune myocarditis/cardiomyopathy<br>Sarcoidosis<br>• Stevens-Johnson syndrome<br>• Sjögren's syndrome<br>• Idiopathic pulmonary fibrosis<br>• Goodpasture syndrome<br>• Raynaud's phenomenon

Liver disorders	Gastrointestinal disorders	Endocrine disorders
• Autoimmune hepatitis<br>• Primary biliary cirrhosis<br>• Primary sclerosing cholangitis<br>• Autoimmune cholangitis	• Inflammatory Bowel disease, including Crohn's disease, ulcerative colitis, microscopic colitis, ulcerative proctitis<br>• Celiac disease<br>• Autoimmune pancreatitis	• Autoimmune thyroiditis (including Hashimoto thyroiditis)<br>• Grave's or Basedow's disease<br>• Diabetes mellitus type I<br>• Addison's disease<br>• Polyglandular autoimmune syndrome<br>• Autoimmune hypophysitis

When there is enough evidence to make any of the above diagnoses, the AE must be reported as a pIMD. Symptoms, signs or conditions which might (or might not) represent the above diagnoses, should be recorded and reported as AEs but not as pIMDs until the final or definitive diagnosis has been determined, and alternative diagnoses have been eliminated or shown to be less likely.

In order to facilitate the documentation of pIMDs in the eCRF, a pIMD standard questionnaire and a list of preferred terms (PTs) and PT codes corresponding to the above diagnoses will be available to investigators at study start.

Once a pIMD is diagnosed (serious or non-serious) in a study subject, the investigator (or designate) must complete, date and sign an electronic Expedited Adverse Events Report. The period for reporting pIMDs for the 1-Additional Dose, Revaccination and Control groups only will be from Visit Month 0 until 12 months (for Control and 1-Additional Dose groups) and from Visit Month 0 until 12 months after last HZ/su vaccination (for the Revaccination group) (see Table 6 and Table 26).

9.2. Events or outcomes not qualifying as adverse events or serious adverse events

9.2.1. Pregnancy

Female subjects who are pregnant or lactating at the time of vaccination must not receive additional doses of study vaccine but may continue other study procedures at the discretion of the investigator.

While pregnancy itself is not considered an AE or SAE, any adverse pregnancy outcome or complication or elective termination of a pregnancy for medical reasons will be recorded and reported as an AE or a SAE.

Note: The pregnancy itself should always be recorded on an electronic pregnancy report.

The following should always be considered as SAE and will be reported as described in Sections 9.4.1 and 9.4.3:

- Spontaneous pregnancy loss, including:
  - spontaneous abortion, (spontaneous pregnancy loss before/at 22 weeks of gestation),

- ectopic and molar pregnancy,
- stillbirth (intrauterine death of foetus after 22 weeks of gestation).

Note: the 22 weeks cut-off in gestational age is based on WHO-ICD 10 noted in the European Medicines Agency (EMA) Guideline on pregnancy exposure [EMA, 2006]. It is recognised that national regulations might be different.

- Any early neonatal death (i.e., death of a live born infant occurring within the first 7 days of life).
- Any congenital anomaly or birth defect (as per [Centers for Disease Control Metropolitan Atlanta Congenital Defects Program (CDC MACDP)] guidelines) identified in the offspring of a study subject (either during pregnancy, at birth or later) regardless of whether the foetus is delivered dead or alive. This includes anomalies identified by prenatal ultrasound, amniocentesis or examination of the products of conception after elective or spontaneous abortion.

Furthermore, any SAE occurring as a result of a post-study pregnancy AND considered by the investigator to be reasonably related to the investigational vaccine will be reported to GSK Biologicals as described in Section 9.4.3. While the investigator is not obligated to actively seek this information from former study participants, he/she may learn of a pregnancy through spontaneous reporting.

9.3. Detecting and recording adverse events, serious adverse events and pregnancies

9.3.1. Time period for detecting and recording adverse events, serious adverse events and pregnancies

The time period for collecting and recording SAEs will begin at the first study visit Month 0 and will end at the last study visit/ contact at the end of the study for each subject. See Section 9.4 for instructions on reporting of SAEs.

SAEs that are related to the investigational vaccine will be collected and recorded from the time of the first receipt of study vaccine until the subject is discharged from the study.

All AEs/SAEs leading to withdrawal from the study will be collected and recorded from the time of the first study visit Month 0 up to the last study visit/ contact at the end of the study*.

* If the AEs/SAEs leading to withdrawal from the study are not related to investigational vaccine, study participation or to GSK concomitant medication/vaccine, or if not due to HZ complications, only the name (diagnosis/description) of the event will be recorded in the eCRF. No other details about these type of AEs/SAEs will be recorded during the entire study (for the LTFU group), after 12 months from visit Month 0 (for the Control and 1-Additional Dose groups) and after 12 months from last HZ/su vaccination (for the Revaccination group).

In addition to the above-mentioned reporting requirements and in order to fulfil international reporting obligations, SAEs that are related to study participation (i.e., protocol-mandated procedures, invasive tests, a change from existing therapy) or are related to a concurrent GSK medication/vaccine will be collected and recorded from the time the subject consents to participate in the study until she/he is discharged from the study.

An overview of the protocol-required reporting periods for AEs/SAEs, IMCs and HZ complications for the LTFU group is given in Table 25. An overview of the protocol-required reporting periods for AEs/SAEs, IMCs and HZ complications as well as medically attended events and pIMDs for the 1-Additional Dose, Revaccination and Control groups, and pregnancies for the 1-Additional Dose and Revaccination groups as applicable, is given in Table 26.

Table 25 Reporting periods for collecting safety information (for the LTFU group)

Event [a]	Visit Month 0	Visit Year 1 - 5	Study conclusion visit/ contact Year 6
**Timing of reporting**	**Month 0**	**Month 12 - [illegible]**	**Month 72**
Reporting of SAEs related to investigational vaccine, related to study participation or to GSK concomitant medication/vaccine			
IMCs (see Section 7.7)			
HZ complications (see Section 4.4) (including SAE information) [b]			
Related AEs/SAEs leading to withdrawal from the study (see Section 9.3.1) [c]			

AE = Adverse Event; SAE = Serious Adverse Event; GSK = GlaxoSmithKline; IMC = Intercurrent Medical Condition; HZ = Herpes Zoster.

[a] The reporting period of all events starts from the time the subject consents to participate in the study.

[b] A HZ complication related to a HZ episode which was initiated during the primary study or during the interval between the end of ZOSTER-006/022 and beginning of the ZOSTER-049 study is to be recorded in general medical history at the first Month 0 Visit of study ZOSTER-049.

[c] If the AEs/SAEs are not related to investigational vaccine, study participation or to GSK concomitant medication/vaccine, or if not due to HZ complications, only the name diagnosis/description) of the event will be recorded in the eCRF. No other details about AEs/SAEs will be recorded.

Table 26 Reporting periods for collecting safety information (for the 1-Additional Dose, Revaccination and Control groups)

Event	Visit Month 0 Dose 1				Visit Month 2‡ Dose 2						Visit/ contact Year 1 - 5	Study conclusion visit/ contact Year 6
**Timing of reporting**	**Month 0**	**Day 6 post Dose 1**	**Day 29 post Dose 1**		**Month 2‡**	**Day 6 post Dose 2‡**	**Day 29 post Dose 2‡**		**Month 6**		**Month 12 - 60**	**Month 72**
Reporting of solicited AEs ¥												
Reporting of unsolicited AEs ¥												
Reporting of medically attended visits until 6 months after last HZ/su vaccination (see Section 9.1.5) [a]												
Reporting of pIMDs until 12 months after last HZ/su vaccination (see Section 9.1.6.1) [b]												
Reporting of pregnancies ¥ (see Section 9.2.1)												
Reporting of IMCs (see Section 7.7)												
Reporting of all SAEs until 12 months after last HZ/su vaccination [b]												
Reporting of SAEs related to investigational vaccine, related to study participation or concurrent GSK medication/vaccine [c]												

Event	Visit Month 0 Dose 1				Visit Month 2‡ Dose 2						Visit/ contact Year 1 - 5	Study conclusion visit/ contact Year 6
**Timing of reporting**	**Month 0**	**Day 6 post Dose 1**	**Day 29 post Dose 1**		**Month 2‡**	**Day 6 post Dose 2‡**	**Day 29 post Dose 2‡**		**Month 6**		**Month 12 - 60**	**Month 72**
HZ complications (see Section 4.4) (including SAE information) [d]												
AEs/SAEs leading to withdrawal from the study (see Section 9.3.1) [e]												

AE = Adverse Event; pIMDs = potential immune-mediated Diseases; IMC = Intercurrent Medical Condition; SAE = Serious Adverse Event; GSK = GlaxoSmithKline; HZ = Herpes Zoster.

‡ Visit Month 2 Dose 2 is only for the Revaccination group.

¥ Only for the 1-Additional Dose and Revaccination groups, as applicable.

[a] The reporting of medically attended visits will be from Visit Month 0 until 6 months (for the Control and 1-Additional Dose groups) and from Visit Month 0 until 6 months after last HZ/su vaccination (for the Revaccination group) (see Table 6).

[b] The reporting of pIMDs and all SAEs will be from Visit Month 0 until 12 months (for the Control and 1-Additional Dose groups) and from Visit Month 0 until 12 months after last HZ/su vaccination (for the Revaccination group) (see Table 6).

[c] The reporting period of these events starts from the time the subject consents to participate in the study.

[d] A HZ complication related to a HZ episode which was initiated during the primary study or during the interval between the end of ZOSTER-006/022 and beginning of the ZOSTER-049 study is to be recorded in general medical history at the first Month 0 Visit of study ZOSTER-049. HZ will be recorded in HZ-specific screens.

[e] Twelve months after visit Month 0 (for the Control and 1-Additional Dose groups) and after 12 months after last HZ/su vaccination (for the Revaccination group), if AEs/SAEs are not related to investigational vaccine, study participation or to GSK concomitant medication/vaccine, or if not due to HZ complications, only the name (diagnosis/description) of the event will be recorded in the eCRF. No other details about AEs/SAEs will be recorded.

9.3.2. Post-Study adverse events and serious adverse events

A post-study AE/SAE is defined as any event that occurs outside of the AE/SAE reporting period defined in Table 25 and Table 26. Investigators are not obligated to actively seek AEs or SAEs in former study participants. However, if the investigator learns of any SAE at any time after a subject has been discharged from the study, and he/she considers the event reasonably related to the investigational vaccine, the investigator will promptly notify the Study Contact for Reporting SAEs.

9.3.3. Evaluation of adverse events and serious adverse events

9.3.3.1. Active questioning to detect adverse events and serious adverse events

As a consistent method of collecting AEs, the subject should be asked a non-leading question such as:

'*Have you felt different in any way since receiving the vaccine or since the previous visit?*'

When an AE/SAE occurs, it is the responsibility of the investigator to review all documentation (e.g., hospital progress notes, laboratory and diagnostics reports) relative to the event. The investigator/ delegate will then record all relevant information regarding an AE/SAE in the eCRF. The investigator is not allowed to send photocopies of the subject's medical records to GSK Biologicals instead of appropriately completing the eCRF. However, there may be instances when copies of medical records for certain cases are requested by GSK Biologicals. In this instance, all subject identifiers will be blinded on the copies of the medical records prior to submission to GSK Biologicals.

The investigator will attempt to establish a diagnosis pertaining to the event based on signs, symptoms, and/or other clinical information. In such cases, the diagnosis should be documented as the AE/SAE and not the individual signs/symptoms.

9.3.3.2. Assessment of adverse events

9.3.3.2.1. Assessment of intensity

The intensity of the following solicited AEs will be assessed as described in Table 27.

Table 27 Intensity scales for solicited symptoms in adults

Adverse Event	Intensity grade	Parameter
Pain at injection site	0	None
	1	Mild: Any pain neither interfering with nor preventing normal every day activities.
	2	Moderate: Painful when limb is moved and interferes with every day activities.
	3	Severe: Significant pain at rest. Prevents normal every day activities.
Redness at injection site		Record greatest surface diameter in mm
Swelling at injection site		Record greatest surface diameter in mm
Fever*		Record temperature in °C/°F<br>Temperature will be analyzed in 0.5°C increments from ≥ 37.5°C
Headache	0	Normal
	1	Mild: Headache that is easily tolerated
	2	Moderate: Headache that interferes with normal activity
	3	Severe: Headache that prevents normal activity
Fatigue	0	Normal
	1	Mild: Fatigue that is easily tolerated
	2	Moderate: Fatigue that interferes with normal activity
	3	Severe: Fatigue that prevents normal activity
Gastrointestinal symptoms (nausea, vomiting, diarrhea and/or abdominal pain)	0	Normal
	1	Mild: Gastrointestinal symptoms that are easily tolerated
	2	Moderate: Gastrointestinal symptoms that interfere with normal activity
	3	Severe: Gastrointestinal symptoms that prevent normal activity
Myalgia	0	Normal
	1	Mild: Myalgia that is easily tolerated
	2	Moderate: Myalgia that interferes with normal activity
	3	Severe: Myalgia that prevents normal activity
Shivering	0	None
	1	Shivering that is easily tolerated
	2	Shivering that interferes with normal activity
	3	Shivering that prevents normal activity

* Fever is defined as temperature ≥ 37.5°C/99.5°F for oral, axillary or tympanic route, or ≥ 38.0°C/100.4°F for rectal route. The preferred route for recording temperature in this study will be oral.

The maximum intensity of local injection site redness/swelling will be scored at GSK Biologicals as follows using GSK Biologicals' standard grading scale based on the US Food and Drug Administration (FDA) guidelines for Toxicity Grading Scale for Healthy Adult and Adolescent Volunteers enrolled in Preventive Vaccine Clinical Trials [FDA, 2007]:

0	:	< 20 mm diameter
1	:	≥ 20 mm to ≤ 50 mm diameter
2	:	> 50 mm to ≤ 100 mm diameter
3	:	> 100 mm diameter

The preferred route for recording temperature in this study is oral.

Grade 3 fever will be defined as temperature > 39.0°C for oral, axillary or tympanic route and > 39.5°C for rectal route.

The investigator will assess the maximum intensity that occurred over the duration of the event for all unsolicited AEs (including SAEs) recorded during the study. The assessment will be based on the investigator's clinical judgement.

The intensity should be assigned to one of the following categories:

1 (mild)	=	An AE which is easily tolerated by the subject, causing minimal discomfort and not interfering with everyday activities.
2 (moderate)	=	An AE which is sufficiently discomforting to interfere with normal everyday activities.
3 (severe)	=	An AE which prevents normal, everyday activities.

An AE that is assessed as Grade 3 (severe) should not be confused with a SAE. Grade 3 is a category used for rating the intensity of an event; and both AEs and SAEs can be assessed as Grade 3. An event is defined as 'serious' when it meets one of the pre-defined outcomes as described in Section 9.1.2.

9.3.3.2.2. Assessment of causality

The investigator is obligated to assess the relationship between investigational vaccine and the occurrence of each AE/SAE. The investigator will use clinical judgement to determine the relationship. Alternative plausible causes, such as natural history of the underlying diseases, concomitant therapy, other risk factors, and the temporal relationship of the event to the investigational vaccine will be considered and investigated. The investigator will also consult the IB to determine his/her assessment.

There may be situations when a SAE has occurred and the investigator has minimal information to include in the initial report to GSK Biologicals. However, it is very important that the investigator always makes an assessment of causality for every event prior to submission of the Expedited Adverse Events Report to GSK Biologicals. The

investigator may change his/her opinion of causality in light of follow-up information and update the SAE information accordingly. The causality assessment is one of the criteria used when determining regulatory reporting requirements.

In case of concomitant administration of multiple vaccines/products, it may not be possible to determine the causal relationship of general AEs to the individual vaccine/product administered. The investigator should, therefore, assess whether the AE could be causally related to vaccination rather than to the individual vaccines.

All solicited local (injection site) reactions will be considered causally related to vaccination. Causality of all other AEs should be assessed by the investigator using the following question:

Is there a reasonable possibility that the AE may have been caused by the investigational vaccine?

YES	:	There is a reasonable possibility that the vaccine contributed to the AE.
NO	:	There is no reasonable possibility that the AE is causally related to the administration of the study vaccine. There are other, more likely causes and administration of the study vaccine is not suspected to have contributed to the AE.

If an event meets the criteria to be determined as 'serious' (see Section 9.1.2), additional examinations/tests will be performed by the investigator in order to determine ALL possible contributing factors for each SAE.

Possible contributing factors include:

- Medical history.
- Other medication.
- Protocol required procedure.
- Other procedure not required by the protocol.
- Other cause (specify).

9.3.3.3. Assessment of outcomes

The investigator will assess the outcome of all unsolicited AEs (including SAEs) recorded during the study as:

- Recovered/resolved.
- Recovering/resolving.
- Not recovered/not resolved.
- Recovered with sequelae/resolved with sequelae.
- Fatal (SAEs).

9.4. Reporting of serious adverse events, pregnancies and other events

9.4.1. Prompt reporting of serious adverse events, pregnancies and other events to GSK Biologicals

SAEs that occur in the time period defined in Section 9.3 will be reported promptly to GSK within the timeframes described in Table 28, once the investigator determines that the event meets the protocol definition of a SAE.

Pregnancies that occur in the time period defined in Section 9.3 will be reported promptly to GSK within the timeframes described in Table 28, once the investigator becomes aware of the pregnancy.

pIMDs that occur in the time period defined in Section 9.3 will be reported promptly to GSK within the timeframes described in Table 28, once the investigator determines that the event meets the protocol definition of a pIMD.

Table 28 Timeframes for submitting serious adverse events, pregnancy and other event reports to GSK Biologicals

Type of Event	Initial Reports		Follow-up of Relevant Information on a Previous Report	
	Timeframe	Documents	Timeframe	Documents
**SAEs**	24 hours*‡	electronic Expedited Adverse Events Report	24 hours*	electronic Expedited Adverse Events Report
**Pregnancies**	2 weeks*	electronic pregnancy report	2 weeks*	electronic pregnancy report
**pIMDs**	24 hours**‡	electronic Expedited Adverse Events Report	24 hours*	electronic Expedited Adverse Events Report

* Timeframe allowed after receipt or awareness of the information.
**Timeframe allowed once the investigator determines that the event meets the protocol definition of a pIMD.
‡ The investigator will be required to confirm review of the SAE/pIMD causality by ticking the 'reviewed' box in the electronic Expedited Adverse Events Report within 72 hours of submission of the SAE/pIMD.

9.4.2. Contact information for reporting serious adverse events, pregnancies and pIMDs

**Study Contact for Reporting SAEs, pregnancies and pIMDs**
**Refer to the local study contact information document.**
**Back-up Study Contact for Reporting SAEs, pregnancies and pIMDs**
24/24 hour and 7/7 day availability:<br><br>**GSK Biologicals Clinical Safety & Pharmacovigilance**<br>Outside US & Canada sites:<br>Fax: +32 2 656 51 16 or +32 2 656 80 09<br>Email address: Rix.CT-safety-vac@gsk.com<br><br>US sites only:<br>Fax: 1-610-787-7053<br><br>Canadian sites only:<br>Fax: 1-866-903-4718

9.4.3. Completion and transmission of SAE reports to GSK Biologicals

Once an investigator becomes aware that a SAE has occurred in a study subject, the investigator (or designate) must complete the information in the electronic Expedited Adverse Events Report WITHIN 24 HOURS. The report will always be completed as thoroughly as possible with all available details of the event. Even if the investigator does not have all information regarding a SAE, the report should still be completed within 24 hours. Once additional relevant information is received, the report should be updated WITHIN 24 HOURS.

The investigator will always provide an assessment of causality at the time of the initial report. The investigator will be required to confirm the review of the SAE causality by ticking the 'reviewed' box in the electronic Expedited Adverse Events Report within 72 hours of submission of the SAE.

9.4.3.1. Back-up system in case the electronic reporting system does not work

If the electronic reporting system does not work, the investigator (or designate) must complete, then date and sign a paper Expedited Adverse Events Report and fax it to the Study Contact for Reporting SAEs (refer to the Sponsor Information) or to GSK Biologicals Clinical Safety and Pharmacovigilance department within 24 hours.

This back-up system should only be used if the electronic reporting system is not working and NOT if the system is slow. As soon as the electronic reporting system is working again, the investigator (or designate) must complete the electronic Expedited Adverse Events Report within 24 hours. The final valid information for regulatory reporting will be the information reported through the electronic SAE reporting system.

9.4.4. Completion and transmission of pregnancy reports to GSK Biologicals

Once the investigator becomes aware that a subject is pregnant, the investigator (or designate) must complete the required information onto the electronic pregnancy report WITHIN 2 WEEKS.

Note: Conventionally, the estimated gestational age (EGA) of a pregnancy is dated from the first day of the last menstrual period (LMP) of the cycle in which a woman conceives. If the LMP is uncertain or unknown, dating of EGA and the estimated date of delivery (EDD) should be estimated by ultrasound examination and recorded in the pregnancy report.

9.4.5. Reporting of pIMDs to GSK Biologicals

Once a pIMD is diagnosed (serious or non-serious) in a study subject, the investigator (or designate) must complete the information in the electronic Expedited Adverse Events Report WITHIN 24 HOURS after he/she becomes aware of the diagnosis. The report allows to specify that the event is a pIMD and whether it is serious or non serious. The report will always be completed as thoroughly as possible with all available details of the event, in accordance with the pIMD standard questionnaire provided. Even if the investigator does not have all information regarding a pIMD, the report should still be completed within 24 hours. Once additional relevant information is received, the report should be updated WITHIN 24 HOURS.

The investigator will always provide an assessment of causality at the time of the initial report. The investigator will be required to confirm the review of the pIMD causality by ticking the 'reviewed' box in the electronic Expedited Adverse Events Report within 72 hours of submission of the pIMD.

Refer to Section 9.4.3.1 for back-up system in case the electronic reporting system does not work.

9.4.6. Updating of SAE, pregnancy, and pIMD information after removal of write access to the subject's eCRF

When additional SAE, pregnancy, or pIMD information is received after removal of the write access to the subject's eCRF, new or updated information should be recorded on the appropriate paper report, with all changes signed and dated by the investigator. The updated report should be faxed to the Study Contact for Reporting SAEs (refer to the Sponsor Information) or to GSK Biologicals Clinical Safety and Pharmacovigilance department within the designated reporting time frames specified in Table 28.

9.4.7. Regulatory reporting requirements for serious adverse events

The investigator will promptly report all SAEs to GSK in accordance with the procedures detailed in Section 9.4.1. GSK Biologicals has a legal responsibility to promptly notify, as appropriate, both the local regulatory authority and other regulatory agencies about the safety of a product under clinical investigation. Prompt notification of SAEs by the investigator to the Study Contact for Reporting SAEs is essential so that legal obligations and ethical responsibilities towards the safety of other subjects are met.

Investigator safety reports are prepared according to the current GSK policy and are forwarded to investigators as necessary. An investigator safety report is prepared for a SAE(s) that is both attributable to the investigational vaccine and unexpected. The purpose of the report is to fulfil specific regulatory and GCP requirements, regarding the product under investigation.

9.5. Follow-up of adverse events, serious adverse events, and pregnancies

9.5.1. Follow-up of adverse events and serious adverse events

9.5.1.1. Follow-up during the study

After the initial AE/SAE report, the investigator is required to proactively follow each subject and provide additional relevant information on the subject's condition to GSK Biologicals (within 24 hours for SAEs; refer to Table 28).

All SAEs and pIMDs (serious or non-serious) documented at a previous visit/ contact and designated as not recovered/not resolved or recovering/resolving will be reviewed at subsequent visits/contacts until the end of the study.

All AEs documented at a previous visit/contact and designated as not recovered/not resolved or recovering/resolving will be reviewed at subsequent visits/contacts until 30 days after the last vaccination.

Cases of new onset of autoimmune diseases and other immune-mediated inflammatory disorders documented at a previous visit/contact and designated as not recovered/not resolved or recovering/resolving will be reviewed at subsequent visits/contacts until study conclusion.

9.5.1.2. Follow-up after the subject is discharged from the study

The investigator will follow subjects:

- with SAEs, pIMDs (serious or non-serious), or subjects withdrawn from the study as a result of an AE, until the event has resolved, subsided, stabilised, disappeared, or until the event is otherwise explained, or the subject is lost to follow-up.
- with other non-serious AEs, cases of new onset of autoimmune diseases, until study conclusion or they are lost to follow-up.

If the investigator receives additional relevant information on a previously reported SAE, he/she will provide this information to GSK Biologicals using a paper/ electronic Expedited Adverse Events Report and/or pregnancy report as applicable.

GSK Biologicals may request that the investigator performs or arranges the conduct of additional clinical examinations/tests and/or evaluations to elucidate as fully as possible the nature and/or causality of the AE or SAE. The investigator is obliged to assist. If a subject dies during participation in the study or during a recognised follow-up period, GSK Biologicals will be provided with any available post-mortem findings, including histopathology.

9.5.2. Follow-up of pregnancies

Pregnant subjects will be followed to determine the outcome of the pregnancy. At the end of the pregnancy, whether full-term or premature, information on the status of the mother and child will be forwarded to GSK Biologicals using the electronic pregnancy report and the Expedited Adverse Events Report if applicable. Generally, the follow-up period doesn't need to be longer than six to eight weeks after the estimated date of delivery.

Regardless of the reporting period for SAEs for this study, if the pregnancy outcome is a SAE, it should always be reported as SAE.

9.6. Treatment of adverse events

Treatment of any AE is at the sole discretion of the investigator and according to current good medical practice. Any medication administered for the treatment of an AE should be recorded in the subject's eCRF (refer to Section 7.6.1).

9.7. Subject card

Study subjects/ subject's caregiver must be provided with the address and telephone number of the main contact for information about the clinical study.

The investigator (or designate) must therefore provide a "subject card" to each subject/ subject's caregiver. In an emergency situation this card serves to inform the responsible attending physician that the subject is in a clinical study and that relevant information may be obtained by contacting the investigator.

Subjects/ subjects' caregiver must be instructed to keep subject cards in their possession at all times.

10. SUBJECT COMPLETION AND WITHDRAWAL

10.1. Subject completion

A subject who returns for the concluding visit/ contact foreseen in the protocol is considered to have completed the study.

10.2. Subject withdrawal

Withdrawals will not be replaced.

10.2.1. Subject withdrawal from the study

From an analysis perspective, a 'withdrawal' from the study refers to any subject who did not come back for the concluding visit/ contact foreseen in the protocol.

All data collected until the date of withdrawal/last contact of the subject will be used for the analysis.

A subject is considered a 'withdrawal' from the study when no study procedure has occurred, no follow-up has been performed and no further information has been collected for this subject from the date of withdrawal/last contact.

Investigators will make an attempt to contact those subjects who do not return for scheduled visits or follow-up.

Information relative to the withdrawal will be documented in the eCRF. The investigator will document whether the decision to withdraw a subject from the study was made by the subject himself/herself, or by the investigator, as well as which of the following possible reasons was responsible for withdrawal:

- Serious adverse event.
- Non-serious adverse event.
- HZ episode.
- Protocol violation (specify).
- Consent withdrawal, not due to an adverse event*.
- Moved from the study area.
- Lost to follow-up.
- Other (specify).

*In case a subject is withdrawn from the study because the subject has withdrawn consent, the investigator will document the reason for withdrawal of consent, if specified by the subject in the eCRF.

Subjects who are withdrawn from the study because of SAEs/AEs must be clearly distinguished from subjects who are withdrawn for other reasons. Investigators will follow subjects who are withdrawn from the study as result of a SAE/AE until resolution of the event (see Section 9.5.1.2).

10.2.2. Subject withdrawal from investigational vaccine

A 'withdrawal' from the investigational vaccine refers to any subject who does not receive the complete treatment, i.e., when no further planned dose is administered from the date of withdrawal. A subject withdrawn from the investigational vaccine may not necessarily be withdrawn from the study as further study procedures or follow-up may be performed (safety or immunogenicity) if planned in the study protocol.

All data collected until the date of withdrawal/last contact of the subject will be used for the analysis.

Information relative to premature discontinuation of the investigational vaccine will be documented on the Vaccine Administration screen of the eCRF. The investigator will document whether the decision to discontinue further vaccination was made by the subject himself/herself or by the investigator, as well as which of the following possible reasons was responsible for withdrawal:

- Serious adverse event.
- Non-serious adverse event.
- Other (specify).

11. STATISTICAL METHODS

11.1. Primary endpoint

- Confirmed HZ cases (LTFU and Control groups);
  - Confirmed HZ cases during the ZOSTER-049 study.

11.2. Secondary endpoints

- Confirmed HZ cases;
  - Confirmed HZ cases since one month post dose 2 in the previous ZOSTER-006/022 studies;
- PHN cases;
  - PHN cases during the ZOSTER-049 study and since one month post dose 2 in the previous ZOSTER-006/022 studies;
- HZ related complications (other than PHN);
  - HZ related complications (other than PHN) during the ZOSTER-049 study and since one month post dose 2 in the previous ZOSTER-006/022 studies;
- Antigen-gE humoral immunogenicity at Months 0, 12, 24, 36, 48, 60 and 72 (LTFU HI subset, 1-Additional Dose, Revaccination and Control groups), at Month 1 (1-Additional Dose, Revaccination and Control groups), and at Month 3 (Revaccination and Control groups);
  - Anti-gE Ab concentrations as determined by ELISA;
- CMI in terms of frequencies of antigen-specific CD4+ T cells at Months 0, 12, 24, 36, 48, 60 and 72 (LTFU CMI subset, 1-Additional Dose, Revaccination and Control groups), at Month 1 (1-Additional Dose, Revaccination and Control groups), and at Month 3 (Revaccination and Control groups);
  - Frequencies of CD4+ T cells with antigen-specific Interferon gamma (IFN-γ) and/or Interleukin-2 (IL-2) and/or Tumour Necrosis Factor alpha (TNF-α) and/or CD40 Ligand (CD40L) secretion/expression to gE as determined by ICS;
- Solicited local and general symptoms in subjects administered with 1 or 2 additional doses of HZ/su vaccine (1-Additional Dose and Revaccination groups);
  - Occurrence, intensity and duration of each solicited local symptom within 7 days (Days 0 – 6) after each vaccination;
  - Occurrence, intensity, duration and relationship to vaccination of each solicited general symptom within 7 days (Days 0 – 6) after each vaccination;

- Unsolicited AEs in subjects administered with 1 or 2 additional doses of HZ/su vaccine (1-Additional Dose and Revaccination groups);
  - Occurrence, intensity and relationship to vaccination of unsolicited AEs during 30 days (Days 0 – 29) after each vaccination, according to the Medical Dictionary for Regulatory Activities (MedDRA) classification;
- Serious AEs;
  - Occurrence and relationship to vaccination of all SAEs;
  - from Visit Month 0 until 12 months: for Control and 1-Additional Dose groups;
  - from Visit Month 0 until 12 months after last HZ/su vaccination: for the Revaccination group;
  - Occurrence of SAEs related to investigational vaccine, related to study participation or to GSK concomitant medication/vaccine during the entire study period in all subjects;
- Occurrence of AEs of specific interest: Potential immune-mediated diseases (pIMDs) (1-Additional Dose, Revaccination and Control groups);
  - Occurrence and relationship to vaccination of all pIMDs;
  - from Visit Month 0 until 12 months: for Control and 1-Additional Dose groups;
  - from Visit Month 0 until 12 months after last HZ/su vaccination: for the Revaccination group.

11.3. Tertiary endpoints

CCI [redacted]

11.4. Determination of sample size

In ZOSTER-006, 7344 subjects have been included in the modified Total Vaccinated cohort (mTVc) for the analysis in that study and will be eligible to be included in this LTFU efficacy analysis. Considering that about 50% of the subjects may not come back for the ZOSTER-049 study, we could expect that 3672 subjects will be willing and eligible to participate.

In ZOSTER-022, 13,500 subjects have been vaccinated with at least one dose of HZ/su vaccine or one dose of placebo. Considering that 90% will be evaluable for the mTVC, we could expect that 6075 subjects from the vaccinated group will be eligible to be included in the LTFU efficacy analysis. Considering that 55% of the subjects may not come back for the ZOSTER-049 study, we could expect that 2734 subjects will be willing and eligible to participate.

Thus in total, 6406 evaluable subjects can be expected in this LTFU efficacy analysis.

11.4.1. VE assessment

Considering that 240 subjects will be randomised within the groups 1-Additional Dose, Revaccination and Control, approximately 6000 subjects would be expected to be evaluable for the efficacy assessment in the mTVc.

The sample size will allow us to get a Lower Limit (LL) of 95% CI (95%LL) of the VE above 30% overall over the study with a probability of at least 99%.

Because no placebo group is enrolled in this study, an approximate power computation has been derived considering the incidence rate in the vaccine group as compared to the incidence rate of the historical control from the ZOSTER-006/022 studies.

For each year of VE exploratory assessment CCI [redacted]

CCI [redacted]

CCI [redacted]

CCI [redacted]

11.5. Cohorts for Analyses

11.5.1. Total vaccinated cohort

The Total Vaccinated cohort (TVc) will include all subjects from the ZOSTER-006/022 studies who are enrolled in the ZOSTER-049.

The TVc for the groups 1-Additional Dose and Revaccination will include all vaccinated subjects from the ZOSTER-006/022 studies that received at least one additional dose of HZ/su vaccine in the ZOSTER-049 study.

The TVc for analysis of efficacy and immunogenicity will include subjects for whom data related to efficacy and immunogenicity endpoints are available.

The TVc for analysis of safety will include all subjects with at least one vaccine dose administered.

11.5.2. Modified Total Vaccinated cohort (LTFU and Control group)

The mTVc will be the primary population for efficacy analysis, which excludes subjects in the TVc for efficacy analysis who were not administered with the second vaccination during the ZOSTER-006/022 study, or who developed a confirmed case of HZ prior to 1 month after the second vaccination in the ZOSTER-006/022 primary study.

11.5.3. According To Protocol cohort for analysis of efficacy (LTFU and Control groups)

The According To Protocol (ATP) cohort for analysis of efficacy will include all evaluable subjects:

- Who meet all eligibility criteria;
- Who have received 2 doses of the HZ/su study vaccine according to their random assignment in the primary ZOSTER-006/022 studies;
- For whom administration site of study vaccine is known and correct from ZOSTER-006/022;
- Who have not received a vaccine not specified or forbidden in the protocol;
- Complying with the procedures defined in the protocol;
- Who did not receive a medication/ product leading to elimination from the ATP analysis;
- Who did not present with an IMC leading to exclusion from an ATP analysis;
- Who complied with the vaccination schedule, *i.e.,* 49-83 days between the first and the second dose in ZOSTER-006/022;
- Who did not develop a confirmed case of HZ prior to 1 month (30 days) after the second vaccination, during the ZOSTER-006/022 studies.

11.5.4. According To Protocol cohort for analysis of immunogenicity (1-Additional Dose, Revaccination and Control groups)

The ATP cohort for analysis will include all subjects from the TVc:

- Who meet all eligibility criteria;
- Who have received 1 dose (1-Additional Dose group) or 2 doses (Revaccination group) of study vaccine(s) according to their random assignment;
- For whom administration site of study vaccine is known and correct;
- Who have not received a vaccine not specified or forbidden in the protocol;
- Who complied with the procedures defined in the protocol;
- Who did not receive a medication/ product leading to exclusion from an ATP analysis;
- Who did not present with an IMC leading to exclusion from an ATP analysis;
- Who complied with the vaccination schedule, *i.e.,* 49-83 days between the first and the second dose for the Revaccination group;
- Who complied with the Month 1 blood sample schedule, *i.e.,* 28-48 days from Month 0 to Month 1 for the 1-Additional Dose group and Control group;

- Who complied with the post-Dose 2 blood sample schedule, *i.e.*, 28-48 days post-Dose 2 for the Revaccination group;
- Who had immunogenicity results available post-dose 1 for the 1-Additional Dose group, ,post dose 2 for the Revaccination group and at Month 1 and Month 3 for the Control group;
- Who had no episode of HZ prior to study start;
- Who received 2 doses of HZ/su vaccine in the primary ZOSTER-006/022 studies.

11.5.5. According To Protocol cohort for analysis of persistence of immunogenicity (LTFU group)

The ATP cohort for analysis of persistence of immunogenicity (humoral/CMI) will include all evaluable subjects i.e., those who were included in the ATP cohort for immunogenicity (humoral/CMI) in the primary ZOSTER-006/022 studies, or were excluded from this cohort solely because they had no blood samples taken, or because of incompliance with blood sample schedule, and:

- Who did not receive a concomitant medication/ product leading to elimination from the ATP analysis for immunogenicity up to the timepoint considered (see Section 7.6.2);
- Who did not present with an IMC leading to elimination from the ATP analysis for immunogenicity (including HZ infection) up to the timepoint considered (see Section 7.7);
- For whom persistence immunogenicity results are available for the considered time point.

11.5.6. According To Protocol cohort for analysis of persistence of immunogenicity (1-Additional Dose, Revaccination and Control groups)

The ATP cohort for analysis of persistence of immunogenicity will include all evaluable subjects i.e., those who were included in the ATP cohort for analysis of immunogenicity in the ZOSTER-049 study, or were excluded from this cohort solely because they had no blood samples taken or because of incompliance with blood sample schedule, and:

- Who did not receive a concomitant medication/ product leading to elimination from the ATP analysis for immunogenicity up to the timepoint considered (see Section 7.6.2);
- Who did not present with an IMC leading to elimination from the ATP analysis for immunogenicity (including HZ infection) up to the timepoint considered (see Section 7.7);
- For whom persistence immunogenicity results are available for the timepoint considered.

11.6. Derived and transformed data

11.6.1. Handling of missing data

For a given subject and a given measurement, missing or non-evaluable measurements will not be imputed. The missing endpoint and censoring are supposed to occur independently, and the pattern of the missing value(s) being either Missing Completely At Random (MCAR) or Missing At Random (MAR) only.

For the analysis of solicited symptoms, missing or non-evaluable measurements will not be replaced. Therefore, the analysis of the solicited symptoms based on the TVc will include only subjects/doses with documented safety data (i.e., symptom screen/sheet completed).

For the analysis of unsolicited symptoms/SAEs/pIMDs/concomitant medication, all vaccinated subjects will be considered and subjects who did not report an event will be considered as subjects without an event.

For the analysis of immunogenicity, missing or non-evaluable measurements will not be replaced. Therefore, a subject will be excluded from an analysis if all measurements are missing or non-evaluable.

11.6.2. Humoral immune response

A seronegative subject is a subject whose Ab concentration is below the cut-off value.

A seropositive subject is a subject whose Ab concentration is greater than or equal to the cut-off value.

The seropositivity rate is defined as the percentage of seropositive subjects.

The Vaccine Response Rate (VRR) for anti-gE is defined as the percentage of subjects who have at least:

- a 4-fold increase in the post-dose 2 anti-gE Ab concentration as compared to the prevaccination anti-gE Ab concentration, for subjects who are seropositive at baseline, or,
- a 4-fold increase in the post-dose 2 anti-gE Ab concentration as compared to the anti-gE Ab cut-off value for seropositivity, for subjects who are seronegative at baseline.

The GMC calculations are performed by taking the anti-log of the mean of the log concentration transformations. Ab concentrations below the cut-off of the assay will be given an arbitrary value equal to half the cut-off for the purpose of GMC calculation.

The Mean Geometric Increase (MGI) is defined as the geometric mean of the within subject ratios of two different timepoints.

11.6.3. CMI response

- For the descriptive analyses, the frequency of CD4[2+] T-cells upon in vitro stimulation with the gE-antigen (induction condition) is calculated by dividing the number of activated CD4[2+] T-cells (numerator) over the total number of CD4 T-cells involved (denominator). The same calculation will be performed for the frequency computation for any kinds of cells and for each individual activation marker as appropriate.

$$Freq_{Induction}^{CD4[2+]} = \frac{n_{Induction}^{2+}}{N_{Induction}^{CD4}}$$

$n_{Induction}^{2+}$ = number of CD4 T – cells secreting at least 2 activation markers after induction with the antigen

N^{CD4} = Total number of CD4 T – cells involved in the assay (induction)

- The frequency of gE-specific CD4 T-cells for each individual subject is calculated as the difference between the frequency of CD4[2+] T-cells, upon in vitro stimulation with the gE antigen (induction condition) minus the frequency of CD4[2+] T-cells upon in vitro stimulation in medium only (background condition). The differences less or equal to one are imputed to one gE-specific CD4[2+] T-cell per 10^6 CD4+ T-cells. The same calculation will be performed for the frequency computation for any kind of cells and for each individual activation marker as appropriate.

$$Freq_{Specific}^{CD4[2+]} = \frac{n_{Induction}^{2+}}{N_{Induction}^{CD4}} - \frac{n_{Background}^{2+}}{N_{Background}^{CD4}} \qquad if \ \frac{n_{Induction}^{2+}}{N_{Induction}^{CD4}} > 1 + \frac{n_{Background}^{2+}}{N_{Background}^{CD4}}$$

$$Freq_{Specific}^{CD4[2+]} = 1 \qquad if \ \frac{n_{Induction}^{2+}}{N_{Induction}^{CD4}} \leq 1 + \frac{n_{Background}^{2+}}{N_{Background}^{CD4}}$$

$n_{Induction}^{2+}$ = number of CD4 T - cells secreting at least 2 activation markers after induction with the gE - antigen

$n_{Background}^{2+}$ = number of CD4 T - cells secreting at least 2 activation markers in the medium conditions

N^{CD4} = Total number of CD4 T - cells involved in the assay (induction of background)

- The GM frequency calculations are performed by taking the anti-log of the mean of the log frequency transformations.
- The CMI vaccine response to gE will be based on the gE-specific data. The VRR is defined as the percentage of subjects who have at least:
  - a 2-fold increase as compared to the threshold 320 polypositive CD4+ T cells/10^6 CD4+ T cells, for subjects with pre-vaccination T-cell frequencies below the threshold 320 polypositive CD4+ T cells/10^6 CD4+ T cells;
  - a 2-fold increase as compared to pre-vaccination T-cell frequencies, for subjects with pre-vaccination above the threshold 320 polypositive CD4+ T cells/10^6 CD4+ T cells.
- CCI [redacted]

11.7. Statistical analyses

11.7.1. Analysis of demographics (for each of the four groups of the study)

Demographic characteristics (age at first vaccination in the ZOSTER-006/022 studies, gender, geographic ancestry, and ethnicity), cohort description and withdrawal status will be summarised by group.

The mean age at first vaccination in the ZOSTER-006/022 studies (plus range and standard deviation) of the enrolled subjects, as a whole, and stratified by age group will be calculated.

In addition, for the 1-Additional Dose, Revaccination and Control groups, the mean age at Month 0 in the ZOSTER-049 (plus range and standard deviation) of the enrolled subjects, as a whole, and by age group (50-59, 60-69 and ≥ 70 YOA) will be calculated.

The distribution of subjects enrolled among the study sites will be tabulated.

Frequency tables will be generated for categorical variables such as gender.

Mean, median and standard error will be provided for continuous data such as age.

11.7.2. Analysis of efficacy (LTFU and Control groups)

The primary analysis of efficacy will be performed on the mTVc, the analysis will be also performed on the TVc and the ATP for efficacy to complement the mTVc analysis.

The analysis on mTVc and ATP cohort for efficacy will be performed over the follow-up time from one month post Dose 2 to the confirmed HZ case for a subject with a confirmed HZ case, and to the date of last contact for subjects without a confirmed HZ case.

The analysis on the TVc will be performed over the complete follow-up period and will include confirmed HZ cases from Dose 1 for vaccine efficacy evaluation. The end of the follow-up period will be calculated according to the same principle as will be done for the mTVc and ATP analyses for efficacy, i.e., up to the confirmed HZ case for a subject with a confirmed HZ case, or up to the date of last contact for subjects without a confirmed HZ case.

All analyses will be performed overall and by age strata. The age strata for reporting purposes are 50-59, 60-69, and, ≥ 70 YOA at primary vaccination. When overall VE is presented, the age factor will include the 3 main age strata 50-59, 60-69, and ≥ 70 YOA. When VE by age is presented, the same model will be run using only the data pertaining to the strata under consideration.

In addition, the analysis will be performed on subjects ≥ 60 YOA at inclusion in the ZOSTER-006/022 studies.

The primary analysis will be to assess the VE during this study and will use the historical control estimates adjusted for age at randomisation during the ZOSTER-006/022 studies. The method using the historical control estimates will be further explained in the Statistical Analysis Plan (SAP). Vaccine effects will be estimated by calculating the VE and estimating 95% CIs based on the variance of the observed population in this study and treating the historical control as constant.

The secondary analysis will be to assess the VE over each year of follow-up. Results from the ZOSTER-006/022 studies will be pooled for each year after vaccination with methods used in these studies. For overlapping years between ZOSTER-006/022 studies and this study, all the data will be pooled. For the non-overlapping years only the data from this ZOSTER-049 study will be used.

The assessment of the VE over the overall period including period from vaccination during the ZOSTER-006/022 studies up to the end of this long-term study will be done with a method that will be described in the SAP.

Any available information for the diagnosis of HZ episodes (clinical diagnosis, PCR, laboratory methods, etc.) during the interval between the end of the ZOSTER-006/022 studies and beginning of the ZOSTER-049 study is to be collected. Confirmation of all of these cases in the exact way as what will be done for cases collected during the clinical study follow-up periods of the ZOSTER-006, ZOSTER-022 and ZOSTER-049 will not be possible. Therefore, these cases will not be part of all efficacy analyses. A sensitivity analysis to assess the impact of these cases on the VE will be performed (this will be described in the SAP).

The purpose of these analyses will be descriptive and therefore the CIs will not be adjusted for multiple testing.

The analyses will be performed for confirmed HZ cases, for PHN and for complications (other than PHN).

Any exploratory or sensitivity analysis may be performed in addition to the analyses described above on an ad-hoc basis. However, the significance level of those analyses may not be fully controlled.

11.7.3. Analysis of immunogenicity

The primary analysis will be based on the ATP cohort. If, in any vaccine group the percentage of vaccinated subjects with serological results excluded from the ATP cohort for analysis of immunogenicity is 5% or more, a second analysis based on the TVc will be performed to complement the ATP analysis.

All analyses will be presented overall and by age strata. The main age strata for reporting purposes are 50-59, 60-69 and ≥ 70 YOA. Additional analysis will be presented in ≥ 60 YOA.

11.7.3.1. Assessment for 1-Additional Dose, Revaccination and Control groups

11.7.3.1.1. Humoral immune response

Descriptive analysis – within group:

The following parameters will be tabulated by vaccine group at each time point when a blood sample result is available:

- Seropositivity rate with exact 95% CI;
- GMC with 95% CI;
- The MGI Post Month 1 and Post Month 3 current study over pre-vaccination in the primary studies with 95% CI;
- The MGI Post Month 3 primary vaccination over the Post Month 1 and Post Month 3 current study with 95% CI;
- The MGI Post Month 1 and Post Month 3 current study over Visit Month 0, in the current study with 95% CI;
- VRR [post additional dose groups over pre-vaccination in the primary studies] with exact 95% CI;
- The distribution of Ab titres will be tabulated and also presented using reverse cumulative curves.

CCI [redacted]

Persistence analysis

Persistence data will be analysed from the Visit Year 1 to the Visit Year 6 timepoint after vaccination.

For each year X (X varying from year 1 to year 6): The analysis of Ab persistence will be based on the ATP cohort for persistence at year X –adapted for each timepoint. If the percentage of subjects, excluded from the ATP cohort for the Year X follow-up serological results, is higher than 5% for any group, a second analysis based on the TVc at Year X will be performed to complement the ATP analysis.

The following parameters will be tabulated by group at each timepoint when a blood sample result is available:

- Seropositivity rate with exact 95% CI;
- GMC with 95% CI;
- The MGI Post Month 3 primary vaccination over the Post year x for the 1-Additional Dose or Revaccination group with 95% CI;
- The MGI Post year x for the 1-Additional Dose or Revaccination group over Visit Month 0, in the current study with 95% CI;
- The MGI Post year x over pre-vaccination in the primary ZOSTER-006/022 studies with 95% CI;
- VRR [post year x (1-Additional Dose or Revaccination group) over pre-vaccination in the primary ZOSTER-006/022 studies] with exact 95% CI;
- The distribution of Ab titres will be tabulated and also presented using reverse cumulative curves.

11.7.3.1.2. Cell-mediated immune response

Descriptive statistics (N, mean, SD, min, Q1, median, Q3, max) of the following parameters will be tabulated by group at all timepoints:

- Descriptive statistics of the frequency of CD4+ T-cells expressing at least two activation markers (among IFN-γ, IL-2, TNF-α, CD40L) for gE-specific stimulation.
- CCI [redacted].

11.7.3.2. Analysis on subjects with HZ cases

The following analysis will be also performed at each time point when a blood sample result is available on subjects who had a HZ case:

- Seropositivity rate with exact 95% CI;
- GMC with 95% CI.

11.7.3.3. Analysis of persistence (immune subset of LTFU group)

Since the interval between the end of ZOSTER-006/022 studies and the start of this study will vary per subject and is dependent on receipt of approval or implementing the study in the different participating countries, some results from the first blood sample may correspond to year 5, year 6 or year 7 post vaccination. To be able to complement the modelling prediction by descriptive data analysis, yearly timeframes will be defined around the anniversary date post vaccination. The details of the set up for these intervals will be described in the SAP.

11.7.3.3.1. Humoral immune response

For the yearly prediction of the GMCs (year 5 to year 10 and beyond): the analysis of Ab persistence will be based on the ATP cohort for persistence adapted for each year.

In order to assess the persistence at Year 5, 6, 7, 8, 9 and 10 and beyond after the primary vaccination in the ZOSTER-006/022 studies, a longitudinal analysis will be performed and will include all results from ZOSTER-006/022 studies and all results from this ZOSTER-049 study.

The following parameters will be tabulated by vaccine group for all timeframes (Refer to the SAP):

- Seropositivity rate with exact 95% CI;
- GMC with 95% CI;
- Vaccine response rate with exact 95% CI;
- MGI from baseline with 95% CI for anti-gE;
- The distribution of Ab titres will be tabulated and also presented using reverse cumulative curves.

Note that the baseline that will be used for the computation of MGI and VRR will be the concentration obtained at pre-vaccination in the primary studies.

11.7.3.3.2. Cell-mediated immune response

For the yearly prediction of the CMI (year 5 to year 10 and beyond): the analysis of CMI persistence will be based on the ATP cohort for persistence adapted for each year.

In order to assess the persistence at Year 5, 6, 7, 8, 9 and 10 and beyond after the primary vaccination in the ZOSTER-006/022, a longitudinal analysis will be performed and will include all results from ZOSTER-006/022 studies and all results from this ZOSTER-049 study.

Descriptive statistics (N, mean, SD, min, Q1, median, Q3, max) of the following parameters will be tabulated by group for all timeframes (Refer to the SAP):

- Descriptive statistics of the frequency of CD4+ T-cells secreting at least two activation markers (from among IFN-γ, IL-2, TNF-α, CD40L) for gE-specific stimulation.

The following parameters will be tabulated by vaccine group for all timeframes when a blood sample result is available:

- Vaccine response rate with exact 95% CI.
- Note that the baseline that will be used for the computation of VRR will be the concentration obtained at pre-vaccination in the primary studies.

11.7.4. Analysis of safety

The analysis will be performed on the TVC.

The results for the analysis of safety will be tabulated as:

- The percentage of subjects with at least one local solicited AE, with at least one general solicited AE and with any solicited AEs during the solicited 7-day follow-up period will be tabulated with exact 95% CI after each vaccine dose and overall (1-Additional Dose and Revaccination groups). The same tabulation will be performed for grade 3;
- The percentage of subjects reporting each individual solicited local and general AE during the solicited 7-day follow-up period will be tabulated with exact 95% CI (1-Additional Dose and Revaccination groups). For all solicited symptoms, the same tabulation will be performed for grade 3 solicited AEs and for solicited general AEs with relationship to vaccination (1-Additional Dose and Revaccination groups);
- The percentage of subjects reporting temperature by half degree (°C) cumulative increments. Similar tabulations will be performed for any fever with a causal relationship to vaccination and for any fever resulting in a medically attended visit (1-Additional Dose and Revaccination groups);
- The proportion of subjects with at least one report of unsolicited AE classified by the MedDRA Preferred Terms and reported up to 30 days after each vaccination will be tabulated with exact 95% CI (1-Additional Dose and Revaccination groups);
- The same tabulation will be performed for grade 3 unsolicited AEs and for unsolicited AEs with a relationship to vaccination. The proportion of AEs resulting in a medically attended visit will also be tabulated (1-Additional Dose and Revaccination groups);
- Total number/percentages of doses (per dose and overall) followed by AEs will be tabulated (1-Additional Dose and Revaccination groups);
- Number of subjects with pIMDs will be tabulated (1-Additional Dose, Revaccination and Control groups);
- SAEs related to investigational vaccine (all groups);
- SAEs related to study participation or to GSK concomitant medication/vaccine (all groups);
- Adverse events (AEs)/SAEs leading to withdrawal from the study (all groups);
- IMCs (all groups);
- HZ complications (all groups).

11.8. Interpretation of analyses

Long term efficacy part:

All the analyses will be descriptive with the aim to characterise the long-term efficacy.

Additional dose and revaccination part:

All the analyses will be descriptive with the aim to characterise the effect of 1 and 2 additional doses of HZ/su vaccine.

11.9. Conduct of analyses

Any deviation(s) or change(s) from the original statistical plan outlined in this protocol will be described and justified in the final study report.

11.9.1. Sequence of analyses

The analysis will be performed in the following steps:

1. A final analysis on:
   - immunogenicity and safety (SAEs and pIMDs) data in (1-Additional dose, Revaccination and Control groups), and;
   - reactogenicity data (1-Additional Dose and Revaccination groups),

   will be performed when all those data up to Visit Month 3 are available and as clean as possible. Because the analysis is purely descriptive, no adjustment on type I error is foreseen. No clinical study report is planned to be written at this time and at this point no individual data listings will be provided. At this point, the GSK central clinical team will have access to the lab data and the treatment assignment from SBIR.
2. Two intermediate analyses to assess the VE at year 2 and year 4 (LTFU and Control groups):
   - An assessment of the VE and immunogenicity will be performed when the last subjects have completed their year 2 visit/contact and when efficacy data (on confirmed HZ cases), immunogenicity data as well as related SAEs up to year 2 are available and as clean as possible. Because the analysis is purely descriptive, no adjustment on type I error is foreseen. A clinical study report is planned to be written at this time but no individual data listings will be provided. Please refer to the SAP.
   - An assessment of the VE and immunogenicity will be performed when the last subjects have completed their year 4 visit/contact and when efficacy data (on confirmed HZ cases), immunogenicity data as well as related SAEs up to year 4 are available and as clean as possible. Because the analysis is purely descriptive, no adjustment on type I error is foreseen. A clinical study report is planned to be written at this time but no individual data listings will be provided. Please refer to the SAP.

3. End of study analysis:
   - The end of study analysis will be performed at the end of study. This end of study analysis will combine both of the previous analyses, as well as analyses up to the study end. Individual data listings will be generated at this stage. An integrated end-of-study report will be written.

11.9.2. Statistical considerations for interim analyses

Because the analysis is purely descriptive, no adjustment on type I error is foreseen.

12. ADMINISTRATIVE MATTERS

To comply with ICH GCP administrative obligations relating to data collection, monitoring, archiving data, audits, confidentiality and publications must be fulfilled.

12.1. electronic Case Report Form instructions

A validated GSK defined electronic data collection tool will be used as the method for data collection.

In all cases, subject initials will not be collected nor transmitted to GSK. Subject data necessary for analysis and reporting will be entered/transmitted into a validated database or data system. Clinical data management will be performed in accordance with applicable GSK standards and data cleaning procedures.

While completed eCRFs are reviewed by a GSK Biologicals' Site Monitor at the study site, omissions or inconsistencies detected by subsequent eCRF review may necessitate clarification or correction of omissions or inconsistencies with documentation and approval by the investigator or appropriately qualified designee. In all cases, the investigator remains accountable for the study data.

The investigator will be provided with a CD-ROM of the final version of the data generated at the investigational site once the database is archived and the study report is complete and approved by all parties.

12.2. Study Monitoring by GSK Biologicals

GSK will monitor the study to verify that, amongst others, the:

- Data are authentic, accurate, and complete.
- Safety and rights of subjects are being protected.
- Study is conducted in accordance with the currently approved protocol, any other study agreements, GCP and all applicable regulatory requirements.

The investigator and the head of the medical institution (where applicable) agrees to allow the monitor direct access to all relevant documents.

The investigator must ensure provision of reasonable time, space and qualified personnel for monitoring visits.

Direct access to all study-site related and source data is mandatory for the purpose of monitoring review. The monitor will perform a eCRF review and a Source Document Verification (SDV). By SDV we understand verifying eCRF entries by comparing them with the source data that will be made available by the investigator for this purpose.

The Source Documentation Agreement Form describes the source data for the different data in the eCRF. This document should be completed and signed by the site monitor and investigator and should be filed in the monitor's and investigator's study file. Any data item for which the eCRF will serve as the source must be identified, agreed and documented in the source documentation agreement form.

For eCRF, the monitor freezes completed and approved screens at each visit.

Upon completion or premature discontinuation of the study, the monitor will conduct site closure activities with the investigator or site staff, as appropriate, in accordance with applicable regulations, GCP, and GSK procedures.

12.3. Record retention

Following closure of the study, the investigator must maintain all site study records (except for those required by local regulations to be maintained elsewhere) in a safe and secure location. The records must be easily accessible, when needed (e.g., audit or inspection), and must be available for review in conjunction with assessment of the facility, supporting systems, and staff. Where permitted by applicable laws/regulations or institutional policy, some or all of these records can be maintained in a validated format other than hard copy (e.g., microfiche, scanned, electronic); however, caution needs to be exercised before such action is taken. The investigator must ensure that all reproductions are legible and are a true and accurate copy of the original and meet accessibility and retrieval standards, including re-generating a hard copy, if required. Furthermore, the investigator must ensure that an acceptable back-up of the reproductions exists and that there is an acceptable quality control procedure in place for making these reproductions.

GSK will inform the investigator/institution of the time period for retaining these records to comply with all applicable regulatory requirements. However, the investigator/institution should seek the written approval of the sponsor before proceeding with the disposal of these records. The minimum retention time will meet the strictest standard applicable to a particular site, as dictated by ICH GCP, any institutional requirements, applicable laws or regulations, or GSK standards/procedures.

The investigator/institution must notify GSK of any changes in the archival arrangements, including, but not limited to archival at an off-site facility, transfer of ownership of the records in the event the investigator leaves the site.

12.4. Quality assurance

To ensure compliance with GCP and all applicable regulatory requirements, GSK may conduct a quality assurance audit. Regulatory agencies may also conduct a regulatory inspection of this study. Such audits/inspections can occur at any time during or after completion of the study. If an audit or inspection occurs, the investigator and institution agree to allow the auditor/inspector direct access to all relevant documents and to allocate his/her time and the time of his/her staff to the auditor/inspector to discuss findings and any relevant issues.

12.5. Posting of information on publicly available clinical trial registers and publication policy

Study information from this protocol will be posted on publicly available clinical trial registers before enrolment of subjects begins.

Summaries of the results of GSK interventional studies (phase I-IV) are posted on publicly available results registers within 12 months of the primary completion date for studies of authorised vaccines and 18 months for studies of non-authorised vaccines.

GSK also aims to publish the results of these studies in the searchable, peer reviewed scientific literature. Manuscripts are submitted for publication within 24 months of the last subject's last visit. At the time of publication, this protocol will be fully disclosed.

Study information from this protocol will be posted on publicly available clinical trial registers following finalization of the protocol and, whenever possible, before the initiation of the analysis/study.

Results are publicly registered within 8 months of the completion of the analysis. GSK also aims to publish the results of these studies in the searchable, peer reviewed scientific literature. Manuscripts are submitted within 18 months of the completion of the analysis. At the time of publication, this protocol will be fully disclosed.

12.6. Provision of study results to investigators

Where required by applicable regulatory requirements, an investigator signatory will be identified for the approval of the study report. The investigator will be provided reasonable access to statistical tables, figures, and relevant reports and will have the opportunity to review the complete study results at a GSK site or other mutually-agreeable location.

GSK Biologicals will also provide the investigator with the full summary of the study results. The investigator is encouraged to share the summary results with the study subjects, as appropriate.

13. COUNTRY SPECIFIC REQUIREMENTS

All countries will comply with AE and SAE reporting as described in Section 9.4 of the protocol. Additionally, countries and sites will follow all applicable local regulations and guidelines for AE and SAE reporting as required by their respective healthcare authorities and ethics committees.

13.1. Requirements for France

This section includes all the requirements of the French law (n° 2004-806 of 09 August 2004), and identifies, item per item, the mandatory modifications or additional information to the study protocol.

Concerning the « STUDY POPULATION »

- In line with the local regulatory requirements, the following text about «PAYMENT TO SUBJECTS » is added:

  Subjects will be paid for the inconvenience of participating in the study. The amount of payment is stated in the ICF. Subjects not completing the study for whatever reason could be paid at the discretion of the Investigator, generally on a pro rata basis.

- In line with the local regulatory requirements, the following text about **« NATIONAL FILE »** is added:

All subjects who will be paid, will be recorded into the "national File" by the investigator. They could be identified and monitored under the « Fichier national ».

The following details will be described:

- Reference of the study,
- Surname and first name,
- Date and place of birth,
- Sex,
- Dates of beginning and termination of the study,
- Exclusion period,
- The total amount of allowance.
- In line with the local regulatory requirements, the following text in section «OTHER STUDY ELIGIBILITY CRITERIA CONSIDERATIONS » is added:

  A subject will be eligible for inclusion in this study if he /she is either affiliated to or beneficiary of a social security category.

  It is the investigator's responsibility to ensure and to document (in source document - patient notes) that the patient is either affiliated to or beneficiary of a social security category.

Concerning the "DATA ANALYSIS AND STATISTICAL CONSIDERATIONS" and specially in the "SAMPLE SIZE ASSUMPTION"

- The expected number of patients to be recruited in France is declared to the French regulatory authority.

Concerning the "STUDY CONDUCT CONSIDERATIONS"

- In section "Regulatory and Ethical Considerations, Including the Informed Consent Process"

Concerning the process for informing the patient or his/her legally authorised representative, the following text is added:

- French Patient ICF is a document which summarises the main features of the study and allows collection of the patient's written consent in triplicate. It also contains a reference to the authorisation of ANSM and the approval from the French Ethic committee and the maintenance of confidentiality of the returned consent form by GSK France.

Concerning the process for obtaining subject informed consent:

- When **biomedical research is carried out on an adult in the care of a "tutelle" guardian**, consent is given by their legal representative and, if the committee mentioned in article L. 1123-1 considers that the research in question, because of the gravity of the restraints or the specificity of the medical acts involved, entails a serious risk of affecting their private life or the integrity of their body, by the family council if it has been instated, or by the judge of "tutelle" guardians.
- When biomedical research is carried out on an adult in the care of a "curatelle" guardian, consent is given by the subject assisted by his guardian.

  However, if the adult in the care of a "curatelle" guardian is invited to participate in research which the committee mentioned in article L. 1123-1 considers, because of the gravity of the restraints or the specificity of the medical acts involved, to entail a serious risk of affecting their private life or the integrity of their body, the matter is submitted to the judge of guardians who decides whether the adult is capable of giving his consent. In the case of incapacity, the judge will decide whether or not to authorise the biomedical research.
- When biomedical research, which complies with the conditions laid down in article L. 1121-8, is considered for **an adult incapable** of expressing his consent and not under a legal protection order, consent is given by a person of confidence as defined in article L. 1111-6 and, failing this, by a person who maintains close and stable links with the subject. However, if the committee mentioned in article L. 1123-1 considers that the research in question, because of the gravity of the restraints or the specificity of the medical acts involved, entails a serious risk of affecting their private life or the integrity of their body, consent is given by the judge of guardians.

Concerning the management of the Patient ICFs, the following text is added:

- The first copy of the Patient ICF is kept by the investigator. The second copy is kept by the Director of the Medical Direction of GSK France and the last copy is given to the patient or his/her legally authorised representative.
- The second copy of all the consent forms will be collected by the investigator at the end of the trial under the Clinical Research Assistant's (CRA's) control, and placed in a sealed envelope bearing only:
  - the study number,
  - the identification of the Centre : name of the principal investigator and number of centre),
  - the number of informed consents,
  - the date,
  - and the principal investigator's signing.

  Then, the CRA hands the sealed envelope over to the Medical Direction, for confidential recording, under the responsibility of the Medical Director.

In section concerning the " NOTIFICATION TO THE HOSPITAL DIRECTOR " the following text is added (if applicable)

- In accordance with Article L1123-13 of the Public Health Code, the Hospital Director is informed of the commitment to the trial in his establishment. The Hospital Director is supplied with the protocol and any information needed for the financial disposition, the name of the investigator(s), the number of sites involved in his establishment and the estimated time schedule of the trial (R.1123-63).

In section concerning the " INFORMATION TO THE HOSPITAL PHARMACIST " the following text is added (if applicable)

- In accordance with Article R.1123-64 of the Public Health Code, the Hospital Pharmacist is informed of the commitment to the trial in his establishment. The Pharmacist is supplied with a copy of the protocol (which allows him to dispense the drug(s) of the trial according to the trial methodology), all information concerning the product(s) of the trial (e.g., included in the CIB), the name of the investigator(s), the number of sites involved in his establishment and the estimated time schedule of the trial.

In section " DATA MANAGEMENT " the following text is added

- " Within the framework of this clinical trial, data regarding the identity of the investigators and/or co-investigators and/or the pharmacist if applicable, involved in this clinical trial, and data regarding the patients recruited in this clinical trial (patient number, treatment number, patient status with respect to the clinical trial, dates of visit, medical data) will be collected and computerised in GSK data bases by GSK Laboratory or on its behalf, for reasons of follow up, clinical trial management and using the results of said clinical trial. According to the Act n° 78-17 of 6th January 1978 further modified, each of these people aforesaid has a right of access, correction and opposition on their own data through GSK Laboratory (Clinical Operations Department)."

Concerning Monitoring visits

- The Health Institution and the Investigator agree to receive on a regular basis a CRA of GSK or of a service provider designated by GSK. The Health Institution and the Investigator agree to be available for any phone call and to systematically answer to all correspondence regarding the Study from GSK or from a service provider designated by GSK. In addition, the Health Institution and the Investigator agree that the CRA or the service provider designated by GSK have direct access to all the data concerning the Study (test results, medical record, etc …). This consultation of the information by GSK is required to validate the data registered in the eCRF, in particular by comparing them directly to the source data. In accordance with the legal and regulatory requirements, the strictest confidentiality will be respected.

Concerning Data entry into the eCRF *(Amended 11 May 2020)*

The Health Institution and the Investigator agree to meet deadlines, terms and conditions of the Study's eCRF use here below :

The Health Institution and the Investigator undertake:

1. That the Investigator and the staff of the investigator centre make themselves available to attend the training concerning the computer system dedicated to the eCRF of the Study provided by GSK or by a company designated by GSK.
2. That the Investigator and the staff of the investigator centre use the IT Equipment loaned and/or the access codes only for the purpose of which they are intended and for which they have been entrusted to them, namely for the Study achievement, to the exclusion of any other use.
3. That the Investigator and the staff of the investigator centre use the IT Equipment loaned according to the specifications and manufacturer's recommendations which will have been provided by GSK.
4. To keep the IT Equipment and/or access codes in a safe and secure place and to only authorise the use of this IT Equipment by investigator centre staff designated by the principal investigator to enter the data of the Study.

5. That the Investigator and the staff of the investigator centre enter the data of the eCRF related to a patient visit in the ***10*** days following the date of the patient visit or, for the patient test results, in the ***10*** days following the reception of the results of such tests. ***For a subject last visit, the recommended time frame for data entry is 5 working days***.
6. That the Investigator resolves and returns to GSK the data queries issued by GSK or a service provider designated by GSK within ***10*** days after the reception of the request of clarification or in a period of ***5*** day***s*** during the final stage of clarification of the data base or in such other period as provided by GSK and/or a company designated by GSK.
7. To be responsible for the installation and payment of the required Internet connections needed for the use of the IT Equipment, Computer systems and/or access codes.
8. To return at the end of the Study the IT Equipment and/or access codes to GSK or to any company designated by GSK and any training material and documentation. The IT Equipment cannot under any circumstances be kept by the Health Institution or the Investigator for any reason whatsoever.

Concerning CTR publication

- It is expressly specified that GSK and/or the Sponsor can make available to the public the results of the Study by the posting of the said results on a website of the GSK Group named Clinical Trial Register (CTR) including the registration of all the clinical trials conduct by the GSK Group and this before or after the publication of such results by any other process.

Concerning Data Protection French Law of 6 January 1978 (CNIL)

- In accordance with the Data Protection French Law of 6 January 1978 as modified, computer files used by GSK to monitor and follow the implementation and the progress of the Study are declared with the CNIL by GSK. The Investigator has regarding the processing data related to him a right of access, of rectification and of opposition with GSK in accordance with the legal provisions. This information can be transferred or be accessed to other entities of GSK Group in France, Britain or United States, what the Investigator agrees by the signature of the present Protocol.

13.2. Requirements for Germany

EXPLANATORY STATEMENT CONCERNING GENDER DISTRIBUTION (ARTICLE 7, PARAGRAPH 2 (12) OF THE GERMAN GCP ORDER)

- There is no intention to conduct specific analyses investigating the relationship between the gender of the subjects and the safety of the GSK Biologicals' HZ/su vaccine. The ratio of male to female subjects recruited into the study ZOSTER-049 is expected to be in line with the demographics of the population aged ≥ 50 YOA in the Member State.

13.3. Requirements for Japan

Regulatory and Ethical Considerations

The study will be conducted in accordance with “the Ministerial Ordinance on the Standards for the Conduct of Clinical Trials of Medicinal Products (Ministry of Health and Welfare [MHW]/ Ministry of Health, Labour and Welfare Notification No.28 dated 27th March, 1997)” GCP and, Article 14-3 and 80-2 of the Pharmaceutical Affairs Law and Evaluation and Licensing Division, Pharmaceutical Safety Bureau of MHW, Notification No. 1061, 1998.

Clinical Trial Notification to Regulatory Authority

GSK Japan will submit the CTN to the regulatory authorities in accordance with Article 80-2 of the Pharmaceutical Affairs Law before conclusion of any contract for the conduct of the study with study sites.

Informed Consent of Subjects

Informed consent will be obtained before the subject can participate in the study. The contents and process of obtaining informed consent will be in accordance with all applicable regulatory requirements.

Informed Consent

Prior to the start of the study, the investigator (or subinvestigator) should fully inform the potential subject of the study including the written information given approval by the IRB. The investigator (or subinvestigator) should provide the subject ample time and opportunity to inquire about details of the study and to decide whether or not to participate in the study. After giving informed consent based on his/her free will, the subject should sign and personally date the consent form. If the subject wishes to consider the content of the written information at home, he/she may sign the consent form at home. The person who conducted the informed consent discussion should sign and personally date the consent form. If the subject is unable to read, an impartial witness should be present during the entire informed consent discussion, and the witness should sign and personally date the consent form. The investigator (or subinvestigator) should retain this signed and dated form (and other written information) together with the source medical records, such as clinical charts (in accordance with the rules for records retention, if any, at each medical institution) and give a copy to the subject.

If information becomes available that may be relevant to the subject's willingness to continue participation in the study (revision of informed consent form and other written information)

If information becomes available that may be relevant to the subject's willingness to continue participation in the study, the investigator (or subinvestigator) should immediately inform the subject of it to confirm the willingness to continue participation in the study, and document the communication of this information (in medical records). If necessary, the investigator should revise the written information to be provided to

subjects, promptly report it to the sponsor, and obtain approval from the IRB. The investigator should not enrol any new subject in the study before the IRB's approval. After the IRB approves the revision of the written information to be provided to subjects, the investigator (or subinvestigator) should inform each subject participating in the study of the revised written information, and obtain written informed consent.

Study Monitoring

By monitoring the parties involved in the study including medical institutions, investigators, subinvestigators, study collaborators, and storage managers, monitors will:

1. Oversee the process of obtaining written informed consent, the control of investigational products and the progress of the study (including withdrawals and adverse events, and ensure that the conduct of the study is in compliance with GCP, Revised GCP, this protocol, and any other written agreement between the sponsor and the investigator/institution.
2. Collect and provide information that is necessary to conduct the study properly (information on investigational products' safety, efficacy and quality).
3. Verify that the investigator/institution has adequate qualifications and resources and remain adequate throughout the study period, and that facilities, including laboratories, equipment, and staff, are adequate to safely and properly conduct the study and remain adequate throughout the study period.
4. Verify that source documents and other study records are accurate, complete, kept up-to-date and maintained.
5. Determine whether the person responsible for retaining records is maintaining the essential documents at each medical institution.
6. Check the accuracy and completeness of the CRF entries, source documents and other study-related records against each other.

The investigator and institution should agree to allow the monitor direct access to essential documents and other relevant documents.

Direct access to essential documents by monitors and the scope of those documents will be specified separately in the written procedures for monitoring prepared for this study.

Source Data Recorded Directly on CRF

The following data may be recorded directly on the CRFs and considered to be source data.

1. Assessment of causality between adverse events and the investigational product.

Deviations from and Changes of Protocol

Deviations from Protocol

The investigator (or sub-investigator) may implement a deviation from, or a change of, the protocol to eliminate an immediate hazard(s) to subjects without agreement by the sponsor or prior IRB approval. As soon as possible, the implemented deviation or change and the reasons for it should be submitted to the head of the medical institution and the IRB for approval, and via the head of the medical institution to the sponsor for agreement.

The investigator (or subinvestigator) should document all deviations from the approved protocol. The investigator should document the reason only for the deviation from, or the change of, the protocol to eliminate an immediate hazard(s) to subjects, and submit it to the sponsor and the head of the medical institution, and retain its copy.

Changes of Protocol

1. If it becomes necessary to make any changes significantly affecting the conduct of the study, and/or increasing the risk to subjects, the sponsor should promptly document the changes and reasons for them and amend the protocol after discussion with the (coordinating [investigator, committee members] and) investigators, and notify the heads of the medical institutions and investigators of the changes of the protocol (sample ICF and other written information, if necessary). The investigator should not implement any significant changes without approval from the IRB.
2. For changes other than the above 1), the sponsor should document the changes and reasons for them and inform the heads of the medical institutions and investigators of the changes of the protocol. Such changes require prior approval from the IRB, except where necessary to eliminate an immediate hazard(s), or when the change(s) involves only logistical or administrative aspects of the study. The investigator should promptly report the changes implemented without prior approval to the IRB for approval.

Study Period

The exact study period has not been confirmed at the time of this protocol. When needed and available, the study period can be communicated at local level, outside of this protocol.

Shingrix was approved in Japan on 23 March 2018, after study start. Therefore, this study is being conducted as a post-marketing study from the approval date in Japan.

14. REFERENCES

Araújo LQ, MacIntyre CR, Vujacich C. Epidemiology and burden of herpes zoster and post-herpetic neuralgia in Australia, Asia and South America. *Herpes* 2007; 14(Suppl 2): 40A–44A.

Carter WP, Germann CA, Baumann MR. Ophthalmic diagnoses in the ED: herpes zoster ophthamicus. *Am. J. Ophthalmol.* 2008; 612-7.

CDC. Prevention of herpes zoster. Recommendations of the Advisory Committee on Immunization Practices (ACIP). *MMWR*. 2008; 57: 1-30.

Centers for Disease Control and Prevention Metropolitan Atlanta Congenital Defects Program (CDC MACDP) guidelines. Birth defects and genetic diseases branch 6-digit code for reportable congenital anomalies; 2007. http://www.cdc.gov/ncbddd/birthdefects/documents/MACDPcode0807.pdf. Website accessed 19 October 2015.

Cohen JI, Straus SE, Arvin AM. Varicella zoster virus and its replication. 2007; p. 2774-2818. In D. M. Knipe and P. M. Howley (ed.), *Fields virology*, 5th ed. Lippincott/The Williams & Wilkins Co., Philadelphia, PA.

Cunningham AL, Lal H, Kovac M, et al., Efficacy of the Herpes Zoster Subunit Vaccine in Adults 70 Years of Age or Older. N Engl J Med. 2016; 375(11): 1019-32.

Dworkin RH, Johnson RW, Breuer J, et al. Recommendations for the management of herpes zoster. *Clin Infect Dis.* 2007;44 Suppl 1:S1–26.

EMA Guideline on the exposure to medicinal products during pregnancy: need for post-authorization data (Doc. Ref. EMEA/CHMP/313666/2005) 'adopted at Community level in May 2006); *http://www.ema.europa.eu/docs/en_GB/document_library/Regulatory_and_procedural_guideline/2009/11/WC500011303.pdf*

EMEA. Zostavax Summary of Product Characteristics. 2011. http://www.ema.europa.eu/docs/en_GB/document_library/EPAR_-_Summary_for_the_public/human/000674/WC500053457.pdf. Website accessed 19 October 2015.

FDA, U.S. Department of Health and Human Services, Center for Biologics Evaluation and Research. Guidance for Industry: Toxicity Grading Scale for Healthy Adult and Adolescent Volunteers Enrolled in Preventive Vaccine Clinical Trials. Version of September 2007.

Garcia Cenoz M, Castilla J, Montes Y, et al. Varicella and herpes zoster incidence prior to the introduction of systematic child vaccination in Navarre, 2005-2006. *An Sist Sanit Navar*. 2008; 31: 71-80.

Gilden D, Cohrs RJ, Mahalingam R, et al. Varicella zoster virus vasculopathies: diverse clinical manifestations, laboratory features, pathogenesis, and treatment. *Lancet Neurology*. 2009; 8(8): 731-40.

Kang CI, Choi CM, Park TS, et al. Incidence of herpes zoster and seroprevalence of varicella-zoster virus in young adults of South Korea. *Int J Inf Dis.* 2008; 12(3): 245-7.

Lal H, Cunningham AL, Godeaux O et al. Efficacy of an Adjuvanted Herpes Zoster Subunit Vaccine in Older Adults. *N Engl J Med.* 2015: DOI: 10.1056/NEJMoa1501184.

Morrison VA, Johnson GR, Schmader KE, et al. Long-Term Persistence of Zoster Vaccine Efficacy. *Clin Infect Dis*. 2015; 60(6): 900-9.

Oxman MN, Levin MJ, Johnson GR, et al. Shingles Prevention Study Group. A vaccine to prevent herpes zoster and postherpetic neuralgia in older adults. *N Engl J Med*. 2005; 352: 2271-84.

Schmader KE, Dworkin RH. Natural history and treatment of herpes zoster. *The Journal of pain*. 2008; 9(1):S3–S9.

Schmader KE, Oxman MN, Levin MJ, et al. Persistence of the Efficacy of Zoster Vaccine in the Shingles Prevention Study and the Short-Term Persistence Substudy. *Clin Infect Dis*. 2012; 55: 1320-28.

Scott F, Johnson R, Leedham-Green M, et al. The burden of herpes zoster: A prospective population based study. *Vaccine.* 2006; 24: 1308-1314.

Sperber SJ, Smith BV, Hayden FG. Serologic response and reactogenicity to booster immunization of healthy seropositive adults with live or inactivated varicella vaccine. *Antiviral Res*. 1992; 17: 213-22.

TGA eBS Australian Register of Therapeutic Goods Medicines. Public summary ZOSTAVAX FROZEN zoster virus vaccine live min 19400 PFU/0.65mL powder for injection vial. 2015. https://www.ebs.tga.gov.au/servlet/xmlmillr6?dbid=ebs%2FPublicHTML%2FpdfStore.nsf&docid=DCED7AF72A4FC616CA257EDE003CB146&agid=(PrintDetailsPublic)&actionid=1. Website accessed 19 October 2015.

Toyama N, Shiraki K, and Members of the Society of the Miyazaki Prefecture Dermatologists. Epidemiology of herpes zoster and its relationship to varicella in Japan: A 10-year survey of 48,388 herpes zoster cases in Miyazaki prefecture. *J Med Virol.* 2009; 81(12): 2053-58.

Zostavax (Zoster Vaccine Live) Prescribing information. June 2011.

APPENDIX A LABORATORY ASSAYS

Specific Ab (anti-gE) measurements:

Anti-gE ELISA: Anti-gE Ab concentrations will be measured using an anti-gE ELISA. Diluted blood serum samples of study subjects will be added to microtitre wells pre-coated with gE antigen. Secondary peroxidase-conjugated anti-human Abs will be added, which bind to the primary human anti-gE Abs. After incubation of the microtitre wells with a chromogen substrate solution, the enzymatic reaction will be stopped. Optical densities will be recorded and anti-gE Ab concentrations are calculated from a standard curve. The assay cut-off is 97 mIU/mL.

Intracellular cytokine staining (ICS):

CMI responses will be performed by GSK Biologicals (or designated laboratory) on thawed Peripheral Blood Mononuclear Cells (PBMCs) by ICS. The assay will be performed on samples collected during the course of the study. This assay provides information on the frequency of CD4 T cells responding to culture medium or antigens (gE peptide pool) by expressing molecules involved in immunity such as IFN-γ, IL-2, TNF-α, and CD40L (here considered as activation markers).

Briefly, PBMC collected from the subjects are stimulated for two hours using culture medium (for evaluation of the non-specific response), a pool of overlapping peptides covering the entire sequence of the vaccine antigen gE. Then, an intracellular block (brefeldin A) is added to inhibit cytokine secretion for a subsequent overnight incubation. Cells are then harvested, stained for surface markers (CD3, CD4 and CD8) and fixed. The fixed cells are then permeabilised and stained with Abs specific to the activation markers assessed, washed and analyzed by cytofluorometry.

The results of ICS assays are expressed as the frequency of specific CD4 T cells per million total CD4 T cells.

CCI

[redacted]

PCR assay for confirmation of suspected case of HZ:

HZ cases will be confirmed by a Polymerase Chain Reaction (PCR) based algorithm that assesses the presence of VZV Deoxyribonucleic Acid (DNA) in samples, and the adequacy of the samples (by assessing the presence of β-actin DNA).

VZV and β-actin DNA in HZ clinical specimens will be assessed using real-time PCR detection by the 5' nuclease assay based on the Taqman probe technology. If the VZV PCR is negative, β-actin PCR will be performed to assess adequacy of the sample and if a specimen is found to be VZV–PCR negative and β-actin–PCR negative, it is considered to be inadequate.

In the Taqman-based PCR experiments, the formation of a PCR product is monitored in real-time during amplification by means of fluorogenic probes that bind specifically to the amplified product. The reporter fluorophore is at the 5' end of the Taqman probe and the quencher is at the 3' end. As long as the probe is intact, no fluorescence is produced by the fluorophore. During the PCR polymerization step, the Taq DNA polymerase displaces the Taqman probe by 3-4 nucleotides, and the 5' nuclease activity of the DNA polymerase separates the fluorophore from the quencher, and a measurable fluorescent signal proportional to the DNA copy number is produced.

As mentioned above, the 5' nuclease-based PCR assay allows the determination of the DNA copy number within samples, but in the present study the VZV and β-actin DNA PCR data on samples from suspected HZ lesions (swabs of vesicles, papules and crusts, and crusts themselves) will be used qualitatively only according to the above mentioned approach.

- **Ascertainment of HZ cases including the PCR testing algorithm to classify HZ suspected cases**

A suspected case of HZ will be documented by digital photography of the rash (if rash is present) and by collecting any relevant information as described in the clinical protocol.

To classify the suspected case of HZ, the samples from the rash lesions (if available) will be collected for laboratory testing by PCR (3 samples, collected on the same day, per subject). If during clinical evaluation at Visit HZ-1, the investigator determines that adequate rash lesion samples cannot be collected (i.e., less than three lesions present, or if only papules are present), the investigator has the option of collecting three additional samples preferably within 7 days, or at the Visit HZ-2 if there is rash progression (i.e., appearance of new/additional lesions if originally less than three lesions present, or appearance of vesicles if originally only papules present). When the subject returns for repeat sample collection, if possible, three samples from separate lesions should be collected. Refer to the SPM for further details on sample collection.

Each rash lesion will be tested using standardised and validated molecular assays according to the PCR testing algorithm described below.

A hierarchical case definition algorithm, similar to the algorithm used by Merck in their Shingle Prevention Study (*Zostavax* efficacy study) [Oxman, 2005] will be used to classify each suspected case of HZ as a confirmed HZ case or not.

- If at least 1 sample coming from a given subject is “VZV positive” by PCR (as defined below), the PCR algorithm will classify the “suspected HZ case” as a “confirmed case of HZ”.
- If all the samples coming from a given subject are “VZV negative” (as defined below), then β-actin PCR will be performed. If one or more “VZV negative” samples are “β-actin positive”, this means that the sampling procedure is valid and that the “suspected HZ case” will be classified as “not a case of HZ”.
- If PCR results for a particular subject do not confirm or exclude a “suspected HZ case” (i.e., samples coming from a given subject are considered as “inadequate” as both VZV and β-actin PCR results are negative, or no samples are available for the subject), only then will the classification by the HZAC be used to confirm or exclude the suspected HZ case. The HZAC will consist of physicians with HZ expertise. For every suspected HZ case, each HZAC member will be asked to make a clinical determination of whether the case is HZ based on review of the available clinical information. A “suspected HZ case” will be considered as “HZ” if all HZAC members concur (unanimous decision); otherwise, it will be classified as “not HZ”.

This algorithm includes the following steps (see Figure 3):

1. DNA extraction from the rash lesion.
2. VZV real-time PCR assay (PCR) targeting the orf62 gene is performed to detect VZV in the rash lesion:
    a. If the VZV PCR signal is ≥ the cut-off level, i.e., the technical limit of detection (LOD) of the assay (10 VZV DNA copies), the sample will be considered as “VZV positive”.
    b. If the VZV PCR signal is above 0 copy/PCR but below the cut-off level of the assay, it will be considered as “VZV borderline” and will be re-tested twice in order to obtain 3 results per sample. The sample will be considered as “VZV positive” if at least 2 results out of the three obtained are ≥ the cut-off level of the assay and it will be considered “VZV negative” if fewer than 2 samples are ≥ the cut-off level of the assay.
    c. If the VZV PCR signal is equal to 0 copies/PCR, the sample will be considered as “VZV negative”. If every sample is VZV negative, then extracted DNA from the samples will be assessed for the presence of β-actin DNA to confirm the validity of the rash lesion sampling procedure (see step 3).
3. As described above, if all the samples are VZV negative for a given subject, then β-actin PCR will be performed on “VZV negative” samples to confirm the validity of the sampling procedure.
    a. If the β-actin PCR signal is below the cut-off level of the assay (β-actin Negative), the sample will be considered as “inadequate” as no β-actin DNA from human cells is detected within the rash lesion sample. If all samples are β-actin Negative, then the classification by the HZAC will be used to confirm or exclude the HZ case.

b. If the β-actin PCR signal is ≥ the cut-off level of the assay (β-actin Positive), the sample will be considered as "valid" but without any VZV DNA. If at least one sample is β-actin Positive, then the HZAC classification of a suspected HZ case, will not be part of the decision-making process for HZ case confirmation.

Note: The cut-off level of the VZV PCR and β-actin PCR assays is defined as the technical limit of detection of these assays (LOD; i.e., lowest concentration that can be detected by PCR in at least 95% of the tests).

Figure 3 Algorithm for HZ case definition by PCR

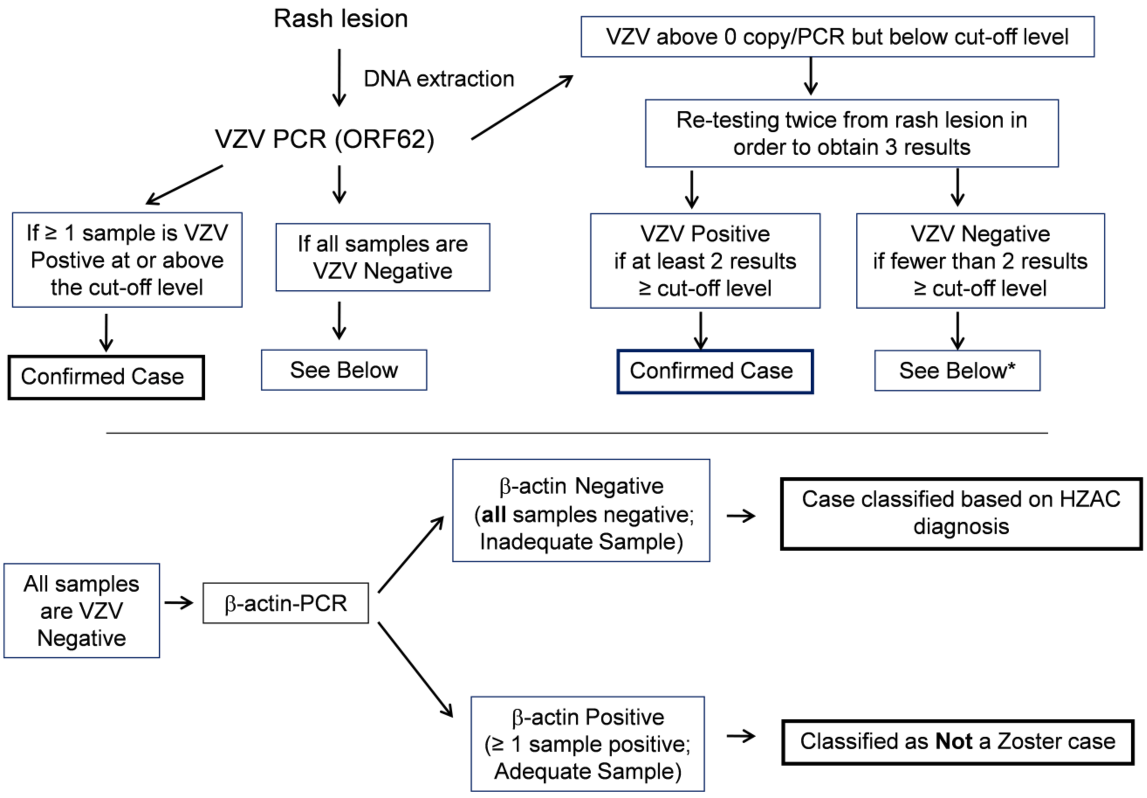

VZV: Varicella Zoster Virus; PCR: real-time Polymerase Chain Reaction; DNA: Deoxyribonucleic Acid; ORF: Open Reading Frame; HZAC: Herpes Zoster Ascertainment Committee.

* If the VZV PCR signal is above 0 copy/PCR but below the cut-off level of the assay, it will be considered as "VZV borderline" and will be re-tested twice in order to obtain 3 results per sample. The sample will be considered as "VZV positive" if at least 2 results out of the three obtained are ≥ the cut-off level of the assay and it will be considered "VZV negative" if fewer than 2 samples are ≥ the cut-off level of the assay. See then below 'All samples are VZV Negative'.

Note: The cut-off level of the VZV PCR assay was defined as the technical limit of detection of the assay (LOD of 10 VZV DNA copies; i.e., lowest concentration that can be detected by PCR in at least 95% of the tests)

APPENDIX B CLINICAL LABORATORIES

Table 31 GSK Biologicals' laboratories

Laboratory	Address
GSK Biologicals Global Vaccine Clinical Laboratory, Rixensart	Biospecimen Reception - B7/44<br>Rue de l'Institut, 89 - B-1330 Rixensart - Belgium
GSK Biologicals Global Vaccine Clinical Laboratory, Wavre-Nord Noir Epine	Avenue Fleming, 20 - B-1300 Wavre - Belgium

GSK = GlaxoSmithKline

Table 32 Outsourced laboratories

Laboratory	Address
CEVAC - University of Ghent	De Pintelaan, 185 - B-9000 Ghent - Belgium

CEVAC = Centre for Vaccinology

APPENDIX C AMENDMENTS AND ADMINISTRATIVE CHANGES TO THE PROTOCOL

GlaxoSmithKline Biologicals	
**Vaccine Value & Health Science (VVHS)**<br>**Protocol Amendment 1**	
**eTrack study number and Abbreviated Title**	201190 (ZOSTER-049 EXT:006-022)
**IND number**	BB-IND 13857
**EudraCT number**	2015-001778-17
**Amendment number:**	Amendment 1
**Amendment date:**	19 February 2016
**Co-ordinating author:**	PPD, Scientific writer, XPE Pharma & Science, Contractor for GSK Biologicals
**Rationale/background for changes:**<br>• This amendment was done for countries that will recruit subjects in the 1-Additional Dose, Revaccination and Control groups. Administrative changes were done for Germany and Japan.<br>• The additional inclusion and exclusion criteria for the 1-Additional Dose and Revaccination groups will also be made applicable to the Control group to allow non-biased randomisation of subjects into the 3 groups (Sections 5.2, 5.3, 6.1.2, 6.5.5, 6.5.6 and Table 6) and an additional exclusion criterion has been added for 1-Additional Dose, Revaccination and Control groups (Section 5.3).<br>• The reference to "GSK Japan" has been changed to "Japan Vaccine Co., Ltd." (Investigator Agreement page for Japan and in the requirements for Japan Section 13.3).<br>• The study period indicated was also corrected as per local request in the requirements for Japan (Section 13.3).<br>• Typographical errors were corrected in addition to the term "ZOSTER-056" that was corrected to "ZOSTER-049" in the requirements for Germany (Section 13.2).	

Amended text has been included in *bold italics* and deleted text in ~~strikethrough~~ in the following sections:

Investigator Agreement page

~~GSK Japan study representative / Joint Vaccine Co~~ *Japan Vaccine Co., Ltd.* representative name, function and title

Synopsis and Section 1.2.2 Rationale for the study design

1-Ad*d*itional Dose Group:

Section 5.2 Inclusion criteria for enrolment

Additional inclusion criteria for the 1-Additional Dose, ~~and~~ Revaccination *and Control* groups, ONLY:

Section 5.3 Exclusion criteria for enrolment

Additional exclusion criteria for the 1-Additional Dose, ~~and~~ Revaccination *and Control* groups, ONLY:

- ***Use of any investigational or non-registered product (pharmaceutical product or device) within 30 days preceding the first dose of study vaccine or planned use during the study period;***

~~**Additional exclusion criterion for the 1-Additional Dose, Revaccination and Control groups, ONLY:**~~

Section 6.1.2 Allocation of subjects to assay subsets

- ~~and~~ did NOT develop HZ (confirmed or suspected HZ) prior to enrolment in this ZOSTER-049 study.
- ***and comply with inclusion and exclusion criteria (see Sections 5.2 and 5.3, respectively) to be part of the randomised groups).***

Table 6 List of study procedures (for the 1-Additional Dose, Revaccination and Control groups)

Epoch	LONG-TERM FOLLOW-UP						
Type of contact	Visit MONTH 0	Visit Month 1	Visit Month 2†	Visit Month 3‡	Monthly contacts [a]	Visit YEAR 1, 2, 3, 4, 5 [b]	Study conclusion Visit [c] Year 6 [b]
Timepoints	Day 0/ Month 0	Month 1	Month 2 †	Month 3 ‡		Month 12, 24, 36, 48, 60	Month 72
Sampling timepoints	Pre-Vacc	Post-Vacc 1		Post-Vacc 2		Post-Vacc 2	Post-Vacc 2
Pregnancy test if applicable [d, e] (see Section 6.5.5)	●		●				
Check contraindications [e] (see Section 6.5.6)	O		O				
Training on self-reporting by subjects [f-e]	O	O	O	O		O	
Pre-vaccination body temperature [e f]	●		●				
Vaccination [e f]	●		●				
Reporting of pregnancy [e f] (see Table 23)	●	●	●	●	●	●	●
Dispensing of 7-day diary cards for solicited AEs to the 7-day diary card and 30-day diary cards for unsolicited AEs and concomitant medication/vaccination to all vaccinated subjects [e f]	O		O				
Daily post-vaccination recording by subjects of solicited symptoms (Days 0 - 6 after each vaccination) on the 7-day diary card by all vaccinated subjects [e f] (see Table 23)	O		O				
Daily post-vaccination recording of unsolicited symptoms (Days 0 - 29 after each vaccination), and concomitant medication/ vaccination (Days 0 - 29 after each vaccination) on the 30-day diary card by all vaccinated subjects [e f] (see Table 23)	O		O				
Returning by subjects of 7-day diary cards for solicited symptoms and 30-day diary cards for unsolicited AEs and concomitant medication and vaccination [e f]		O		O			

Epoch	LONG-TERM FOLLOW-UP						
Type of contact	Visit MONTH 0	Visit Month 1	Visit Month 2†	Visit Month 3‡	Monthly contacts [a]	Visit YEAR 1, 2, 3, 4, 5 [b]	Study conclusion Visit [c] Year 6 [b]
Timepoints	Day 0/ Month 0	Month 1	Month 2 †	Month 3 ‡		Month 12, 24, 36, 48, 60	Month 72
Sampling timepoints	Pre-Vacc	Post-Vacc 1		Post-Vacc 2		Post-Vacc 2	Post-Vacc 2
Transcription of 7-day diary cards for solicited symptoms and 30-day diary cards for unsolicited AEs and concomitant medication and vaccination by study staff/investigator [e] [f]		●		●			

[e] ~~A study procedure only for the two groups vaccinated with 1 or 2 additional doses of HZ/su vaccine (1-Additional Dose and Revaccination).~~ ***Subjects/ subject's caregiver will be instructed to contact their study site immediately if he/she develops any symptoms suggestive of HZ and/or, if he/she manifests any symptoms he/she perceives as serious.***

[f] ~~Subjects/ subject's caregiver will be instructed to contact their study site immediately if he/she develops any symptoms suggestive of HZ and/or, if he/she manifests any symptoms he/she perceives as serious.~~ ***A study procedure only for the two groups vaccinated with 1 or 2 additional doses of HZ/su vaccine (1-Additional Dose and Revaccination).***

Section 6.5.5 Pregnancy test

Female subjects of childbearing potential are to have a urine or serum pregnancy test ***before randomisation (in the 1-Additional Dose, Revaccination and Control groups) and*** prior to ~~any~~ study vaccine ***second dose*** administration (in ~~groups 1-Additional Dose and~~ ***the*** Revaccination ***group***, only).The study vaccines may only be administered if the pregnancy test is negative. Note: The urine pregnancy test must be performed even if the subject is menstruating at the time of the study visit.

Section 6.5.6 Check contraindications, warnings and precautions to vaccination

Contraindications to vaccination must be checked ~~at the beginning of each vaccination visit~~***before randomisation*** (in ~~groups~~ ***the*** 1-Additional Dose, ~~and~~ Revaccination ***and Control groups***, only) ***and prior to study vaccine second dose administration (in the Revaccination group, only)***. Refer to Sections~~s~~ 7.5 for more details.

Section 13.2 Requirements for Germany

- There is no intention to conduct specific analyses investigating the relationship between the gender of the subjects and the safety of the GSK Biologicals' HZ/su vaccine. The ratio of male to female subjects recruited into the study ZOSTER-0~~56~~***49*** is expected to be in line with the demographics of the population aged ≥ 50 YOA in the Member State.

Section 13.3 Requirements for Japan

Clinical Trial Notification to Regulatory Authority

~~GSK~~***Japan Vaccine Co., Ltd.*** will submit the CTN to the regulatory authorities in accordance with Article 80-2 of the Pharmaceutical Affairs Law before conclusion of any contract for the conduct of the study with study sites.

Study Period

~~January, 2016 – February, 2018 (current estimation)~~***The exact study period has not been confirmed at the time of this protocol. When needed and available, the study period can be communicated at local level, outside of this protocol***.

GlaxoSmithKline Biologicals

Vaccine Value & Health Science (VVHS)
Protocol Amendment 2

**eTrack study number and Abbreviated Title**	201190 (ZOSTER-049 EXT:006-022)
**IND number**	BB-IND 13857
**EudraCT number**	2015-001778-17
**Amendment number:**	Amendment 2
**Amendment date:**	19 January 2017
**Co-ordinating author:**	PPD, Scientific Writer

Rationale/background for changes:

- This protocol is being amended after study start, because this being a long-term follow-up study and considering the age of the study population and the study duration (6 years), the current safety data collection requirement of the protocol will lead to collection of safety data that is not relevant for assessing the safety profile of the vaccine. This amendment was done to qualify that for the recording of AEs/SAEs leading to withdrawal which are not related to:
  - investigational vaccine,
  - study participation,
  - GSK concomitant medication/vaccine,
  - HZ complications.

  Only the name (diagnosis/description) of the event will be recorded in the eCRF. No other details about these types of AEs/SAEs will be recorded during the entire study (for the LTFU group), after 12 months from Visit Month 0 (for the Control and 1-Additional Dose groups) and after 12 months from last HZ/su vaccination (for the Revaccination group) as indicated in Sections 9.3.1 and Tables 22 and 23.
- The footnote was added to Synopsis Table 1 and Tables 1 and 16 for clarity that all subjects entering the study will have a humoral immunogenicity (HI) blood sample at Visit Month 0 and for subjects in the LTFU group, who do not belong to any subset, these samples will be stored and tested for HI only if the subject develops HZ during the ZOSTER-049 study or if there are other reasons requiring the HI testing of these samples.
- Although no confirmed signals related to hypersensitivity reactions (including anaphylaxis) have been identified during the HZ/su clinical program, a mitigation strategy for the potential risk has been added to the Risk Assessment Section 1.3.1.
- The CMI Vaccine Response Rate (VRR) for gE was defined to align with the Statistical Analysis Plan (SAP) in Section 11.5.3.

- In addition to the Vaccine Efficacy (VE), it is now specified that assessments of immunogenicity will be performed when the last subjects have completed their year 2 and year 4 visit/contact, in the sequence of analyses Section 11.8.1.
- The statement was added "If during the conduct of the study the HZ/su vaccine (GSK 1437173A) is approved in Japan, the study will then be locally amended to be conducted as a post-marketing study." in Section 13.3.
- The PCR algorithm text and Figure 3 were updated in APPENDIX A.
- Typographic errors were corrected and other minor modifications were made throughout for clarification.

Amended text has been included in *bold italics* and deleted text in ~~strikethrough~~ in the following sections:

Title page

**Co-ordinating author**	PPD, ~~XPE Pharma and Science, Contractor for GSK Biologicals~~***Scientific Writer***
**Contributing authors**	• PPD***, Clinical Research and Development Lead***<br>• PPD***, Lead Study Delivery Lead***<br>• PPD***, Lead Study Delivery Lead***<br>• PPD, ***Lead*** Study Delivery Lead<br>• PPD ***and*** PPD***, Study Delivery Leads***<br>• PPD***, Study Delivery Lead, SynteractHCR Benelux, Contractor for GSK Biologicals***<br>• PPD, ~~and~~ PPD, ***and*** PPD PPD, SynteractHCR Benelux, Contractors for GSK Biologicals/<br>• PPD, ~~***Global Vaccine Clinical Laboratories***~~***Clinical Laboratory Sciences*** Clinical readout Team Lead***er***<br>• PPD, ~~***Global Vaccine Clinical Laboratories***~~***Clinical Laboratory Sciences*** Study Manager<br>• PPD***, Vaccine Supply Coordinator***<br>• PPD PPD***, Safety representative***<br>• PPD, ***Oversight*** Data Manager<br>• PPD ***and*** PPD ***(USA), Global Regulatory Affairs***<br>• PPD, ~~Zoster Portfolio level~~ Clinical ~~Research and Development~~ ***and Epidemiology Project*** Lead ***for Zoster***, GlaxoSmithKline Biologicals, Belgian RDC

Copyright 2015-201~~6~~7 the GlaxoSmithKline group of companies. All rights reserved. Unauthorised copying or use of this information is prohibited.

Sponsor signatory

Lidia Oostvogels, ~~Zoster Portfolio level~~ Clinical ~~Research and Development~~ ***and Epidemiology Project*** Lead ***for Zoster***, GlaxoSmithKline Biologicals, Belgian RDC

Synopsis

Rationale for the study and study design

Two large pivotal phase III trials ZOE-50 and ZOE-70 that enrolled subjects ≥ 50 and ≥ 70 years of age (YOA) respectively, ~~are~~ evaluat***ed***~~ing~~ the vaccine efficacy (VE), immunogenicity and safety of GSK Biologicals' HZ/su vaccine.

The results for the ZOSTER-022 study ~~are not yet available~~ ***demonstrated that the HZ/su vaccine reduced the risks of HZ and PHN among adults ≥ 70 YOA***.

Since the mean follow-up period for VE at the time of the final analysis in the ZOSTER-006/022 studies ~~will only be around~~***was about*** four years, further follow-up for long term efficacy assessment is needed~~,~~ in order to establish that the HZ/su vaccine provides not only strong, but also persistent protection.

Due to the high VE observed in the ZOSTER-006***/022*** stud~~y~~***ies***, it was considered that subjects having received placebo during ***both of*** the ~~ZOSTER-006/022~~ studies should be offered cross-vaccination with HZ/su vaccine as soon as possible.

Objectives

Secondary

- To assess the VE in the prevention of HZ over each year of follow-up from one month post dose 2 ***in the ZOSTER-006/022 studies*** as measured by the reduction in HZ risk in subjects ≥ 50 YOA and within each of the specified age ranges* at the time of first vaccination in the ZOSTER-006/022 studies;

Synopsis Table 1 Study groups and epochs foreseen in the study

Study groups	Number of subjects			Epochs
	From ZOSTER-006 primary study	From ZOSTER-022 primary study	Total	Epoch 001
LTFU			≤ 14208 **	x

***** All subjects (N ≤ 14,448) entering the study will have a HI blood sample (approximately 5 mL) at Visit Month 0. For subjects in the LTFU group, who do not belong to any subset, these samples will be stored and tested for HI only if the subject develops HZ during the ZOSTER-049 study or if there are other reasons requiring the HI testing of these samples.***

LIST OF ABBREVIATIONS

ORF:	***Open Reading Frame***
~~VR:~~	~~Vaccine Response~~

TRADEMARKS

Trademarks not owned by the GlaxoSmithKline group of companies
Varivax® (Merck & Co***., Inc.***)

Section 1.1 Background

The potential of vaccination to protect against HZ was evaluated in a large efficacy study in which *Zostavax* (a live attenuated HZ vaccine that is a high titre preparation of the varicella vaccine, *Varivax* [both manufactured by Merck & Co***., Inc.***]) partially protected immunocompetent older adults against HZ [Oxman, 2005].

Two large pivotal phase III trials ZOE-50 and ZOE-70 that enrolled subjects ≥ 50 and ≥ 70 years of age (YOA) respectively, ~~are~~ evaluat***ed***~~ing~~ the vaccine efficacy (VE), immunogenicity and safety of GSK Biologicals' HZ/su vaccine.

The results for the ZOSTER-022 study ~~are not yet available~~ ***demonstrated that the HZ/su vaccine reduced the risks of HZ and PHN among adults ≥ 70 YOA [Cunningham, 2016]***.

Section 1.2.1 Rationale for the study

Since the mean follow-up period for VE at the time of the final analysis in the ZOSTER-006/022 studies ~~will only be around~~***was about*** four years, further follow-up for long term efficacy assessment is needed~~,~~ in order to establish that the HZ/su vaccine provides not only strong, but also persistent protection.

Due to the high VE observed in the ZOSTER-006***/022*** stud~~y~~***ies***, it was considered that subjects having received placebo during ***both of*** the ~~ZOSTER-006/022~~ studies should be offered cross-vaccination with HZ/su vaccine as soon as possible.

Section 1.3.1 Risk Assessment

Important Potential/Identified Risk	Data/Rationale for Risk	Mitigation Strategy
**Investigational HZ/su vaccine**		
Hypersensitivity reactions (including anaphylaxis)	***No confirmed signals related to this potential risk have been identified during the clinical program. Available clinical data do not highlight any concern.***	***Administration of the study vaccination is to be preceded by a review of the subjects' medical history (especially with regard to previous vaccination and possible occurrence of undesirable events) and a clinical examination.<br>As with all injectable vaccines, appropriate medical treatment and supervision should always be readily available in case of an anaphylactic event following the administration of the vaccine.***

Section 2.2 Secondary objectives

- To assess the VE in the prevention of HZ over each year of follow-up from one month post dose 2 ***in the ZOSTER-006/022 studies*** as measured by the reduction in HZ risk in subjects ≥ 50 YOA and within each of the specified age ranges* at the time of first vaccination in the ZOSTER-006/022 studies;

Section 3 STUDY DESIGN OVERVIEW

Table 1 Study groups and epochs foreseen in the study

Study groups	Number of subjects			Epochs
	From ZOSTER-006 primary study	From ZOSTER-022 primary study	Total	Epoch 001
LTFU			≤ 14208 **	x

***** All subjects (N ≤ 14,448) entering the study will have a HI blood sample (approximately 5 mL) at Visit Month 0. For subjects in the LTFU group, who do not belong to any subset, these samples will be stored and tested for HI only if the subject develops HZ during the ZOSTER-049 study or if there are other reasons requiring the HI testing of these samples (see Section 6.5.8.1).***

Section 5.3 Exclusion criteria for enrolment

- Chronic administration (defined as ~~≥~~ > 14 consecutive days in total) of immunosuppressants or other immune-modifying drugs during the period starting six months prior to Visit Month 0 of study ZOSTER-049 or expected administration at any time during the study period.

Section 6.3.1.1 Monthly contacts

After Visit Month 0, monthly contacts between the subjects/ subject's caregiver and the investigator and/or his delegate will take place to collect information on any event of interest that may have occurred [see Section 6.5.7 for details].

Section 6.3.1.2 Diary cards and questionnaires

For subjects in the two groups (1-Additional Dose and Revaccination, only)**,** 7-day and 30-day diary cards will be dispensed on the day of vaccination to be completed by the subjects/ subjects' caregiver.

Section 6.5.5 Pregnancy test

Female subjects of childbearing potential are to have a urine or serum pregnancy test before randomisation (in the 1-Additional Dose, Revaccination and Control groups) and prior to study vaccine second dose administration (in the Revaccination group, only). The study vaccines may only be administered if the pregnancy test is negative. Note: The urine pregnancy test must be performed even if the subject is menstruating at the time of the study visit.

Section 6.6.4.1. Immunological read-outs

Table 16 Immunological read-outs

Blood sampling timepoint [1]			Subset Name [2]	Approximate No. of Subjects	Component
Visit No.[3]	Month	Timing			
Visit Month 0	0	Post-Primary	All Subjects	≤ 14448 *[4]*	Ab gE ELISA
			LTFU Group CMI subset	≤ 234	ICS gE
		Pre-Vacc	1-Additional Dose, Revaccination and Control Groups	240	ICS gE
Visit Month 1	1	Post-Vacc 1	1-Additional Dose, Revaccination and Control Groups	240	ICS gE
			1-Additional Dose, Revaccination and Control Groups	240	Ab gE ELISA
Visit Month 3	3	Post–Vacc 2	Revaccination and Control Groups	180	ICS gE
			Revaccination and Control Groups	180	Ab gE ELISA
Visit Year 1, Visit Year 2, Visit Year 3, Visit Year 4, Visit Year 5, Visit Year 6	12, 24, 36, 48, 60, 72	Post-Primary	LTFU Group CMI subset	≤ 234	ICS gE
			LTFU Group HI subset	≤ 1729 [45]	Ab gE ELISA
			HZ subset	TBD	Ab gE ELISA
		Post-Vacc 1/2	1-Additional Dose, Revaccination and Control Groups	240	ICS gE
			1-Additional Dose, Revaccination and Control Groups	240	Ab gE ELISA

Post-Primary = Post-primary vaccination during ZOSTER-006/022; Pre-Vacc = Pre Vaccination; Post-Vacc 1 = Post Vaccination 1 (post dose 1); Post-Vacc 2 = Post-Vaccination 2 (post dose 2); LTFU = Long-Term Follow-Up; CMI = Cell-Mediated Immunity; HI = Humoral Immunogenicity; HZ = Herpes Zoster; TBD = To Be Determined; ICS = Intracellular cytokine staining; gE = Glycoprotein E; Ab = Antibody; ELISA = Enzyme-linked Immunosorbent Assay.

[1] Refer to Section 6.5.8.1 for the blood sampling timepoints.

[2] Refer to Table 4 and Table 12 for description of the subsets.

[3] Refer to Table 8, Table 9 and Table 10 for the intervals between study visits for the LTFU group, the 1-Additional Dose and Control groups and for the Revaccination group, respectively.

[4] All subjects (N ≤ 14,448) entering the study will have a HI blood sample (approximately 5 mL) at Visit Month 0. For subjects in the LTFU group, who do not belong to any subset, these samples will be stored and tested for HI only if the subject develops HZ during the ZOSTER-049 study or if there are other reasons requiring the HI testing of these samples (see Section 6.5.8.1).

[45] Subjects to be included in the LTFU Group HI subset are from the immunogenicity subset in studies ZOSTER-006/022 [N = 1269 (ZOSTER-006) + 460 (ZOSTER-022) = 1729 subjects at a maximum].

Section 7.5 Contraindications to subsequent vaccination

Also, for corticosteroids, prednisone < 20 mg/day, or equivalent, is allowed. Inhaled, ~~and~~ topical ***and intra-articular cortico***steroids are allowed.

Section 7.6.1 Recording of concomitant medications/products and concomitant vaccinations

Any concomitant medications relevant to a SAE/pIMD to be reported as per protocol or administered during the study period for the treatment of a SAE/pIMD. In addition, concomitant medications relevant to SAEs and pIMD need to be recorded on the ~~e~~***E***xpedited Adverse Event report.

Section 7.6.2 Concomitant medications/products/vaccines that may lead to the elimination of a subject from ATP analyses

- Chronic administration (defined as > 14 consecutive days ***in total***) of immunosuppressants or other immune-modifying drugs at any time during the study period. For corticosteroids, this will mean prednisone ≥ 20 mg/day or equivalent.

Section 9.3.1 Time period for detecting and recording adverse events, serious adverse events and pregnancies

All AEs/SAEs leading to withdrawal from the study will be collected and recorded from the time of the first study visit Month 0 up to the last study visit/ contact at the end of the study***.

> **** If the AEs/SAEs are not related to investigational vaccine, study participation or to GSK concomitant medication/vaccine, or if not due to HZ complications, only the name (diagnosis/description) of the event will be recorded in the eCRF. No other details about AEs/SAEs will be recorded during the entire study (for the LTFU group), from Visit Month 0 until 12 months (for the Control and 1-Additional Dose groups) and from Visit Month 0 until 12 months after last HZ/su vaccination (for the Revaccination group).***

Table 22 Reporting periods for collecting safety information (for the LTFU group)

Event [a]	Visit Month 0	Visit Year 1 - 5	Study conclusion visit/ contact Year 6
**Timing of reporting**	**Month 0**	**Month 12 - 60**	**Month 72**
Reporting of SAEs related to investigational vaccine, related to study participation or to GSK concomitant medication/vaccine			
~~AEs/SAEs leading to withdrawal from the study~~			
IMCs (see Section 7.7)			
HZ complications (see Section 4.4) (including SAE information) [b]			
AEs/SAEs leading to withdrawal from the study ***(see Section 9.3.1)*** [c]			

AE = Adverse Event; SAE = Serious Adverse Event; GSK = GlaxoSmithKline; IMC = Intercurrent Medical Condition; HZ = Herpes Zoster.

[a] The reporting period of all events starts from the time the subject consents to participate in the study.

[b] A HZ complication related to a HZ episode which was initiated during the primary study or during the interval between the end of ZOSTER-006/022 and beginning of the ZOSTER-049 study is to be recorded in general medical history at the first Month 0 Visit of study ZOSTER-049.

[c] ***If the AEs/SAEs are not related to investigational vaccine, study participation or to GSK concomitant medication/vaccine, or if not due to HZ complications, only the name diagnosis/description) of the event will be recorded in the eCRF. No other details about AEs/SAEs will be recorded.***

Table 23 Reporting periods for collecting safety information (for the 1-Additional Dose, Revaccination and Control groups)

Event	Visit Month 0 Dose 1				Visit Month 2‡ Dose 2						Visit/ contact Year 1 - 5	Study conclusion visit/ contact Year 6
Timing of reporting	Month 0	Day 6 post Dose 1	Day 29 post Dose 1		Month 2‡	Day 6 post Dose 2‡	Day 29 post Dose 2‡		Month 6		Month 12 - 60	Month 72
~~AEs/SAEs leading to withdrawal from the study~~												
HZ complications (see Section 4.4) (including SAE information) [d]												
AEs/SAEs leading to withdrawal from the study ***(see Section 9.3.1)*** [e]												

[e] ***Twelve months after last HZ/su vaccination, if the AEs/SAEs are not related to investigational vaccine, study participation or to GSK concomitant medication/vaccine, or if not due to HZ complications, only the name (diagnosis/description) of the event will be recorded in the eCRF. No other details about AEs/SAEs will be recorded.***

Section 9.5.1.2 Follow-up after the subject is discharged from the study

- with other non-serious AEs**,** cases of new onset of autoimmune diseases, until study conclusion or they are lost to follow-up.

Section 11.5.3 CMI response

- The CMI vaccine response to gE will be based on the gE-specific data ~~as computed above. The lower limit of linearity (LLL) for the assay will be used as threshold for vaccine response assessment and the estimated value will be detailed in the Statistical Analysis Plan (SAP)~~. The ~~Vaccine Response (~~VR***R***~~)~~ is defined as the percentage of subjects who have ***at least***:
  - ~~at least~~ a 2-fold increase as compared to the ~~LLL, for subjects with pre-vaccination T-cell frequencies below the LLL~~***threshold 320 polypositive CD4+ T cells/10E6 CD4+ T cells, for subjects with pre-vaccination T-cell frequencies below the threshold 320 polypositive CD4+ T cells/10E6 CD4+ T cells***;
  - ~~at least~~ a 2-fold increase as compared to pre-vaccination T-cell frequencies, for subjects with pre-vaccination above the ~~LLL~~***threshold 320 polypositive CD4+ T cells/10E6 CD4+ T cells***.

Section 11.6.3.3 Analysis of persistence (immune subset of LTFU group)

Since the interval between the end of ZOSTER-006/022 studies and the start of this study will vary per subject and is dependent on receipt of approval or implementing the study in the different participating countries, some results from the first blood sample may correspond to year 5, year 6 or year 7 post vaccination.

Section 11.8.1. Sequence of analyses

At this point, the GSK central clinical team will have access to the lab data and the treatment assignment from SBIR.

2. Two intermediate analyses to assess the VE at year 2 and year 4 (LTFU and Control groups):
   - An assessment of the VE ***and immunogenicity*** will be performed when the last subjects have completed their year 2 visit/contact and when efficacy data (on confirmed HZ cases)***, immunogenicity data*** as well as related SAEs up to year 2 are available and as clean as possible. Because the analysis is purely descriptive, no adjustment on type I error is foreseen. A clinical study report is planned to be written at this time but no individual data listings will be provided.
   - An assessment of the VE ***and immunogenicity*** will be performed when the last subjects have completed their year 4 visit/contact and when efficacy data (on confirmed HZ cases)***, immunogenicity data*** as well as related SAEs up to year 4 are available and as clean as possible. Because the analysis is purely descriptive, no adjustment on type I error is foreseen. A clinical study report is planned to be written at this time but no individual data listings will be provided.

Section 13.3 Requirements for Japan

Regulatory and Ethical Considerations

The study will be conducted in accordance with "the Ministerial Ordinance on the Standards for the Conduct of Clinical Trials of Medicinal Products (Ministry of Health and Welfare [MHW]/ Ministry of Health, Labour and Welfare Notification No.28 dated 27th March, 1997)" GCP and, Article 14-3 and 80-2 of the Pharmaceutical Affairs Law ***and Evaluation and Licensing Division, Pharmaceutical Safety Bureau of MHW, Notification No. 1061, 1998***.

Study Period

The exact study period has not been confirmed at the time of this protocol. When needed and available, the study period can be communicated at local level, outside of this protocol.

If during the conduct of the study the HZ/su vaccine (GSK 1437173A) is approved in Japan, the study will then be locally amended to be conducted as a post-marketing study.

REFERENCES

Cunningham AL, Lal H, Kovac M, et al., Efficacy of the Herpes Zoster Subunit Vaccine in Adults 70 Years of Age or Older. N Engl J Med. 2016; 375(11): 1019-32.

APPENDIX A LABORATORY ASSAYS

PCR assay for confirmation of suspected case of HZ:

VZV and β-actin DNA in HZ clinical specimens will be assessed using real-time PCR detection by the 5' nuclease assay based on the Taqman probe technology.

- **Ascertainment of HZ cases including the PCR testing algorithm to classify HZ suspected cases**

~~Blood samples collected at Visit Month 0 will be tested to assess VZV serological status of subjects born in 1980 or later or before 1980 in a tropical region, and with no serological evidence of prior VZV infection available at the time of Visit Month 0, in the following cases:~~

- ~~The subject presents with suspected HZ according to criterion (1) with rash disseminated from onset and PCR confirmation of the presence of VZV DNA (see Figure 3): If the subject is found to have been seronegative at Visit Month 0 (baseline), the case will not be considered a confirmed HZ case for the efficacy analysis.~~
- ~~The subject presents with suspected HZ with rash disseminated from onset and PCR results are inconclusive (see Figure 3): If the subject is found to have been seronegative at Visit Month 0 (baseline), the case will not be considered a confirmed HZ case for the efficacy analysis.~~
- ~~The subject presents with suspected HZ according to criterion (1) with rash disseminated from onset but no rash lesion samples are available for PCR testing: If~~

~~the subject is found to have been seronegative at Visit Month 0 (baseline), the case will not be considered a confirmed HZ case for the efficacy analysis.~~

- ~~The subject presents with suspected HZ and was diagnosed with VZV-related disease according criterion (2): if the subject is found to have been seronegative at Visit Month 0 (baseline), the case will not be considered a confirmed HZ case for the efficacy analysis.~~

Figure 3 Algorithm for HZ case definition by PCR

Former Figure 3

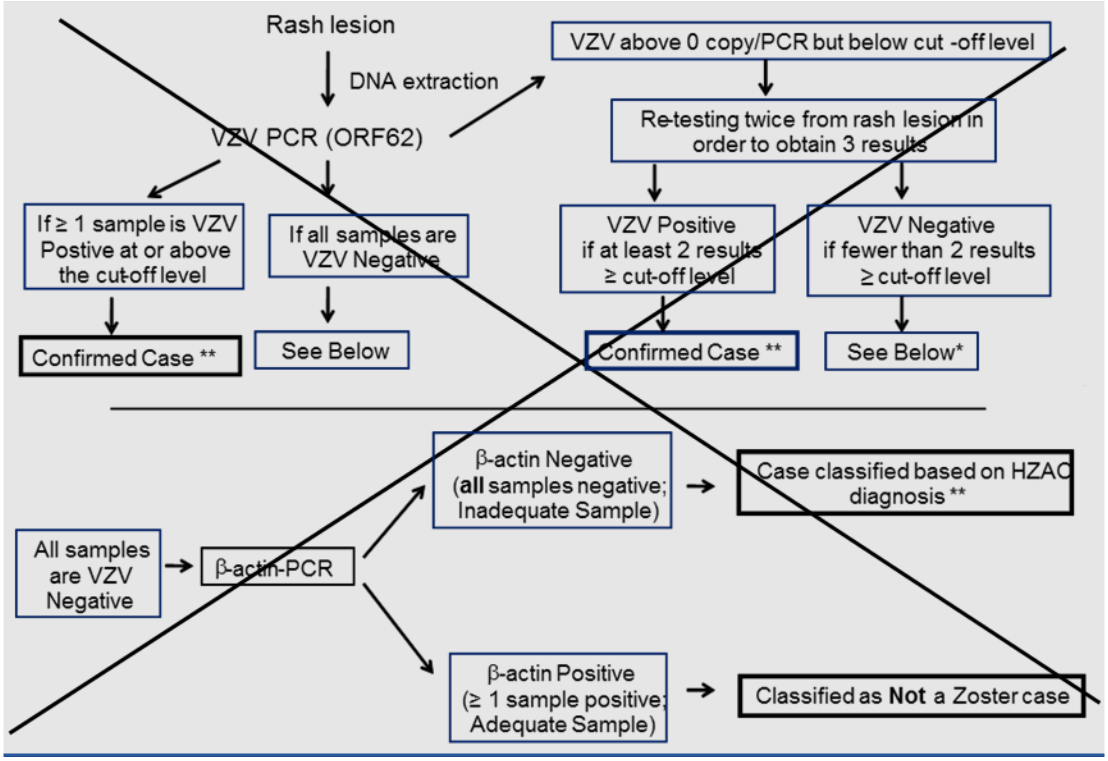

New Figure 3

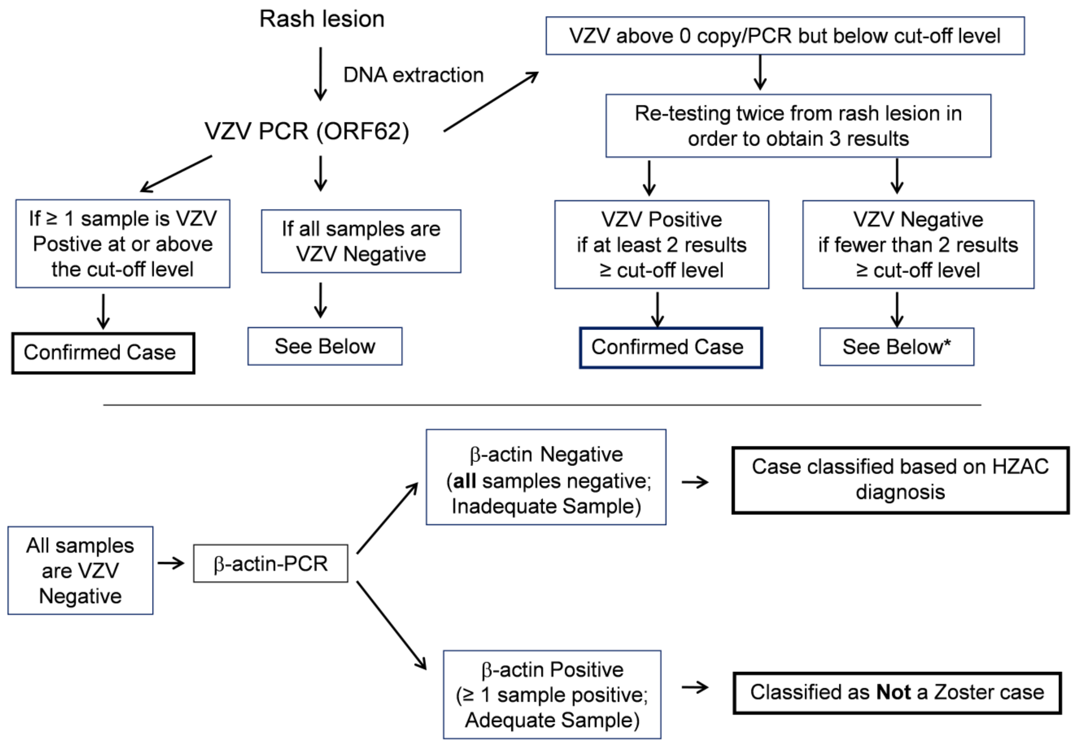

VZV: Varicella Zoster Virus; PCR: real-time P***olymerase*** C***hain*** R***eaction***; DNA: Deoxyribonucleic Acid; ORF: Open Reading Frame; ***HZAC: Herpes Zoster Ascertainment Committee***.

~~Note: The cut-off level of the VZV PCR assay is defined as the technical limit of detection of the assay (LOD of 10 VZV DNA copies; i.e., lowest concentration that can be detected by PCR in at least 95% of the tests)~~

Note: The cut-off level of the VZV PCR assay was defined as the technical limit of detection of the assay (LOD of 10 VZV DNA copies; i.e., lowest concentration that can be detected by PCR in at least 95% of the tests)

~~** Blood samples collected at Visit Month 0 prior to vaccination will be used for testing of VZV serological-status if all of the following three conditions apply:~~

- ~~Subject was diagnosed with suspected HZ, presented with disseminated rash from onset, and had VZV-positive PCR results, or VZV inconclusive PCR results, according the algorithm above; Subject was born in 1980 or later, OR was born before 1980 in a tropical region;~~
- ~~No serological evidence of prior VZV infection was available at Visit Month 0.~~

~~In these subjects, a suspected HZ case with VZV positive PCR results according to the algorithm above is not considered a confirmed case of HZ if the subject was diagnosed with suspected HZ (see Section 4.5.2) and presented with a disseminated rash from onset, and the subject was VZV seronegative at baseline.~~

~~In these subjects, a suspected HZ case with VZV inconclusive PCR results according to the algorithm above is not considered a confirmed case of HZ if the subject was diagnosed with suspected HZ according criterion (1) (see Section 4.5.2) and presented with a disseminated rash from onset, and the subject was VZV seronegative at baseline.~~

GlaxoSmithKline Biologicals	
**Clinical Research & Development**<br><br>**Protocol Administrative Change 1**	
**eTrack study number and Abbreviated Title**	201190 (ZOSTER-049 EXT:006-022)
**IND number**	BB-IND 13857
**Administrative change number:**	Administrative change 1
**Administrative change date:**	05 May 2017
**Co-ordinating author:**	PPD, Scientific writer
**Rationale/background for changes:**<br>• The back-up study contact telephone number for reporting SAEs, pregnancies and pIMDs in the US was deleted in Section 9.4.2, since that telephone number, which was originally provided to the sites as a courtesy, has been inactivated.	

Amended text has been included in *bold italics* and deleted text in ~~strikethrough~~ in the following sections:

Section 9.4.2 Contact information for reporting serious adverse events, pregnancies and pIMDs

**Study Contact for Reporting SAEs, pregnancies and pIMDs**
**Back-up Study Contact for Reporting SAEs, pregnancies and pIMDs**
24/24 hour and 7/7 day availability:<br><br>**GSK Biologicals Clinical Safety & Pharmacovigilance**<br>US sites only:<br>Fax: ~~Tel: 31 610 787 3185~~

GlaxoSmithKline Biologicals

Vaccine Value & Health Science (VVHS)
Protocol Amendment 3

**eTrack study number and Abbreviated Title**	201190 (ZOSTER-049 EXT:006-022)
**IND number**	BB-IND 13857
**EudraCT number**	2015-001778-17
**Amendment number:**	Amendment 3
**Amendment date:**	16 March 2018
**Co-ordinating author:**	PPD, Scientific Writer

Rationale/background for changes:

- This protocol is being amended following a request by the European Medicines Agency (EMA). Vaccine efficacy (VE) in prevention of Herpes Zoster (HZ) related complications (other than Postherpetic Neuralgia [PHN]) in the overall study population and VE in the prevention of PHN over each year of follow-up from one month post dose 2 in the ZOSTER-006/022 studies until the end of the ZOSTER-049 study will be assessed. The corresponding objectives and endpoints were added. In addition, as per EMA's request, a sensitivity analysis will be performed to assess the impact on VE of HZ episodes occurring during the interval between the end of the ZOSTER-006/022 studies and beginning of the ZOSTER-049.
- [redacted]
- Since the HZ/su vaccine was first approved in ***Canada and*** the United States in October 2017, the Trademarks were updated to include the trade name *Shingrix*. **(Amended 23 October 2018)**
- Typographic errors were corrected and other minor modifications were made throughout for clarification.

Amended text has been included in *bold italics* and deleted text in ~~strikethrough~~ in the following sections:

Title page

**Contributing authors**	~~• PPD, Clinical Research and Development Lead, XPE Pharma and Science, Consultant for GSK Vaccines~~<br>~~•~~ PPD PPD, Project Statistician<br>~~• PPD, Lead Study Delivery Lead~~<br>~~• PPD, Lead Study Delivery Lead~~

- PPD ~~, Lead Study Delivery Lead~~
- PPD and PPD PPD PPD Study Delivery Leads
- ~~PPD , PPD and PPD PPD SynteractHCR Benelux, Contractors for GSK Biologicals/ PPD , Study Delivery Managers~~
- PPD PPD , Vaccine Supply Coordinator
- ~~PPD , Vaccine Supply Coordinator~~
- PPD , ***Clinical*** Safety representative
- PPD ***, Clinical Safety representative***
- ~~PPD PPD , Safety representative~~
- PPD , Oversight Data Manager
- PPD PPD and PPD PPD (USA), Global Regulatory Affairs
- ~~PPD and PPD (USA), Global Regulatory Affairs~~
- PPD PPD , Global Patent
- ~~PPD , Project level Clinical Research and Development Lead, GlaxoSmithKline Biologicals, Belgian RDC~~
- PPD PPD , Clinical and Epidemiology Project Lead for Zoster, GlaxoSmithKline Biologicals, ~~Belgian~~ ***US*** RDC

GSK Biologicals' Protocol DS v14.1.1

~~Copyright 2015-~~*~~2018~~*~~ the GlaxoSmithKline group of companies. All rights reserved. Unauthorised copying or use of this information is prohibited.~~ *©2015-2018 GSK group of companies or its licensor*

Protocol Amendment 3 Sponsor Signatory Approval

**Sponsor signatory**	~~Lidia Oostvogels~~ ***Anne Schuind***, Clinical and Epidemiology Project Lead for Zoster, GlaxoSmithKline Biologicals, ~~Belgian~~ ***US*** RDC

Synopsis

**Rationale for the study and study design**	• Rationale for the study<br><br>GlaxoSmithKline (GSK) Biologicals' ~~candidate~~ ***study*** vaccine for the prevention of HZ, is a recombinant subunit (su) vaccine consisting of Varicella Zoster Virus (VZV) glycoprotein E (gE) as antigen and an adjuvant system (AS01), has been and is being evaluated in several studies in older adults and immunocompromised adults. In these studies it was shown to elicit strong cellular and humoral immune responses. Furthermore, the safety and reactogenicity profile of the ~~candidate~~ ***study*** vaccine was clinically acceptable.
**Objectives**	**Primary**<br><br>• To assess the VE in the prevention of HZ over the total duration of the ZOSTER-049 study ~~overall~~ as measured by the reduction in HZ risk in subjects ≥ 50 YOA ***overall*** at the time of first vaccination in the ZOSTER-006/022 studies.<br><br>**Secondary**<br><br>• To assess the VE in the prevention of HZ from one month post dose 2 in the ZOSTER-006/022 studies until the end of the ZOSTER-049 study as measured by the reduction in HZ risk in subjects ≥ 50 YOA ***overall*** and within each of the specified age ranges* at the time of first vaccination in the ZOSTER-006/022 studies;<br><br>• To assess the VE in the prevention of HZ over each year of follow-up from one month post dose 2 in the ZOSTER-006/022 studies as measured by the reduction in HZ risk in subjects ≥ 50 YOA ***overall*** and within each of the specified age ranges* at the time of first vaccination in the ZOSTER-006/022 studies;<br><br>• To assess the VE over the total duration of the ZOSTER-049 study ~~overall~~ in prevention of PHN in subjects ≥ 50 YOA ***overall*** and within each of the specified age ranges* at the time of first vaccination in the ZOSTER-006/022 studies;<br><br>• To assess the VE in the prevention of PHN from one month post dose 2 in the ZOSTER-006/022 studies until the end of the ZOSTER-049 study in subjects ≥ 50 YOA ***overall*** and within each of the specified age ranges* at the time of first vaccination in the ZOSTER-006/022 studies;

- ***To assess the VE over the total duration of the ZOSTER-049 study in prevention of HZ related complications (other than PHN) in subjects ≥ 50 YOA overall and within each of the specified age ranges* at the time of first vaccination in the ZOSTER-006/022 studies;***
- ***To assess the VE in the prevention of HZ related complications (other than PHN) from one month post dose 2 in the ZOSTER-006/022 studies until the end of the ZOSTER-049 study in subjects ≥ 50 YOA overall and within each of the specified age ranges* at the time of first vaccination in the ZOSTER-006/022 studies;***
- To assess persistence of humoral immune responses at Year 5, 6, 7, 8, 9 and 10 and beyond after the primary vaccination in the ZOSTER-006/022 studies in the HI subset in subjects ≥ 50 YOA ***overall*** and within each of the specified age ranges* at the time of first vaccination, in the ZOSTER-006/022 studies;
- To assess persistence of vaccine induced cell-mediated immune responses at Year 5, 6, 7, 8, 9 and 10 and beyond after the primary vaccination in the ZOSTER-006/022 studies in the CMI subset in subjects ≥ 50 YOA ***overall*** and within each of the specified age ranges* at the time of first vaccination, in the ZOSTER-006 study;

Tertiary

CCI
[redacted]

**Endpoints**	**Primary**<br>• Confirmed HZ cases (LTFU and Control groups);<br>– Confirmed HZ cases during the ***ZOSTER-049*** study.<br>**Secondary**<br>• PHN cases;<br>– PHN cases during the ***ZOSTER-049*** study and since one month post dose 2 in the previous ZOSTER-006/022 studies;<br>• ***HZ related complications (other than PHN);***<br>– ***HZ related complications (other than PHN) during the ZOSTER-049 study and since one month post dose 2 in the previous ZOSTER-006/022 studies;***<br>**Tertiary**<br>[redacted]

LIST OF APPENDICES

		PAGE
APPENDIX A	***LABORATORY ASSAYS***	***141***
APPENDIX B	***CLINICAL LABORATORIES***	***145***
APPENDIX C	***AMENDMENTS AND ADMINISTRATIVE CHANGES TO THE PROTOCOL***	***146***

LIST OF ABBREVIATIONS

[redacted]

EMA:	***European Medicines Agency***

TRADEMARKS

Trademarks not owned by the GlaxoSmithKline group of companies		Generic description
Varivax® (Merck & Co., Inc.)		Varicella vaccine consisting of live attenuated varicella-zoster virus (Oka strain)
Zostavax® (Merck & Co., Inc.)		Herpes zoster vaccine consisting of high-titre live attenuated Varicella-zoster virus (Oka strain)
~~QS-21: *Quillaja saponaria* Molina, fraction 21 (Licensed by GSK from Antigenics Inc, a wholly owned subsidiary of Agenus Inc., a Delaware, USA corporation)~~		~~Triterpene glycoside immune enhancer~~

Trademarks of the GSK group of companies		***Generic description***
Shingrix		***Herpes zoster vaccine non-live recombinant, $AS01_B$ adjuvanted***

Section 1.1 Background

GlaxoSmithKline (GSK) Biologicals' candidate ***study*** vaccine ***(Shingrix)*** for the prevention of HZ, is a recombinant subunit (su) vaccine consisting of Varicella Zoster Virus (VZV) glycoprotein E (gE) as antigen and an adjuvant system (AS01), has been and is being evaluated in several studies in older adults and immunocompromised adults. In these studies it was shown to elicit strong cellular and humoral immune responses. Furthermore, the safety and reactogenicity profile of the candidate ***study*** vaccine was clinically acceptable.

An indication in adults ≥ 50 YOA was filed for registration. HZ/su (trade name Shingrix) was first approved in the United States and Canada in October 2017.

Section 2.1 Primary Objective

- To assess the VE in the prevention of HZ over the total duration of the ZOSTER-049 study ~~overall~~ as measured by the reduction in HZ risk in subjects ≥ 50 YOA ***overall*** at the time of first vaccination in the ZOSTER-006/022 studies.

Section 2.2 Secondary Objective

- To assess the VE in the prevention of HZ from one month post dose 2 in the ZOSTER-006/022 studies until the end of the ZOSTER-049 study as measured by the reduction in HZ risk in subjects ≥ 50 YOA ***overall*** and within each of the specified age ranges* at the time of first vaccination in the ZOSTER-006/022 studies;
- To assess the VE in the prevention of HZ over each year of follow-up from one month post dose 2 in the ZOSTER-006/022 studies as measured by the reduction in

HZ risk in subjects ≥ 50 YOA ***overall*** and within each of the specified age ranges* at the time of first vaccination in the ZOSTER-006/022 studies;

- To assess the VE over the total duration of the ZOSTER-049 study ~~overall~~ in prevention of PHN in subjects ≥ 50 YOA ***overall*** and within each of the specified age ranges* at the time of first vaccination in the ZOSTER-006/022 studies;
- To assess the VE in the prevention of PHN from one month post dose 2 in the ZOSTER-006/022 studies until the end of the ZOSTER-049 study in subjects ≥ 50 YOA ***overall*** and within each of the specified age ranges* at the time of first vaccination in the ZOSTER-006/022 studies;
- ***To assess the VE over the total duration of the ZOSTER-049 study in prevention of HZ related complications (other than PHN) in subjects ≥ 50 YOA overall and within each of the specified age ranges* at the time of first vaccination in the ZOSTER-006/022 studies;***
- ***To assess the VE in the prevention of HZ related complications (other than PHN) from one month post dose 2 in the ZOSTER-006/022 studies until the end of the ZOSTER-049 study in subjects ≥ 50 YOA overall and within each of the specified age ranges* at the time of first vaccination in the ZOSTER-006/022 studies;***
- To assess persistence of humoral immune responses at Year 5, 6, 7, 8, 9 and 10 and beyond after the primary vaccination in the ZOSTER-006/022 studies in the HI subset in subjects ≥ 50 YOA ***overall*** and within each of the specified age ranges* at the time of first vaccination, in the ZOSTER-006/022 studies;
- To assess persistence of vaccine induced cell-mediated immune responses at Year 5, 6, 7, 8, 9 and 10 and beyond after the primary vaccination in the ZOSTER-006/022 studies in the CMI subset in subjects ≥ 50 YOA ***overall*** and within each of the specified age ranges* at the time of first vaccination, in the ZOSTER-006 study;

Section 2.3 Tertiary Objective

CCI [redacted]

Table 14 Cell-Mediated Immunity (CMI)

System	Component	Challenge	Method	Unit	Cut-off	Laboratory
PBMC	[redacted]					***GSK Biologicals****

PBMC = Peripheral Blood Mononuclear Cells; IL-2 = Interleukin-2; TNF = Tumor Necrosis Factor; IFN = Interferon; gE = Glycoprotein E; ICS = Intracellular cytokine staining; [redacted]***;*** N/A = Not Applicable.

**** see Table 28 in Appendix B.***

Table 16 Immunological read-outs

Blood sampling timepoint [1]			Subset Name [2]	Approximate No. of Subjects	Component
**Visit No.[3]**	**Month**	**Timing**			
Visit Month 0	0	Post-Primary	All Subjects	≤ 14448 [4]	Ab gE ELISA
			LTFU Group CMI subset	≤ 234	ICS gE
		Pre-Vacc	1-Additional Dose, Revaccination and Control Groups	240	***gE*** ICS ~~gE~~***&*** [redacted]
Visit Month 1	1	Post-Vacc 1	1-Additional Dose, Revaccination and Control Groups	240	***gE*** ICS ~~gE~~***&*** [redacted]
			1-Additional Dose, Revaccination and Control Groups	240	Ab gE ELISA
Visit Month 3	3	Post–Vacc 2	Revaccination and Control Groups	180	***gE*** ICS ~~gE~~***&*** [redacted]
			Revaccination and Control Groups	180	Ab gE ELISA
Visit Year 1, Visit Year 2, Visit Year 3, Visit Year 4, Visit Year 5, Visit Year 6	12, 24, 36, 48, 60, 72	Post-Primary	LTFU Group CMI subset	≤ 234	**gE** ICS ~~gE~~**g**
			LTFU Group HI subset	≤ 1729 [5]	Ab gE ELISA
			HZ subset	TBD	Ab gE ELISA
		Post-Vacc 1/2	1-Additional Dose, Revaccination and Control Groups	240	***gE*** ICS ~~gE~~***&*** [redacted]
			1-Additional Dose, Revaccination and Control Groups	240	Ab gE ELISA

Post-Primary = Post-primary vaccination during ZOSTER-006/022; Pre-Vacc = Pre Vaccination; Post-Vacc 1 = Post Vaccination 1 (post dose 1); Post-Vacc 2 = Post-Vaccination 2 (post dose 2); LTFU = Long-Term Follow-Up; CMI = Cell-Mediated Immunity; HI = Humoral Immunogenicity; HZ = Herpes Zoster; TBD = To Be Determined; ICS = Intracellular cytokine staining; gE = Glycoprotein E; Ab = Antibody; ELISA = Enzyme-linked Immunosorbent Assay***;*** [redacted] ..

[6] [redacted] ***on available PBMC following gE ICS analysis.***

Section 6.6.5 Immunological correlates of protection

No generally accepted immunological correlate of protection against HZ has been demonstrated so far for the gE antigen used in the HZ/su ~~candidate~~ ***study*** vaccine.

Section 7.1 Description of study vaccine

The ~~candidate~~ ***study*** vaccine/product to be used has been developed and manufactured by GSK Biologicals.

Section 9.2.1 Pregnancy

Note: the 22 weeks cut-off in gestational age is based on WHO-ICD 10 noted in the EMA ***European Medicines Agency (***EMA***)*** Guideline on pregnancy exposure [EMA, 2006]. It is recognised that national regulations might be different.

Section 11.1 Primary endpoint

- Confirmed HZ cases (LTFU and Control groups);
  - Confirmed HZ cases during the ***ZOSTER-049*** study.

Section 11.2 Secondary endpoint

- PHN cases;
  - PHN cases during the ***ZOSTER-049*** study and since one month post dose 2 in the previous ZOSTER-006/022 studies;
- ***HZ related complications (other than PHN);***
  - ***HZ related complications (other than PHN) during the ZOSTER-049 study and since one month post dose 2 in the previous ZOSTER-006/022 studies;***

Section 11.3 Tertiary endpoint

CCI [redacted]

Section 11.6.2 Humoral immune response

CCI [redacted]

Section 11.7.2. Analysis of efficacy (LTFU and Control groups)

The primary analysis will be to assess the VE during this study and will use the historical control estimates adjusted for age at randomisation during the ZOSTER-006/022 studies. The method using the historical control estimates will be further explained in the ***Statistical Analysis Plan (***SAP***)***.

Any available information for the diagnosis of HZ episodes (clinical diagnosis, PCR, laboratory methods, etc.) during the interval between the end of the ZOSTER-006/022 studies and beginning of the ZOSTER-049 study is to be collected. Confirmation of all of these cases in the exact way as what will be done for cases collected during the clinical study follow-up periods of the ZOSTER-006, ZOSTER-022 and ZOSTER-049 will not be possible. Therefore, these cases will not be part of all efficacy analyses. A sensitivity analysis to assess the impact of these cases on the VE will be performed (this will be described in the SAP).

will be described in the SAP).

The purpose of these analyses will be descriptive and therefore the CIs will not be adjusted for multiple testing.

The analyses will be performed for confirmed HZ ~~case and for PHN~~ case*s*, for PHN ***and for complications (other than PHN)***.

Section 11.7.3.1.2. Cell-mediated immune response

- Descriptive statistics of the frequency of CD4+ T-cells ***expressing*** at least two activation markers (~~from~~ among IFN-γ, IL-2, TNF-α, CD40L) for gE-specific stimulation.

CCI [redacted]

Section 11.7.3.3.2 Cell-mediated immune response

For the yearly prediction of the ~~GMCs~~ ***CMI*** (year 5 to year 10 and beyond): the analysis of CMI persistence will be based on the ATP cohort for persistence adapted for each year.

Section 11.9.1 Sequence of analyses

2. Two intermediate analyses to assess the VE at year 2 and year 4 (LTFU and Control groups):
   – An assessment of the VE and immunogenicity will be performed when the last subjects have completed their year 2 visit/contact and when efficacy data (on confirmed HZ cases), immunogenicity data as well as related SAEs up to year 2 are available and as clean as possible. Because the analysis is purely descriptive, no adjustment on type I error is foreseen. A clinical study report is planned to be written at this time but no individual data listings will be provided. ***Please refer to the SAP***
   – An assessment of the VE and immunogenicity will be performed when the last subjects have completed their year 4 visit/contact and when efficacy data (on confirmed HZ cases), immunogenicity data as well as related SAEs up to year 4 are available and as clean as possible. Because the analysis is purely descriptive, no adjustment on type I error is foreseen. A clinical study report is planned to be written at this time but no individual data listings will be provided. ***Please refer to the SAP***

Section 13.1 Requirements for France

It is expressly specified that GSK and/or the Sponsor can make available to the public the results of the Study by the posting of the said results on a website of the GSK Group named Clinical Trial Regist~~r~~***e***~~d~~***r*** (CTR) including the registration of all the clinical trials conduct by the GSK Group and this before or after the publication of such results by any other process.

APPENDIX A LABORATORY ASSAYS

Intracellular cytokine staining (ICS):

CMI responses will be performed by GSK Biologicals (or designated laboratory) on thawed Peripheral Blood Mononuclear Cells (PBMCs) by ICS. The assay will be performed on samples collected during the course of the study. This assay provides information on the frequency of CD4 T cells responding to culture medium or antigens (gE peptide pool) by ~~secreting cytokine~~ ***expressing*** molecules involved in immunity such as IFN-γ, IL-2, TNF-α, and CD40L ***(here considered as activation markers)***.

Briefly, PBMC collected from the subjects are stimulated for two hours using culture medium (for evaluation of the non-specific response), a pool of overlapping peptides covering the entire sequence of the vaccine antigen gE. Then, an intracellular block (brefeldin A) is added to inhibit cytokine secretion for a subsequent overnight incubation. Cells are then harvested, stained for surface markers (CD3, CD4 and CD8) and fixed. The fixed cells are then permeabilised and stained with ~~anti-cytokine~~ Abs ***specific to the activation markers assessed,*** washed and analyzed by cytofluorometry.

The results of ICS assays are expressed as the frequency of specific CD4 T cells per million total CD4 T cells.

CCI [redacted]

GlaxoSmithKline Biologicals

Vaccine Value & Health Science (VVHS)
Protocol Amendment 4

**eTrack study number and Abbreviated Title**	201190 (ZOSTER-049 EXT:006-022)
**IND number**	BB-IND 13857
**EudraCT number**	2015-001778-17
**Amendment number:**	Amendment 4
**Amendment date:**	23 October 2018
**Co-ordinating author:**	PPD, Scientific Writer, XPE Belgium, Contractor for GSK

Rationale/background for changes:

(from page 4)

- This amendment relates to the time frame of completing the Zoster Brief Pain Inventory (ZBPI) questionnaire. This change will ensure thorough reporting of postherpetic neuralgia cases. Subjects with suspected herpes zoster (HZ) are required to complete the questionnaire until a 4-week pain-free period is documented and beyond Day HZ-91, if applicable. This amendment removes the stipulation "or until Day HZ-91" and therefore, if pain develops after Day HZ-91 to capture of cases of postherpetic neuralgia according to the protocol definition.
- Reporting of cytotoxic chemotherapy as a concomitant medication was clarified.
- Other minor changes include correcting and updating text regarding country approvals of Shingrix, and correcting minor typographical errors.

Amended text has been included in *bold italics* and deleted text in ~~strikethrough~~ in the following sections:

Section 4.5.1 Definitions

For all subjects in case of a suspected or confirmed case of HZ:

Zoster Brief Pain Inventory (ZBPI) questionnaire: To be completed by subjects with suspected HZ (or subject's caregiver) on Day HZ-0 (Visit HZ-1) and daily from Day HZ-1 (day after the Visit HZ-1) up to Day HZ-28, and weekly from Day HZ-29 onwards until a 4-week pain-free period is documented ~~or until Day HZ-91.~~

Section 4.5.2 Evaluation of suspected case of HZ

Changed this sentence to a subheader: 4.5.2.1: For clinically diagnosed suspected HZ cases, the following will take place at Visit HZ-1:

Deleted or until Day HZ-91 in 3 places:

- 28 days after HZ-associated pain ceases ~~or until Day HZ-91~~.

Have you had any pain caused by your shingles in the last 24 hours' (item 1) at each assessment during that entire period) ~~or until Day HZ-91~~

If HZ-associated pain ceases (defined as a 28-day [or 4-week] pain free period) ~~or until Day HZ-91~~, the study staff/investigator will inform the subject/ subject's caregiver to stop completing the ZBPI questionnaires and will provide instructions for the subject to return the completed questionnaires to the study site.

Text was edited as follows:

If a 4-week pain-free period is achieved and the HZ rash resolves, subsequent follow-up visits or contacts related to this case of HZ will be cancelled ***meaning that c***~~C~~ollection of subsequent HZ episode-related information will be stopped and no further information on that ***particular*** suspected HZ episode will be encoded in the clinical database. ***However, if pain reappears in the same area after a 4-week pain-free period and is not accompanied by a new HZ rash, it will be assigned to the previous HZ-episode. Visits/contacts will restart with Day HZ-0 defined as the first visit of the assigned episode, prior to the pain free period.***

Follow-up of HZ-associated pain persisting beyond Visit HZ-7 (Day HZ-91) or other complications will be done at monthly contacts between the subject and the investigator and/or investigator's delegate.

~~If pain continues beyond Day HZ-91, and rash has resolved, collection of information on pain will end for study purposes and the subject will be followed as per the investigators discretion. Once a pain free period of 4 weeks has been documented or information on pain till Day HZ-91 has been collected, subjects with ongoing symptoms related to suspected HZ episode including ongoing pain will be followed as per the investigator discretion~~.

Changed this sentence to a subheader: 4.5.2.2: The following will take place at each visit or contact that occurs for each episode:

Other subheading numbers in this section changed when new subheadings were added:

~~4.5.2.1~~ ***4.5.2.3*** Evaluation of severity of HZ-associated pain using the Zoster Brief Pain Inventory

~~4.5.2.2~~ ***4.5.2.4*** Confirmation of a suspected case of HZ

~~4.5.2.2~~.1 ***4.5.2.4.1*** Confirmation of suspected HZ by PCR

~~4.5.2.2.2~~ ***4.5.2.4.2*** Confirmation of suspected HZ by the HZAC

Section 4.5.3 Follow-up of suspected HZ cases and HZ-associated pain

Data will be collected on all suspected HZ cases that occur from Visit Month 0 until the study conclusion Visit. For each suspected case of HZ that the investigator concludes is clinically consistent with HZ, data on HZ-associated pain (using ZBPI questionnaires completed by the subject) will be collected ***daily*** until Day- HZ-28, and ***weekly*** from Day HZ-29 until the subject has no HZ-associated pain for 4 consecutive weeks, ~~or until Day HZ-91.~~

Visits/contacts will also restart according to the schedule in Table 7 with Day HZ-0 defined as the first visit of the assigned episode, prior to the pain free period. Follow-up ~~will end at Day HZ-91 as~~ ***is*** described in Section 4.5.2.

Notes to Table 7 Study Procedure

† Subjects with suspected HZ will be asked to complete the ZBPI questionnaire at Day HZ-0 (Visit HZ-1) to rate HZ-associated pain within the last 24 hours (If the time between the HZ onset and clinical evaluation at Visit HZ-1 is greater than 24 hours, the subject will be asked to complete a second ZBPI also for the elapsed time between the HZ onset and 24 hours before Visit HZ-1); daily from Day HZ- 1 to Day HZ-28, and weekly from Day HZ-29 onwards until a 4-week pain-free period is documented ~~or until Day HZ-91~~.

Added † to the column heading for

**Visit HZ-7**<br>**Day HZ-91†**

Note to Table 11 Intervals between visits/…

Note: If HZ-associated pain ceases (i.e., after a 4-week pain-free period is documented) and the HZ rash resolves, subsequent follow-up HZ visits or contacts will be cancelled (see Section 4.5.2). Follow-up of HZ-associated pain persisting beyond Visit HZ-7 (Day HZ-91) or other complications ***will be done at monthly contacts between the subject and the investigator and/or investigator's delegate.*** ~~will end for study purposes and the subject will be followed as per the investigators discretion.~~

Section 6.5.1 Informed Consent

For subjects with suspected HZ at Visit HZ-1 (i.e., either ongoing cases or subjects developing a new case), an informed consent addendum or update may be required to inform these subjects of the change in the follow-up period for pain assessment questionnaire.

Section 7.6.2 Concomitant medications/products/vaccines that may lead to the elimination of a subject from ATP analyses

Added: ***Administration of cytotoxic chemotherapy at any time during the study period***

Section 13.3 Requirements for Japan

Study Period

Shingrix was approved in Japan on 23 March 2018, after study start. Therefore, this study is being conducted as a post-marketing study from the approval date in Japan.

Minor changes include:

Title page:

Co-ordinating Author: added ***GSK*** Scientific Writer to PPD and added PPD***, Scientific Writer XPE Belgium, Contractor for GSK***

Contributing Authors:

Added PPD, Clinical Research and Development Lead and deleted PPD

- Added ***and*** PPD, Study Delivery Leads and deleted: PPD PPD Study Delivery Lead, Synteract, HCR Benelux, Contractor for GSK Biologicals
- added PPD**,** Clinical Laboratory Sciences Study Manager and deleted PPD ~~,~~

Changed:

- PPD, Clinical Safety representative
- PPD, Clinical Safety representative

To:

- PPD and PPD, Clinical Safety representatives
- Changed: PPD, Clinical Safety Representative Physician
- Added PPD and deleted PPD (USA), Global Regulatory Affairs
- Added PPD and, Lead Statisticians

Section 1.1. Background

An indication in adults ≥ 50 YOA was filed for registration. HZ/su (trade name Shingrix) was first approved in ***Canada and*** the United States ~~and Canada~~ in October 2017.

Section 13.3 Requirements for Japan

~~If during the conduct of the study the HZ/su vaccine (GSK 1437173A) is approved in Japan, the study will then be locally amended to be conducted as a post-marketing study.~~

Shingrix was approved in Japan on 23 March 2018, after study start. Therefore, this study is being conducted as a post-marketing study from the approval date in Japan.

Changes to APPENDIX C: Amendments And Administrative Changes To The Protocol

Protocol Administrative Change 1 date

Date of Administrative Change ~~19 February 2016~~ ***05 May 2017***

- Amendment 3 rationale in appendix section

Since the HZ/su vaccine was first approved in ***Canada and*** the United States ~~and Canada~~ in October 2017, the Trademarks were updated to include the trade name *Shingrix*.

GlaxoSmithKline Biologicals	
**Clinical Research & Development**<br><br>**Protocol Administrative Change 2**	
**eTrack study number and Abbreviated Title**	201190 (ZOSTER-049 EXT:006-022)
**IND number**	BB-IND 13857
**Administrative change number:**	Administrative change 2
**Administrative change date:**	11 February 2019
**Co-ordinating author:**	PPD, Scientific writer
**Rationale/background for changes:**<br><br>The Japan Vaccine Company will no longer be in operation as of 01 April 2019. Therefore, the reference to "Japan Vaccine Co., Ltd." has been changed to "GSK Japan".	

Amended text has been included in *bold italics* and deleted text in ~~strikethrough~~ in the following sections:

Signature page (page 7): ~~Japan Vaccine Company, *Ltd*~~ ***GSK Japan*** **representative name, function and title**

- **Authors:** deleted Study Delivery Lead~~, and~~ PPD,

Section 13.3 Requirements for Japan

Clinical Trial Notification to Regulatory Authority

~~Japan Vaccine Company, Ltd~~ ***GSK Japan*** will submit the CTN to the regulatory authorities in accordance with Article 80-2 of the Pharmaceutical Affairs Law before conclusion of any contract for the conduct of the study with study sites.

Other changes: Footer on page 2: *©2015-201~~8~~**9** GSK group of companies or its licensor*

GlaxoSmithKline Biologicals SA

Vaccines R &D

Protocol Amendment 5

**eTrack study number and Abbreviated Title**	201190 (ZOSTER-049 EXT:006-022)
**IND number**	BB-IND 13857
**EudraCT number**	2015-001778-17
**Amendment number:**	Amendment 5
**Amendment date:**	11 May 2020
**Co-ordinating author:**	PPD, Scientific Writer

Rationale/background for changes:

This protocol amendment 5 outlines measures that may be applicable during special circumstances (e.g., during COVID-19 pandemic). The purpose of the amendment is to introduce measures that may allow protection of subject's welfare and safety, as well as maintaining the integrity of the study.

This amendment is considered substantial based on the criteria defined in Article 10(a) of Directive 2001/20/EC of the European Parliament and the Council of the European Union because it significantly impacts the safety of subjects or/nor the scientific value of the study.

As much as possible all study specified visits and procedures should be completed according to the protocol, taking into account clinical judgment and local public health guidance to protect the safety of staff and subjects.

Section 6.6 outlines the measures which include allowing flexibility in schedule and procedures to optimize site staff safety, patient safety and to preserve study integrity.

Other changes include,

- The classification of HZ cases by HZAC has been clarified with the addition of "not able to decide" to be classified as "not HZ" to Section 4.5.2.4.2 for confirmation of suspected HZ by the HZAC.
- The timelines concerning data entry into the eCRF for France in Section 13.1 have been updated to align with the timelines presented in the data management plan.

Amended text has been included in *bold italics* and deleted text in ~~strikethrough~~ in the following sections:

List of abbreviations

COVID-19: ***Coronavirus Disease 2019***

Synopsis and Section 3. Study design overview

Refer to Section 6.6 for study procedures to be considered during special circumstances.

Section 4.5.2.4.2. Confirmation of suspected HZ by the HZAC

All suspected HZ cases will be referred to the HZAC. The HZAC will classify all referred cases as either "HZ" or "not HZ" ***or "not able to decide".***

A suspected case of HZ will be considered as "HZ" if the HZAC members concur unanimously; otherwise, it will be classified as "not HZ". ***A case of "not able to decide" will be classified as "not HZ".*** As described above, the HZAC case assignment will only be considered as the final case assignment if definitive PCR results are not available. Further details will be provided in the HZAC charter.

Section 6.4. Outline of study procedures

Refer to Section 6.6 for study procedures to be considered during special circumstances.

Section 6.6. Study procedures during special circumstances

During special circumstances (e.g., COVID-19 pandemic), the specific guidance from local public health and other competent authorities regarding the protection of individuals' welfare must be applied. For the duration of such special circumstances, the following measures may be implemented for enrolled subjects:

The impact of special circumstances to the study conduct will be documented in the clinical study report.

- ***Yearly site visits/contact may be made by other means of virtual contact or home visit, if appropriate.***
- ***Biological samples may be collected at a different location* other than the study site or at subject's home. Biological samples should not be collected if they cannot be processed in a timely manner or appropriately stored until the intended use.***

**** It is the investigator's responsibility to identify an alternate location. The investigator should ensure that this alternate location meets ICH GCP requirements, such as adequate facilities to perform study procedures, appropriate training of the staff and documented delegation of responsibilities in this location. This alternate location should be covered by proper insurance for the conduct of study on subjects by investigator and staff at a site other than the designated study site. Refer to EMA Guidance on the Management of Clinical Trials during the COVID-19 (Coronavirus) pandemic (version 3, 28 April 2020) or other relevant authority guidance for more details.***

- ***If despite best efforts it is not possible to collect the biological samples within the interval predefined in the protocol (see Table 8 to Table 10), then the allowed interval may be extended by 30 days as outlined from Table 12 to Table 14.***
  - ***The visit can be replaced by a phone contact or other means of virtual contact and will be performed in the interval predefined in the protocol and the blood sampling can be performed according to the interval allowed during special circumstances as shown from Table 12 to Table 14.***

Table 12 Intervals for blood sampling for the LTFU group during special circumstances

Interval for blood sampling	Optimal length of interval (in months)	Allowed interval during normal circumstances (range in days)	*Allowed interval during special circumstances (range in days)*
Visit Month 0 → Blood sampling Year 1	12 months	335 – 395	335 ***– 425***
Visit Month 0 → Blood sampling Year 2	24 months	700 – 760	700 ***– 790***
Visit Month 0 → Blood sampling Year 3	36 months	1065 – 1125	1065 ***– 1155***
Visit Month 0 → Blood sampling Year 4	48 months	1430 – 1490	1430 ***– 1520***
Visit Month 0 → Blood sampling Year 5	60 months	1795 – 1855	1795 ***– 1885***
Visit Month 0 → Blood sampling Year 6	72 months	2160 – 2220	2160 ***– 2250***

Note: Investigator should prioritize performing the blood sampling as close to the optimal window as possible.

Table 13 Intervals for blood sampling for the 1-Additional Dose and Control groups during special circumstances

Interval for blood sampling	Optimal length of interval (in months)	Allowed interval during normal circumstances (range in days)	*Allowed interval during special circumstances (range in days)*
Visit Month 0 → Blood sampling Year 1	12 months	335 – 395	335 ***– 425***
Visit Month 0 → Blood sampling Year 2	24 months	700 – 760	700 ***– 790***
Visit Month 0 → Blood sampling Year 3	36 months	1065 – 1125	1065 ***– 1155***
Visit Month 0 → Blood sampling Year 4	48 months	1430 – 1490	1430 ***– 1520***
Visit Month 0 → Blood sampling Year 5	60 months	1795 – 1855	1795 ***– 1885***
Visit Month 0 → Blood sampling Year 6	72 months	2160 – 2220	2160 ***– 2250***

Note: Investigator should prioritize performing the blood sampling as close to the optimal window as possible.

Table 14 Intervals for blood sampling for the Revaccination group during special circumstances

Interval for blood sampling	Optimal length of interval (in months)	Allowed interval during normal circumstances (range in days)	*Allowed interval during special circumstances (range in days)*
Visit Month 2 → Blood sampling Year 1	12 months	335 – 395	335 ***– 425***
Visit Month 2 → Blood sampling Year 2	24 months	700 – 760	700 ***– 790***
Visit Month 2 → Blood sampling Year 3	36 months	1065 – 1125	1065 ***– 1155***
Visit Month 2 → Blood sampling Year 4	48 months	1430 – 1490	1430 ***– 1520***
Visit Month 2 → Blood sampling Year 5	60 months	1795 – 1855	1795 ***– 1885***
Visit Month 2 → Blood sampling Year 6	72 months	2160 – 2220	2160 ***– 2250***
Note: Investigator should prioritize performing the blood sampling as close to the optimal window as possible.			

- ***Visits for suspected HZ may take place in a different location* other than the study site or at subject's home. If this is not feasible, then the medical evaluation of suspected HZ may take place virtually with documentation of all the signs and symptoms as outlined in Table 7 and Table 11.***
  - ***If the subject is not able to immediately contact the study staff/investigator to evaluate/ clinically diagnose the suspected HZ case, the subject should be encouraged to document all the signs and symptoms, HZ related treatments and record the progression of the rash and share it with the investigator when possible.***
  - ***Digital photographs can be taken by the study staff/investigator at a different location* other than the study site or at subject's home. If this is not feasible, subjects might be asked to take photographs of their suspected HZ lesions themselves. The photographs will be transferred to the investigator via email or other modes of virtual contact when possible.***
  - ***HZ specific diary cards may be transmitted from and to the site by electronic means and/or conventional mail.***
  - ***ZBPI questionnaire may be transmitted from and to the site by electronic means and/or conventional mail. If this is not feasible, the study staff/investigator can conduct ZBPI questionnaire by telephone contact. Study staff/investigator will read the questions (verbatim) and transcribe the subject's responses on the questionnaires.***

Impact on the modified TVC for efficacy and ATP for efficacy and immunogenicity (humoral and CMI) will be determined on a case by case basis.

Section 13.1. Requirements for France

Concerning Data entry into the eCRF

9. That the Investigator and the staff of the investigator centre enter the data of the eCRF related to a patient visit in the ~~3~~ ***10*** days following the date of the patient visit or, for the patient test results, in the ~~3~~ ***10*** days following the reception of the results of such tests. ***For a subject last visit, the recommended time frame for data entry is 5 working days***.
10. That the Investigator resolves and returns to GSK the data queries issued by GSK or a service provider designated by GSK within ~~7~~ ***10*** days after the reception of the request of clarification or in a period of ~~one (1)~~***5*** day***s*** during the final stage of clarification of the data base or in such other period as provided by GSK and/or a company designated by GSK.
