# Supplementary material for: Final analysis of the ZOE-LTFU trial to 11 years post-vaccination: efficacy of the adjuvanted recombinant zoster vaccine against herpes zoster and related complications
Source: eClinicalMedicine. 2025 May 9;83:103241. doi: 10.1016/j.eclinm.2025.103241 (PMC12235393; doi:10.1016/j.eclinm.2025.103241)
Supplement: Appendix [file mmc2.docx]

**Final analysis of the ZOE-LTFU trial to 11 years post-vaccination: efficacy of the adjuvanted recombinant zoster vaccine against herpes zoster and related complications**

**Table of contents**

[Study group 4](#_Toc185238175)

[Eligibility criteria for the ZOE long term follow-up (ZOE-LTFU) study 5](#_Toc185238176)

[Inclusion criteria 5](#_Toc185238177)

[Exclusion criteria 5](#_Toc185238178)

[Primary and secondary objectives 6](#_Toc185238179)

[Primary objective 6](#_Toc185238180)

[Secondary objectives 6](#_Toc185238181)

[Definitions of non-PHN complications 8](#_Toc185238182)

[Criteria for According to Protocol (ATP) cohort for analysis of persistence of immunogenicity (LTFU group) 9](#_Toc185238183)

[Safety analysis: protocol amendment 10](#_Toc185238184)

[Appendix Table 1. Vaccine efficacy against HZ from 1 month after the second vaccine dose to the end of the ZOE-LTFU study in each study year, overall and by age strata (mTVC) 11](#_Toc185238185)

[Appendix Table 2. Vaccine efficacy against HZ from 1 month after the second vaccine dose to the end of the ZOE-LTFU study: sensitivity analysis including potential HZ cases identified during the gap period (mTVC) 15](#_Toc185238186)

[Appendix Table 3. Description of PHN and other HZ-associated complications during ZOE-LTFU (mTVC) 16](#_Toc185238187)

[Appendix Table 4. Vaccine efficacy against PHN from 1 month after the second vaccine dose to the end of the ZOE-LTFU study overall and by age strata (mTVC) 17](#_Toc185238188)

[Appendix Table 5. Vaccine efficacy against complications other than PHN from 1 month after the second vaccine dose to the end of the ZOE-LTFU study overall and by age strata (mTVC) 18](#_Toc185238189)

[Appendix Table 6. Seropositivity rates and GMCs of anti-gE antibody overall and by age (adapted ATP cohort, LTFU group) 19](#_Toc185238190)

[Appendix Table 7. Vaccine response rates for anti-gE antibody by age stratum for each year of follow-up (adapted ATP cohort, LTFU group) 21](#_Toc185238191)

[Appendix Table 8. Mean geometric increase of anti-gE antibody by age stratum for each year of follow-up (adapted ATP cohort, LTFU group) 23](#_Toc185238192)

[Appendix Table 9. Frequency of gE-specific CD4[2+] T cells by age stratum for each year of follow-up (adapted ATP cohort, LTFU group) 25](#_Toc185238193)

## Study group

**Investigators:** Abul Kashem Munir, Agnes Csuth, Agnes Himpel-Boenninghoff, Aino Forsten, Airi Poder, Ake Olsson, Alain Baty, Alain Boye, Alen Jambrecina, Alex Rodríguez Badia, Alexander Thompson, Andrea Gori, Anitta Ahonen, Anna Vilella Morato, Anthony Cunningham, Antje Dahmen, Axel Schaefer, Azhar Toma, Barry Lubin, Beate Moeckesch, Beatrice Gerlach, Benita Ukkonen, Benjamin Lasko, Benoit Daguzan, Bernhard Schmitt, Bo Liu, Brian Webster, Bruce Rankin, Calvin Powell, Carlos Brotons Cuixart, Carol Pretswell, Catherine Vaillancourt, Cecil Farrington, Charles Andrews, Chester Fisher, Chigomezgo Munthali, Chiu-Shong Liu, Chong-Jen Yu, Christian Duroy, Christian Schubert, Christiane Klein, Christine Cerna, Christine Grigat, Christophe Genies, Christopher Lucasti, Cláudia Murta de Oliveira, Claus Keller, Clovis Cunha, Concepcion Nunez Lopez, Covadonga Caso, Cristiano Zerbini, Dae Won Park, Damien Mcnally, Dan Curiac, David Francyk, David García Vidal, David Shu Cheong Hui, Denis Taminau, Domenico Montu, Dominique Saillard, Donald Quinn, Duane Wombolt, Edmund Kwok Yiu Sha, Elina Sirnela-Rif, Elisabeth Barberan, Eric St-Amour, Eriko Kinugasa, Ernie Riffer, Essam Abdulhakim, Eugene Athan, Eun-Ju Choo, Eva Ackefelt Frick, Felix Proepper, Ferdinandus de Looze, Francesco Schioppa, François Brault, Frederick Martin, Gabriele Illies, Georg Plassmann, George Freeman, George Raad, Gerald Shockey, Giancarlo Icardi, Giuseppe Fiore, Guglielmo Migliorino, Guy Tellier, Hanna Karhusaari, Hans-Joachim Koenig, Hee Jin Cheong, Hiroaki Ogata, Hirohiko Sueki, Holly Dushkin, Hsiao-Ting Chang, Huey-Shinn Cheng, Hyo Youl Kim, Ignacio Bardón Fernández-Pacheco, Ilkka Seppa, Irina Zahharova, Iris Gorfinkel, Isaac Marcadis, Isabelle Schenkenberger, Jacob Lee, Jan Dutz, Janice Patrick, Javier Diez-Domingo, Jean Beytout, Jean-Sebastien Gauthier, Jeannette Janzen, Jeffrey Zacher, Jérôme Nuel, Jessica Claveau, Jin-Soo Lee, Joachim Minnich, Joachim Sauter, Joakim Aronsson, Joan Rothenberg, Johan Sanmartin Berglund, John Earl, John Ervin, Jonathan Staub, Jonathan Wilson, José Luiz Neto, José Ramón Méndez Rivas, Jose-Fernando Barba-Gómez, Josef Grosskopf, Juan Carlos Tinoco, Juergen Berger-Roscher, Juergen Schmidt, Juergen Stockhausen, Juergen Wachter, Jukka Markkula, Junya Irimajiri, Jurij Eremenko, Kae Kobayashi, Karl Wilhelm, Karlis Pauksens, Katarina Berndtsson Blom, Ken Heaton, Kenjiro Nakamura, Kyong Ran Peck, Lars Rombo, Lauri Peltonen, Laurie Breger, Lluis Martínez Via, Loïc Boucher, Luciano Goldani, M Luisa Rodrguez de la Pinta, Mahadev Ramjee, Maija Rossi, Manuel Terns Riera, Marc Dionne, Margaret Rhee, Maria Giuseppina Desole, Maria Hemming-Harlo, Maria Maestre Naranjo, Marita Paassilta, Marjaana Sipila, Mark Turner, Marshall Freedman, Marta Aldea Novo, Martin Lundvall, Martin Van Cleeff, Mary Beth Manning, Matthew Finneran, Maximilian Kropp, Megumi Inoue, Meral Esen, Merce Perez Vera, Michael Adams, Michael Mueller, Michael Redmond, Miia Virta, Monika Hamann, Murdo Ferguson, Nell Wyatt, Nicolas Galerne, Nicole Toursarkissian, Niklas Bengtsson, Noah Vale, Olli Henriksson, Otso Arponen, Outi Laajalahti, Pascal Hanrion, Patrice Nault, Patrick Robert, Paul Hartley, Paul Ivan, Paula Gyllemark, Pauliina Paavola, Pavel Kosina, Pavel Naplava, Pekka Koskinen, Pembe Ozunlu, Peter Eizenberg, Peter Gal, Peter Levins, Petr Dite, Philippe Remaud, Piero Barbanti, Pierre André Ferrand, Pierre Lachance, Pierre-Alain Houle, Pyrene Martinez Piera, Ralf Freese, Rie Kuroki, Robert Lipetz, Robert Rosen, Roman Chlibek, Samir Purnell-Mullick, Satu Kokko, Scott Polster, Shari Rozen, Shelly McNeil, Shin Suzuki, Shinn-Jang Hwang, Silvia Narejos Perez, Spyridon Miyakis, Srikanth Malempati, Stephan Morscher, Stephanie Powell, Steve Mueller, Steven Geller, Suganthi Luci Magimaiseelan, Susan Datta, Susanna Koski, Susannah Eyre, Susanne Hoeltz-Roehrig, Suvi-Tuuli Simojoki, Sylvia Shoffner, Takashi Eto, Tamara Eckermann, Tark Kim, Terry Poling, Tetsuhiko Nagao, Thomas Horacek, Thomas Jung, Thomas Weinke, Tiina Haapaniemi, Tiina Karppa, Tiina Korhonen, Tino Schwarz, Tommaso Staniscia, Trevor Gooding, Uwe Kleinecke, Wayne Ghesquiere, Wilfred Yeo, William Ellison, Wilson Jacob, Xavier Farres Fabre, Yieng Huong, Young Goo Song, Yuji Naritomi

**GSK:** Agnes Mwakingwe-Omari, Alemnew F Dagnew, Amy Tan, Ana Strezova, Anne Schuind, Andrew Hastie, Bruno Salaun, Céline Boutry, Emeline de Viron, Emmanuel Di Paolo, Hao Wang, Huizi Zhang, Joon Hyung Kim, Kamal Al Shawafi, Lidia Oostvogels, Mamadou Drame, Martine Douha, Mélanie Gilbert, Meng Shi, Mohamed Amakrane, Mohd Tariq, Nurhan Albayrak, Olivier Godeaux, Paola Pirrotta, Toufik Zahaf

## Eligibility criteria for the ZOE long term follow-up (ZOE-LTFU) study

### Inclusion criteria

- Participants who, in the opinion of the investigator, can and will comply with the requirements of the protocol (e.g., completion of the diary cards, return for follow-up visits, ability to have scheduled contacts to allow evaluation during the study). Or participants with a caregiver who, in the opinion of the investigator, can and will comply with the requirements of the protocol (e.g., completion of the diary cards, availability for follow-up contacts).
- Written informed consent obtained from the participant prior to performance of any study specific procedure.
- Participant who participated in the ZOE-50 or ZOE-70 studies and received at least one dose of recombinant zoster vaccine (RZV).

### Exclusion criteria

- Use of any investigational or non-registered product (pharmaceutical product or device**)** at the time of enrolment or planned use during the study period.
- Previous vaccination against varicella zoster virus (VZV) or herpes zoster (HZ) and/or planned administration during the study of a VZV or HZ vaccine (including an investigational or non-registered vaccine other than the RZV vaccine administered in studies ZOE-50/70).
- Chronic administration (defined as >14 consecutive days in total) of immunosuppressants or other immune-modifying drugs during the period starting 6 months prior to Visit Month 0 of the ZOE-LTFU study or expected administration at any time during the study period. For corticosteroids, this meant prednisone ≥20 mg/day or equivalent. A prednisone dose of <20 mg/day was allowed. Inhaled, topical and intra-articular corticosteroids were allowed.
- Administration of long-acting immune-modifying drugs (e.g., infliximab, rituximab) within 6 months prior to Visit Month 0 of the ZOE-LTFU study or expected administration at any time during the study period.
- Any confirmed or suspected immunosuppressive or immunodeficient condition resulting from disease (e.g. malignancy, human immunodeficiency virus [HIV] infection) or immunosuppressive/cytotoxic therapy (e.g. medications used during cancer chemotherapy, organ transplantation or to treat autoimmune disorders).
- Administration of immunoglobulins and/or any blood products within 3 months prior to Visit Month 0 of the ZOE-LTFU study or planned administration during the study period.
- Prolonged use (>14 consecutive days) of oral and/or parenteral antiviral agents that are active against VZV (acyclovir, valacyclovir, famciclovir, etc.) and planned to be used during the study period for an indication other than to treat suspected or confirmed HZ or an HZ-related complication (topical use of these antiviral agents was allowed).
- Important underlying illness that in the opinion of the investigator would be expected to interfere significantly with the study.

## Primary and secondary objectives

### Primary objective

To assess vaccine efficacy (VE) in the prevention of HZ over the total duration of the ZOE-LTFU study as measured by the reduction in HZ risk overall (i.e. in participants ≥50 years of age at the time of first vaccination in the ZOE-50/70 studies)

### Secondary objectives

To assess VE in the prevention of HZ over the total duration of the ZOE-LTFU study in participants in each age group*

To assess VE in the prevention of HZ from one month post dose 2 in the ZOE-50/70 studies until the end of the ZOE-LTFU study overall and within each age group*

To assess VE in the prevention of HZ over each year of follow-up from one month post dose 2 in the ZOE-50/70 studies overall and within each age group*

To assess VE over the total duration of the ZOE-LTFU study in prevention of post-herpetic neuralgia (PHN) overall and within each age group*

To assess VE in the prevention of PHN from one month post dose 2 in the ZOE-50/70 studies until the end of the ZOE-LTFU study overall and within each age group*

To assess VE over the total duration of the ZOE-LTFU study in prevention of HZ-related non-PHN complications overall and within each age group*

To assess VE in the prevention of HZ-related non-PHN complications from one month post dose 2 in the ZOE-50/70 studies until the end of the ZOE-LTFU study overall and within each age group*

To assess persistence of the humoral immune responses at Year 5, 6, 7, 8, 9 and 10 and beyond after the primary vaccination in the ZOE-50/70 studies in the Humoral Immunogenicity (HI) subset overall and within each age group*

To assess persistence of vaccine induced cell-mediated immune responses at Year 5, 6, 7, 8, 9 and 10 and beyond after the primary vaccination in the ZOE-50/70 studies in the Cell-Mediated Immunogenicity (CMI) subset overall and within each age group*

To assess humoral immune responses at Year 5, 6, 7, 8, 9 and 10 and beyond after the primary vaccination in the ZOE-50/70 studies in the HI subset who had a confirmed HZ episode previously for the timepoint considered

To assess vaccine induced cell-mediated immune responses at Year 5, 6, 7, 8, 9 and 10 and beyond after the primary vaccination in the ZOE-50/70 studies in the CMI subset who had a confirmed HZ episode previously for the timepoint considered

To assess humoral immune responses one month after the first additional RZV dose (1-additional dose and revaccination groups) and at the same timepoint in the control group

To assess vaccine induced cell-mediated immune responses one month after the first additional RZV dose (1-additional dose and revaccination groups) and at the same timepoint in the control group

To assess humoral immune responses one month after the second additional RZV dose (revaccination group) and at the same timepoint in the control group

To assess vaccine induced cell-mediated immune responses one month after the second additional RZV dose (revaccination group) and at the same timepoint in the control group

To assess persistence of humoral immune responses at Year 1, 2, 3, 4, 5 and 6 timepoints of the ZOE-LTFU study in participants from the 1-additional dose, revaccination and control groups

To assess persistence of vaccine induced cell-mediated immune responses at Year 1, 2, 3, 4, 5 and 6 timepoints of the ZOE-LTFU study in participants from the 1-additional dose, revaccination and control groups

To assess vaccine safety and reactogenicity in the 1-additional dose and revaccination groups

To assess vaccine safety in the long term follow-up and control groups

*Age groups were defined as the participants’ age at the time of first vaccination in the ZOE-50/70 studies: 50–59 years, 60–69 years, 70–79 years, ≥80 years, ≥60 years, and ≥70 years

## Definitions of non-PHN complications

HZ vasculitis: Vasculopathy or vasculitis (based on clinical, laboratory or radiologic findings) that is temporally associated with an episode of HZ and, in the opinion of the investigator, was caused directly by the VZV infection arising from the HZ episode.

Disseminated disease: Defined as ≥ 6 HZ lesions outside the primary dermatome as per the investigator’s judgment.

Ophthalmic disease: Defined as HZ affecting any eye structure as per investigator’s judgment.

Neurological disease: Defined as cranial or peripheral nerve palsies, myelitis, meningoencephalitis, stroke, etc. that is temporally associated with an episode of HZ and, in the opinion of the investigator, was caused directly by VZV infection arising from the HZ episode.

Visceral disease: Defined as an abnormality of one or more internal organs (e.g., hepatitis, pneumonitis, gastroenteritis, etc.) that is temporally associated with an episode of HZ and, in the opinion of the investigator, was caused directly by VZV infection arising from the HZ episode.

Stroke A: Diagnosis of stroke requires that criteria 1, 2 and 3 are fulfilled or criteria 1 and 4 and in the opinion of the investigator is temporally associated with an episode of HZ:

- Criterion 1: Rapid onset of localising neurological deficit and/or change in level of consciousness;
- Criterion 2: Localising neurological deficit or change in level of consciousness that lasts greater than 24 hours;
- Criterion 3: No other cerebral process, peripheral lesion, or other disorder is the cause of the localising neurological deficit or change in level of consciousness;
- Criterion 4: Computerised Tomography (CT) scan or Magnetic Resonance Imaging (MRI) scan evidence of an acute thrombotic or haemorrhagic lesion.

## Criteria for According to Protocol (ATP) cohort for analysis of persistence of immunogenicity (LTFU group)

All evaluable participants i.e., those who were included in the ATP cohort for immunogenicity (humoral/CMI) in the primary ZOE-50/70 studies, or were excluded from this cohort solely because they had no blood samples taken, or because of non-compliance with blood sample schedule, and:

- Who did not receive a concomitant medication/ product leading to elimination from the ATP analysis for immunogenicity up to the timepoint considered.
- Who did not present with an intercurrent medical condition leading to elimination from the ATP analysis for immunogenicity (including HZ infection) up to the timepoint considered.
- For whom persistence immunogenicity results were available for the considered time point.

## Safety analysis: protocol amendment

Before Protocol Amendment 2, all adverse events (AEs) or serious adverse events (SAEs) leading to study withdrawal were analysed; after Protocol Amendment 2, only AEs and SAEs leading to withdrawal and related to investigational vaccine, study participation, a concurrent GSK medication or vaccine, as well as HZ-associated complications, were analysed.

## Appendix Table 1. Vaccine efficacy against HZ from 1 month after the second vaccine dose to the end of the ZOE-LTFU study in each study year, overall and by age strata (mTVC)

|  |  |  | | | | | | | | **VE** | | |
| --- | --- | --- | --- | --- | --- | --- | --- | --- | --- | --- | --- | --- |
|  |  | **RZV^1^** | | | | **Placebo/ historical control^2^** | | | |  | **95% (CI)** | |
| **Study year** | **Age strata (years)** | **N** | **n** | **T(year)** | **n/T (per 1000)** | **N** | **n** | **T(year)** | **n/T (per 1000)** | **%** | **LL** | **UL** |
| **Year 1** | 50-59 | 3491 | 1 | 3462·9 | 0·3 | 3523 | 31 | 3475·0 | 8·9 | 96·76 | 80·57 | 99·92 |
|  | 60-69 | 2140 | 0 | 2125·3 | 0·0 | 2166 | 16 | 2142·1 | 7·5 | 100·00 | 79·32 | 100·00 |
|  | 70-79 | 6468 | 1 | 6410·4 | 0·2 | 6554 | 57 | 6466·9 | 8·8 | 98·23 | 89·73 | 99·96 |
|  | ≥80 | 1782 | 1 | 1745·8 | 0·6 | 1792 | 26 | 1739·3 | 14·9 | 96·16 | 76·63 | 99·91 |
|  | ≥60 | 10390 | 2 | 10281·6 | 0·2 | 10512 | 99 | 10348·2 | 9·6 | 97·97 | 92·46 | 99·76 |
|  | ≥70 | 8250 | 2 | 8156·2 | 0·2 | 8346 | 83 | 8206·2 | 10·1 | 97·57 | 90·96 | 99·71 |
|  | Overall | 13881 | 3 | 13744·5 | 0·2 | 14035 | 130 | 13823·3 | 9·4 | 97·68 | 93·07 | 99·53 |
| **Year 2** | 50-59 | 3421 | 2 | 3400·0 | 0·6 | 3427 | 27 | 3385·0 | 8·0 | 92·66 | 70·78 | 99·15 |
|  | 60-69 | 2109 | 1 | 2098·8 | 0·5 | 2113 | 22 | 2087·0 | 10·5 | 95·49 | 72·11 | 99·89 |
|  | 70-79 | 6337 | 5 | 6267·9 | 0·8 | 6345 | 73 | 6236·9 | 11·7 | 93·20 | 83·39 | 97·86 |
|  | ≥80 | 1702 | 2 | 1648·9 | 1·2 | 1679 | 14 | 1623·6 | 8·6 | 85·99 | 39·00 | 98·45 |
|  | ≥60 | 10148 | 8 | 10015·6 | 0·8 | 10137 | 109 | 9947·4 | 11·0 | 92·71 | 85·13 | 96·93 |
|  | ≥70 | 8039 | 7 | 7916·9 | 0·9 | 8024 | 87 | 7860·5 | 11·1 | 92·01 | 82·82 | 96·88 |
|  | Overall | 13569 | 10 | 13415·6 | 0·7 | 13564 | 136 | 13332·5 | 10·2 | 92·69 | 86·15 | 96·57 |
| **Year 3** | 50-59 | 3371 | 0 | 3340·9 | 0·0 | 3351 | 29 | 3319·9 | 8·7 | 100·00 | 89·20 | 100·00 |
|  | 60-69 | 2078 | 0 | 2063·1 | 0·0 | 2062 | 29 | 2025·7 | 14·3 | 100·00 | 89·39 | 100·00 |
|  | 70-79 | 6155 | 8 | 6072·6 | 1·3 | 6104 | 42 | 6001·2 | 7·0 | 81·24 | 59·56 | 92·39 |
|  | ≥80 | 1581 | 1 | 1539·6 | 0·6 | 1557 | 16 | 1487·3 | 10·8 | 93·88 | 60·63 | 99·85 |
|  | ≥60 | 9814 | 9 | 9675·2 | 0·9 | 9723 | 87 | 9514·2 | 9·1 | 89·84 | 79·81 | 95·51 |
|  | ≥70 | 7736 | 9 | 7612·2 | 1·2 | 7661 | 58 | 7488·4 | 7·7 | 84·74 | 68·99 | 93·35 |
|  | Overall | 13185 | 9 | 13016·1 | 0·7 | 13074 | 116 | 12834·0 | 9·0 | 92·36 | 84·98 | 96·59 |
| **Year 4** | 50-59 | 3301 | 1 | 3576·2 | 0·3 | 3272 | 16 | 3534·1 | 4·5 | 93·88 | 60·62 | 99·85 |
|  | 60-69 | 2030 | 2 | 2330·2 | 0·9 | 1978 | 23 | 2243·8 | 10·3 | 91·62 | 66·10 | 99·04 |
|  | 70-79 | 5950 | 5 | 5660·0 | 0·9 | 5863 | 44 | 5557·8 | 7·9 | 88·90 | 72·09 | 96·57 |
|  | ≥80 | 1476 | 2 | 1380·3 | 1·4 | 1404 | 12 | 1301·8 | 9·2 | 84·21 | 29·04 | 98·28 |
|  | ≥60 | 9456 | 9 | 9370·4 | 1·0 | 9245 | 79 | 9103·4 | 8·7 | 88·96 | 77·96 | 95·13 |
|  | ≥70 | 7426 | 7 | 7040·3 | 1·0 | 7267 | 56 | 6859·6 | 8·2 | 87·86 | 73·30 | 95·33 |
|  | Overall | 12757 | 10 | 12946·7 | 0·8 | 12517 | 95 | 12637·4 | 7·5 | 89·75 | 80·31 | 95·24 |
| **Year 6** | 50-59 | 2043 | 2 | 2032·8 | 1·0 | 2043 | 16 | 2032·8 | 7·9 | 87·50 | 46·83 | 98·61 |
|  | 60-69 | 1242 | 1 | 1233·1 | 0·8 | 1242 | 13 | 1233·1 | 10·5 | 92·31 | 48·79 | 99·82 |
|  | 70-79 | 3349 | 5 | 3318·6 | 1·5 | 3349 | 27 | 3318·6 | 8·1 | 81·48 | 51·22 | 94·43 |
|  | ≥80 | 624 | 2 | 606·7 | 3·3 | 624 | 7 | 606·7 | 11·5 | 71·43 | -50·06 | 97·10 |
|  | ≥60 | 5215 | 8 | 5158·4 | 1·6 | 5215 | 47 | 5158·4 | 9·1 | 82·98 | 63·64 | 93·05 |
|  | ≥70 | 3973 | 7 | 3925·3 | 1·8 | 3973 | 35 | 3925·3 | 8·9 | 80·00 | 54·30 | 92·50 |
|  | Overall | 7258 | 10 | 7191·2 | 1·4 | 7258 | 62 | 7191·2 | 8·6 | 83·87 | 68·31 | 92·63 |
| **Year 7** | 50-59 | 2020 | 1 | 2008·6 | 0·5 | 2020 | 15 | 2008·6 | 7·5 | 93·33 | 56·67 | 99·84 |
|  | 60-69 | 1222 | 2 | 1213·3 | 1·6 | 1222 | 13 | 1213·3 | 10·7 | 84·62 | 32·04 | 98·31 |
|  | 70-79 | 3260 | 4 | 3202·6 | 1·2 | 3260 | 27 | 3202·6 | 8·4 | 85·19 | 57·48 | 96·23 |
|  | ≥80 | 581 | 3 | 552·0 | 5·4 | 581 | 5 | 552·0 | 9·1 | 40·00 | -208·39 | 90·68 |
|  | ≥60 | 5063 | 9 | 4967·9 | 1·8 | 5063 | 46 | 4967·9 | 9·3 | 80·43 | 59·54 | 91·58 |
|  | ≥70 | 3841 | 7 | 3754·6 | 1·9 | 3841 | 33 | 3754·6 | 8·8 | 78·79 | 51·24 | 92·08 |
|  | Overall | 7083 | 10 | 6976·4 | 1·4 | 7083 | 61 | 6976·4 | 8·7 | 83·61 | 67·76 | 92·51 |
| **Year 8** | 50-59 | 1998 | 2 | 1985·6 | 1·0 | 1998 | 15 | 1985·6 | 7·6 | 86·67 | 42·67 | 98·52 |
|  | 60-69 | 1198 | 2 | 1189·2 | 1·7 | 1198 | 13 | 1189·2 | 10·9 | 84·62 | 32·04 | 98·31 |
|  | 70-79 | 3140 | 5 | 3067·5 | 1·6 | 3140 | 25 | 3067·5 | 8·1 | 80·00 | 46·81 | 94·02 |
|  | ≥80 | 521 | 1 | 500·7 | 2·0 | 521 | 4 | 500·7 | 8·0 | 75·00 | -152·63 | 99·49 |
|  | ≥60 | 4859 | 8 | 4757·5 | 1·7 | 4859 | 43 | 4757·5 | 9·0 | 81·40 | 59·97 | 92·45 |
|  | ≥70 | 3661 | 6 | 3568·3 | 1·7 | 3661 | 31 | 3568·3 | 8·7 | 80·65 | 52·91 | 93·40 |
|  | Overall | 6857 | 10 | 6743·1 | 1·5 | 6857 | 58 | 6743·1 | 8·6 | 82·76 | 65·98 | 92·14 |
| **Year 9** | 50-59 | 1977 | 5 | 1950·0 | 2·6 | 1977 | 15 | 1950·0 | 7·7 | 66·67 | 3·52 | 90·52 |
|  | 60-69 | 1181 | 3 | 1158·5 | 2·6 | 1181 | 11 | 1158·5 | 9·5 | 72·73 | -3·24 | 95·11 |
|  | 70-79 | 2992 | 6 | 2914·6 | 2·1 | 2992 | 25 | 2914·6 | 8·6 | 76·00 | 40·07 | 91·95 |
|  | ≥80 | 477 | 1 | 443·4 | 2·3 | 477 | 4 | 443·4 | 9·0 | 75·00 | -152·63 | 99·49 |
|  | ≥60 | 4650 | 10 | 4516·5 | 2·2 | 4650 | 42 | 4516·5 | 9·3 | 76·19 | 51·78 | 89·35 |
|  | ≥70 | 3469 | 7 | 3358·0 | 2·1 | 3469 | 29 | 3358·0 | 8·6 | 75·86 | 43·69 | 91·07 |
|  | Overall | 6627 | 15 | 6466·5 | 2·3 | 6627 | 57 | 6466·5 | 8·8 | 73·68 | 52·89 | 86·16 |
| **Year 10** | 50-59 | 1908 | 0 | 1895·2 | 0·0 | 1908 | 15 | 1895·2 | 7·9 | 100·00 | 77·89 | 100·00 |
|  | 60-69 | 1122 | 1 | 1098·5 | 0·9 | 1122 | 10 | 1098·5 | 9·1 | 90·00 | 29·71 | 99·77 |
|  | 70-79 | 2801 | 14 | 2674·9 | 5·2 | 2801 | 22 | 2674·9 | 8·2 | 36·36 | -30·08 | 69·89 |
|  | ≥80 | 408 | 0 | 365·7 | 0·0 | 408 | 4 | 365·7 | 10·9 | 100·00 | -11·47 | 100·00 |
|  | ≥60 | 4331 | 15 | 4139·1 | 3·6 | 4331 | 38 | 4139·1 | 9·2 | 60·53 | 26·55 | 79·83 |
|  | ≥70 | 3209 | 14 | 3040·6 | 4·6 | 3209 | 27 | 3040·6 | 8·9 | 48·15 | -2·41 | 74·87 |
|  | Overall | 6239 | 15 | 6034·3 | 2·5 | 6239 | 53 | 6034·3 | 8·8 | 71·70 | 49·03 | 85·18 |
| **Year 11** | 50-59 | 1883 | 2 | 1867·1 | 1·1 | 1883 | 15 | 1867·1 | 8·0 | 86·67 | 42·67 | 98·52 |
|  | 60-69 | 1075 | 0 | 1064·7 | 0·0 | 1075 | 10 | 1064·7 | 9·4 | 100·00 | 65·07 | 100·00 |
|  | 70-79 | 2563 | 7 | 2469·8 | 2·8 | 2563 | 21 | 2469·8 | 8·5 | 66.67 | 18·61 | 88·30 |
|  | ≥80 | 328 | 0 | 296·1 | 0·0 | 328 | 3 | 296·1 | 10·1 | 100·00 | -71·44 | 100·00 |
|  | ≥60 | 3966 | 7 | 3830·6 | 1·8 | 3966 | 34 | 3830·6 | 8·9 | 79·41 | 52·82 | 92·30 |
|  | ≥70 | 2891 | 7 | 2765·9 | 2·5 | 2891 | 25 | 2765·9 | 9·0 | 72·00 | 33·41 | 89·77 |
|  | Overall | 5849 | 9 | 5697·7 | 1·6 | 5849 | 50 | 5697·7 | 8·8 | 82·00 | 63·03 | 92·22 |

^1^Data from participants in the RZV group of ZOE-50/70 were used for Year 1 to Year 4; data from participants in the LTFU and control groups of the ZOE-LTFU study were used for Year 6 onwards. ^2^Data from participants in the placebo group of ZOE-50/70 were used for Year 1 to Year 4; an historical control formed with data from participants in the placebo group of ZOE-50/70 was used for Year 6 onwards.

Age strata are based on age at first vaccination in the parent studies.

CI, confidence interval; HZ, herpes zoster; LL, lower limit; LTFU, long-term follow-up; mTVC, modified total vaccinated cohort; N, number of participants in each group; n, number of participants with event; n/T, incidence; RZV, recombinant zoster vaccine; T, sum of follow-up; UL, upper limit; VE, vaccine efficacy

##

## Appendix Table 2. Vaccine efficacy against HZ from 1 month after the second vaccine dose to the end of the ZOE-LTFU study: sensitivity analysis including potential HZ cases identified during the gap period (mTVC)

|  | | | **Sensitivity VE results** | | | |
| --- | --- | --- | --- | --- | --- | --- |
| **Assumed positive rate** | **Age strata (years)** | **Mean** | **Min** | **Max** | **Q1** | **Q3** |
| 30% | 50-59 | 90·40 | 89·11 | 91·74 | 90·15 | 90·67 |
|  | 60-69 | 92·02 | 90·72 | 92·57 | 91·95 | 92·57 |
|  | 70-79 | 82·85 | 82·13 | 83·50 | 82·68 | 82·95 |
|  | ≥80 | 85·86 | 83·59 | 86·80 | 84·77 | 86·80 |
|  | ≥60 | 85·79 | 85·34 | 86·13 | 85·66 | 85·97 |
|  | ≥70 | 83·63 | 83·05 | 84·12 | 83·47 | 83·69 |
|  | Overall | 86·92 | 86·86 | 87·00 | 86·88 | 87·00 |
| 40% | 50-59 | 90·07 | 88·53 | 91·23 | 89·63 | 90·66 |
|  | 60-69 | 91·82 | 90·72 | 92·57 | 91·34 | 91·95 |
|  | 70-79 | 82·69 | 82·13 | 83·50 | 82·40 | 82·95 |
|  | ≥80 | 85·46 | 83·59 | 86·80 | 84·62 | 85·79 |
|  | ≥60 | 85·59 | 85·18 | 85·97 | 85·50 | 85·66 |
|  | ≥70 | 83·43 | 82·83 | 83·90 | 83·26 | 83·58 |
|  | Overall | 86·68 | 86·62 | 86·75 | 86·63 | 86·75 |
| 50% | 50-59 | 89·78 | 88·53 | 91·18 | 89·58 | 90·15 |
|  | 60-69 | 91·60 | 90·72 | 92·57 | 91·34 | 91·95 |
|  | 70-79 | 82·54 | 81·85 | 83·23 | 82·40 | 82·68 |
|  | ≥80 | 84·94 | 83·59 | 86·80 | 84·61 | 85·79 |
|  | ≥60 | 85·37 | 85·02 | 85·81 | 85·18 | 85·50 |
|  | ≥70 | 83·21 | 82·83 | 83·90 | 83·05 | 83·26 |
|  | Overall | 86·44 | 86·36 | 86·51 | 86·37 | 86·49 |

Mean = Average VE from 100 simulation

Min = Minimum VE from 100 simulation

Max = Maximum VE from 100 simulation

Q1 = 1st quartile VE from 100 simulation

Q3 = 3rd quartile VE from 100 simulation

Self-reported cases of HZ that occurred during the gap period between the end of ZOE-50/70 and the start of ZOE-LTFU could not be considered as confirmed cases according to protocol-defined criteria. To assess the impact of potentially positive cases, the sensitivity analysis assumed that 30%, 40%, and 50% of self-reported cases would be positive. These assumed positive rates were based on the true positive rates among clinically evaluated suspected cases in the RZV cohorts from the ZOE-50, ZOE-70, and ZOE-LTFU studies: 13%, 35%, and 39%, respectively. These selected positive rates are conservatively high and are deemed suitable to adequately assess the possible influence on VE of cases occurring during the gap period.

Age strata are based on age at first vaccination in the parent studies.

HZ, herpes zoster; LTFU, long-term follow-up; mTVC, modified total vaccinated cohort; RZV, recombinant zoster vaccine; VE, vaccine efficacy

##

## Appendix Table 3. Description of PHN and other HZ-associated complications during ZOE-LTFU (mTVC)

| **Age at time of HZ onset** | **Time between second dose of RZV and HZ onset** | **HZ complication** | **Comorbidities** | **Pain medication** | **Antiviral medication** | **Outcome** |
| --- | --- | --- | --- | --- | --- | --- |
| 81 years | 10 years | PHN | Hypertension, asthma | Lidocaine | Valaciclovir | Ongoing* |
| 78 years | 9 years 3 months | PHN | Hypertension, congestive heart failure, type 2 diabetes, dyslipidaemia, mild renal insufficiency | Dipyrone, gabapentin | Acyclovir | Ongoing* |
| 86 years | 6 years 4 months | PHN | Previous prostate cancer, joint pain, urinary incontinence, erectile dysfunction, pruritus, hypertension | Acetaminophen, codeine, pregabalin | None | Ongoing* |
| 76 years | 9 years 7 months | PHN | Hypothyroidism, dementia, arrhythmia, lumber osteophyte | Acetaminophen | Acyclovir | Ongoing* |
| 78 years | 7 years 3 months | Disseminated HZ | Vascular dementia, end-stage renal disease | Flurbiprofen, gabapentin, lidocaine, acetaminophen | None | Resolved |

*Complication was ongoing at the time of study completion or last contact with the participant

HZ, herpes zoster; LTFU, long-term follow-up; mTVC, modified total vaccinated cohort; PHN, postherpetic neuralgia

## Appendix Table 4. Vaccine efficacy against PHN from 1 month after the second vaccine dose to the end of the ZOE-LTFU study overall and by age strata (mTVC)

|  |  | | | | | | | | **VE** | | |
| --- | --- | --- | --- | --- | --- | --- | --- | --- | --- | --- | --- |
|  | **RZV^1^** | | | | **Placebo/historical control^2^** | | | |  | **95% CI** | |
| **Age strata (years)** | **N** | **n** | **T(year)** | **n/T (per 1000)** | **N** | **n** | **T(year)** | **n/T (per 1000)** | **%** | **LL** | **UL** |
| 50-59 | 3491 | 0 | 25585·3 | 0·0 | 3523 | 15 | 25724·2 | 0·6 | 100·00 | 77·79 | 100·00 |
| 60-69 | 2140 | 1 | 15607·5 | 0·1 | 2166 | 4 | 15660·4 | 0·3 | 74·75 | -155·17 | 99·49 |
| 70-79 | 6468 | 4 | 42211·9 | 0·1 | 6554 | 49 | 42433·5 | 1·2 | 91·79 | 77·60 | 97·85 |
| ≥80 | 1782 | 3 | 9114·1 | 0·3 | 1792 | 9 | 9074·2 | 1·0 | 66·52 | -34·17 | 94·17 |
| ≥60 | 10390 | 8 | 66933·5 | 0·1 | 10512 | 63 | 67168·1 | 0·9 | 87·24 | 73·29 | 94·72 |
| ≥70 | 8250 | 7 | 51326·0 | 0·1 | 8346 | 59 | 51507·7 | 1·1 | 88·08 | 73·87 | 95·41 |
| Overall | 13881 | 8 | 92518·8 | 0·1 | 14035 | 78 | 92892·4 | 0·8 | 89·69 | 78·67 | 95·70 |

^1^Data from participants in the RZV group of ZOE-50/70 were used for Year 1 to Year 4; data from participants in the LTFU and control groups of the ZOE-LTFU study were used for Year 6 onwards. ^2^Data from participants in the placebo group of ZOE-50/70 were used for Year 1 to Year 4 an historical control formed with data from participants in the placebo group of ZOE-50/70 was used for Year 6 onwards.

Age strata are based on age at first vaccination in the parent studies.

CI, confidence interval; HZ, herpes zoster; LL, lower limit; LTFU, long-term follow-up; mTVC, modified total vaccinated cohort; N, number of participants in each group; n, number of participants with event; n/T, incidence; PHN, post-herpetic neuralgia; RZV, recombinant zoster vaccine; T, sum of follow-up; UL, upper limit; VE, vaccine efficacy

## Appendix Table 5. Vaccine efficacy against complications other than PHN from 1 month after the second vaccine dose to the end of the ZOE-LTFU study overall and by age strata (mTVC)

|  |  | | | | | | | | **VE** | | |
| --- | --- | --- | --- | --- | --- | --- | --- | --- | --- | --- | --- |
|  | **RZV^1^** | | | | **Placebo/historical control^2^** | | | |  | **95% CI** | |
| **Age strata (years)** | **N** | **n** | **T(year)** | **n/T (per 1000)** | **N** | **n** | **T(year)** | **n/T (per 1000)** | **%** | **LL** | **UL** |
| 50-59 | 3491 | 0 | 25585·3 | 0·0 | 3523 | 1 | 25736·9 | 0·0 | 100·00 | -1830·98 | 100·00 |
| 60-69 | 2140 | 0 | 15607·7 | 0·0 | 2166 | 5 | 15658·1 | 0·3 | 100·00 | 17·55 | 100·00 |
| 70-79 | 6468 | 2 | 42218·2 | 0·0 | 6554 | 14 | 42472·3 | 0·3 | 85·63 | 37·45 | 98·42 |
| ≥80 | 1782 | 0 | 9121·6 | 0·0 | 1792 | 6 | 9074·7 | 0·7 | 100·00 | 35·34 | 100·00 |
| ≥60 | 10390 | 2 | 66947·5 | 0·0 | 10512 | 26 | 67205·1 | 0·4 | 92·28 | 69·17 | 99·11 |
| ≥70 | 8250 | 2 | 51339·8 | 0·0 | 8346 | 21 | 51547·0 | 0·4 | 90·45 | 60·93 | 98·91 |
| Overall | 13881 | 2 | 92532·8 | 0·0 | 14035 | 28 | 92942·0 | 0·3 | 92·83 | 71·57 | 99·17 |

^1^Data from participants in the RZV group of ZOE-50/70 were used for Year 1 to Year 4; data from participants in the LTFU and control groups of the ZOE-LTFU study were used for Year 6 onwards. ^2^Data from participants in the placebo group of ZOE-50/70 were used for Year 1 to Year 4; an historical control formed with data from participants in the placebo group of ZOE-50/70 was used for Year 6 onwards.

Age strata are based on age at first vaccination in the parent studies.

CI, confidence interval; HZ, herpes zoster; LL, lower limit; LTFU, long-term follow-up; mTVC, modified total vaccinated cohort; N, number of participants in each group; n, number of participants with event; n/T, incidence; PHN, post-herpetic neuralgia; RZV, recombinant zoster vaccine; T, sum of follow-up; UL, upper limit; VE, vaccine efficacy

## Appendix Table 6. Seropositivity rates and GMCs of anti-gE antibody overall and by age (adapted ATP cohort, LTFU group)

|  |  |  | **≥97 mIU/mL** | | | | **GMC** | | |
| --- | --- | --- | --- | --- | --- | --- | --- | --- | --- |
|  |  |  |  |  | **95% CI** | |  | **95% CI** | |
| **Age strata (years)** | **Study year** | **N** | **n** | **%** | **LL** | **UL** | **value** | **LL** | **UL** |
| Overall | Year 5 | 214 | 214 | 100 | 98·3 | 100 | 8043·5 | 7224·9 | 8954·7 |
|  | Year 6 | 786 | 786 | 100 | 99·5 | 100 | 8536·6 | 8113·5 | 8981·7 |
|  | Year 7 | 757 | 757 | 100 | 99·5 | 100 | 8375·1 | 7941·0 | 8833·0 |
|  | Year 8 | 732 | 732 | 100 | 99·5 | 100 | 8231·2 | 7778·2 | 8710·6 |
|  | Year 9 | 641 | 641 | 100 | 99·4 | 100 | 7219·4 | 6803·4 | 7660·9 |
|  | Year 10 | 606 | 606 | 100 | 99·4 | 100 | 6861·5 | 6433·2 | 7318·3 |
|  | Year 11 | 612 | 612 | 100 | 99·4 | 100 | 7039·3 | 6589·0 | 7520·5 |
|  | Year 12 | 435 | 434 | 99·8 | 98·7 | 100 | 6844·3 | 6335·2 | 7394·3 |
| 50-59 | Year 5 | 51 | 51 | 100 | 93·0 | 100 | 8044·2 | 6106·6 | 10596·6 |
|  | Year 6 | 219 | 219 | 100 | 98·3 | 100 | 8584·3 | 7739·9 | 9520·7 |
|  | Year 7 | 215 | 215 | 100 | 98·3 | 100 | 8325·6 | 7483·0 | 9263·1 |
|  | Year 8 | 215 | 215 | 100 | 98·3 | 100 | 8318·0 | 7412·7 | 9333·9 |
|  | Year 9 | 199 | 199 | 100 | 98·2 | 100 | 7097·6 | 6341·6 | 7943·7 |
|  | Year 10 | 197 | 197 | 100 | 98·1 | 100 | 7007·0 | 6228·4 | 7882·9 |
|  | Year 11 | 196 | 196 | 100 | 98·1 | 100 | 7146·9 | 6294·9 | 8114·3 |
|  | Year 12 | 151 | 151 | 100 | 97·6 | 100 | 6737·1 | 5900·9 | 7691·7 |
| 60-69 | Year 5 | 35 | 35 | 100 | 90·0 | 100 | 8715·1 | 6807·0 | 11158·1 |
|  | Year 6 | 230 | 230 | 100 | 98·4 | 100 | 8913·4 | 8155·1 | 9742·3 |
|  | Year 7 | 222 | 222 | 100 | 98·4 | 100 | 8869·6 | 8084·4 | 9731·1 |
|  | Year 8 | 219 | 219 | 100 | 98·3 | 100 | 8680·0 | 7901·2 | 9535·5 |
|  | Year 9 | 195 | 195 | 100 | 98·1 | 100 | 7706·7 | 6994·6 | 8491·4 |
|  | Year 10 | 187 | 187 | 100 | 98·0 | 100 | 7060·8 | 6304·2 | 7908·3 |
|  | Year 11 | 192 | 192 | 100 | 98·1 | 100 | 7159·8 | 6406·3 | 8001·9 |
|  | Year 12 | 161 | 160 | 99·4 | 96·6 | 100 | 6943·4 | 6071·3 | 7940·7 |
| ≥70 | Year 5 | 128 | 128 | 100 | 97·2 | 100 | 7868·7 | 6915·4 | 8953·5 |
|  | Year 6 | 337 | 337 | 100 | 98·9 | 100 | 8258·7 | 7646·0 | 8920·4 |
|  | Year 7 | 320 | 320 | 100 | 98·9 | 100 | 8080·4 | 7446·1 | 8768·7 |
|  | Year 8 | 298 | 298 | 100 | 98·8 | 100 | 7856·6 | 7193·3 | 8581·0 |
|  | Year 9 | 247 | 247 | 100 | 98·5 | 100 | 6951·3 | 6295·8 | 7674·9 |
|  | Year 10 | 222 | 222 | 100 | 98·4 | 100 | 6574·4 | 5913·8 | 7308·8 |
|  | Year 11 | 224 | 224 | 100 | 98·4 | 100 | 6846·3 | 6148·5 | 7623·3 |
|  | Year 12 | 123 | 123 | 100 | 97·0 | 100 | 6848·2 | 5976·0 | 7847·6 |
| ≥60 | Year 5 | 163 | 163 | 100 | 97·8 | 100 | 8043·2 | 7180·7 | 9009·3 |
|  | Year 6 | 567 | 567 | 100 | 99·4 | 100 | 8518·3 | 8036·5 | 9028·9 |
|  | Year 7 | 542 | 542 | 100 | 99·3 | 100 | 8394·8 | 7895·3 | 8925·8 |
|  | Year 8 | 517 | 517 | 100 | 99·3 | 100 | 8195·4 | 7683·4 | 8741·5 |
|  | Year 9 | 442 | 442 | 100 | 99·2 | 100 | 7275·0 | 6784·2 | 7801·3 |
|  | Year 10 | 409 | 409 | 100 | 99·1 | 100 | 6792·5 | 6288·2 | 7337·3 |
|  | Year 11 | 416 | 416 | 100 | 99·1 | 100 | 6989·2 | 6470·8 | 7549·2 |
|  | Year 12 | 284 | 283 | 99·6 | 98·1 | 100 | 6902·0 | 6272·0 | 7595·3 |

Age strata are based on age at first vaccination in the parent studies.

ATP, according to protocol; CI, confidence interval; GMC, geometric mean concentration; LL, lower limit; LTFU, long-term follow-up; N, number of participants in each group; n, number of participants with value; UL, upper limit

##

## Appendix Table 7. Vaccine response rates for anti-gE antibody by age stratum for each year of follow-up (adapted ATP cohort, LTFU group)

|  | | | **Vaccine response rate** | | | |
| --- | --- | --- | --- | --- | --- | --- |
|  | | |  | | **95% CI** | |
| **Age strata (years)** | **Study year** | **N** | **n** | **%** | **LL** | **UL** |
| 50-59 | Year 5 | 51 | 39 | 76·5 | 62·5 | 87·2 |
|  | Year 6 | 219 | 162 | 74·0 | 67·6 | 79·7 |
|  | Year 7 | 215 | 162 | 75·3 | 69·0 | 81·0 |
|  | Year 8 | 215 | 158 | 73·5 | 67·1 | 79·3 |
|  | Year 9 | 199 | 143 | 71·9 | 65·1 | 78·0 |
|  | Year 10 | 197 | 140 | 71·1 | 64·2 | 77·3 |
|  | Year 11 | 196 | 136 | 69·4 | 62·4 | 75·8 |
|  | Year 12 | 151 | 101 | 66·9 | 58·8 | 74·3 |
| 60-69 | Year 5 | 35 | 19 | 54·3 | 36·6 | 71·2 |
|  | Year 6 | 230 | 158 | 68·7 | 62·3 | 74·6 |
|  | Year 7 | 222 | 157 | 70·7 | 64·3 | 76·6 |
|  | Year 8 | 219 | 151 | 68·9 | 62·4 | 75·0 |
|  | Year 9 | 195 | 121 | 62·1 | 54·8 | 68·9 |
|  | Year 10 | 187 | 119 | 63·6 | 56·3 | 70·5 |
|  | Year 11 | 192 | 122 | 63·5 | 56·3 | 70·4 |
|  | Year 12 | 161 | 101 | 62·7 | 54·8 | 70·2 |
| ≥70 | Year 5 | 128 | 74 | 57·8 | 48·8 | 66·5 |
|  | Year 6 | 337 | 216 | 64·1 | 58·7 | 69·2 |
|  | Year 7 | 320 | 201 | 62·8 | 57·3 | 68·1 |
|  | Year 8 | 298 | 187 | 62·8 | 57·0 | 68·3 |
|  | Year 9 | 247 | 145 | 58·7 | 52·3 | 64·9 |
|  | Year 10 | 222 | 120 | 54·1 | 47·3 | 60·7 |
|  | Year 11 | 224 | 131 | 58·5 | 51·7 | 65·0 |
|  | Year 12 | 123 | 75 | 61·0 | 51·8 | 69·6 |
| ≥60 | Year 5 | 163 | 93 | 57·1 | 49·1 | 64·8 |
|  | Year 6 | 567 | 374 | 66·0 | 61·9 | 69·9 |
|  | Year 7 | 542 | 358 | 66·1 | 61·9 | 70·0 |
|  | Year 8 | 517 | 338 | 65·4 | 61·1 | 69·5 |
|  | Year 9 | 442 | 266 | 60·2 | 55·4 | 64·8 |
|  | Year 10 | 409 | 239 | 58·4 | 53·5 | 63·3 |
|  | Year 11 | 416 | 253 | 60·8 | 55·9 | 65·5 |
|  | Year 12 | 284 | 176 | 62·0 | 56·0 | 67·6 |

Age strata are based on age at first vaccination in the parent studies.

Vaccine response rate: percentage of participants with post-vaccination anti-gE antibody concentration ≥4-fold the cut-off value for initially seronegative participants and ≥4-fold the pre-vaccination value for initially seropositive participants

ATP, according to protocol; CI, confidence interval; LL, lower limit; LTFU, long-term follow-up; N, number of participants in each group; n, number of participants with value; UL, upper limit

## Appendix Table 8. Mean geometric increase of anti-gE antibody by age stratum for each year of follow-up (adapted ATP cohort, LTFU group)

|  |  |  |  |  | **MGI** | | |
| --- | --- | --- | --- | --- | --- | --- | --- |
|  |  |  |  |  |  | **95% CI** | |
| **Age strata (years)** | **Study year** | **N** | **GMC** | **Pre-vaccination GMC** | **Value** | **LL** | **UL** |
| 50-59 | Year 5 | 51 | 8044·2 | 902·5 | 8·91 | 6·54 | 12·15 |
|  | Year 6 | 219 | 8584·3 | 1018·6 | 8·43 | 7·25 | 9·80 |
|  | Year 7 | 215 | 8325·6 | 1006·1 | 8·28 | 7·12 | 9·62 |
|  | Year 8 | 215 | 8318·0 | 1011·3 | 8·23 | 7·03 | 9·62 |
|  | Year 9 | 199 | 7097·6 | 967·7 | 7·33 | 6·26 | 8·59 |
|  | Year 10 | 197 | 7007·0 | 967·7 | 7·24 | 6·17 | 8·50 |
|  | Year 11 | 196 | 7146·9 | 995·9 | 7·18 | 6·09 | 8·45 |
|  | Year 12 | 151 | 6737·1 | 1012·4 | 6·65 | 5·55 | 7·98 |
| 60-69 | Year 5 | 35 | 8715·1 | 1510·6 | 5·77 | 3·85 | 8·65 |
|  | Year 6 | 230 | 8913·4 | 1265·4 | 7·04 | 6·14 | 8·09 |
|  | Year 7 | 222 | 8869·6 | 1287·3 | 6·89 | 6·00 | 7·91 |
|  | Year 8 | 219 | 8680·0 | 1280·9 | 6·78 | 5·89 | 7·80 |
|  | Year 9 | 195 | 7706·7 | 1330·1 | 5·79 | 4·98 | 6·74 |
|  | Year 10 | 187 | 7060·8 | 1286·9 | 5·49 | 4·69 | 6·42 |
|  | Year 11 | 192 | 7159·8 | 1283·5 | 5·58 | 4·79 | 6·50 |
|  | Year 12 | 161 | 6943·4 | 1246·7 | 5·57 | 4·69 | 6·61 |
| ≥70 | Year 5 | 128 | 7868·7 | 1586·1 | 4·96 | 4·10 | 6·00 |
|  | Year 6 | 337 | 8258·7 | 1465·7 | 5·63 | 5·10 | 6·23 |
|  | Year 7 | 320 | 8080·4 | 1448·9 | 5·58 | 5·02 | 6·19 |
|  | Year 8 | 298 | 7856·6 | 1444·1 | 5·44 | 4·85 | 6·10 |
|  | Year 9 | 247 | 6951·3 | 1487·0 | 4·67 | 4·13 | 5·29 |
|  | Year 10 | 222 | 6574·4 | 1452·2 | 4·53 | 3·95 | 5·19 |
|  | Year 11 | 224 | 6846·3 | 1432·0 | 4·78 | 4·21 | 5·43 |
|  | Year 12 | 123 | 6848·2 | 1327·9 | 5·16 | 4·37 | 6·09 |
| ≥60 | Year 5 | 163 | 8043·2 | 1569·6 | 5·12 | 4·32 | 6·08 |
|  | Year 6 | 567 | 8518·3 | 1380·9 | 6·17 | 5·68 | 6·70 |
|  | Year 7 | 542 | 8394·8 | 1380·4 | 6·08 | 5·59 | 6·62 |
|  | Year 8 | 517 | 8195·4 | 1372·5 | 5·97 | 5·46 | 6·53 |
|  | Year 9 | 442 | 7275·0 | 1415·6 | 5·14 | 4·67 | 5·66 |
|  | Year 10 | 409 | 6792·5 | 1374·1 | 4·94 | 4·46 | 5·48 |
|  | Year 11 | 416 | 6989·2 | 1361·4 | 5·13 | 4·65 | 5·66 |
|  | Year 12 | 284 | 6902·0 | 1281·3 | 5·39 | 4·78 | 6·08 |

Age strata are based on age at first vaccination in the parent studies.

MGI: ratio of GMC in study year/GMC pre-vaccination.

ATP, according to protocol; CI, confidence interval; GMC, geometric mean concentration; LL, lower limit; LTFU, long-term follow-up; MGI, mean geometric increase; N, number of participants in each group; UL, upper limit

##

## Appendix Table 9. Frequency of gE-specific CD4[2+] T cells by age stratum for each year of follow-up (adapted ATP cohort, LTFU group)

| **Age strata (years)** | **Study year** | **N** | **N (missing)** | **Mean** | **SD** | **Minimum** | **Q1** | **Median** | **Q3** | **Maximum** |
| --- | --- | --- | --- | --- | --- | --- | --- | --- | --- | --- |
| 50-59 | Year 5 | 2 | 1 | 858·92 | 639·37 | 406·8 | 406·8 | 858·9 | 1311·0 | 1311·0 |
|  | Year 6 | 39 | 1 | 1174·70 | 1103·76 | 170·5 | 522·0 | 800·6 | 1473·0 | 5296·4 |
|  | Year 7 | 39 | 0 | 1141·00 | 1074·96 | 69·8 | 455·0 | 915·2 | 1454·0 | 5363·1 |
|  | Year 8 | 37 | 2 | 1217·84 | 1155·53 | 176·5 | 425·4 | 914·0 | 1735·4 | 5437·4 |
|  | Year 9 | 34 | 4 | 1403·78 | 1435·49 | 219·7 | 565·0 | 1114·2 | 1703·5 | 7096·9 |
|  | Year 10 | 35 | 2 | 1210·28 | 1216·92 | 2·5 | 358·2 | 1043·9 | 1586·3 | 5924·4 |
|  | Year 11 | 36 | 2 | 1113·90 | 1317·91 | 51·4 | 456·7 | 718·9 | 1248·6 | 7020·6 |
|  | Year 12 | 32 | 2 | 1030·37 | 971·86 | 41·4 | 452·2 | 764·4 | 1476·3 | 5363·9 |
| 60-69 | Year 5 | 0 | 1 | NA | NA | NA | NA | NA | NA | NA |
|  | Year 6 | 38 | 3 | 839·31 | 644·97 | 1·0 | 253·3 | 688·4 | 1202·4 | 2710·4 |
|  | Year 7 | 37 | 3 | 745·36 | 572·57 | 1·0 | 349·9 | 591·9 | 1074·6 | 2128·2 |
|  | Year 8 | 38 | 2 | 737·78 | 602·91 | 66·2 | 282·7 | 621·7 | 907·7 | 2512·8 |
|  | Year 9 | 35 | 4 | 930·89 | 796·20 | 98·5 | 309·2 | 750·0 | 1307·2 | 3792·2 |
|  | Year 10 | 31 | 3 | 952·86 | 763·05 | 1·0 | 320·4 | 684·4 | 1531·8 | 2916·5 |
|  | Year 11 | 33 | 2 | 884·65 | 712·45 | 1·0 | 329·1 | 596·2 | 1364·4 | 3069·0 |
|  | Year 12 | 29 | 3 | 828·62 | 666·18 | 46·4 | 253·8 | 697·8 | 1459·9 | 2222·7 |
| ≥70 | Year 5 | 1 | 2 | 376·16 | NA | 376·2 | 376·2 | 376·2 | 376·2 | 376·2 |
|  | Year 6 | 23 | 1 | 474·23 | 515·96 | 1·0 | 130·5 | 345·3 | 554·9 | 2206·9 |
|  | Year 7 | 24 | 0 | 577·87 | 828·60 | 1·0 | 83·9 | 338·5 | 634·0 | 3398·0 |
|  | Year 8 | 22 | 0 | 629·18 | 827·35 | 1·0 | 66·5 | 348·5 | 724·1 | 3030·3 |
|  | Year 9 | 15 | 4 | 801·54 | 1033·67 | 1·0 | 162·7 | 416·7 | 1076·4 | 3486·1 |
|  | Year 10 | 14 | 3 | 670·87 | 726·96 | 27·7 | 182·7 | 358·3 | 1110·2 | 2522·0 |
|  | Year 11 | 14 | 0 | 445·15 | 597·48 | 1·0 | 55·0 | 225·3 | 649·9 | 2254·3 |
|  | Year 12 | 12 | 0 | 443·65 | 531·44 | 1·0 | 132·6 | 235·6 | 576·1 | 1941·7 |
| ≥60 | Year 5 | 1 | 3 | 376·16 | NA | 376·2 | 376·2 | 376·2 | 376·2 | 376·2 |
|  | Year 6 | 61 | 4 | 701·66 | 621·26 | 1·0 | 252·8 | 511·6 | 981·4 | 2710·4 |
|  | Year 7 | 61 | 3 | 679·46 | 683·15 | 1·0 | 230·5 | 540·3 | 775·7 | 3398·0 |
|  | Year 8 | 60 | 2 | 697·96 | 688·76 | 1·0 | 227·0 | 496·4 | 847·0 | 3030·3 |
|  | Year 9 | 50 | 8 | 892·09 | 865·29 | 1·0 | 284·1 | 590·3 | 1238·2 | 3792·2 |
|  | Year 10 | 45 | 6 | 865·13 | 755·35 | 1·0 | 318·9 | 580·3 | 1262·4 | 2916·5 |
|  | Year 11 | 47 | 2 | 753·74 | 703·75 | 1·0 | 215·5 | 512·4 | 1298·8 | 3069·0 |
|  | Year 12 | 41 | 3 | 715·95 | 647·90 | 1·0 | 222·3 | 526·3 | 959·5 | 2222·7 |

Age strata are based on age at first vaccination in the parent studies.

ATP, according to protocol; LTFU, long-term follow-up; N, number of participants in each group; N (missing), number of participants with missing value; NA, not applicable; Q1, first quartile; Q3, third quartile; SD, standard deviation
